# Optimizing the preparation of labeled *N*-glycans for rapid, simplified, and high-precision analysis

**DOI:** 10.1371/journal.pone.0336565

**Published:** 2025-12-16

**Authors:** Riko Makino, Shunji Natsuka

**Affiliations:** Department of Life and Food Sciences, Graduate School of Science and Technology, Niigata University Ikarashi-nino-cho, Nishi-ku, Niigata, Japan; University of Mississippi, UNITED STATES OF AMERICA

## Abstract

Glycan structures hold promise as biomarkers for the early detection of diseases, owing to their sensitive reflection of cellular states. However, glycan analysis remains complex and time-consuming, and the use of hazardous chemicals in traditional hydrazinolysis methods presents a significant barrier to broader application and cross-disciplinary research. To enable efficient glycan biomarker discovery, we aimed to develop a simple and accurate *N*-glycan analysis method capable of high-throughput sample processing. A key feature of this study is the use of pyridylamination, a fluorescent labeling technique that enables high isomer separation efficiency in reversed-phase LC/MS. After comparing various methods for *N*-glycan release and purification, we identified Rapid PNGase F (New England Biolabs) and BlotGlyco (Sumitomo Bakelite) as optimal for this application. To improve accuracy by reducing artifact formation, the BlotGlyco protocol was modified from the manufacturer’s original instructions. Using this optimized workflow, we successfully analyzed human serum, human urine, and CHO-K1 membrane fractions, demonstrating that distinct glycan structures in each sample type could be effectively separated and quantified. This work supports the broader adoption of glycan analysis across diverse research fields.

## Introduction

Glycosylation is one of the major post-translational modifications of proteins. *N*-glycans are initially transferred to nascent proteins in the endoplasmic reticulum (ER), and their structures are subsequently modified by a series of glycosidases and glycosyltransferases as they pass through the Golgi apparatus from ER. The initial high-mannose glycans are trimmed and processed into hybrid-type and complex-type structures. These reactions are influenced not only by the activity of enzymes but also by the availability of sugar transporters and substrates. As a result, glycan structures are highly heterogeneous—even a single protein species can carry multiple glycoforms [1]. This diversity, known as “microheterogeneity”, is believed to modulate protein function [2]. Glycan structures are known to significantly impact various biological processes [3], including the immunogenicity, efficacy, proteolytic resistance, solubility, and half-life of therapeutic proteins [1,4,5].

Due to their sensitivity to environmental and physiological changes, glycans vary in their monosaccharide composition, linkages, branching patterns, and anomeric configurations. This variability has drawn attention to glycans as potential biomarkers. The field of glycomics, which aims to comprehensively characterize glycan structures and their biological roles, is gaining prominence as it enables precise profiling of cells. This is particularly critical for early disease diagnosis and for ensuring the quality of cells used in regenerative medicine. In short, glycomics is becoming indispensable for the advancement of accessible and personalized medical care.

One example of glycan biomarkers is sialyl Lewis A (SLe^a^, also known as CA19-9) and sialyl Lewis X (SLe^x^) [6]. SLe^a^, a tetrasaccharide found on glycoproteins and glycolipids, is elevated mainly in pancreatic diseases. It serves as a ligand for selectins on vascular endothelial cells and is implicated in cancer metastasis and lesion vascularization [7]. However, SLe^a^ alone is insufficient for definitive diagnosis or for screening asymptomatic individuals. Although it shows relatively high levels in early-stage pancreatic ductal adenocarcinoma, its positivity rate remains low until late stages in biliary tract and gastric cancers. Furthermore, patients who are Lewis antigen-negative (Lewis-negative genotype) cannot express the SLe^a^ epitope, limiting its utility [8]. Given these limitations, there is a strong need to identify ideal glycan biomarkers—those that are detectable at early disease stages, have low false-positive rates, offer prognostic and metastatic insights, and can be measured in minimally invasive samples such as blood or urine.

To identify such ideal biomarkers, it is essential to comprehensively and precisely characterize glycan structures and their relative abundances in biological samples. Due to the vast structural diversity of glycans, including isomers, manual analysis is no longer feasible. While major glycan structures have been characterized, biomarker candidates present at low abundance or specific to early disease stages remain elusive. Moreover, integrating glycomics with proteomics is critical for advancing glycobiology, but the technical complexity and time-consuming nature of glycan analysis remain barriers to entry. Discovering trace-level glycan structures through more precise, accurate, and streamlined analyses would significantly expand biomarker research.

Recent efforts have aimed to automate glycan preparation and LC/MS analysis [9]. Given the immense structural diversity of branched glycans, databases are essential for cohort studies. Although various glycan databases prepared from mass analysis exist, most include only structural information, *m/z* values, sugar compositions estimated from them, and sample origin. These are not sufficient for distinguishing isomers in unknown samples, especially when relying solely on mass spectrometry data.

In this study, we utilized pyridylamination to fluorescently label glycans. Since one pyridylamino (PA-) residue links to each glycan molecule and its fluorescent intensity is scarcely affected by differences in the structure of the glycans [10], fluorescence intensity enables quantitative analysis. Additionally, PA-residue carry a positive charge under acidic conditions, making PA-glycans highly suitable for MS analysis in positive mode and identification of MS/MS fragments from their reducing end. Most importantly, due to their fine-tuned hydrophobicity, PA-glycans allow for the separation of structural isomers—including epimers and anomers—by reversed-phase (RP) LC [11,12]. This enables the detailed structural elucidation and quantification of complex glycan mixtures in a single RP-LC-MS/MS run. While other fluorescent labels such as 2-aminobenzamide (2-AB) and 2-anthranilic acid (2-AA) are widely used, they lack the capacity for effective isomer separation due to their relatively high hydrophobicity [13]. Similarly, MALDI MS offers rapid analysis but cannot resolve isomers. Our RP-LC/MS approach, optimized for PA-glycans, enables high-resolution isomer separation in a single run and is thus well-suited for precise multi-sample analysis.

Against this background, we first developed a rapid, simple, and highly accurate method for preparing PA-labeled *N*-glycans. We then refined the method to minimize artifacts and contaminants. Using the improved protocol, we analyzed *N*-glycan structures in human serum, urine, and CHO-K1 cell membrane fractions to evaluate the method’s applicability across diverse biological samples. Comparative analysis revealed that the method combining Rapid PNGase F and BlotGlyco was the simplest and most accurate for processing multiple serum samples. Furthermore, the improved method—enhanced for artifact reduction—enabled the sensitive identification of various glycan species, including multi-sialylated, hyper-branched, and sulfated glycans, from healthy human urine samples.

## Materials and methods

### Materials

A human serum pool was obtained from Cosmo Bio Inc. (Tokyo, Japan), in which the protein concentration was 66.1 mg/mL. Human urine samples were from healthy volunteers in the early morning (male, age is in his twenties), designated M1 and M2, in which protein concentration was 39.48 µg/mL for M1, and 68.48 µg/mL for M2. Urine samples were stored at −20°C and thawed for protein concentration measurement and glycan analysis, respectively. This study received approval from the Ethics Committee of Niigata University (Approval No. 2024−0192). All participants were provided with a written explanation of the study procedures, and written informed consent was obtained prior to their enrollment. Recruitment of volunteers was begun on November 27, 2024. CHO-K1 cells were cultured in α-modified Eagle’s medium (α-MEM) supplemented with 10% fetal calf serum, 100 μg/mL streptomycin, and 100 U/mL penicillin G. at 37°C in a humidified atmosphere of 5% CO_2_. Isolation of membrane fraction is described in supporting information. In this study, the term water refers to ultrapure water.

### *N*-glycans release by hydrazinolysis

Hydrazinolysis was performed as previously reported [14], with some modifications. 0.2 mL of anhydrous hydrazine (Tokyo Chemical Industry Co., Ltd., Tokyo) was added to a lyophilized 1 µL of human serum pool and heated at 100°C for 12 hours. The hydrazine was then removed by vacuum evaporation, followed by co-evaporation with 100 µL of toluene several times for complete removal. After that, re-*N*-acetylation of the amino groups was performed on ice with ice-cold saturated sodium hydrogen carbonate aqueous in 4% (v/v) acetic anhydride. Further desalting was performed by 1 g of Dowex 50Wx2 (H^+^ form).

### *N*-glycans release by PNGase F and rapid PNGase F

The enzymatic digestions by PNGase F and Rapid PNGase F were performed according to the manufacturer’s protocol (details are described in supporting information). For comparison of preparation methods, samples were 1 µL of human serum (protein content 66.1 µg). For verification of sample adaptability, samples were 3 µL of human serum (protein content 198.3 µg), 50.0 µg of human urinary protein, 267.4 µg of CHO-K1 microsomal fraction (protein content 54.7 µg). Urinary protein was extracted with methanol/chloroform extraction (water: MeOH: chloroform = 4: 4: 1) by the regular method.

### Glycan purification by conventional method

Fluorescent labeling of the reducing end of the glycan was performed by reductive amination as previously reported [4,5]. In Brief, pyridylamination was conducted by the addition of 20 µL of fluorescent labeling reagent (138 mg 2-aminopyridine, 50 µL acetic acid) to lyophilized sample, and heating to 80°C for 60 min, 110 µL of reduction reagent (100 mg dimethylamine borane, 40 µL acetic acid, 25 µL water) was added and the Schiff base was reduced at 70°C for 35 min. Excess labeling reagent was removed by twice of water-saturated-phenol/chloroform (1:1 (v/v)) extractions, once chloroform extraction, and gel filtration (1 x 8 cm, TSK-gel Toyopearl HW-40F; Tosoh Corporation, Tokyo). After that, the sample was further purified by graphite carbon solid-phase extraction (GL-Pak Carbograph 150 mg, GL Sciences Inc., Tokyo). Eluent was lyophilized, and dissolved in 100 µL of water, and then stored at −20°C for LC/MS analysis.

### Glycan purification by BlotGlyco

The following procedures were performed essentially according to the manufacturer’s protocol, but with some modifications. Modified steps are marked with an asterisk (*), and detailed protocols including these changes are available on Protocols.io [15]. As a result of the improvement from manufacturer’s protocol, the peak tailing of contaminants was reduced, and the baseline was lowered (data not shown). This made it easier to confirm the fluorescence peaks of glycans. In mass spectrometry, the matrix effect in the ion trap was also mitigated.

50 µL of polymer beads suspension in the column was washed with 100 µL of water, twice* (Unless otherwise noted, washing was done as spin down using a mini centrifuge at room temperature). After that, the enzymatically digested sample solution was added to the column, heated with 180 µL of 2% acetic acid in acetonitrile (ACN) at 80°C for 60 min so that hydrazide groups on the beads captured *N*-glycans. Next, the beads with captured *N*-glycans were washed twice each in 200 µL of 2 M guanidine hydrochloride aqueous, 200 µL of water, and 200 µL of 1% triethylamine in methanol to remove peptides and contaminants. At this time, the reagent and beads were well pipetted to ensure metamorphic action was spread through the mixture*. Thereafter, glycans were released from the beads as follows; first, free hydrazide groups on the beads were *N*-Acetylated capped by 100 µL of 10% acetic anhydride in methanol at room temperature. At this time, the reagent and beads were pipetted well*. The glycans were liberated stoichiometrically by heating in 20 µL of water and 180 µL of 2% acetic acid in ACN, at 70°C for 90 min.

Next, labeling of *N*-glycans was performed by reductive amination using 30 µL of fluorescent labeling reagent (138 mg of 2-aminopyridine, 50 µL of acetic acid), 80°C for 60 min. Subsequently, Schiff base was reduced by the addition of 110 µL of reduction reagent (100 mg of dimethylamine borane, 40 µL acetic acid, 25 µL water), at 70°C for 35 min. PA-*N*-glycans were recovered from the beads by centrifuging at 13,200 rpm for 1 min and purified by HILIC, a normal-phase chromatography using a polar stationary phase in which the column contained in the BlotGlyco kit was conditioned with water and ACN, and sample solution was prepared in a 90% ACN solution, and then loaded to the column. After the solution was completely loaded*, it was then centrifuged at 2,000 rpm for 1 min. Subsequently, the column was washed with 95% ACN in water twice by centrifuged at 2,000 rpm for 1 min. And then centrifuged at 13,200 rpm for 30 sec to remove the fluid. Finally, purified PA-*N*-glycans were eluted with 25 µL of water, centrifuged at 2,000 rpm for 1 min and 13,200 rpm for 30 sec*, and further eluted with 25 µL of 0.1% acetic acid aqueous by centrifugation at 2,000 rpm for 1 min and 13,200 rpm for 1 min*. Collected solution was lyophilized, and dissolved in 100 µL of water, and then stored at −20°C for LC/MS analysis.

### Removal of artificially added *O*-Acetyl groups

To detach artificial *O*-acetyl groups (*O*-Ac), 200 µL of 0.28% aqueous ammonium was added to lyophilized eluent of HILIC and heated at 70°C for 30 min. To simplify, 200 µL of 0.40% aqueous ammonium was added to the eluent of HILIC and heated at 70°C for 30 min. Reacted solution was lyophilized, and dissolved in 100 µL of water, and then stored at −20°C for LC/MS analysis.

### Reversed phase LC-MS/MS

Samples were analyzed with ESI-MS on an LTQ XL linear ion trap mass spectrometer coupled to a Dionex Ultimate 3000 HPLC system (Thermo Scientific, Franklin, MA), as described in the literature [16] with slight modification. PA-*N*-glycan was dissolved in 100 µL of water, and 20 µL of this solution was used for analysis. PA-*N*-glycan were eluted with 0.2% (v/v) formic acid in water (solvent A) and 20% ACN in 0.2% formic acid (solvent B), separated on reversed-phase using InertSustain AQ-C18 column (3 µm, 0.21 x 15 cm, GL Sciences Inc., Tokyo, Japan) as described in the literature [17]. In brief, elution was performed by HPLC under gradient elution conditions using 0.2% formic acid (Eluent A) and 20% acetonitrile in 0.2% formic acid (Eluent B). The column was equilibrated with Eluent A, and 3 min after sample injection, the proportion of Eluent B was increased linearly from 0 to 25% over 100 min, followed by an increase to 100% over 5 min. Fluorescence detection was performed in online connection with MS, and the eluate, after separation on the column, was split and analyzed by both the fluorescence detector (FLD) and the MS device.

### Data analysis

LC-MS/MS data were analyzed using Xcalibur 2.2 (Thermo Fisher Scientific). In the glycan structure analysis, the elution time in RP/LC of the PA-glycans was normalized in addition to the MS and MS/MS data. Two types of normalization were performed: conversion to the RP scale using the core structure of PA-*N*-glycans [18] and conversion to the glucose unit scale using PA-isomalto-oligosaccharides [10]. In addition, the partial elution time of the glycan structure [19].

The quantification of PA-*N*-glycans was based on the peak area in the RP-LC fluorescence chromatogram. When multiple glycans were present on the same peak, the fluorescence peak area was distributed by the intensity ratio of MS. Symbol nomenclature was notated according to the international guideline Symbol Nomenclature for Glycans (SNFG). The notation and abbreviations of glycan structures are described in S1 Fig in S1 File.

## Results and discussion

### Comparison of methods for PA-*N*-Glycan preparation

#### Comparison of methods for Glycan liberation.

Three glycan liberation methods were compared: hydrazinolysis, PNGase F digestion and Rapid PNGase F digestion. Rapid PNGase F requires only 10 minutes of reaction time, significantly shorter than the 60 minutes needed for conventional PNGase F digestion and the 12 hours for hydrazinolysis. Subsequent labeling and purification were performed using a combination of multiple columns, hereafter referred to as “conventional method”. The comparison focused on the following five aspects:

1. Yield ratio:

The yield of each method was assessed based on the area of the double-α2,6-NeuAc double-antennary glycan (66N-BI, S1 Fig in S1 File. (f)), the most abundant glycan in serum, as observed in fluorescence chromatograms. A higher yield indicates a better detection of minor glycans and enables more comprehensive MS/MS analysis, making the method more promising.

1. Comprehensiveness:

To evaluate whether glycan yield varied with structural features such as the number of sialic acids or molecular mass, the relative yields of major *N*-glycans were compared. The yield of each glycan was normalized to the 66N-BI yield, which was set to 100%.

1. Artifact formation:

Artifacts such as epimers, glycosylamine (GA), and *O*-acetylated (*O*-Ac) derivatives can arise during sample preparation. These were evaluated by examining artifacts derived from 66N-BI. Epimers result from C2-epimerization of the unlabeled reducing terminal sugar (GlcNAc to ManNAc) via keto-enol tautomerization under alkaline conditions (S2 Fig in S1 File). GA refers to the *N*-glycoside form at the reducing terminal, with a mass two units smaller, and weaker fluorescence due to altered electron density on PA (S2 Fig in S1 File). Therefore, the ratio of GA formation was calculated from florescence and mass chromatography. *O*-Ac refers to acetylation of a hydroxyl group and occurs somewhat in *N*-acetylation process. Naturally occurring *O*-acetylated glycans (e.g., 9-*O*-Ac NeuAc) have been reported, but in very small amounts and indistinguishable from artifacts in this method.

1. Time and simplicity:

Duration was defined as the total time from sample preparation to LC/MS analysis. Simplicity was evaluated based on experimental time, number of steps, equipment required, and use of hazardous reagents.

1. Adaptability to high-throughput processing:

Methods were assessed for suitability in large-scale experiments based on scalability, mechanization compatibility, and feasibility of simultaneous sample preparation.

**Fig 1:**
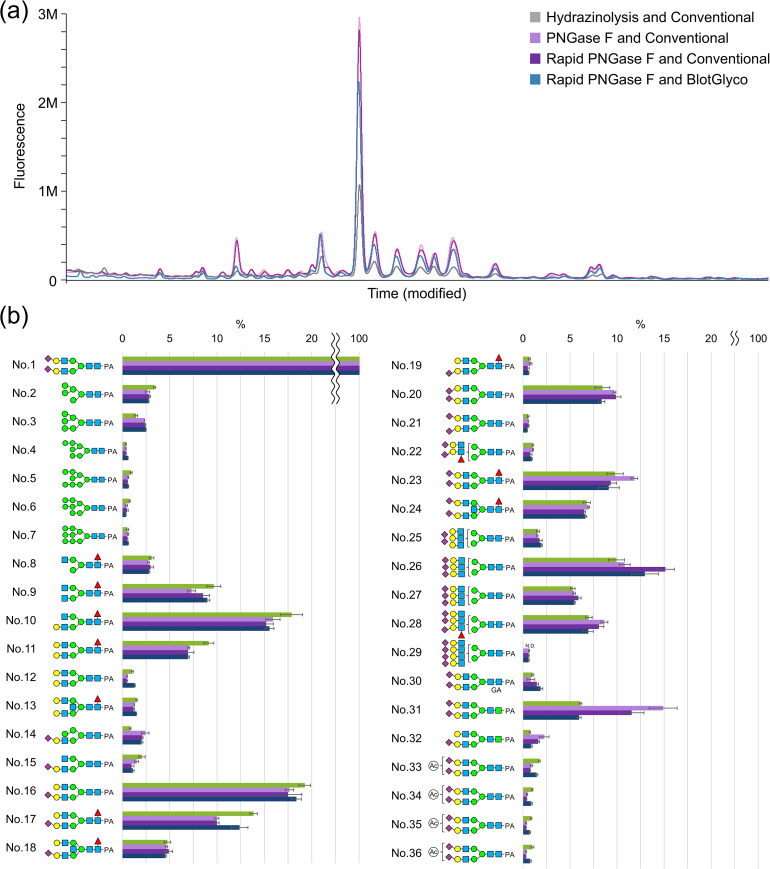
Comparative analysis of glycosylation and fluorescent labeling/purification methods. **(a)** Fluorescence chromatograms obtained by RP-LC analysis. The vertical axis represents fluorescence intensity, and the horizontal axis represents elution time. **(b)** Comparison of the yield ratios of major glycans. The horizontal axis indicates the structures of the major glycans, and the vertical axis shows the relative yield, with the 66N-BI glycan yield set to 100% for each method. 66N-BI is the most abundant glycan structure found in serum. Glycans No. 33–36 are artificially *O*-acetylated.

Experimental results and comparative analyses are shown in Fig 1, and glycan profiles are summarized in S1 Table in S2 File. When the yield of 66N-BI in hydrazinolysis was normalized to 1.00 ± 0.08, the yields in PNGase F and Rapid PNGase F were 2.97 ± 0.24 (mean ± SE) and 2.90 ± 0.22, respectively. F-tests revealed significant differences between hydrazinolysis and PNGase F (p < 0.01) and between hydrazinolysis and Rapid PNGase F (p < 0.001), but no significant difference between PNGase F and Rapid PNGase F (p = 0.70).

Regarding comprehensiveness, Fig 1(b) shows that variations in glycan ratio due to sialic acid number or glycan mass were generally within experimental error. However, certain trends were observed:

- Hydrazinolysis method yielded slightly higher amounts of glycans No. 9–12 (asialo-complex-type substructures) and No. 16–17 (mono-sialylated complex-type substructures). This is possibly due to slight de-sialylation by the process.
- Contrary, tri-sialylated tri-antennary (trN-TR, No. 26) had lower yields, and tetra-sialylated tetra-antennary (teN-TE, No. 29) glycans had undetectable in hydrazinolysis method, again likely due to partial de-sialylation and chemical degradation.

These results suggest that PNGase F-based methods may better preserve highly sialylated and branched glycans, reflecting their *in vivo* distribution.

The detection of artifact products showed:

- Epimers (No. 31, 32) were more prevalent in enzymatic methods. PNGase F digestion, with its long reaction time, produced 15.2% epimers, compared to 6.1% in hydrazinolysis. Rapid PNGase F showed an intermediate rate of 11.7%.
- Detection of GA form (No. 30) *via* fluorescence was difficult due to its weak fluorescence yield. Mass spectrometry revealed no significant differences among methods (Table 1).
- *O*-Ac artifacts (No. 33–36) were nearly twice as abundant in hydrazinolysis. This is likely due to the re-*N*-acetylation step required after hydrazinolysis. In addition, the use of BlotGlyco has also increased *O*-Ac artifacts. This is still likely due to the *N*-acetylation step, which is necessary for capping the hydrazide groups on the beads.

**Table 1:** Comparison results for each method.

Liberation		Hydrazinolysis	PNGase F	Rapid PNGase F	Rapid PNGase F
Labeling & Purification		Conventional	Conventional	Conventional	BlotGlyco
**Yield Ratio** ^**a**^		1.00 ± 0.08	2.97 ± 0.24	2.90 ± 0.22	2.35 ± 0.04
**Artifact** ^**b**^	***O*-acetylation**	1.7%	0.9%	0.8%	1.5%
	**Epimer**	6.1%	15.2%	11.7%	6.0%
	**Glycosylamine** **(FLD, MS)**	1.0% 12.3%	0.8% 11.4%	1.5% 15.0%	1.9% 8.3%
**Duration**		4 days	3 days	3 days	6 hours
**Simplicity**		△	〇	〇	◎
**High-throughput Processing**		△	〇	〇	◎
**Note**		• Also analyze *O*-glycan • Deleterious substance • Peeling reaction			• Suitable for small samples • Expensive

(a) The yield ratio was determined based on the yield of 66N-BI, the most abundant glycan in serum. Yield ratios are expressed as relative values (mean ± SE), with the combination of hydrazinolysis and conventional methods set to a mean of 1.00.

(b) The presence ratio of each artifact was expressed as the ratio of the structure derived from 66N-BI to the original 66N-BI.

In terms of method simplicity: Hydrazinolysis involves the use of explosive hydrazine for 12 hours, followed by hydrazine removal using a cold trap and an oil rotary vacuum pump. The process also requires re-*N*-acetylation of hexosamines and subsequent desalting via cation exchange. Overall, it takes several days to prepare LC/MS-ready samples from serum using this method. In contrast, the enzymatic methods are simpler. The reaction time is 60 minutes for PNGase F and only 10 minutes for Rapid PNGase F—both significantly shorter than hydrazinolysis. Furthermore, the required equipment is minimal: a heat block, a centrifuge, and standard pipettes.

Based on these findings, we concluded that the enzymatic methods are superior to hydrazinolysis in terms of practicality, safety, and efficiency, despite concerns over the higher epimerization rates. Among the two enzymatic options, Rapid PNGase F, which exhibits a lower level of epimerization, was deemed the most promising. Further comparisons were therefore focused on fluorescent labeling and purification methods, which will be discussed in the following section.

#### Comparison of fluorescent labeling and purification methods.

Two samples from the same serum pool were prepared, and glycans were released using Rapid PNGase F, then labeled and purified using both the conventional method employed in our laboratory and BlotGlyco, a glycan sample preparation kit. The BlotGlyco protocol was modified from the manufacturer’s instructions to improve impurity removal and enhance signal-to-noise ratios. Human serum and urine can contain hundreds of distinct *N*-glycan structures, with abundance levels varying by up to 1000-fold. Therefore, minimizing noise is crucial for the high-sensitivity detection of minor glycan species.

The conventional method requires several days due to multiple lyophilization steps. In contrast, BlotGlyco enables labeling and purification within 6 hours.

Experimental results and comparative analyses are shown in Fig 1, with the glycan profiles in S1 Table in S2 File. The yield obtained using the combination of Rapid PNGase F and BlotGlyco was approximately 30% lower than that achieved with Rapid PNGase F and the conventional purification method, a statistically significant difference confirmed by F-test (p < 0.01).

Regarding comprehensiveness, a comparison of the major glycan ratio indicated that yield differences related to factors such as the number of sialic acids and glycan mass were generally within the margin of error (Fig 1(b)). Glycan yields for structures No. 12 and No. 17 using Rapid PNGase F and BlotGlyco were slightly higher than using Rapid PNGase F and the conventional method, while that of No. 20 was slightly lower.

In terms of artifact formation, Rapid PNGase F combined with BlotGlyco produced approximately half the number of epimers compared to the conventional method, likely because the reducing ends were promptly linked on the beads after enzymatic release. However, the *O*-acetylation (*O*-Ac) rate was about twice that observed with the conventional method, likely due to *N*-acetylation step for capping the hydrazide groups on the beads prior to glycan release.

Additionally, Rapid PNGase F and BlotGlyco combination is highly suited for processing multiple samples, requiring less time, equipment, and materials, and is compatible with 96-well plate formats.

Based on these findings, we conclude that the Rapid PNGase F-BlotGlyco method is superior for labeling and purifying *N*-glycans. Comparative results of the preparation methods are shown in Table 1. The combination of Rapid PNGase F and BlotGlyco was selected due to its high accuracy, simplicity, and suitability for processing multiple samples simultaneously.

**Fig 2:**
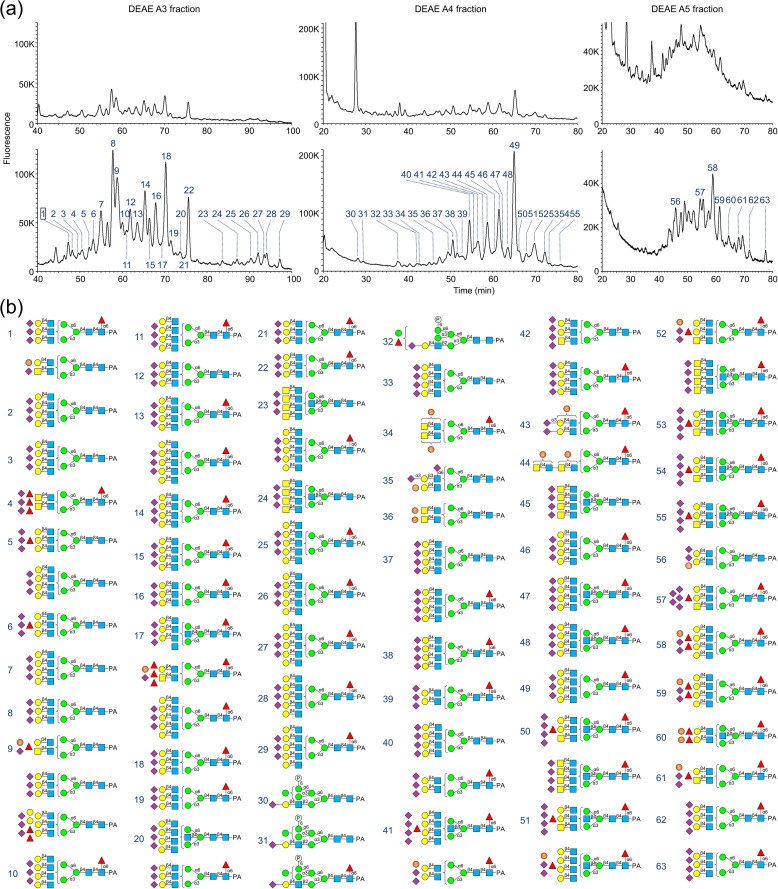
Comparison of the number of glycan species in the A3, A4, and A5 fractions separated by DEAE-HPLC using different preparation methods. Purified samples from healthy human urine (M1) were fractionated by DEAE-HPLC based on the number of negative charges to determine the diversity of glycan species. PA-glycans were prepared from 50 μg of urinary protein, and a portion equivalent to 24 μg of protein was subjected to RP-LC-MS/MS analysis. The glycan profiles are provided in S2 Table in S2 File. **(a)** Fluorescence chromatograms obtained by RP-LC analysis. The upper panel shows the results from the combination of hydrazinolysis and the conventional method, and the lower panel shows the results from the combination of rapid PNGase F and BlotGlyco. **(b)** Glycan structures identified in the lower panel of **(a)**.

*N*-Glycans of human urine proteins (sample M1) were prepared using either hydrazinolysis followed by a conventional method or the Rapid PNGase F-BlotGlyco method. These were then fractionated by anion-exchange HPLC based on negative charge (experimental details are provided in Supporting Information and S3 Fig in S1 File) and analyzed by RP-LC-MS/MS (Fig 2 (a)). Compared to the hydrazinolysis-conventional method, the Rapid PNGase F-BlotGlyco method yielded more trN- and teN-glycans (Fig 2 (b)). Contrary to expectations, hydrazinolysis method resulted in a low yield of multi-sialylated *N*-glycans, rendering it unsuitable for their analysis.

#### Removal of artificial *O*-Ac groups.

**Fig 3:**
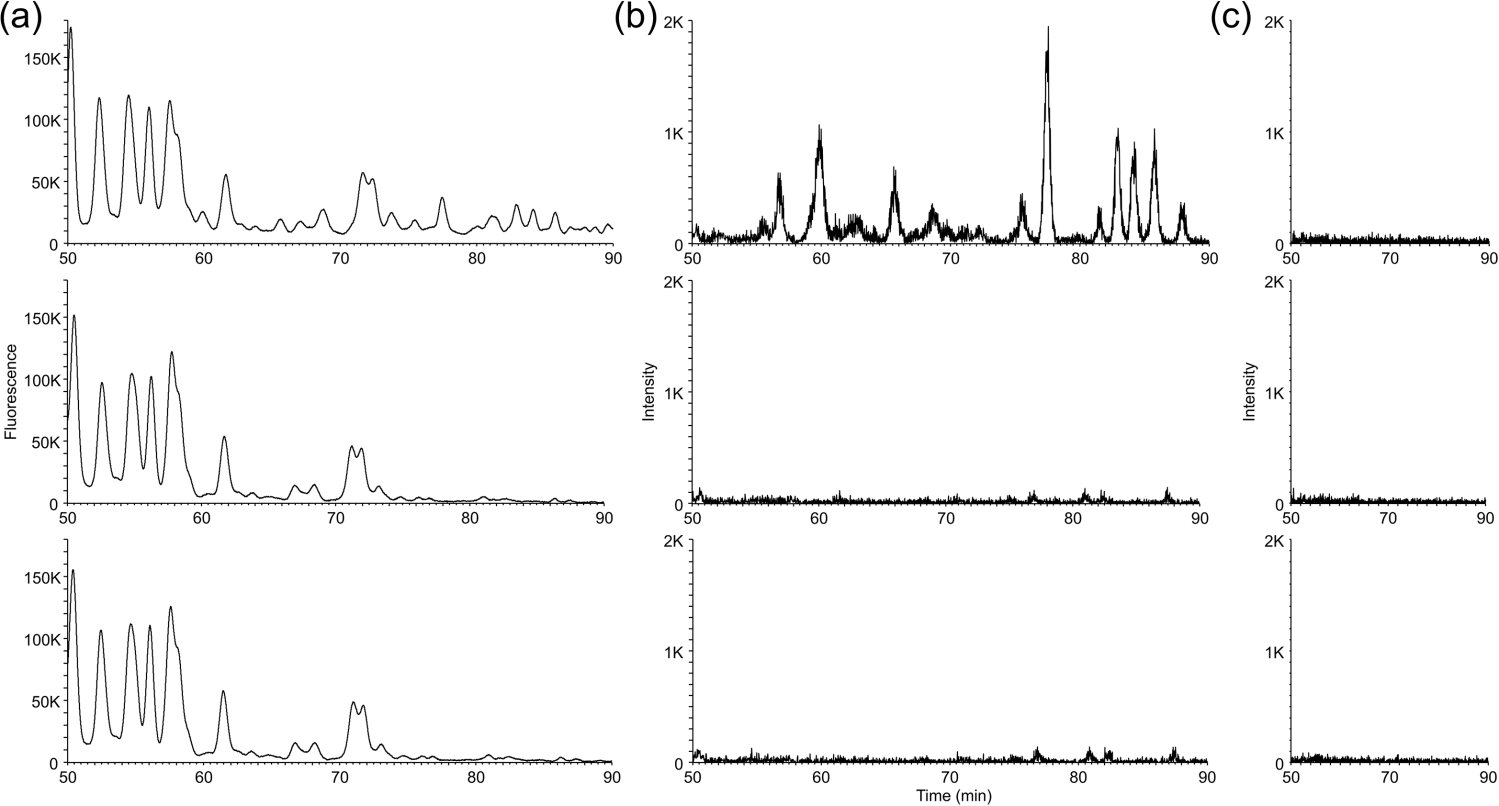
Results of *O*-acetyl group removal by aqueous ammonium treatment. HILIC eluates of *N*-glycans from a human serum pool, prepared using rapid PNGase F and BlotGlyco, were either lyophilized and then heated at 70°C for 30 min in 200 µL of 0.28% aqueous ammonium, or directly heated at 70°C for 30 min after the addition of 200 µL of 0.40% aqueous ammonium without lyophilization. From top to bottom: original sample; lyophilized and treated with 0.28% solution; treated with 0.40% solution without lyophilization. **(a)** Fluorescence chromatogram obtained by RP-LC analysis. **(b)** Mass chromatogram of the [M + 2H]^2+^ ion (*m/z* = 1172.43) corresponding to *O*-acetylated dN-BI artifacts (*m/z* range: 1171.93–1174.43). **(c)** Mass chromatogram of the [M + 2H]^2+^ ion of dN-BI (*m/z* = 1139.46) with excessive deacetylation at one site (*m/z* range: 1138.96–1141.46).

The removal of *O*-Ac groups by ester hydrolysis under mild basic conditions was investigated. PA-glycan samples (each 50 µL) of prepared from a pooled human serum with Rapid PNGase F and BlotGlyco were lyophilized and then heated in 0.28% aqueous ammonium or added 200 µL of 0.40% aqueous ammonium directly to the aliquot and heated. The fluorescence chromatogram of RP-LC is shown in Fig 3(a), and the comparison of *O*-Ac-double-NeuAc (dN)-BI artifacts by mass chromatogram is presented in Fig 3(b). As shown in Fig 3 (b), all *O*-Ac-dN-BI artifacts completely disappeared under both treatments. Furthermore, the fluorescence peaks corresponding to the elution times of these *O*-Ac-dN-BI artifacts also disappeared. In addition, Fig 3 (c) demonstrates that the *N*-acetyl groups were scarcely removed, indicating that excessive deacetylation did not occur. To simplify the experimental procedure, we adopted the method of detaching *O*-Ac groups by adding 200 µL of 0.40% aqueous ammonium to 50 µL of HILIC eluate following the use of BlotGlyco.

### Sample adaptability

Glycan structural analyses of three sample types were performed using the improved method: pooled healthy human serum, healthy human urine, and the CHO-K1 membrane fraction. The *N*-glycan profile of serum was readily obtained due to its solubility and high protein concentration (60–80 mg/mL). Glycans in human serum are mainly derived from plasma cells and hepatocytes; however, trace amounts of proteins originating from the entire body are also present, making serum a useful systemic biomarker source through precise glycan analysis [20].

Glycans in healthy human urine originate from both serum and urinary organs, but the protein concentration (20–40 μg/mL) is less than one-thousandth that of serum. Urine is an excellent biological sample due to the non-invasive, qualification-free collection, and the availability of large volumes. Since protein expression and glycosylation modifications vary between organs depending on disease onset and progression, urine glycan analysis provides insight into physiological and pathological conditions across multiple organs [21].

CHO-K1 is a cell line derived from Chinese hamster ovary and is a mainstay in the industrial production of recombinant proteins due to its rapid growth, robustness, and mutagenesis suitability resulting from its non-diploid genome [22]. Quality control of recombinant proteins is crucial to avoid immunogenicity caused by non-human epitopes [23]. The membrane fraction includes various membrane structures, excluding the nuclear membrane.

Artifacts were observed as Y ions derived from degradation products caused by in-source decay (ISD), occurring simultaneously with or immediately after ionization, and post-source decay (PSD), occurring after ionization (S4 Fig (a) in S1 File). ISD is the internal fragmentation of ions due to excess energy during ionization [24]. While PSD is fragmentation that occurs after ions leave the ionization chamber, either due to excess internal energy or collisions with residual deactivation gas [25]. In this study, artifacts from ISD or PSD are referred to as “frag”. Additionally, a structure consisting of two glycans linked with three cations was observed (S4 Fig in S1 File), referred to here as “ag”. The number of glycan artifacts described below includes epimers, GA, and formylation products (described later), which are generated during experimental process and exhibit different elution times from the original glycans. However, frag and ag are not included, as they are generated during ionization of MS analysis.

#### Human serum.

**Fig 4:**
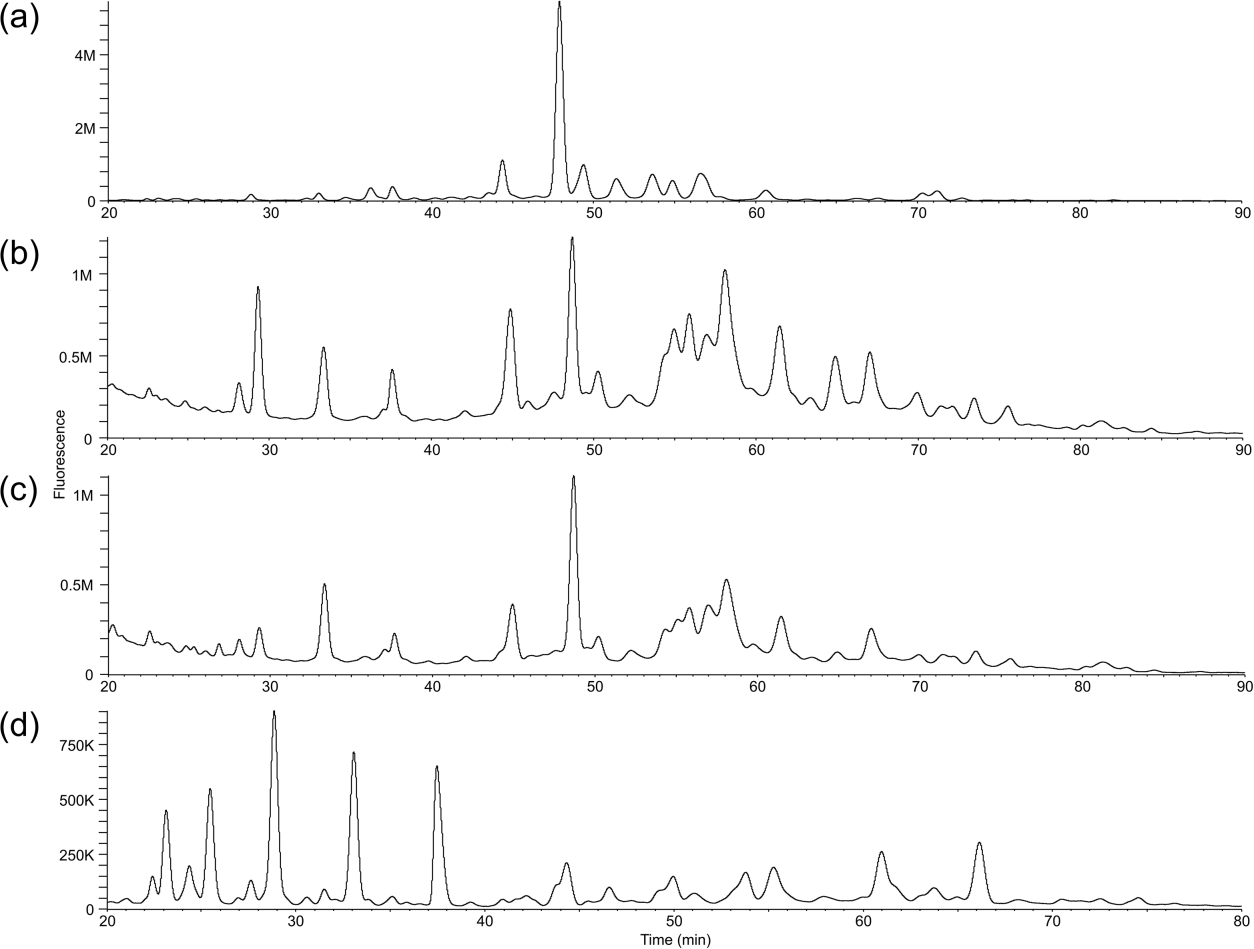
RP-LC chromatograms of samples purified using the improved method. (a) human serum; (b) human urine M1; (c) human urine M2; **(d)** CHO-K1 membrane fraction.

**Fig 5:**
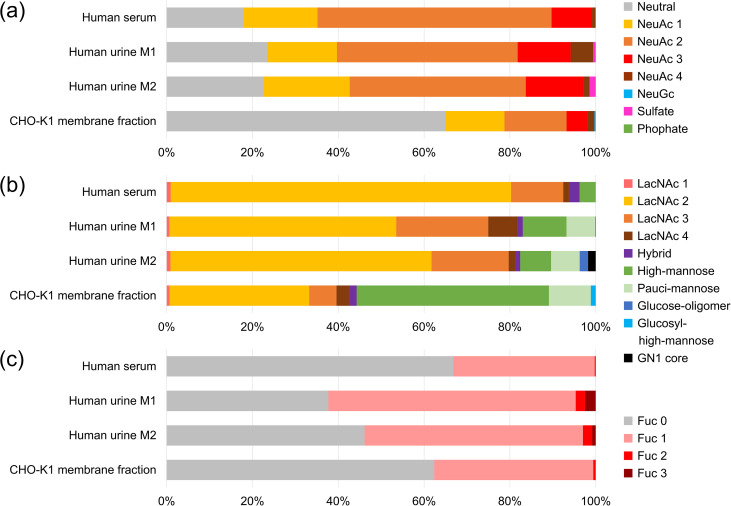
Glycan analysis of samples purified using the improved method. **(a)** Classification based on the number of negative charges on glycans. The number of sialic acids and the corresponding yields are shown in the graph. “NeuGc” indicates the presence of one or more NeuGc residues; “Sulfate” indicates the presence of one or more sulfate groups; “Phosphate” indicates the presence of one or more phosphate groups. **(b)** Structural profile. The structural types and their corresponding yields are shown in the graph. **(c)** Fucosylation profile.

**Fig 6:**
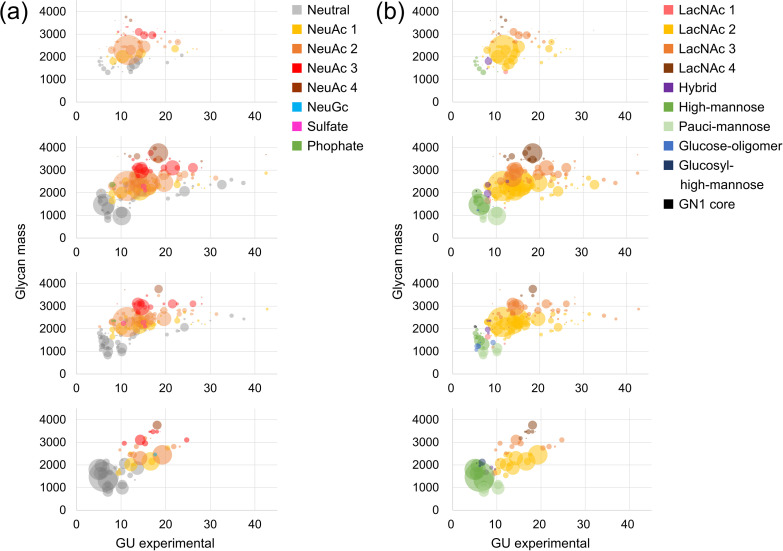
Characteristics of each sample shown in scatter plots. The vertical axis represents glycan mass, and the horizontal axis represents the elution time standardized by glucose units (GU). The size of each circle reflects the yield of glycans within a sample; however, the sizes are relative within each sample and cannot be directly compared across samples. **(a)** Plot classified by the number of negative charges. **(b)** Plot classified by structural type.

The *N*-glycan profile of the pooled healthy human serum using the improved method is shown in Figs 4(a), 5, and 6, and summarized in S3 Table in S2 File. Three microliters of the serum pool (corresponding to 198.3 µg of protein) were used, and a portion equivalent to 99.2 μg of protein was subjected to RP-LC-MS/MS analysis.

In the human serum, 73 glycan structures matched the database of Sugar Scan (MÖBIUS Co., Ltd.), and an additional 23 structures were presumed to be intact glycans. Furthermore, 35 artifacts, including frag, GA, epimer, *O*-Ac, and formylation, were identified.

Human serum contained abundant complex-type glycans with two LacNAc units, and numerous structures with three or four LacNAc units, which are minor in blood, were also identified. Many of them were sialylated. It has been reported that dN-BI structures, abundant in serum, are mainly derived from α1-antitrypsin, hemopexin, and transferrin [26]. Most LacNAc units were presumed to be Type 2 (Galβ1–4GlcNAc), which predominates in humans. Some MS/MS spectra showed fragment ions at *m/z* = 731.26 [M + H]^+^, suggesting tandem LacNAc binding (Galβ1–4GlcNAcβ1–3Galβ1–4GlcNAc, Table 2).

**Table 2:** Partial structure of *N*-glycan prepared from each sample.

	Human serum	Human urine M1	Human urine M2	CHO-K1 membrane fraction
**Lewis X**	–	0.63	0.36	2.36
**sialyl Lewis X**	2.56	6.29	5.25	–
**Lewis Y**	–	0.49	0.53	–
**LacdiNAc**	0.18	3.27	6.12	0.56
**tandem LacNAc binding**	1.39	9.52	2.53	2.57
**disialylated NeuAc**	0.23	4.41	1.69	–
**bisecting GlcNAc**	5.86	7.39	6.77	2.67
**core Fucose**	29.44	50.71	45.23	29.29

The numbers indicate the percentage of total glycans for each method. “–” indicates not detected.

Asialo-complex-type glycans in blood are thought to be rapidly removed by hepatocyte asialoglycoprotein receptors, which preferentially bind highly branched complex-type glycans [27]. Consequently, highly branched asialo-complex-type glycans were scarcely detected. Indeed, the plasma half-life of intravenously administered recombinant human EPO (rhEPO) is reported to be 5–6 hours for sialylated rhEPO, compared to less than 2 minutes for desialylated rhEPO [28]. Consistent with this, serum complex-type glycans were predominantly sialylated.

Agalactosyl-BI (lacking galactose, S1 Fig (d) and (e) in S1 File), BI, sialyl-BI, and BI with bisecting GlcNAc were abundant, consistent with glycans typically found on the IgG Fc region (Asn^297^). The glycan at Asn^297^ has been reported to affect antibody activity, dynamics, and safety [29,30]. The presence of core fucose significantly reduces antibody-dependent cellular cytotoxicity (ADCC) by 50- to 100-fold [31,32], while bisecting GlcNAc enhances ADCC activity [33,34]. Moreover, increased Gal residues promote complement-dependent cytotoxicity via enhanced C1q binding [35]. In this study, core fucose was present in 29.44% of total glycans, and bisecting GlcNAc in 5.86%.

Sialyl-TR, sialyl-TE, core fucose, and Lewis X (Le^x^) were also identified, presumably derived from liver α1-acid glycoprotein (α1-AGP, orosomucoid) [36]. Large amounts of dN-BI and trN-TR, likely from ferritin, were detected, along with several low-abundance TE structures. Thus, this method enables detailed analysis of complex and trace multi-branched structures.

High-mannose glycans were present at low levels. Reported high-mannose glycoproteins include complement component C3, IgM, and α2-macroglobulin [37,38]. High-mannose glycans in blood are rapidly cleared by mannose receptors on liver Kupffer cells, which bind fungal mannans [39,40]. Consistent with this, few high-mannose glycans were detected in serum.

Formylated M5A was identified as a new modified structure. Formylation has been reported to occur time-dependently between glycan hydroxyl groups and formic acid during glycan preparation [41]. Formic acid was included in the LC/MS solvent in this method, it remains unclear whether formylation occurred during the LC/MS process.

The human plasma *N*-glycan profile is known to be highly stable with minimal intra-individual variation [42]. Furthermore, for the non-invasive exploration of biomarkers, subsequent analyses used human urine, which contains glycoproteins derived from both serum and urinary system organs.

#### Human urine.

The *N*-glycan profiles of healthy human urine obtained using the improved method are shown in Figs 4, 5 and 6, and summarized in S4 and S5 Table in S2 File. PA-glycans were prepared from 50 μg of urinary protein, and a portion equivalent to 24 μg of protein was subjected to RP-LC-MS/MS analysis.

In M1 urine sample, 84 structures matched entries in the glycan database, and an additional 73 structures were presumed to be intact glycans. Moreover, 32 artifacts, including GA and epimer, were identified. In sample M2, 84 structures also matched the database, 63 were presumed intact glycans, and 34 artifacts were identified.

The glycan profile of human urine—both in terms of structure and relative abundance—showed partial similarity to that of human serum. However, features distinguishing it from serum, such as Lewis antigens, sulfated glycans, LacdiNAc, and glucose oligomers were also observed (Table 2, Figs 5 and 6). For example, sialyl Lewis X (SLe^x^) was present at 6.29% in M1, 5.25% in M2, and 2.56% in serum. Le^x^ and Lewis Y (Le^y^) were each detected at 0.49% in M1 and 0.53% in M2 but were not detected in serum. LacdiNAc was present at 3.27% in M1, 6.12% in M2, and 0.56% in serum. Some variations were observed between M1 and M2, such as the detection of disialylated LacNAc (NeuAcα2–3Galβ1–3(NeuAcα2–6)GlcNAc) or tandem LacNAc binding. It is important to note that some glycan structures may have gone undetected due to a lack of MS/MS data or absence from the database.

Uromodulin, also known as Tamm-Horsfall glycoprotein, is the most abundant glycoprotein in human urine. It is a transmembrane protein produced in the thick ascending limb of the loop of Henle and in the nephrons of the distal tubules and is secreted into the urine following dissociation from its glycosylphosphatidylinositol (GPI) membrane anchor [43]. The brush border of distal tubular epithelial cells is susceptible to contact with pathogens such as bacteria and viruses, many of which adhere *via* high-mannose-glycan specific lectins. Uromodulin is thought to prevent such adhesion by acting as a decoy receptor [44,45]. It has been reported that a high mannose-type sugar chain is added to Asn^251^ on uromodulin [46], and the length of the high mannose-type sugar chain on uromodulin causes changes in its binding to type 1-fimbriated E. coli [47]. It has been also reported that uromodulin carries complex-type glycans such as sialyl-BI and sialyl-TR in addition to sulfate groups [48], consistent with our findings.

Fucose and Le^x^ were found at significantly higher levels in both urine samples compared to serum. Among α1,3-fucosyltransferases (FUT3–7 and 9) responsible for Le^x^ synthesis, FUT4, 5, and 7 are expressed in the human kidney and are reported to contribute to Le^x^ biosynthesis [49]. Thus, Le^x^ is likely derived from urogenital glycoproteins. In terms of core fucosylation by FUT8, 50.71% and 45.23% of glycans in M1 and M2, respectively, contained core fucose, both of which were higher than the 29.44% observed in serum.

Numerous pauci-mannose structures (glycans with four or fewer mannose residues) were identified. Although rare in vertebrates, pauci-mannose is the major glycan type found in quail ovomucoid [50]. In invertebrates, these are generated by specific *N*-acetylglucosaminidase that hydrolyze the sugar residue added by *N*-acetylglucosaminyltransferase-I [51]. In vertebrates, lysosomal *N*-acetylhexosaminidase B is believed to catalyze their formation [52]. Pauci-mannose glycans have been detected in mammals and may be upregulated in conditions such as cancer, inflammation, and stem cell development [1].

LacdiNAc, a motif found in glycoprotein hormones like thyroid-stimulating hormone, plays important roles in metabolism. Sulfated LacdiNAc has been shown to influence the plasma half-life of pituitary glycoprotein hormones [53]. One sulfated structure, SO₄-4GalNAcβ1–4GlcNAcβ1-, is rapidly cleared from blood by the mannose receptor on liver Kupffer cells [33], suggesting its urinary presence originates from uromodulin rather than serum proteins. Sulfated LacdiNAc motifs such as SO₄-3Galβ1–4GlcNAcβ1- and SO₄-4GalNAcβ1–4GlcNAcβ1-, as well as 2–4 antenna complex-type glycans, have been reported in uromodulin [33,54].

The disialylated LacNAc structure (NeuAcα2–3Galβ1–3(NeuAcα2–6)GlcNAc) is likely generated by α2,3-sialylation of type 1 LacNAc *via* ST3GAL, followed by α2,6-sialylation by ST6GALNAC6. When this disialylated LacNAc is further α1,4-fucosylated by FUT3, it becomes disialyl Le^a^ [55].

Several phosphorylated glycans were identified, which are believed to be mannose-6-phosphate (M6P) modifications originating from the *cis*-Golgi. Lysosomal enzymes are initially modified with GlcNAc-1-phosphate, which is subsequently processed to M6P by α-*N*-acetylglucosaminidase. M6P serves as a lysosomal targeting signal by binding to mannose-6-phosphate receptors (M6PR) in the trans-Golgi and mediating transport *via* vesicles to lysosomes. A hybrid-type glycans with M6P were also confirmed. The frequent detection of hybrid-type M6P glycans may be attributed to their weak binding affinity to M6PR [56], especially when only one M6P residue is present, resulting in secretion rather than lysosomal targeting. Previous studies have reported the presence of various lysosomal proteins in human urine [57] and the presence of M6P residues on uromodulin have also been reported [58].

Finally, glucose oligomers were detected in urine sample treated by methanol precipitation, but not in serum. The glucans observed in urine are presumed to originate either from the liver, concentrated through urinary excretion, or directly from the kidney. The kidney, like the liver, is known to store glycogen.

#### CHO-K1 membrane fraction.

The *N*-glycan profile of the CHO-K1 membrane fraction obtained using the improved method is shown in Figs 4(d), 5, and 6, and summarized in S6 Table in S2 File. PA-glycans were prepared from 267.4 μg of membrane fraction, corresponding to 54.7 μg of protein, and a portion equivalent to 27.4 μg of protein was subjected to RP-LC-MS/MS analysis. In total, 43 glycan structures matched entries in the glycan database, and an additional 14 were presumed to be intact glycans. Five artifact structures, including GA, and epimers, were also identified.

Several glucosylated high-mannose glycans—precursors of *N*-glycans in the ER—were identified in the CHO-K1 membrane fraction. High-mannose glycans showed both greater structural diversity and higher abundance than in serum or urine. This likely reflects the presence of ER and Golgi membranes in the membrane fraction, where these glycan precursors accumulate.

Notably, *N*-glycolylneuraminic acid (NeuGc), which is not synthesized in humans, was detected. Although NeuGc expression is low in CHO-K1 cells, its presence can impact the immunogenicity and efficacy of therapeutic glycoproteins [59]. NeuGc can also be taken up by human cells and incorporated into cell surface glycans [60]. This method demonstrated high sensitivity in detecting low-abundance NeuGc, highlighting its potential utility in quality control of biopharmaceuticals. This method allows determination of the overall structure of glycans containing low-abundance NeuGc and is considered applicable to the quality control of biopharmaceuticals.

In addition to NeuGc, glycan substructures were identified that are thought to require glycosyltransferases not normally expressed in CHO-K1 cells. For example, although CHO-K1 lacks α1,3-fucosyltransferase (namely Le^x^ synthase) expression [61]. Le^x^ containing structures were detected, accounting for 2.36% of total glycans. Likewise, despite reported deficiencies in β-1,4-N-acetylglucosaminyltransferase III (GnT-III) activity [62], bisecting GlcNAc structures were found at an abundance of 2.67%.

Finally, regarding sialylated glycans, CHO-K1 cells lack β-galactoside α2,6-sialyltransferase [63], suggesting that the detected NeuAc residues are predominantly α2,3-linked. Indeed, no structures consistent with α2,6-sialylation were found when compared against the glycan database.

While a wide variety of glycan species were detected, a substantial number of ions with unknown structures were also observed. MS/MS analysis indicated that nearly half of these ions were peptide-containing species (S5 Fig in S1 File). Many displayed features characteristic of peptides, such as neutral losses corresponding to amino acid residues, and fragment ions resulting from dehydration or primary amine elimination. However, detailed peptide sequences or associated proteins could not be identified. It remains unclear how these peptides were retained by the hydrazide beads. For samples with high peptide content, additional purification steps prior to analysis may be necessary.

## Conclusions

In this study, we compared three *N*-glycan release methods and two fluorescent labeling and purification strategies. Among them, the combination of Rapid PNGase F and BlotGlyco was identified as the most accurate, simple, and suitable for high-throughput sample processing. Using this optimized method, we analyzed glycan structures from three sample types while minimizing *O*-acetylation artifacts. The improved approach enabled the detection and structural characterization of complex, low-abundance, multi-branched glycans. The making use of pyridylamination allowed for high-resolution isomer separation *via* reversed-phase LC/MS, which facilitated the identification of precise glycan structures. The improved method had broad applicability across different sample types. MS analysis successfully estimated glycan structures present at 0.1–0.01% of the total glycan pool, and MS/MS enabled reliable structure estimation at approximately 0.1% abundance. For sample preparation, water-insoluble materials such as CHO-K1 membranes required solubilization with a surfactant, indicating that the method is particularly well-suited for water-soluble samples. Overall, this study establishes a robust and high-precision analytical workflow that supports both biomarker discovery and the quality control of recombinant glycoproteins. In addition, by simplifying complex glycan analysis, this method has the potential to encourage researchers from other fields to enter the field of glycobiology.

## Supporting information

S1 File Supporting Materials and S1–5 Figs. (PDF)

S2 File S1–6 Tables. (PDF)
