# Supplementary material for: Optimizing the preparation of labeled N-glycans for rapid, simplified, and high-precision analysis
Source: PLoS One. 2025 Dec 16;20(12):e0336565. doi: 10.1371/journal.pone.0336565 (PMC12707664; doi:10.1371/journal.pone.0336565)
Supplement: S1 File — (PDF) [file pone.0336565.s001.pdf]

Supporting Information (S1 File)

Rapid, simple, and precise optimization of preparation processes for *N*-glycan analysis

Riko Makino[1] and Shunji Natsuka[1]*

[1] Department of Food and Life Sciences, Graduate School of Science and Technology, Niigata University 8050 Ikarashi-nino-cho, Nishi-ku, Niigata 950-2181, Japan

*To whom correspondence should be addressed: Tel/Fax: +81-25-262-6174; e-mail: natsuka@bio.sc.niigata-u.ac.jp

Supporting Materials and Methods (p.2,3)

S1 Fig. The notation and abbreviations of PA-*N*-glycans. (p.4)

S2 Fig. Main and side reactions of reducing terminal GlcNAc. (p.5)

S3 Fig. Fractionation by number of negative charges by DEAE-HPLC. (p.6)

S4 Fig. 66N-BI derived byproducts present at the 66N-BI elution position. (p.7)

S5 Fig. Peptide-like ions in the CHO-K1 membrane fraction. (p.8)

Supporting references (p.9)

Supporting Materials and Methods

Materials

Reagents were obtained from Kanto Chemical Co., Inc. (Tokyo, Japan) or Fujifilm Wako Pure Chemical Co. (Osaka, Japan). 2-aminopyridine was purified by recrystallization in n-hexane. Rapid PNGase F was obtained from New England BioLabs Inc. (cat. no. P0710S, Hitchin, UK). BlotGlyco was obtained from Sumitomo Bakelite Co., Ltd. (cat. no. BS-45414, Tokyo, Japan). Bio-Rad Protein Assay Kit was obtained from Bio-Rad Laboratories, Inc. (Hercules, CA) and an BCA Protein Assay Kit was obtained from Takara Bio Inc. (Shiga, Japan).

Isolation of CHO-K1 Membrane Fraction

CHO-K1 ($\sim 1\times10^8$ cells) suspended in 10 mL lysis buffer (10 mM HEPES-KOH pH 7.9, 1.5 mM $MgCl_2$, 10 mM KCl, 0.1 mM PMSF), were kept on ice for 15 min. Lysate was homogenized using a Potter homogenizer 20 stroker, 300 rpm, and centrifuged 700 x g for 5 min. Supernatant was collected, and the precipitate was again suspended, homogenized and centrifuged under the same conditions. Each supernatant was combined, and centrifuged 10,5000 ×g at 4°C for 60 min. The precipitate containing lysosome, ER and Golgi was lyophilized, and then stored at -20°C. The protein content of CHO-K1 microsomal fraction was 20% (w/w) when analyzed by BCA assay.

N-glycan Release by PNGase F and Rapid PNGase F

PNGase F: Sample was suspended in 16 µL water in 0.2 mL PCR tubes, to which was added 1 µL of Glycoprotein Denaturing Buffer, and then mixed gently with a pipette. The mixture was denatured at 99°C for 10 min, and cool downed. After that, 2 µL of GlycoBuffer 2 (10x), 2 µL 10% NP-40 and 6 µL water was added to sample and mix gently. And then 1 µL PNGase F was added and incubated at 37°C for 60 min.

Rapid PNGase F: Each sample was suspended in 16 µL water (0.1 % NP-40 in the case of CHO-K1 microsomal fraction) in 0.2 mL PCR tubes, to which was added 4 µL of Rapid PNGase F Buffer (5x), and then mixed gently with a pipette. The mixture was denatured at 80°C for 2 min, and cool downed. After that, 1 µL of Rapid PNGase F was added to sample and then incubated at 50°C for 10 min.

Anion-Exchange HPLC

Prior to LC-MS analysis, the samples shown in Fig. 3 were fractionated based on the number

of negative charges using anion-exchange HPLC. This was performed with a TSK-gel diethylaminoethyl (DEAE)-5PW column (0.75 × 7.5 cm; Tosoh Corporation) and a Waters Alliance HPLC system equipped with a Waters 2475 fluorescence detector (Milford, MA), as described in the literature [1].

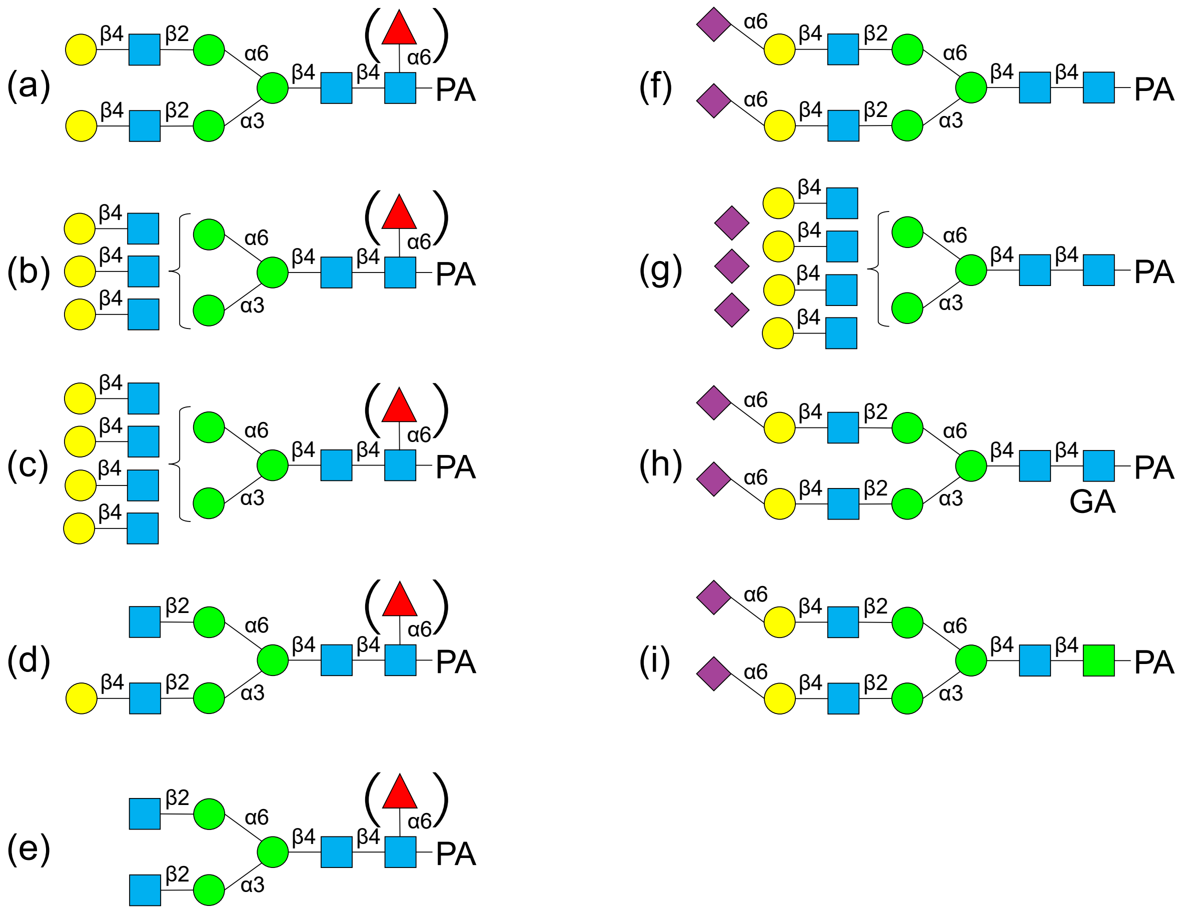

S1 Fig. Notation and abbreviations of PA-*N*-glycans.

(a–c) Representation of complex-type *N*-glycans classified by the number of LacNAc branches (antennae). (a) Bi-antennary glycan (BI); (b) Tri-antennary glycan (TR); (c) Tetra-antennary glycan (TE). For structures with unknown antennae binding positions, these positions are indicated in parentheses, as shown in (b) and (c). (d, e) Agalactosylated BI glycans. (f) Double-α2,6-sialylated bi-antennary glycan, the most abundant structure in serum, referred to as 66N-BI in this study. (g) Tetra-antennary glycan with three additional NeuAc residues of unknown positions, abbreviated as trN-TE in this study. Prefixes such as dN- (di-sialylated) and teN- (tetra-sialylated) are used similarly to indicate the number of NeuAc residues. (h) Glycosylamine (GA) form of 66N-BI. (i) Epimeric structure of 66N-BI.

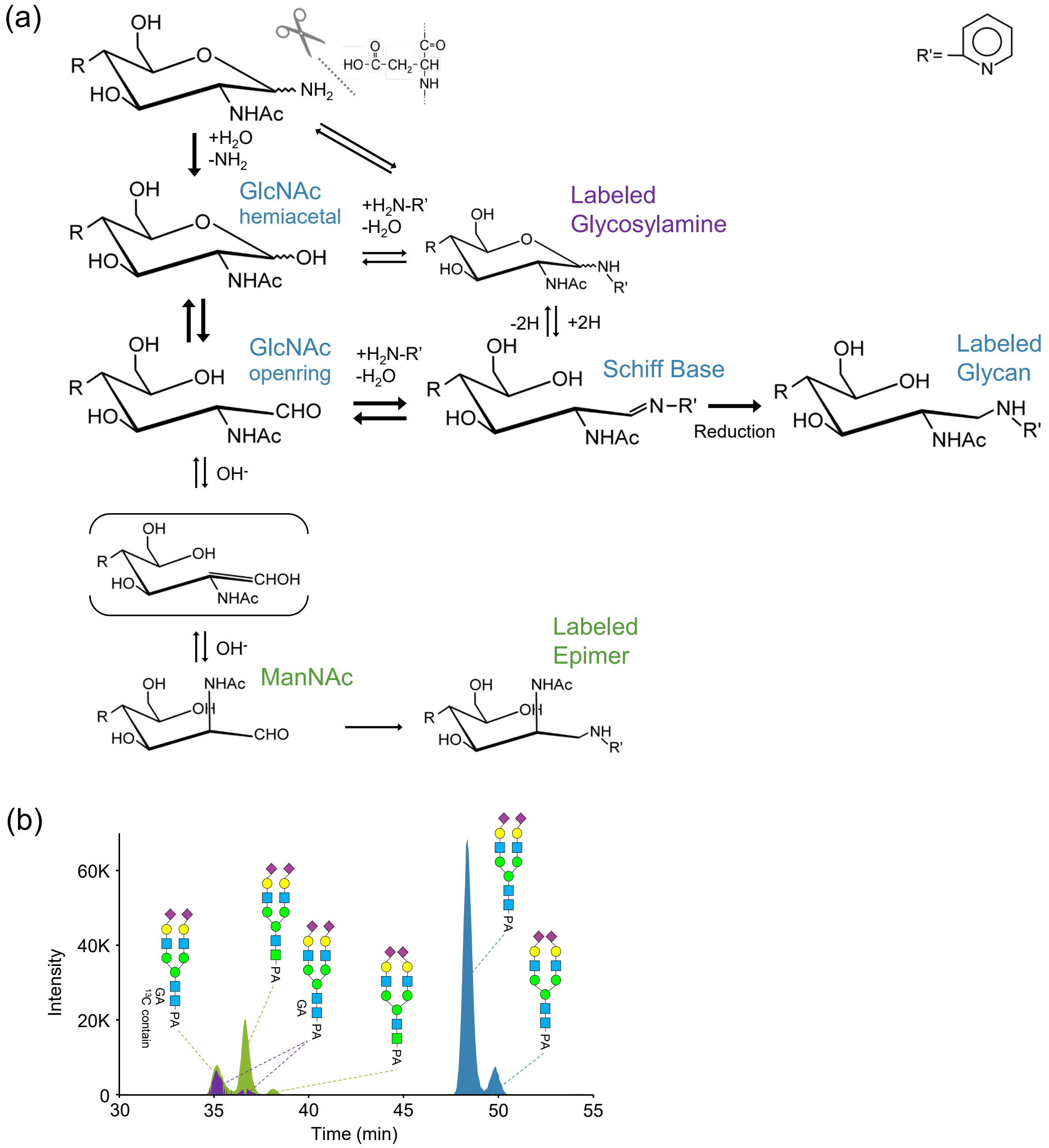

S2 Fig. Main and side reactions at the reducing terminal GlcNAc.

(a) Schematic illustration of the reaction pathways. The main reaction pathway is indicated in blue, while side reaction pathways are shown in purple (formation of glycosylamine, GA) and green (epimerization). The reactions involve *N*-glycans released from Asn residues.

(b) Mass chromatograms showing the RP-LC elution profiles of the main and side products derived from double-sialylated BI-type glycans. The color coding corresponds to the pathways depicted in (a).

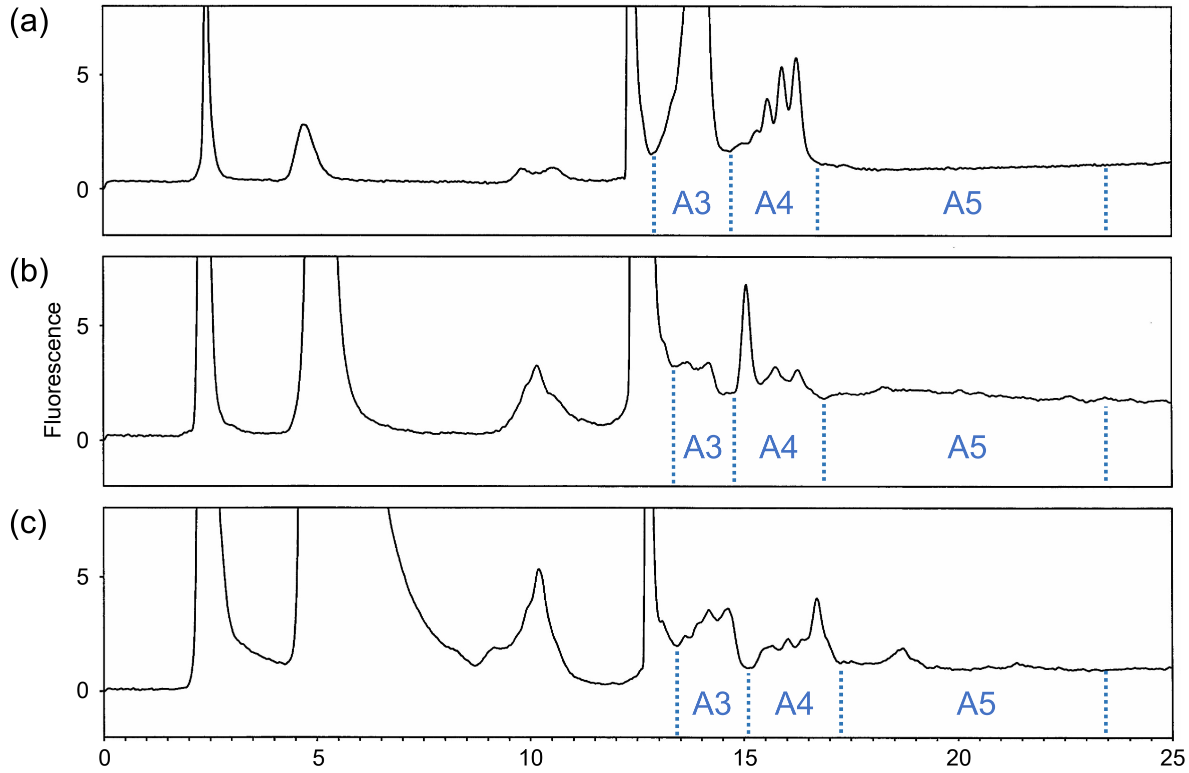

S3 Fig. Fractionation of glycans by number of negative charges using DEAE-HPLC. DEAE-HPLC fractionation corresponding to the data presented in Fig. 2. (a) Glycans derived from human α1-acid glycoprotein (α1-AGP), used as an external standard. Empirically defined anionic fractions A3, A4, and A5 are indicated. It is well established that fraction A3 predominantly contains glycans with three negative charges, while A4 primarily contains those with four. (b) Fractionation of glycans prepared using a combination of hydrazinolysis and conventional methods. The major fluorescence peak observed in fraction A4 is presumed to originate from *O*-glycans rather than N-glycans. (c) Fractionation of glycans prepared using Rapid PNGase F treatment combined with the BlotGlyco method.

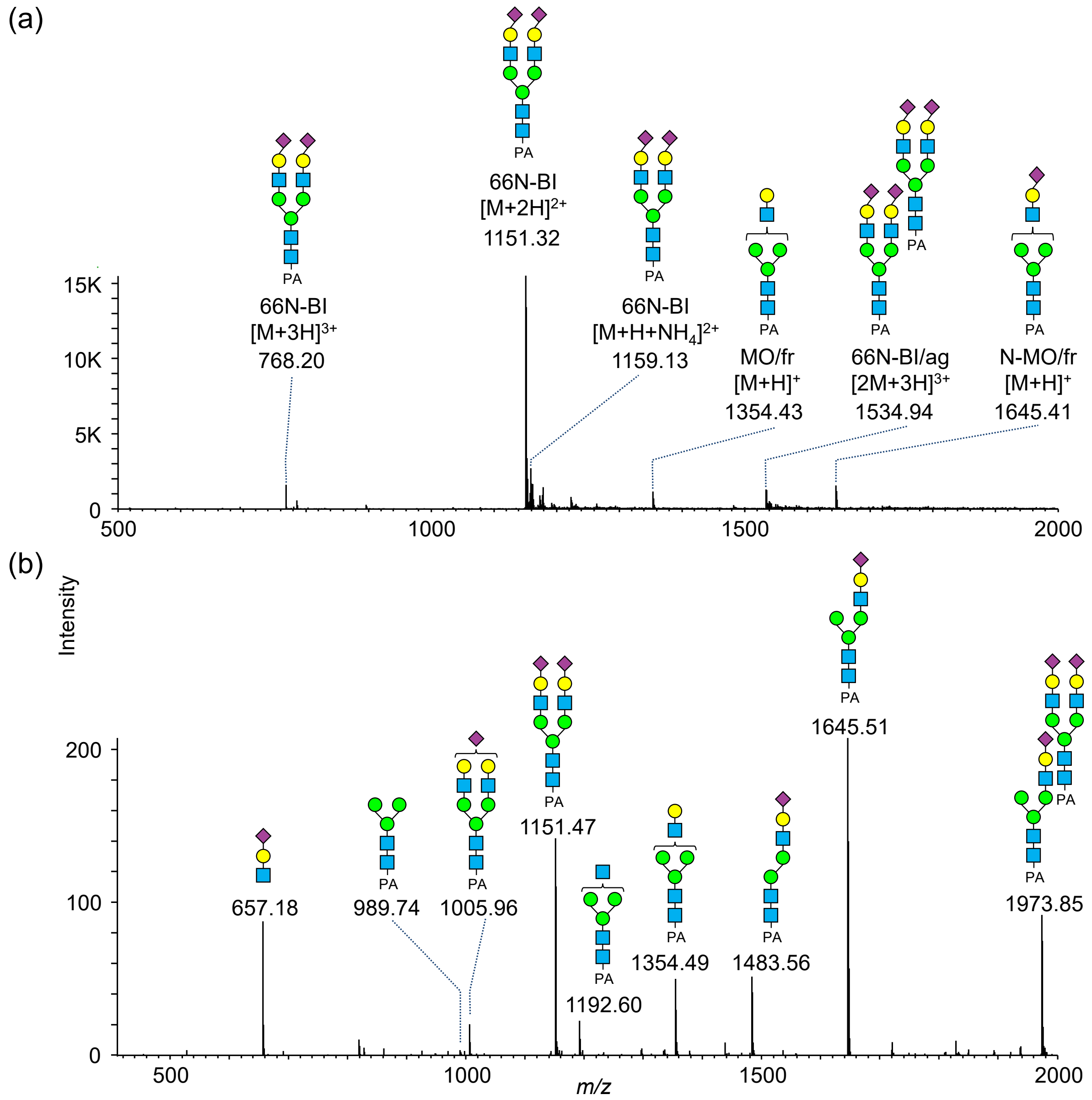

S4 Fig. Byproducts of 66N-BI detected at its elution position.

(a) MS[1] profile of human urine sample M1 at RT = 48.68 min, corresponding to the elution position of 66N-BI. Signals labeled as 66N-BI represent various ion forms of the molecule; "/fr" denotes in-source decay (ISD) or post-source decay (PSD) fragments, and "/ag" refers to multivalent ion aggregates composed of two 66N-BI molecules. (b) MS/MS spectrum of the 66N-BI aggregate (66N-BI/ag).

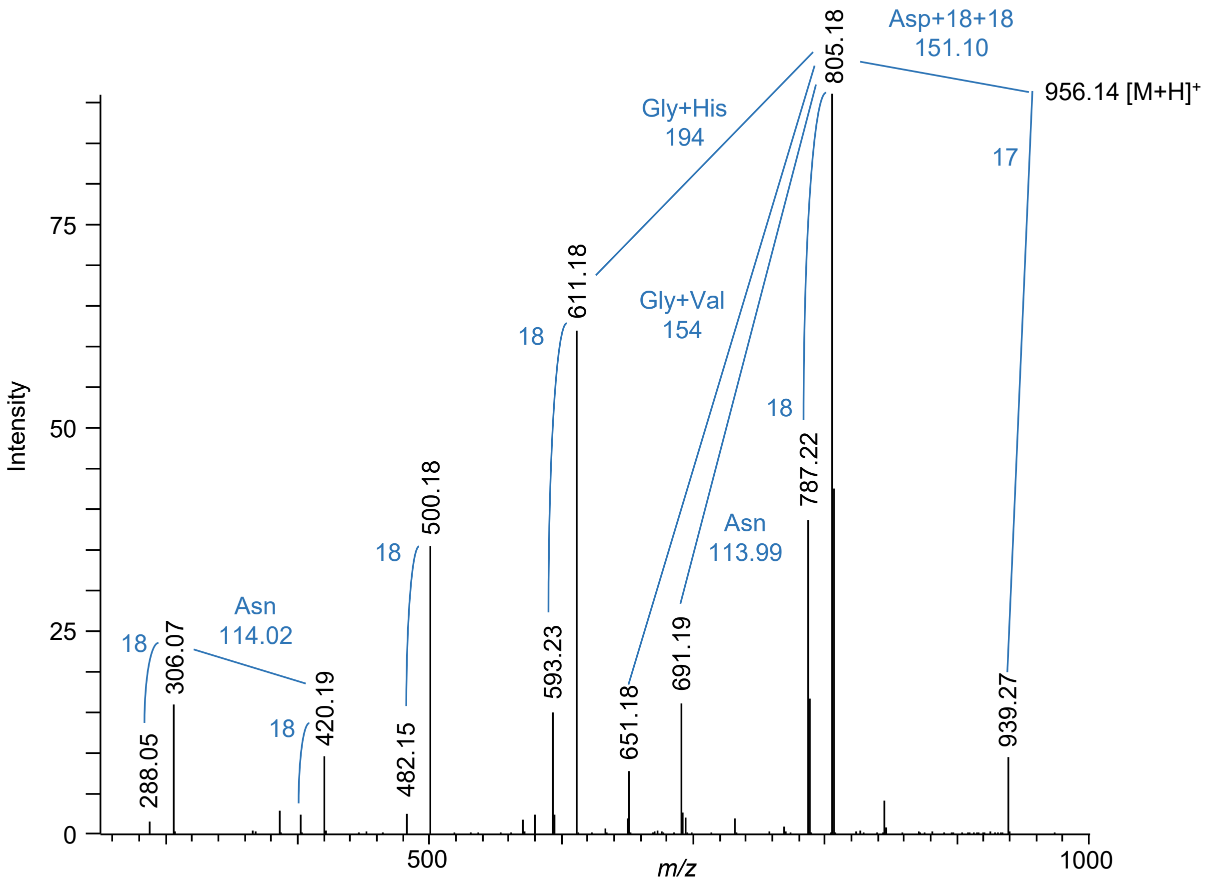

S5 Fig. Peptide-like ions detected in the CHO-K1 membrane fraction.

MS/MS profile showing neutral losses corresponding to peptide masses. Extensive losses of water molecules and amino groups, characteristic of peptide fragmentation, are also observed.

Supporting Reference

[1] Natsuka S, Masuda M, Sumiyoshi W, Nakakita S. Improved method for drawing of a glycan map, and the first page of glycan atlas, which is a compilation of glycan maps for a whole organism. *PLoS ONE*. 2014, 9:e102219. DOI: 10.1371/journal.pone.0102219
