# Supplementary material for: Optimizing the preparation of labeled N-glycans for rapid, simplified, and high-precision analysis
Source: PLoS One. 2025 Dec 16;20(12):e0336565. doi: 10.1371/journal.pone.0336565 (PMC12707664; doi:10.1371/journal.pone.0336565)
Supplement: S2 File — (PDF) [file pone.0336565.s002.pdf]

Supporting Information (S2 File)

Rapid, simple, and precise optimization of preparation processes for *N*-glycan analysis

Riko Makino and Shunji Natsuka*
Department of Food and Life Sciences, Graduate School of Science and Technology, Niigata University 8050 Ikarashi-nino-cho, Nishi-ku, Niigata 950-2181, Japan

*Correspondence author: Shunji Natsuka
Tel/Fax: +81-25-262-6174; e-mail: natsuka@bio.sc.niigata-u.ac.jp

Table S1. *N* -glycans from human serum pool analyzed by RP-LC-MS/MS. (p.2–4)
Table S2-1. DEAE A3 fraction of *N* -glycans prepared from human urine using the improved method. (p.5–7)
Table S2-2. DEAE A4 fraction of *N* -glycans prepared from human urine using the improved method. (p.8, 9)
Table S2-3. DEAE A5 fraction of *N* -glycans prepared from human urine using the improved method. (p.10)
Table S3. *N*-glycans from human serum pool analyzed using the improved method. (p.11–17)
Table S4. *N*-glycans from human urine M1 analyzed using the improved method. (p.18–26)
Table S5. *N*-glycans from human urine M2 analyzed using the improved method. (p.27–35)
Table S6. *N*-glycans from CHO-K1 analyzed using the improved method. (p.36–40)

Table S1. *N*-glycans from human serum pool analyzed by RP-LC-MS/MS.
For each sample, the relative yield of each glycan is shown, with the yield of 66N-BI normalized to 100.

Fig 1 No.	RT (min)	*m/z* experi-mental	*m/z* theoretical	*m/z* ion form	MS	Composition (+PA)	Glycan structure		Hydrazinolysis & Conventional				PNGase F & Conventional			Rapid PNGase F & Conventional					RapidPNGase F & BlotGlyco			
									1	2	3	4	1	2	3	1	2	3	4	5	1	2	3	4
5	23.62	889.75	900.33	2H+	1798.65	H8Hn2	**M8A**	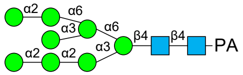	1.028	1.032	0.824	0.829	0.565	0.682	0.639	0.516	0.504	0.568	0.573	0.513	0.680	0.607	0.649	0.639
4	24.50	818.94	819.31	2H+	1636.60	H7Hn2	**M7A**	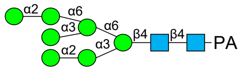	0.307	0.324	0.370	0.462	0.388	0.356	0.419	0.455	0.391	0.352	0.295	0.378	0.585	0.595	0.624	0.620
7	25.60	980.84	981.36	2H+	1960.70	H9Hn2	**M9A**	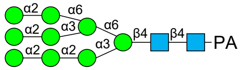	0.676	0.340	0.531	0.610	0.581	0.704	0.587	0.464	0.636	0.485	0.502	0.475	0.644	0.679	0.602	0.608
6	27.20	899.86	900.33	2H+	1798.65	H8Hn2	**M8B**	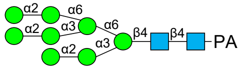	0.692	0.784	0.824	0.798	0.312	0.538	0.473	0.352	0.313	0.518	0.591	0.643	0.429	0.365	0.317	0.367
3	29.05	737.81	738.28	2H+	1474.54	H6Hn2	**M6B**	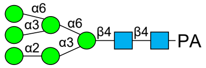	1.289	1.276	1.652	1.573	2.322	2.347	2.344	2.409	2.513	2.437	2.311	2.358	2.524	2.494	2.445	2.516
32	32.48	1005.84	1005.88	2H+	2009.75	N1H5Hn4	06N-**BI**/epi	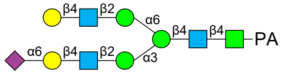	0.645	0.824	0.707	0.777	2.896	2.234	1.698	1.852	1.405	1.554	1.645	1.675	1.045	0.901	0.809	0.819
2	33.20	1313.44	1313.50	H+	1312.49	H5Hn2	**M5A**	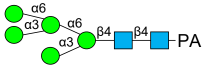	3.337	3.372	3.388	3.595	2.341	2.924	2.692	2.774	2.745	3.080	2.879	2.829	2.734	2.848	2.751	2.749
30	34.80	1150.60	1151.43	2H+	2300.84	N2H5Hn4	66N-**BI**/ga	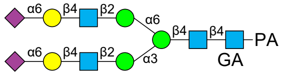	1.190	1.081	0.869	1.025	1.429	0.583	0.521	1.148	1.387	1.637	1.593	1.599	2.185	1.506	1.919	1.881
31	36.42	1151.44	1151.43	2H+	2300.84	N2H5Hn4	66N-**BI**/epi	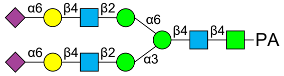	6.259	6.114	5.892	6.213	16.825	14.679	13.195	11.348	9.091	12.615	12.541	12.175	6.017	6.273	5.846	5.716
	37.04	1150.47	1151.43	2H+	2300.84	N2H5Hn4	36N-**BI**/ga	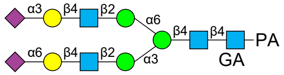	0.060	0.094	0.123	0.162	0.060	0.019	0.064	0.024	0.024	0.166	0.230	0.218	0.032	0.030	0.019	0.015
	37.04	985.30	985.37	2H+	1968.72	N1H6Hn3	6N-Ln-**M5A**	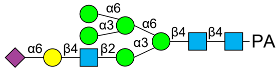	0.275	0.274	0.218	0.209	0.054	0.032	0.082	0.042	0.027	0.045	0.065	0.051	0.072	0.087	0.046	0.031
	37.04	823.34	823.31	2H+	1644.61	N1H4Hn3	6N-**MO1**	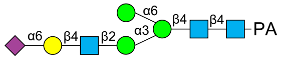	0.368	0.381	0.495	0.475	0.026	0.016	0.032	0.026	0.014	0.018	0.030	0.024	0.036	0.047	0.022	0.015
14	37.90	904.16	904.34	2H+	1806.67	N1H5Hn3	6N-Ln-**M4C**	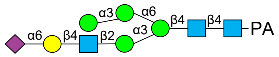	0.827	0.930	0.894	0.724	2.974	2.081	2.201	2.174	2.039	2.226	2.069	2.001	2.245	2.013	2.030	1.855
15	42.63	924.87	924.85	2H+	1847.69	N1H4Hn4	06N-**BI**-Ga2	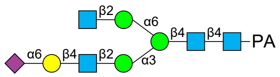	1.871	1.615	2.385	2.402	1.815	1.330	1.394	0.986	1.449	0.921	0.806	0.674	0.975	1.291	1.034	1.168
22	43.74	1224.50	1224.46	2H+	2446.90	N2H5Hn4dH1	N-3N-F(3)-**BI**	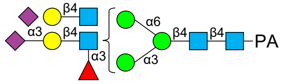	1.090	1.185	0.926	1.039	1.112	0.997	1.122	1.135	0.648	0.727	0.722	0.768	0.898	0.905	1.072	0.919

Table S1 continued.

Fig 1 No.	RT (min)	*m/z* experi-mental	*m/z* theoretical	*m/z* ion form	MS	Composition (+PA)	Glycan structure		Hydrazinolysis & Conventional 1	Hydrazinolysis & Conventional 2	Hydrazinolysis & Conventional 3	Hydrazinolysis & Conventional 4	PNGase F & Conventional 1	PNGase F & Conventional 2	PNGase F & Conventional 3	Rapid PNGase F & Conventional 1	Rapid PNGase F & Conventional 2	Rapid PNGase F & Conventional 3	Rapid PNGase F & Conventional 4	Rapid PNGase F & Conventional 5	RapidPNGase F & BlotGlyco 1	RapidPNGase F & BlotGlyco 2	RapidPNGase F & BlotGlyco 3	RapidPNGase F & BlotGlyco 4
	43.74	986.66	986.70	3H+	2957.07	N3H6Hn5	trN-**TR**	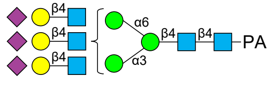	N.D.	N.D.	N.D.	N.D.	2.874	2.083	2.110	2.030	1.726	1.921	1.791	1.893	0.769	0.717	0.662	0.498
12	43.74	860.43	860.33	2H+	1718.65	H5Hn4	**BI**	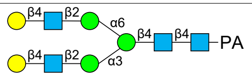	0.930	0.959	1.053	1.227	0.515	0.512	0.552	0.550	0.365	0.542	0.482	0.469	1.309	1.415	1.275	1.283
16	44.62	1006.01	1005.88	2H+	2009.75	N1H5Hn4	06N-**BI**	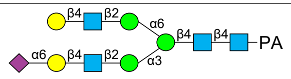	19.047	18.589	19.119	20.294	18.127	17.063	17.733	18.275	20.014	16.440	16.368	16.401	17.690	18.960	18.027	18.845
1	48.17	1151.78	1151.43	2H+	2300.84	N2H5Hn4	66N-**BI**	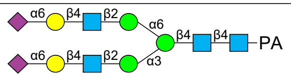	100	100	100	100	100	100	100	100	100	100	100	100	100	100	100	100
20	49.64	1151.51	1151.43	2H+	2300.84	N2H5Hn4	36N-**BI**	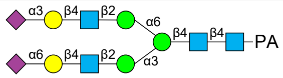	9.459	8.354	7.266	8.685	9.895	9.764	9.539	10.281	10.605	9.417	9.173	9.929	8.441	8.804	8.312	7.844
9	49.64	771.37	771.31	2H+	1540.60	H3Hn4dH1	**AG12**F(6)	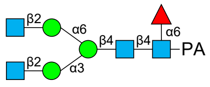	9.210	8.717	10.046	10.648	7.879	6.848	7.205	7.480	8.079	9.109	9.351	8.622	9.055	8.633	8.883	9.341
8	49.64	1338.48	1338.53	H+	1337.52	H3Hn3dH1	**AG2**F(6)	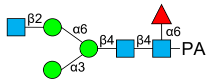	3.286	3.059	3.317	2.734	2.890	2.728	2.668	3.558	3.072	2.646	2.928	2.686	2.880	2.694	2.925	2.786
29	49.64	1205.59	1205.44	3H+	3613.30	N4H7Hn6	teN-**TE**	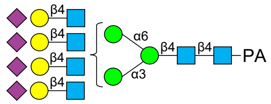	N.D.	N.D.	N.D.	N.D.	0.655	0.726	0.614	0.652	0.692	0.550	0.571	0.609	0.545	0.632	0.703	0.589
10	51.71	852.38	852.33	2H+	1702.66	H4Hn4dH1	**BI**F(6)-Ga2	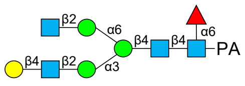	16.080	17.794	18.397	19.257	16.958	15.526	15.246	16.250	15.950	14.522	14.641	14.590	16.067	15.750	15.555	14.780
11	53.92	933.49	933.36	2H+	1864.71	H5Hn4dH1	**BI**F(6)	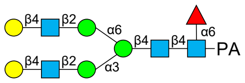	8.765	10.004	9.088	8.636	6.931	6.974	7.150	6.107	6.422	7.317	6.906	7.903	7.054	7.137	6.898	6.806
25	53.92	889.73	889.67	3H+	2665.97	N2H6Hn5	dN-**TR**	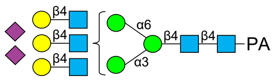	1.361	1.581	1.623	1.836	1.626	1.535	1.445	1.816	2.213	1.846	1.392	1.595	1.930	2.021	1.818	2.104
21	53.92	1151.16	1151.43	2H+	2300.84	N2H5Hn4	63N-**BI**	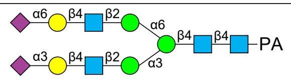	0.432	0.673	0.531	0.581	0.593	0.576	0.578	0.582	0.590	0.701	0.587	0.562	0.416	0.468	0.507	0.441
28	53.92	1035.51	1035.38	3H+	3103.13	N3H6Hn5dH1	dN-3N-F(3)-**TR**	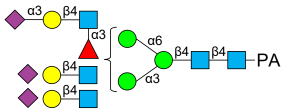	7.385	7.044	6.425	7.141	8.786	9.020	8.156	8.677	7.364	7.562	8.544	8.204	7.850	6.625	6.773	6.519
17	55.15	1078.94	1078.91	2H+	2155.80	N1H5Hn4dH1	06N-**BI**F(6)	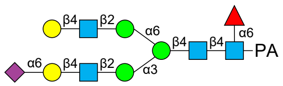	13.851	13.251	14.314	14.023	10.292	9.840	9.806	10.265	10.089	10.147	9.804	9.587	11.521	11.565	13.588	12.870

Table S1 continued.

Fig 1 No.	RT (min)	*m/z* experi-mental	*m/z* theoretical	*m/z* ion form	MS	Composition (+PA)	Glycan structure		Hydrazinolysis & Conventional 1	Hydrazinolysis & Conventional 2	Hydrazinolysis & Conventional 3	Hydrazinolysis & Conventional 4	PNGase F & Conventional 1	PNGase F & Conventional 2	PNGase F & Conventional 3	Rapid PNGase F & Conventional 1	Rapid PNGase F & Conventional 2	Rapid PNGase F & Conventional 3	Rapid PNGase F & Conventional 4	Rapid PNGase F & Conventional 5	RapidPNGase F & BlotGlyco 1	RapidPNGase F & BlotGlyco 2	RapidPNGase F & BlotGlyco 3	RapidPNGase F & BlotGlyco 4
23	56.96	1224.51	1224.46	2H+	2446.90	N2H5Hn4dH1	66N-**BI**F(6)	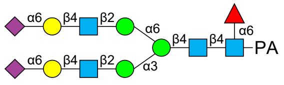	10.976	10.076	9.505	8.619	11.761	11.458	12.316	9.199	9.702	10.151	9.202	8.358	9.063	7.398	10.475	9.534
26	56.96	986.86	986.70	3H+	2957.07	N3H6Hn5	trN-**TR**	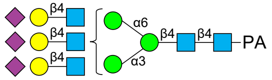	10.706	10.858	8.995	9.169	10.162	11.566	10.694	15.435	14.111	13.997	15.586	16.529	14.173	14.606	11.493	11.561
27	60.96	986.83	986.70	3H+	2957.07	N3H6Hn5	trN-**TR**	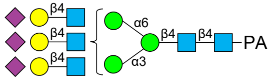	5.389	5.030	5.642	5.111	5.308	5.603	5.400	5.683	5.411	6.184	6.101	6.085	5.507	5.408	5.431	5.607
19	60.96	1078.88	1078.91	2H+	2155.80	N1H5Hn4dH1	03N-**BI**F(6)	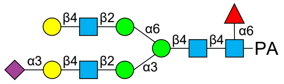	0.877	0.722	0.572	0.621	0.668	0.833	0.985	0.705	0.700	0.343	0.419	0.433	0.641	0.654	0.630	0.558
18	70.52	1180.45	1180.45	2H+	2358.88	N1H5Hn5dH1	06N-**BI**BsF(6)	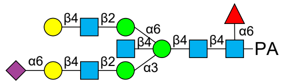	4.813	5.270	4.680	4.278	4.636	4.514	4.821	5.348	5.267	4.830	4.860	4.217	4.564	4.370	4.637	4.615
24	71.43	884.53	884.33	3H+	2649.98	N2H5Hn5dH1	66N-**BI**BsF(6)	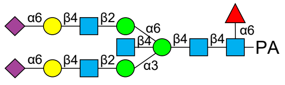	6.904	7.340	6.439	6.439	6.903	7.120	6.964	6.551	6.650	6.295	6.614	6.470	6.764	6.819	6.466	6.534
13	73.03	1035.29	1034.90	2H+	2067.79	H5Hn5dH1	**BI**BsF(6)	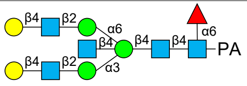	1.474	1.428	1.582	1.568	1.264	1.298	1.273	1.337	1.261	1.153	1.128	1.091	1.499	1.504	1.501	1.445
33	76.94	1172.13	1172.43	2H+	2342.85	N2H5Hn4Ac1	Ac-66N-**BI**	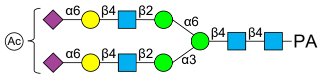	1.857	1.713	1.819	1.618	0.721	0.936	1.059	0.802	0.675	0.763	0.825	0.689	1.365	1.272	1.557	1.626
34	82.37	1172.13	1172.43	2H+	2342.85	N2H5Hn4Ac1	Ac-66N-**BI**	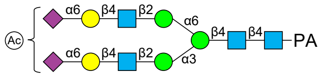	0.983	0.950	1.058	1.053	0.387	0.439	0.520	0.441	0.354	0.388	0.396	0.345	0.851	0.774	0.934	1.056
35	83.42	1172.13	1172.43	2H+	2342.85	N2H5Hn4Ac1	Ac-66N-**BI**	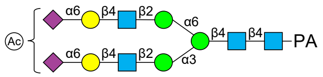	0.982	0.872	0.879	0.831	0.278	0.344	0.416	0.392	0.329	0.288	0.345	0.309	0.706	0.604	0.757	0.862
36	85.09	1172.13	1172.43	2H+	2342.85	N2H5Hn4Ac1	Ac-66N-**BI**	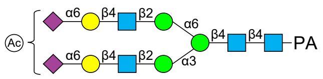	1.000	1.252	0.917	0.855	0.313	0.346	0.387	0.322	0.330	0.281	0.284	0.309	0.769	0.591	0.850	0.910

Table S2-1. DEAE A3 fraction of *N*-glycans prepared from human urine using the improved method. Peak numbers correspond to those shown in Fig. 2. GU and RP indicate the normalized elution times based on PA-isomaltooligosaccharides and PA-*N*-glycan core structures, respectively. “Experimental” refers to the measured values. “Composition” indicates the presence of the following components: S (sulfate group), P (phosphate group), N (NeuAc), H (Hex), Hn (HexNAc), and dH (deoxyHex). “% for top” indicates the yield relative to the most abundant glycan in the fraction, while “% for whole” indicates the yield as a proportion of the entire fraction. In the MS^2 column, a check mark (✓) indicates glycan structures for which MS/MS data were obtained, while a dash (–) indicates that MS/MS data were acquired but insufficient for structural assignment. The “Note” column highlights characteristic substructures. “Hydrazine RT” indicates the retention times of peaks also observed in samples prepared by hydrazinolysis and conventional methods.

Peak No.	RT (min)	GU	RP	*m/z*			MS	Composition (+PA)	Glycan structure		Quantity ratio		MS^2	Note	Hydrazine RT (min)
		experi-mental	experi-mental	experi-mental	theoretical	ion form					% for top	% for whole			
1	47.26	10.93	54.57	1035.01	1035.38	3H+	3103.13	N3H6Hn5dH1	trN-**TRF**(6)	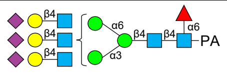	9.49	1.14			
	47.26	10.93	54.57	1066.18	1066.37	2H+	2130.73	S1N1H4Hn5	S-N-Ldn-**MO**	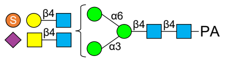	6.02	0.72		Ldn	47.16
2	48.19	11.25	55.51	1108.33	1108.41	3H+	3322.20	N3H7Hn6	trN-**TE**	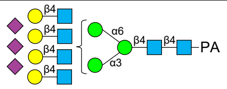	7.58	0.91			48.07
3	49.73	11.79	57.06	1108.68	1108.41	3H+	3322.20	N3H7Hn6	trN-**TE**	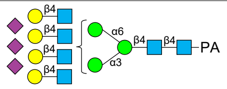	4.71	0.57			49.62
4	50.62	12.11	57.96	1156.74	1157.09	3H+	3468.26	N3H7Hn6dH1	trN-F-**TE**/ga	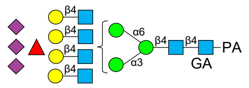					
	50.62	12.11	57.96	1086.84	1087.08	3H+	3258.22	N3H3Hn6dH4	trN-trF-dLdn-**M3**F(6)	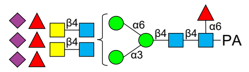	8.92	1.07		Ldn	50.58
5	52.21	12.69	59.56	1551.13	1552.57	2H+	3103.13	N3H6Hn5dH1	trN-F-**TR**	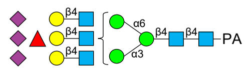	9.92	1.19			51.93
	52.21	12.69	59.56	1156.62	1157.09	3H+	3468.26	N3H7Hn6dH1	trN-**TE**F(6)/ga	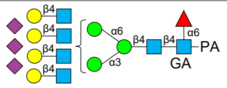					51.93
	52.21	12.69	59.56	1108.24	1108.41	3H+	3322.20	N3H7Hn6	trN-**TE**	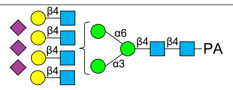	1.72	0.21			51.93
6	53.20	13.07	60.56	1551.11	1552.57	2H+	3103.13	N3H6Hn5dH1	trN-F-**TR**	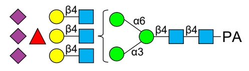	21.33	2.56			52.86
7	55.00	13.77	62.37	1551.91	1552.57	2H+	3103.13	N3H6Hn5dH1	trN-F-**TR**/ga	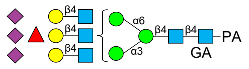					54.72
	55.00	13.77	62.37	1108.31	1108.41	3H+	3322.20	N3H7Hn6	trN-**TE**	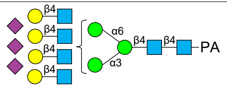	61.92	7.43			54.72
	56.48	14.38	63.86	1552.17	1552.57	2H+	3103.13	N3H6Hn5dH1	trN-F(3)-**TR**/ga	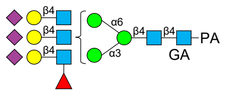			✓	Lex	56.28
8	57.81	14.94	65.20	1479.44	1479.54	2H+	2957.07	N3H6Hn5	trN-**TR**	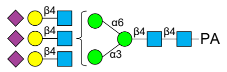	94.48	11.34	✓		57.55
9	58.81	15.38	66.21	1139.01	1139.40	2H+	2276.79	S1N1H4Hn5dH 1	S-N-F-Ldn-**MO**	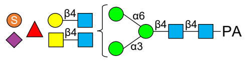	59.51	7.14		Ldn	58.64
	58.81	15.38	66.21	1479.68	1479.54	2H+	2957.07	N3H6Hn5	trN-**TR**	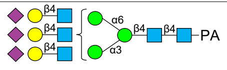	31.48	3.78			58.64
	58.81	15.38	66.21	1241.15	1240.79	3H+	3719.35	N3H8Hn5dH3	trN-dGa-trF-**TR**	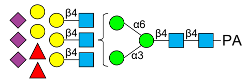	4.77	0.57			
10	60.03	15.93	67.44	1156.94	1157.09	3H+	3468.26	N3H7Hn6dH1	trN-**TE**F(6)	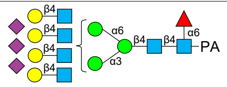	11.18	1.34			59.57

Table S2-1 continued.

Peak No.	RT (min)	GU	RP	*m/z*			MS	Composition (+PA)	Glycan structure		Quantity ratio		MS^2	Note	Hydrazine RT (min)
		experi-mental	experi-mental	experi-mental	theoretical	ion form					% for top	% for whole			
11	61.04	16.41	68.42	1156.76	1157.09	3H+	3468.26	N3H7Hn6dH1	trN-**TEF**(6)	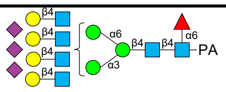	4.54	0.55			60.76
12	61.88	16.81	69.23	1479.17	1479.54	2H+	2957.07	N3H6Hn5	trN-**TR**	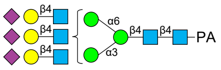	46.58	5.59	-		61.63
13	63.58	17.68	70.86	1156.84	1157.09	3H+	3468.26	N3H7Hn6dH1	trN-**TEF**(6)	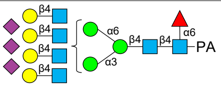	39.46	4.74			63.33
	63.58	17.68	70.86	1278.53	1278.80	3H+	3833.39	N3H8Hn7dH1	trN-**PEF**(6)	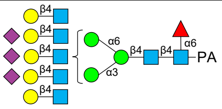	3.79	0.45			
14	65.38	18.65	72.58	1157.05	1157.09	3H+	3468.26	N3H7Hn6dH1	trN-**TEF**(6)	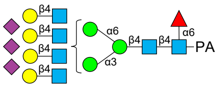	69.88	8.39			65.20
15	66.39	19.24	73.55	1156.89	1157.09	3H+	3468.26	N3H7Hn6dH1	trN-**TEF**(6)	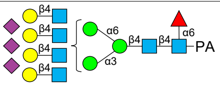	33.48	4.02			66.24
16	67.91	20.17	75.01	1552.05	1552.57	2H+	3103.13	N3H6Hn5dH1	trN-**TRF**(6)	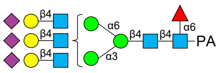	65.60	7.88	-		67.73
17	69.71	21.36	76.74	1170.71	1170.77	3H+	3509.29	N3H6Hn7dH1	trN-**TE**BsF(6)-Ga	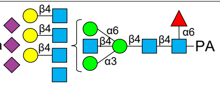	0.31	0.04			
	69.71	21.36	76.74	1176.13	1175.92	2H+	2349.83	S1N1H3Hn2dH4	S-N-trF-Ldn-**MO**F(6)	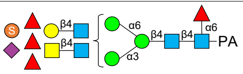	0.75	0.09		Ldn	69.61
	69.71	21.36	76.74	1278.95	1278.80	3H+	3833.39	N3H8Hn7dH1	trN-**PEF**(6)	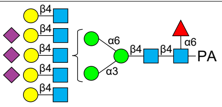	0.90	0.11			
18	70.27	21.75	77.28	1552.05	1552.57	2H+	3103.13	N3H6Hn5dH1	trN-**TRF**(6)	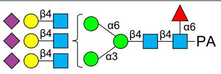	100	12.00	✓		70.11
19	71.46	22.62	78.42	1552.05	1552.57	2H+	3103.13	N3H6Hn5dH1	trN-**TRF**(6)	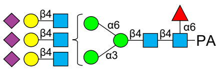	10.23	1.23			71.35
	71.46	22.62	78.42	1176.21	1176.10	3H+	3525.28	N3H7Hn5	trN-**TE**Bs	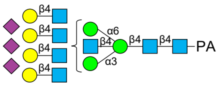	0.65	0.08			71.35
20	73.62	24.37	80.49	1552.05	1552.57	2H+	3103.13	N3H6Hn5dH1	trN-**TRF**(6)	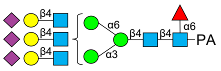	3.98	0.48			73.66
21	75.64	26.20	82.43	1552.34	1552.57	2H+	3103.13	N3H6Hn5dH1	trN-**TRF**(6)	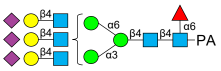	8.66	1.04			75.62
22	77.73	28.35	84.43	1552.05	1552.57	2H+	3103.13	N3H6Hn5dH1	trN-**TRF**(6)	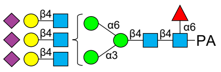	53.53	6.43			
23	83.61	36.16	90.07	1230.63	1230.80	3H+	3689.39	N3H3Hn9	trN-teLdn-**M3**Bs	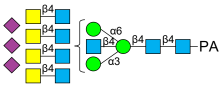	1.52	0.18		Ldn	
	83.61	36.16	90.07	1278.65	1278.80	3H+	3833.39	N3H8Hn7dH1	trN-**PEF**(6)	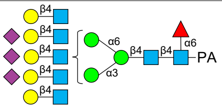	2.05	0.25			
24	87.09	42.38	93.41	1230.76	1230.80	3H+	3689.39	N3H3Hn9	trN-teLdn-**M3**Bs	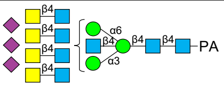	5.41	0.65		Ldn	
25	90.37	49.67	96.56	1279.50	1278.80	3H+	3833.39	N3H8Hn7dH1	trN-**PEF**(6)	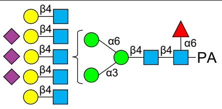	7.60	0.91			
26	92.05	54.04	98.17	1279.20	1278.80	3H+	3833.39	N3H8Hn7dH1	trN-**PEF**(6)	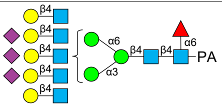	13.39	1.61			

Table S2-1 continued.

Peak No.	RT (min)	GU	RP	*m/z*			MS	Composition (+PA)	Glycan structure	Quantity ratio		MS^2	Note	Hydrazine
		experi-mental	experi-mental	experi-mental	theoretical	ion form				% for top	% for whole			RT (min)
27	93.46	58.08	99.52	1279.48	1278.80	3H+	3833.39	N3H8Hn7dH1	trN-**PE**F(6) 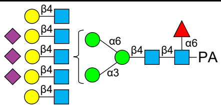	9.72	1.17			
28	94.04	59.85	100.08	1279.42	1278.80	3H+	3833.39	N3H8Hn7dH1	trN-**PE**F(6) 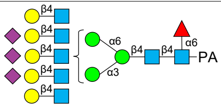	10.73	1.29			94.10
29	97.16	70.54	103.07	1279.16	1278.80	3H+	3833.39	N3H8Hn7dH1	trN-**PE**F(6) 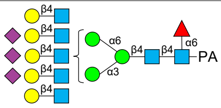	12.17	0.87			

Table S2-2. DEAE A4 fraction of *N*-glycans prepared from human urine using the improved method.

Peak No.	RT (min)	GU	RP	*m/z*			MS	Composition (+PA)	Glycan structure		Quantity ratio		MS^2	Note	Hydrazine
		experi-mental	experi-mental	experi-mental	theoretical	ion form					% for top	% for whole			RT (min)
30	28.23	5.96	35.05	1106.12	1106.38	2H+	2210.74	P1N1H7Hn3	6P-N-Ln-**M6**	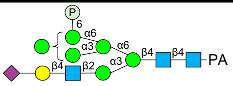	3.30	0.48			
31	29.50	6.20	36.27	1106.00	1106.38	2H+	2210.74	P1N1H7Hn3	6P-N-Ln-**M6**	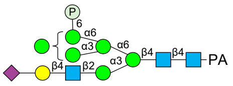	0.32	0.08			
	29.50	6.20	36.27	1178.10	1179.41	2H+	2356.80	P1N1H7Hn3dH1	6P-N-Ln-**M6**F(6)	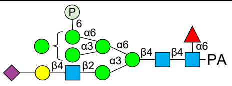	0.35	0.09			
32	37.64	8.05	44.53	1179.04	1179.41	2H+	2356.80	P1N1H7Hn3dH1	6P-N-F-Ln-**M6**	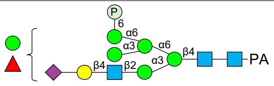	4.11	1.02	✓		
	40.41	8.81	47.45	1204.57	1205.44	3H+	3613.30	N4H7Hn6	teN-**TE**/ga	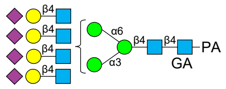					
33	42.11	9.30	49.25	1205.18	1205.44	3H+	3613.30	N4H7Hn6	teN-**TE**	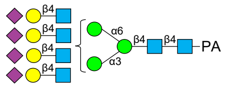	1.39	0.34			
	42.11	9.30	49.25	1062.57	1062.86	H+NH4+	2106.67	S2H3Hn6dH1	dS-dLdn12-**M3**F(6)						
34	42.71	9.48	49.88	1053.77	1054.34	2H+	2106.67	S2H3Hn6dH1	dS-dLdn12-**M3**F(6)	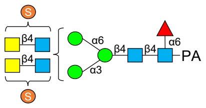	3.07	0.76		Ldn	
35	46.13	10.56	53.43	1191.71	1191.41	2H+	2380.80	S1N2H5Hn4	S-3N-6N-**BI**	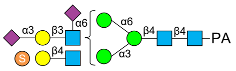	1.76	0.44		dN-Ln	
	47.49	11.01	54.80	1253.42	1254.13	3H+	3777.39	N4H7Hn6dH1	teN-**TE**F(6)/ga	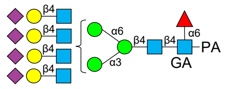	3.01	0.75			
	47.49	11.01	54.80	1368.70	1370.00	2H+	2737.99	N3H5Hn4dH1	trN-**BI**F(6)/ga	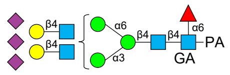	2.68	0.66			
36	49.41	11.67	56.74	981.16	981.32	2H+	1960.62	S2H3Hn6	dS-dLdn-**M3**	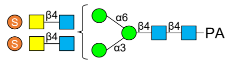	3.81	0.94		Ldn	
	49.41	11.67	56.74	1445.46							2.97	0.74			
	49.41	11.67	56.74	1253.54	1254.13	3H+	3777.39	N4H7Hn6dH1	teN-**TE**F(6)						
	49.41	11.67	56.74	1205.25	1205.44	3H+	3631.33	N4H7Hn6	teN-**TE**						
37	50.58	12.09	57.91	1205.32	1205.44	3H+	3631.33	N4H7Hn6	teN-**TE**	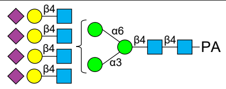	11.99	2.97			50.72
	50.58	12.09	57.91	1253.83	1254.13	3H+	3777.39	N4H7Hn6dH1	teN-**TE**F(6)	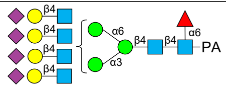	6.51	1.61			
	50.58	12.09	57.91	1446.41							1.61	0.40			
38	51.60	12.46	58.94	1253.72	1254.13	3H+	3777.39	N4H7Hn6dH1	teN-**TE**F(6)	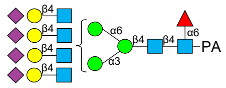	14.28	3.54			51.7
39	52.79	12.91	60.14	1253.72	1254.13	3H+	3777.39	N4H7Hn6dH1	teN-**TE**F(6)						
	52.79	12.91	60.14	1369.46	1370.00	2H+	2737.99	N3H5Hn4dH1	trN-**BI**F(6)	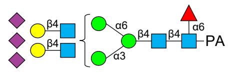	1.83	0.45			
40	54.54	13.59	61.91	1205.45	1205.44	3H+	3631.33	N4H7Hn6	teN-**TE**	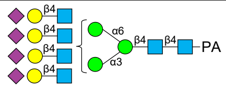	32.80	8.13			54.67
	54.54	13.59	61.91	1369.93	1370.00	2H+	2737.99	N3H5Hn4dH1	trN-**BI**F(6)	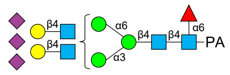	2.59	0.64			54.67
	54.54	13.59	61.91	1033.65							2.50	0.62			
41	55.75	14.08	63.12	1369.65	1370.50	3H+	4108.49	N4H7Hn7dH2	teN-F-**TE**BsF(6)	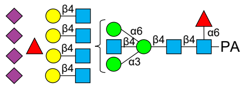	4.72	1.17	✓	dN-Ln	55.65
	55.75	14.08	63.12	1264.11	1264.44	2H+	2526.82	S1N2H5Hn4dH1	S-dN-**BI**F(6)	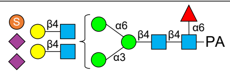	3.83	0.95			

Table S2-2 continued.

Peak No.	RT (min)	GU	RP	*m/z*			MS	Composition (+PA)	Glycan structure		Quantity ratio		MS²	Note	Hydrazine
		experi-mental	experi-mental	experi-mental	theoretical	ion form					% for top	% for whole			RT (min)
	55.75	14.08	63.12	1253.85	1254.13	3H+	3759.35	N4H7Hn6dH1	teN-**TEF**(6)		0.88	0.22			
42	56.54	14.40	63.92	1525.68							18.09	4.48			56.76
	56.54	14.40	63.92	1462.68	1463.04	2H+	2924.06	N4H4Hn5	teN-Ldn-**BI**	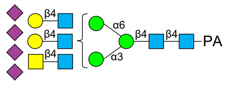	3.74	0.93		Ldn	
	56.54	14.40	63.92	1254.08	1254.13	3H+	3777.39	N4H7Hn6dH1	teN-**TEF**(6)	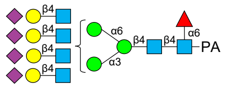	3.74	0.93			
	56.54	14.40	63.92	1525.07							1.51	0.37			
43	58.13	15.08	65.52	1263.97	1264.44	2H+	2526.82	S1N2H5Hn4dH1	S-dN-**BIF**(6)	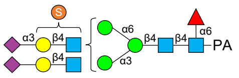	3.42	0.85		S-N-Ln	
44	58.76	15.36	66.16	1053.94	1054.34	2H+	2106.67	S2H3Hn6dH1	dS-dLdn-**M3F**(6)	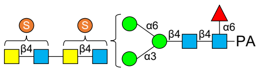	42.21	10.46	-	Ldn	58.95
45	60.37	16.09	67.78	1327.36	1327.83	3H+	3980.48	N4H3Hn11dH4	teN-teLdn-**M3**Bs	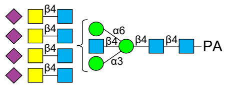	2.37	0.59		Ldn	
46	61.49	16.62	68.85	1253.91	1254.13	3H+	3777.39	N4H7Hn6dH1	trN-**TEF**(6)	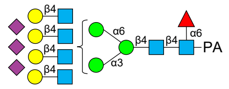	58.22	14.43			61.73
47	62.23	16.99	69.56	1322.21	1321.82	3H+	3962.43	N4H7Hn7dH1	teN-**TE**BsF(6)	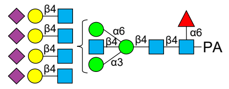	1.26	0.31			
	62.23	16.99	69.56	1254.13	1254.13	3H+	3777.39	N4H7Hn6dH1	trN-**TEF**(6)		0.54	0.13			
48	63.63	17.70	70.90	1321.92	1321.82	3H+	3962.43	N4H7Hn7dH1	teN-**TE**BsF(6)	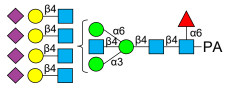	12.61	3.12			63.71
49	65.08	18.49	72.30	1253.86	1254.13	3H+	3777.39	N4H7Hn6dH1	trN-**TEF**(6)	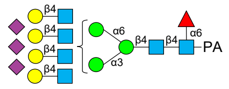	100	24.78	✓		65.29
50	66.23	19.14	73.40	1446.40	1447.04	2H+	2883.03	N4H5Hn7dH2	teN-F-Ldn-**BI**BsF(6)	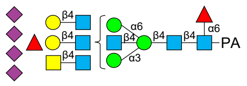	2.75	0.68		Ldn	
	66.23	19.14	73.40	1376.14	1376.52	3H+	4126.54	N4H3Hn11dH1	teN-teLdn-**M3**BsF(6)	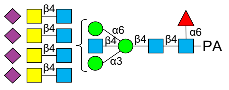	2.05	0.51		Ldn	
	66.23	19.14	73.40	1253.91	1254.13	3H+	3777.39	N4H7Hn6dH1	trN-**TEF**(6)		1.21	0.30			
51	67.95	20.19	75.05	1446.64	1447.04	2H+	2883.03	N4H5Hn7dH2	teN-F-Ldn-**BI**BsF(6)	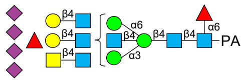	6.60	1.64		Ldn	68.10
	67.95	20.19	75.05	1203.51	1203.09	3H+	3606.26	S1N2H7Hn7dH2	S-dN-F-**TE**BsF(6)	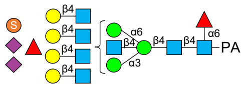	1.97	0.49			
52	69.86	21.46	76.88	1467.23	1467.51	2H+	2933.01	S1N2H5Hn6dH	S-dN-Ldn-**BIF**(6)	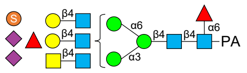	16.02	3.97		Ldn	70.06
	69.86	21.46	76.88	1375.92	1376.52	3H+	4144.57	N4H3Hn11dH1	teN-teLdn-**M3**BsF(6)	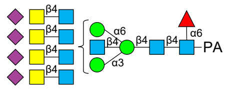	6.49	1.61		Ldn	
53	72.35	23.32	79.27	1446.69	1447.04	2H+	2883.03	N4H5Hn7dH2	teN-F-Ldn-**BI**BsF(6)	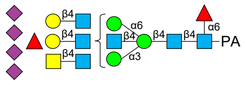	9.07	2.25		Ldn	72.34
54	73.42	24.20	80.30	1447.36	1447.04	2H+	2883.03	N4H5Hn7dH2	teN-F-Ldn-**BI**BsF(6)	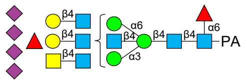	1.61	0.40		Ldn	
55	75.94	26.49	82.72	1446.90	1447.04	2H+	2883.03	N4H5Hn7dH2	teN-F-Ldn-**BI**BsF(6)	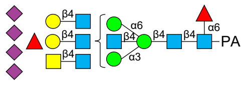	1.77	0.44		Ldn	

Table S2-3. DEAE A5 fraction of *N*-glycans prepared from human urine using the improved method.

Peak No.	RT (min)	GU	RP	*m/z*			MS	Composition (+PA)	Glycan structure		Quantity ratio		MS^2	Note	Hydrazine
		experi-mental	experi-mental	experi-mental	theoretical	ion form					% for top	% for whole			RT (min)
56	45.83	10.46	53.13	1191.28	1191.41	2H+	2380.80	S1N2H5Hn4	S-dN-**BI**	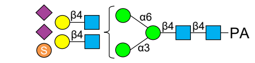	35.97	11.07			46.10
57	54.52	13.58	61.89	1291.36	1291.86	3H+	3872.40	N5H5Hn6dH2	peN-F-Ldn-**BI**F(6)	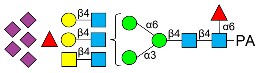	62.71	19.30			
58	58.92	15.43	66.32	1183.88	1184.09	3H+	3549.24	S1N2H7Hn6dH3	S-dN-dF-**TE**F(6)	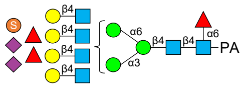	100	30.78			59.31
59	61.40	16.58	68.76	1184.18	1184.09	3H+	3549.24	S1N2H7Hn6dH3	S-dN-dF-**TE**F(6)	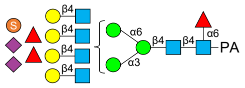	51.21	15.76			
60	64.50	18.17	71.74	1261.14	1260.92	2H+	2519.82	S2H5Hn5dH3	dS-dF-**BI**BsF(6)	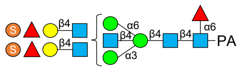	13.75	4.23			
61	68.11	20.29	75.20	1358.09	1357.98	2H+	2713.94	S1N2H4Hn5dH2	S-dN-F-Ldn-**MO**F(6)	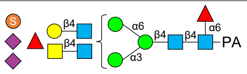	19.52	6.01			
	69.45	21.18	76.49	1340.86							25.04	7.71			
62	71.82	22.90	78.76	1132.17	1132.41	3H+	3412.26	N4H6Hn5dH1	teN-**TR**F(6)	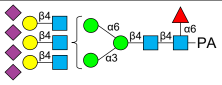	9.44	2.91			
63	77.48	28.08	84.19	1132.24	1132.41	3H+	3412.26	N4H6Hn5dH1	teN-**TR**F(6)	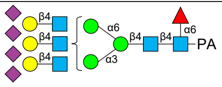	7.24	2.23			

Table S3. *N*-glycans from human serum pool analyzed using the improved method.
Structures highlighted in blue span two fluorescence peaks with a lower abundance ratio; their area values were combined with the corresponding peak having the higher ratio. GU and RP indicate the normalized elution times based on PA-isomaltooligosaccharides and PA-*N*-glycan core structures, respectively. "Experimental" refers to the measured values, and "std." indicates standard values listed in SugarScan. "Composition" indicates the following components: Ac (acetyl group), S (sulfate group), P (phosphate group), N (NeuAc), H (Hex), Hn (HexNAc), and dH (deoxyHex). The "Quantity ratio" refers to the relative yield calculated from fluorescence peak areas. "% for top" indicates the yield relative to the most abundant glycan in the sample, while "% for whole" indicates the yield as a proportion of the entire sample. In the MS^2 column, a check mark (✓) indicates glycan structures for which MS/MS data were obtained, while a dash (–) indicates that MS/MS data were acquired but insufficient for structural assignment. The "Note" column highlights characteristic substructures.

RT (min)	GU		RP		*m/z*			MS	Composition (+PA)		Glycan structure	Quantity ratio		MS^2	Note
	experi-mental	std.	experi-mental	std.	experi-mental	theoretical	ion form					% for top	% for whole		
23.57	5.10	5.11	29.95	29.07	889.75	900.33	2H+	1798.65	H8Hn2	**M8A**	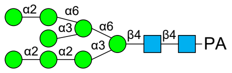	1.348	0.477	✓	
23.57	5.10		29.95		1186.44		2H+	2371.88	N2H2Hn2+1059.16			0.099	0.035	✓	
24.52	5.25	5.31	30.86	30.36	818.93	819.31	2H+	1636.60	H7Hn2	**M7A**					
24.75	5.29	5.31	31.08	30.36	818.94	819.31	2H+	1636.60	H7Hn2	**M7A**	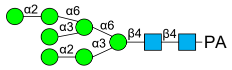	1.263	0.447		
24.75	5.29		31.08		903.07	904.34	2H+	1806.67	N1H5Hn3	6N-Ln-**M4C**/ga	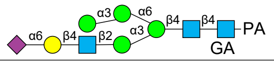			✓	
24.75	5.29	5.82	31.08		985.28	985.37	2H+	1968.72	N1H6Hn3	6N-Ln-**M5A**/epi	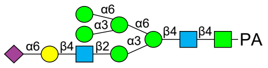	0.009	0.003		
25.92	5.49	5.50	32.20	31.33	980.84	981.36	2H+	1960.70	H9Hn2	**M9A**	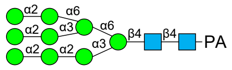	0.814	0.288	✓	
27.41	5.75	5.76	33.63	32.76	899.86	900.33	2H+	1798.65	H8Hn2	**M8B**	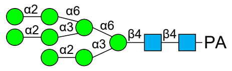	0.368	0.130		
28.12	5.88	5.88	34.31	33.44	818.85	819.31	2H+	1636.60	H7Hn2	**M7B**	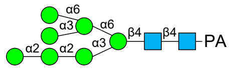	0.347	0.123		
28.12	5.88		34.31		1106.58	1107.42	2H+	2212.83	N1H5Hn5	N-**BI**Bs/ga	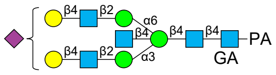				
29.34	6.12	6.11	35.48	34.37	737.81	738.28	2H+	1474.54	H6Hn2	**M6B**	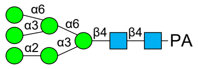	2.818	0.997	✓	
29.89	6.23		36.01		859.24	859.99	3H+	2576.96	N2H4Hn4dH3	N-**TR**Bs/ga	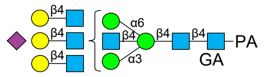				
29.89	6.23		36.01		1142.83		2H+					0.084	0.030	✓	
29.89	6.23	6.11	36.01	34.37	738.03	738.28	2H+	1474.54	H6Hn2	**M6B**					
31.96	6.67	6.72	38.00		1004.97	1005.88	2H+	2009.75	N1H5Hn4	06N-**BI**/ga	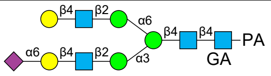			✓	
32.62	6.81	6.72	38.63		1005.12	1005.88	2H+	2009.75	N1H5Hn4	06N-**BI**/ga	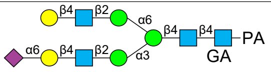			✓	
32.62	6.81		38.63		1223.49	1224.46	2H+	2446.90	N2H5Hn4dH1	66N-**BI**F(6)/epi	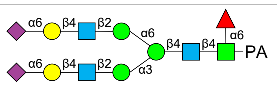	0.079	0.028		
32.62	6.81	6.80	38.63	37.73	818.88	819.31	2H+	1636.60	H7Hn2	**M7D**	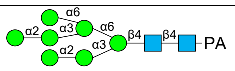	0.045	0.016		
33.12	6.93	6.92	39.11		1005.84	1005.88	2H+	2009.75	N1H5Hn4	06N-**BI**/epi	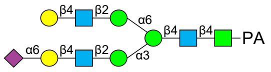	0.516	0.183	✓	
33.12	6.93	7.02	39.11	38.72	656.95	657.25	2H+	1312.49	H5Hn2	**M5A**					
33.58	7.04	7.01	39.58	38.72	1313.44	1313.50	H+	1312.49	H5Hn2	**M5A**	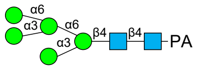	3.480	1.232	✓	
33.58	7.04	6.92	39.58		1005.96	1005.88	2H+	2009.75	N1H5Hn4	06N-**BI**/epi				✓	
34.31	7.21		40.35		1004.93	1005.88	2H+	2009.75	N1H5Hn4	30N-**BI**/ga	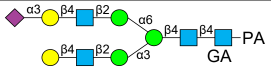			✓	

Table S3 continued.

RT (min)	GU experi-mental	GU std.	RP experi-mental	RP std.	m/z experi-mental	m/z theoretical	m/z ion form	MS	Composition (+PA)		Glycan structure	Quantity ratio % for top	Quantity ratio % for whole	MS2	Note
35.68	7.55	7.56	41.81		1150.60	1151.43	2H+	2300.84	N2H5Hn4	66N-**BI**/ga	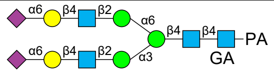			✓	
37.08	7.92	7.88	43.29		1151.44	1151.43	2H+	2300.84	N2H5Hn4	66N-**BI**/epi	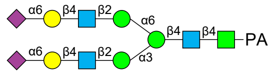	6.479	2.293	✓	
37.08	7.92	7.90	43.29		737.92	738.28	2H+	1474.54	H6Hn2	**M6C**	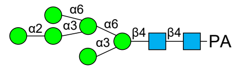	0.309	0.109	✓	
37.08	7.92	7.93	43.29		1188.47	1188.45	2H+	2374.88	N1H6Hn5	006N-**TR124**	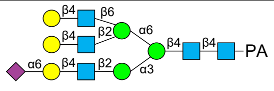	0.127	0.045	✓	
37.60	8.06	8.30	43.85		1150.47	1151.43	2H+	2300.84	N2H5Hn4	36N-**BI**/ga	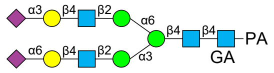			✓	
37.60	8.06	8.03	43.85	42.84	985.30	985.37	2H+	1968.72	N1H6Hn3	6N-Ln-**M5A**	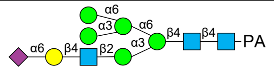	0.359	0.127	✓	
37.60	8.06	8.09	43.85	42.84	823.34	823.31	2H+	1644.61	N1H4Hn3	6N-**MO1**	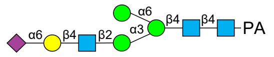	0.122	0.043		
38.20	8.22	8.25	44.48	43.68	904.16	904.34	2H+	1806.67	N1H5Hn3	6N-Ln-**M4C**	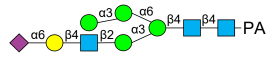	6.295	2.228	✓	
38.20	8.22	8.28	44.48		771.08	771.31	2H+	1540.60	H3Hn4dH1	**AG12**F(6)/epi	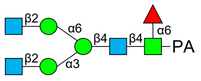	0.935	0.331	✓	
38.20	8.22		44.48		1150.51	1151.43	2H+	2300.84	N2H5Hn4	36N-**BI**/ga				✓	
38.60	8.34	8.30	44.91		1151.39	1151.43	2H+	2300.84	N2H5Hn4	36N-**BI**/epi	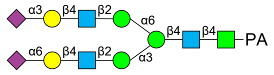	0.259	0.092		
38.60	8.34	8.25	44.91	43.68	904.42	904.34	2H+	1806.67	N1H5Hn3	6N-Ln-**M4C**					
38.60	8.34		44.91		1107.82	1108.41	3H+	3322.20	N3H6Hn5	trN-**TE**/ga	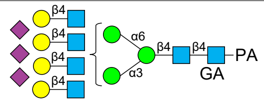				
39.73	8.66	8.67	46.11		770.33	771.31	2H+	1540.60	H3Hn4dH1	**AG12**F(6)/ga	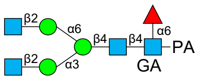			✓	
39.73	8.66	8.66	46.11		852.39	852.33	2H+	1702.66	H4Hn4dH1	**BI**F(6)-Ga/epi	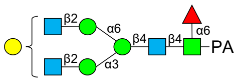	0.632	0.224	✓	
39.73	8.66	9.00	46.11		889.10	889.67	3H+	2665.97	N2H6Hn5	dN-**TR**	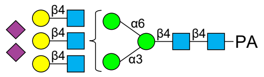	0.119	0.042		
39.73	8.66		46.11		1205.02	1205.44	3H+	3613.30	N4H7Hn6	teN-**TE**	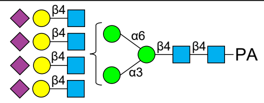	0.073	0.026		
39.73	8.66		46.11		938.03	938.35	3H+	2812.03	N2H6Hn4dH1	dN-F-**TR**/ga	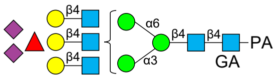				
39.73	8.66		46.11		792.60	792.63	3H+	2374.88	N1H6Hn5	N-**TR**	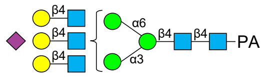	0.026	0.009		
41.08	9.05	9.02	47.54		851.49	852.33	2H+	1702.66	H4Hn4dH1	**BI**F(6)-Ga/ga	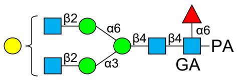			✓	
41.08	9.05		47.54		1034.82	1035.38	3H+	3103.13	N3H6Hn5dH1	trN-**TR**F(6)/ga	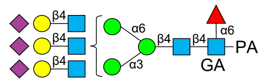			✓	
41.08	9.05	9.00	47.54		889.94	889.67	3H+	2665.97	N2H6Hn5	dN-**TR**	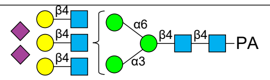	0.697	0.247	✓	
41.08	9.05		47.54		1106.30	1106.38	3H+	3016.13	N1H6Hn6dH3	N-trF-**TRBs**	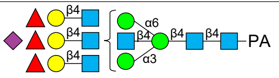	0.246	0.087		
42.06	9.35		48.58		986.06	986.70	3H+	2957.07	N3H6Hn5	trN-**TR**/ga	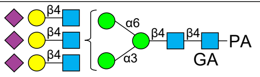			✓	
42.06	9.35		48.58		1035.51	1035.38	3H+	3103.13	N3H6Hn5dH1	trN-F-**TR**	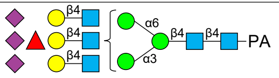	0.703	0.249		

Table S3 continued.

RT (min)	GU experi-mental	GU std.	RP experi-mental	RP std.	*m/z* experi-mental	*m/z* theoretical	*m/z* ion form	MS	Composition (+PA)		Glycan structure	Quantity ratio % for top	Quantity ratio % for whole	MS^2	Note
42.06	9.35	9.34	48.58		932.41	933.36	2H+	1864.71	H5Hn4dH1	**BI**F(6)/ga	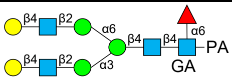			✓	
42.06	9.35	9.30	48.58		889.36	889.67	3H+	2665.97	N2H6Hn5	dN-**TR**	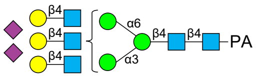	0.619	0.219		
42.06	9.35	9.33	48.58		779.18	779.31	3H+	1556.60	H4Hn2	**BI**-Ga2	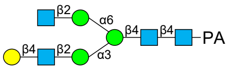	0.265	0.094	✓	
43.12	9.68		49.71		986.19	986.70	3H+	2957.07	N3H6Hn5	trN-**TR**/ga	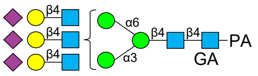			✓	
43.12	9.68	9.63	49.71	48.64	924.87	924.85	2H+	1847.69	N1H4Hn4	06N-**BI**-Ga2	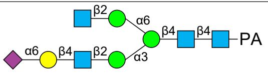	0.828	0.293	✓	
43.12	9.68	9.71	49.71		889.86	889.67	3H+	2665.97	N2H6Hn5	33N-**TR**	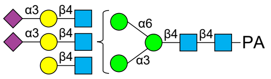	0.034	0.012	✓	
43.12	9.68		49.71		1205.54	1205.44	3H+	3613.30	N4H7Hn6	teN-**TE**	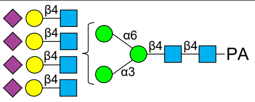	0.009	0.003		
43.55	9.81		50.17		986.11	986.70	3H+	2957.07	N3H6Hn5	trN-**TR**/ga					
43.55	9.81		50.17		914.44	914.35	3H+	2740.01	N1H7Hn6	N-**TE**	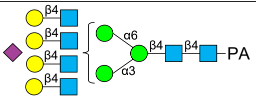	0.086	0.030		
43.55	9.81		50.17		1060.12	1060.06	3H+	3177.16	N2H7Hn6dH1	dN-F-**TE**	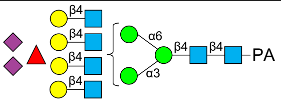	0.062	0.022		
44.37	10.08	9.98	51.04		1224.50	1224.46	2H+	2446.90	N2H5Hn4dH1	?3N-F(3)1-**BI**	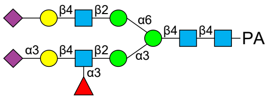	0.308	0.109	✓	SLe^x
44.37	10.08		51.04		986.66	986.70	3H+	2957.07	N3H6Hn5	trN-**TR**	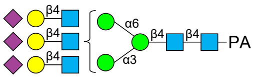	0.268	0.095	✓	
44.37	10.08	10.05	51.04		860.43	860.33	2H+	1718.65	H5Hn4	**BI**	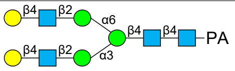	0.187	0.066	✓	
44.37	10.08	10.14	51.04		1077.70	1078.91	2H+	2155.80	N1H5Hn4dH1	06N-**BI**F(6)/ga				✓	
45.18	10.34	10.29	51.88	50.87	1006.01	1005.88	2H+	2009.75	N1H5Hn4	06N-**BI**	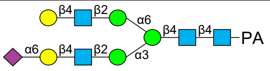	19.649	6.954	✓	
45.18	10.34		51.88		1354.48	1354.53	H+	1353.52	H4Hn3	**MO**/fr				✓	
45.18	10.34	10.14	51.88		1078.02	1078.91	2H+	2155.80	N1H5Hn4dH1	06N-**BI**F(6)/ga	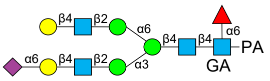			✓	
45.18	10.34	9.82	51.88		1340.68	1341.49	2H+	1340.49	H5Hn2Fo1	Fo-**M5A**	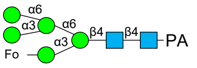	0.186	0.066		
45.95	10.59	10.55	52.64		816.11	816.64	3H+	2446.90	N2H5Hn4dH1	66N-**BI**F(6)/epi	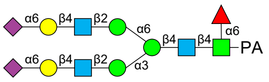	1.297	0.459	✓	
45.95	10.59	10.55	52.64		1011.63	1011.38	3H+	3031.11	N1H7Hn6dH2	dN-**TE**	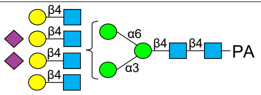	0.550	0.195	✓	
45.95	10.59		52.64		986.14	986.70	3H+	2957.07	N3H6Hn5	trN-**TR**/ga	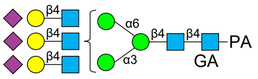			✓	
45.95	10.59	10.29	52.64	50.87	1005.93	1005.88	2H+	2009.75	N1H5Hn4	06N-**BI**				✓	
45.95	10.59		52.64		1157.31	1157.09	3H+	3468.26	N3Hn7H6dH1	trN-F-**TE**	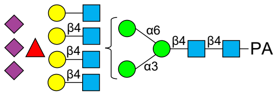	0.020	0.007		
47.05	10.96		53.72		986.72	986.70	3H+	2957.07	N3H6Hn5	trN-**TR**	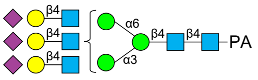	0.454	0.161	✓	

Table S3 continued.

RT (min)	GU		RP		*m/z*			MS	Composition (+PA)		Glycan structure	Quantity ratio		MS²	Note
	experi-mental	std.	experi-mental	std.	experi-mental	theoretical	ion form					% for top	% for whole		
47.05	10.96	10.91	53.72		816.22	816.64	3H+	2446.90	N2H5Hn4dH1	36N-**BI**F(6)/ga	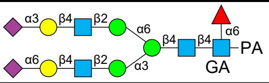			✓	
47.05	10.96	10.55	53.72		1011.54	1011.38	3H+	3031.11	N2H7Hn6	dN-**TE**				✓	
47.05	10.96		53.72		1254.35	1254.13	3H+	3759.35	N4H7Hn6dH1	teN-F-**TE**					
47.54	11.12		54.20		1254.11	1254.13	3H+	3759.35	N4H7Hn6dH1	teN-F-**TE**	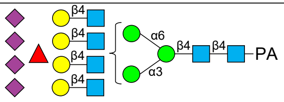	0.861	0.305		
47.54	11.12	11.06	54.20		816.74	816.64	3H+	2446.90	N2H5Hn4dH1	66N-**BI**F(6)/epi	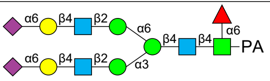	0.744	0.263	✓	
47.54	11.12		54.20		1108.65	1108.41	3H+	3322.20	N3H7Hn6	N-3N-6N-Ln-**TR**	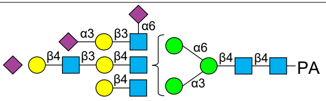	0.219	0.077	✓	dN-Ln, dLn
47.54	11.12	10.91	54.20		1077.84	1078.91	2H+	2155.80	N1H5Hn4dH1	30N-**BI**-Ga1	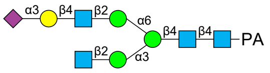	0.044	0.015		
47.54	11.12	10.55	54.20		1011.53	1011.38	3H+	3031.11	N2H7Hn6	dN-**TE**	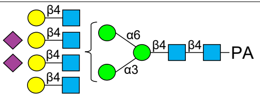	0.021	0.007		
47.54	11.12		54.20		986.91	986.70	3H+	2957.07	N3H6Hn5	trN-**TR**	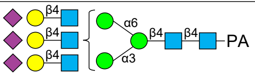	0.018	0.006		
48.35	11.40		55.00		1108.51	1108.41	3H+	3322.20	N3H7Hn6	N-3N-6N-Ln-**TR**	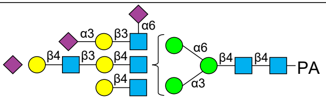	0.344	0.122	✓	dN-Ln, dLn
48.35	11.40	11.49	55.00	54.54	1151.75	1151.43	2H+	2300.84	N2H5Hn4	66N-**BI**					
48.35	11.40	10.55	55.00		1011.69	1011.38	3H+	3031.11	N2H7Hn6	dN-**TE**					
49.07	11.65	11.49	55.71	54.54	1151.78	1151.43	2H+	2300.84	N2H5Hn4	66N-**BI**	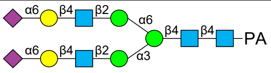	100	35.391	✓	
49.07	11.65		55.71		1108.74	1108.41	3H+	3322.20	N3H7Hn6	N-3N-6N-Ln-**TR**	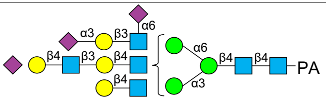	0.095	0.034	✓	dN-Ln, dLn
50.55	12.17	12.02	57.16	56.14	1151.51	1151.43	2H+	2300.84	N2H5Hn4	36N-**BI**	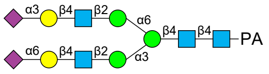	11.871	4.201	✓	
50.55	12.17	12.00	57.16	56.09	771.37	771.31	2H+	1540.60	H3Hn4dH1	**AG12**F(6)	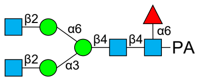	7.154	2.532	✓	
50.55	12.17	13.10	57.16	59.36	1338.48	1338.53	H+	1337.52	H3Hn3dH1	**AG2**F(6)	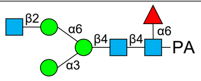	2.529	0.895	✓	
50.55	12.17	11.98	57.16	55.95	1005.98	1005.88	2H+	2009.75	N1H5Hn4	30N-**BI**	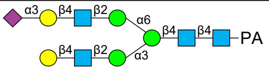	1.882	0.666	✓	
50.55	12.17	12.08	57.16	57.74	1205.59	1205.44	3H+	3613.30	N4H7Hn6	teN-**TE**	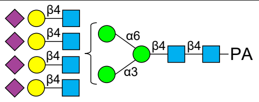	1.274	0.451	✓	
52.10	12.73	13.08	58.69		1034.86	1035.38	3H+	3103.13	N3H6Hn5dH1	trN-F-**TR**	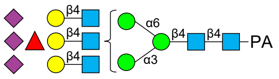	0.106	0.038		
52.10	12.73	12.27	58.69	56.80	1005.79	1005.88	2H+	2009.75	N1H5Hn4	03N-**BI**	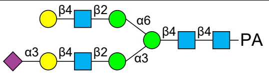	0.101	0.036		
52.10	12.73	12.73	58.69		1188.27	1188.45	2H+	2374.88	N1H6Hn5	N-Ldn-**BI**	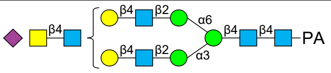	0.053	0.019		Ldn
52.10	12.73	13.08	58.69		852.02	852.33	2H+	1702.66	H4Hn4dH1	**BI**F(6)-Ga2					
52.76	12.97	13.08	59.34		852.38	852.33	2H+	1702.66	H4Hn4dH1	**BI**F(6)-Ga2	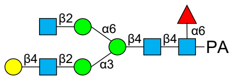	14.543	5.147	✓	
52.76	12.97	13.02	59.34		938.61	938.35	3H+	2812.03	N2H6Hn4dH1	dN-**TR**F(6)	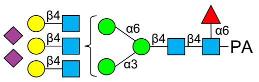	0.158	0.056		
52.76	12.97		59.34		874.74		2H+		Hn-H-Hn-(S-Hn-H-)+1017.13			0.147	0.052	✓	

Table S3 continued.

RT (min)	GU experi-mental	GU std.	RP experi-mental	RP std.	*m/z* experi-mental	*m/z* theoretical	*m/z* ion form	MS	Composition (+PA)	Glycan structure		Quantity ratio % for top	Quantity ratio % for whole	MS^2	Note
53.64	13.29	13.42	60.20		1026.26	1026.39	2H+	2050.77	N1H4Hn5	06N-**BI**Bs-Ga2	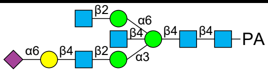	0.497	0.176	✓	
53.64	13.29	13.02	60.20		938.49	938.35	3H+	2812.03	N2H6Hn4dH1	dN-**TR**F(6)	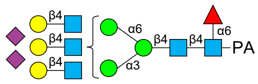	0.035	0.012		
53.64	13.29	13.59	60.20	61.78	1205.50	1205.44	3H+	3613.30	N4H7Hn6	teN-**TE**	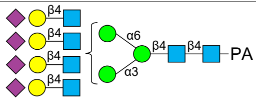	0.014	0.005		
53.64	13.29		60.20		852.42	852.33	2H+	1702.66	H4Hn4dH1	**BI**F(6)-Ga2					
55.12	13.85	13.61	61.66	60.83	933.49	933.36	2H+	1864.71	H5Hn4dH1	**BI**F(6)	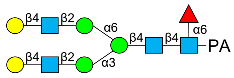	10.367	3.669	✓	
55.12	13.85	13.80	61.66	62.30	1035.19	1035.38	3H+	3103.13	N3H6Hn5dH1	trN-F-**TR**				✓	SLe^x
55.12	13.85		61.66		883.96	884.33	3H+	2649.98	N2H5Hn5dH1	66N-**BI**BsF(6)/epi				✓	
55.12	13.85		61.66		889.73	889.67	3H+	2665.97	N2H6Hn5	dN-**TR**	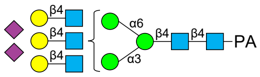	0.067	0.024		
55.12	13.85	13.35	61.66	60.99	1151.16	1151.43	2H+	2300.84	N2H5Hn4	63N-**BI**	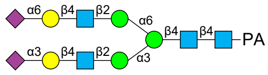	0.044	0.016		
55.12	13.85	13.72	61.66		997.73	997.88	2H+	1993.75	N1H4Hn4dH1	06N-**BI**F(6)-Ga2	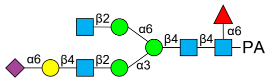	0.025	0.009		
55.46	13.97	13.80	61.99		1035.51	1035.38	3H+	3103.13	N3H6Hn5dH1	dN-3N-F(3)-**TR**	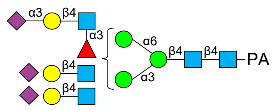	6.937	2.455	✓	SLe^x
55.46	13.97		61.99		883.94	884.33	3H+	2649.98	N2H5Hn5dH1	66N-**BI**BsF(6)/epi	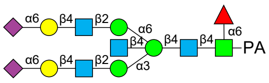	1.238	0.438	✓	
55.46	13.97	14.01	61.99		889.73	889.67	3H+	2665.97	N2H6Hn5	66N-Ln-**BI**	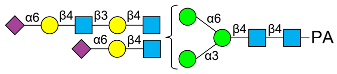	0.752	0.266	✓	N-dLn
55.46	13.97	13.61	61.99	60.83	933.50	933.36	2H+	1864.71	H5Hn4dH1	**BI**F(6)					
55.46	13.97	14.23	61.99	62.56	1151.94	1151.43	2H+	2300.84	N2H5Hn4	33N-**BI**	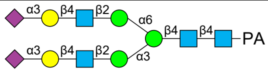	0.046	0.016		
56.65	14.43	14.52	63.16	62.25	1078.94	1078.91	2H+	2155.80	N1H5Hn4dH1	06N-**BI**F(6)	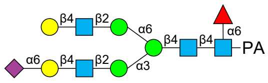	10.573	3.742	✓	
56.65	14.43	14.45	63.16		889.86	889.67	3H+	2665.97	N2H6Hn5	66N-Ln-**BI**	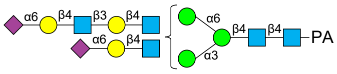	2.090	0.740	✓	N-dLn
56.65	14.43	14.36	63.16	63.69	1035.65	1035.38	3H+	3103.13	N3H6Hn5dH1	trN-F-**TR**	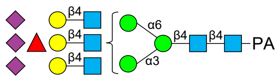	0.102	0.036		
57.85	14.91	14.96	64.34		1107.84	1107.42	2H+	2212.83	N1H5Hn5	06N-**BI**Bs	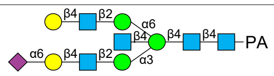	0.348	0.123	✓	
57.85	14.91	14.99	64.34	65.17	986.80	986.70	3H+	2957.07	N3H6Hn5	trN-**TR**				✓	
58.57	15.19	14.87	65.05	64.27	1224.51	1224.46	2H+	2446.90	N2H5Hn4dH1	66N-**BI**F(6)	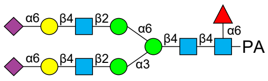	16.518	5.846	✓	
58.57	15.19	14.99	65.05	65.17	986.86	986.70	3H+	2957.07	N3H6Hn5	trN-**TR**	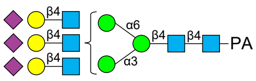	7.422	2.627	✓	
58.57	15.19		65.05		1014.18	1014.05	3H+	3039.12	N3H4Hn7	trN-dLdn-**MO**	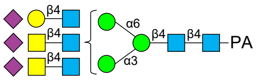	0.458	0.162		Ldn
59.83	15.71	15.57	66.29	66.49	986.85	986.70	3H+	2957.07	N3H6Hn5	trN-**TR**	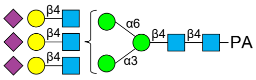	0.550	0.195	✓	
59.83	15.71	16.34	66.29		889.88	889.67	3H+	2665.97	N2H6Hn5	33N-Ln-**BI**	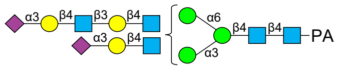	0.090	0.032		N-dLn

Table S3 continued.

RT (min)	GU experi-mental	GU std.	RP experi-mental	RP std.	*m/z* experi-mental	*m/z* theoretical	*m/z* ion form	MS	Composition (+PA)	Glycan name	Glycan structure	Quantity ratio % for top	Quantity ratio % for whole	MS^2	Note
59.83	15.71	15.86	66.29	68.72	1079.05	1078.91	2H+	2155.80	N1H5Hn4dH1	60N-**BI**F(6)	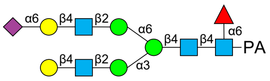	0.029	0.010		
59.83	15.71	16.30	66.29	67.84	1224.80	1224.46	2H+	2446.90	N2H5Hn4dH1	36N-**BI**F(6)	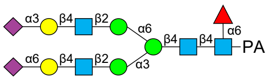	0.005	0.002		
61.53	16.43	16.73	67.94		961.74	961.87	2H+	1921.73	H5Hn5	**BI**Bs	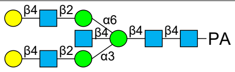	0.254	0.090		
62.84	17.01	16.96	69.13	69.36	986.83	986.70	3H+	2957.07	N3H6Hn5	trN-**TR**	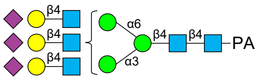	7.014	2.482	✓	
62.84	17.01	17.28	69.13		1078.88	1078.91	2H+	2155.80	N1H5Hn4dH1	03N-**BI**F(6)	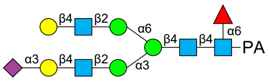	0.004	0.001		
62.84	17.01	17.43	69.13	70.68	1224.52	1224.46	2H+	2446.90	N2H5Hn4dH1	63N-**BI**F(6)	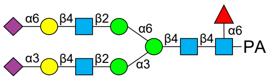	0.002	0.001		
62.84	17.01		69.13		938.40	938.35	3H+	2812.03	N2H6Hn4dH1	dN-**TR**F(6)	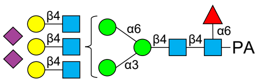	0.001	0.000		
63.94	17.51		70.12		986.78	986.70	3H+	2957.07	N3H6Hn5	trN-**TR**	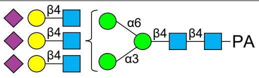	1.448	0.512		
65.37	18.19	18.39	71.42		889.83	889.67	3H+	2665.97	N2H6Hn5	dN-**TR**	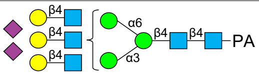	0.699	0.247		
65.37	18.19		71.42		1084.15	1084.07	3H+	3249.18	N3H6Hn5dH2	trN-dF-**TR**	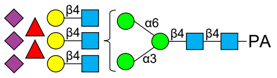	0.037	0.013		
67.03	19.03	19.64	72.92		938.45	938.35	3H+	2812.03	N2H6Hn4dH1	dN-**TR**F(6)	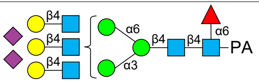	0.588	0.208		
68.50	19.82	19.43	74.25	73.91	1224.80	1224.46	2H+	2446.90	N2H5Hn4dH1	33N-**BI**F(6)	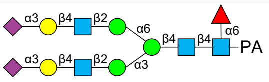	1.335	0.472		
68.50	19.82	20.35	74.25	75.07	1035.42	1035.38	3H+	3103.13	N3H6Hn5dH1	trN-F-**TR**	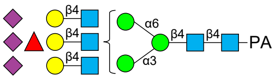	0.454	0.161		
68.50	19.82	20.65	74.25	74.28	1099.12	1099.42	2H+	2196.83	N1H4Hn5dH1	06N-**BI**BsF(6)-Ga2	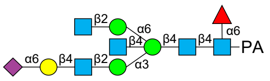	0.042	0.015		
69.47	20.37	20.29	75.13	75.27	873.18	872.85	2H+	1743.68	H3Hn5dH1	**AG12**BsF(6)	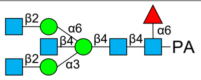	1.394	0.493	✓	
69.47	20.37	20.35	75.13		1035.65	1035.38	3H+	3103.13	N3H6Hn5dH1	trN-F-**TR**					
70.44	20.95		76.00		889.85	889.67	3H+	2665.97	N2H6Hn5	dN-**TR**	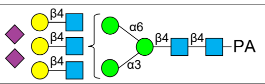	1.448	0.512		
72.25	22.11	21.99	77.64	77.61	1180.45	1180.45	2H+	2358.88	N1H5Hn5dH1	06N-**BI**BsF(6)	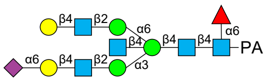	6.155	2.178	✓	
72.25	22.11	22.47	77.64		953.81	953.87	2H+	1905.73	H4Hn5dH1	**BI**BsF(6)-Ga2	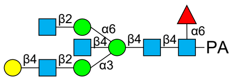	0.088	0.031	✓	
72.25	22.11	22.02	77.64	77.39	1035.55	1035.38	3H+	3103.13	N3H6Hn5dH1	trN-F-**TR**	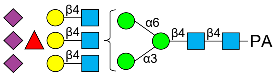	0.048	0.017		
73.13	22.72	23.29	78.44		884.53	884.33	3H+	2649.98	N2H5Hn5dH1	66N-**BI**BsF(6)	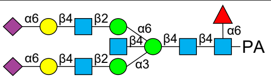	4.253	1.505	✓	
73.13	22.72	22.65	78.44		889.80	889.67	3H+	2665.97	N2H6Hn5	33N-Ln-**BI**F(6)	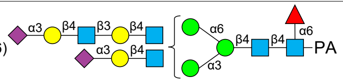	0.337	0.119		N-dLn
73.13	22.72	22.47	78.44		953.55	953.87	2H+	1905.73	H4Hn5dH1	**BI**BsF(6)-Ga2					
74.35	23.61	23.84	79.54	80.00	1035.29	1034.90	2H+	2067.79	H5Hn5dH1	**BI**BsF(6)	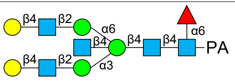	1.469	0.520	✓	

Table S3 continued.

RT (min)	GU		RP		*m/z*			MS	Composition (+PA)	Glycan structure	Quantity ratio		MS^2	Note
	experi-mental	std.	experi-mental	std.	experi-mental	theoretical	ion form				% for top	% for whole		
74.35	23.61	24.25	79.54		938.25	938.35	3H+	2812.03	N2H6Hn4dH1	dN-**TR**F(6) 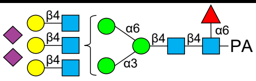	0.038	0.013		
75.92	24.86	25.85	80.96	81.49	1099.54	1099.42	2H+	2196.83	N1H4Hn5dH1	30N-**BI**BsF(6)-Ga1 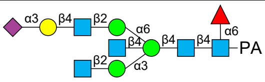	0.323	0.114		
77.36	26.13	27.33	82.26	83.11	1180.30	1180.45	2H+	2358.88	N1H5Hn5dH1	30N-**BI**BsF(6) 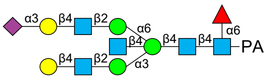	0.238	0.084		
78.26	26.98	26.16	83.07		1172.83	1172.43	2H+	2342.85	N2H5Hn4Ac1	Ac-66N-**BI** 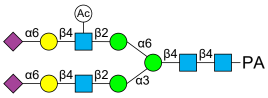	0.293	0.104		
83.00	32.42		87.36		1060.83	1060.06	3H+	3177.16	N2H7Hn6dH1	dN-**TE**F(6) 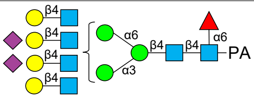	0.059	0.021		

Table S4. *N*-glycans from human urine M1 analyzed using the improved method.

RT (min)	GU experimental	GU std.	RP experimental	RP std.	*m/z* experimental	*m/z* theoretical	*m/z* ion form	MS	Composition (+PA)		Glycan structure	Quantity ratio % for top	Quantity ratio % for whole	MS^2	Only	Note
23.62	5.17	5.11	30.61	29.07	899.85	900.33	2H+	1798.65	H8Hn2	**M8A**	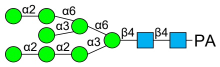	1.331	0.115	✓		
23.62	5.17		30.61		1186.09		2H+		N2H2Hn2+1059.16			1.010	0.087	✓		
24.81	5.36	5.31	31.75	30.36	818.75	819.31	2H+	1636.60	H7Hn2	**M7A**	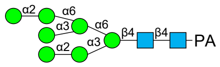	2.354	0.204	✓		
25.30	5.44		32.23		1040.79	1041.89	2H+	2081.77	N2H4Hn3dH1	dN-F-Ln-**M2'**	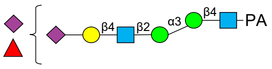	0.281	0.024			
26.04	5.57	5.50	32.94	31.33	980.83	981.36	2H+	1960.70	H9Hn2	**M9A**	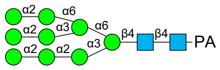	11.664	1.009	✓		
26.04	5.57		32.94		1186.02		2H+					4.370	0.378			
26.83	5.70		33.70		1231.35	1231.45	H+	1230.44	H7	**Glc7**	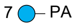	0.544	0.047	✓		
27.47	5.82	5.76	34.32	32.76	899.39	900.33	2H+	1798.65	H8Hn2	**M8B**	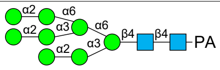	0.439	0.038	✓		
28.13	5.94	5.88	34.95	33.44	818.97	819.31	2H+	1636.60	H7Hn2	**M7B**	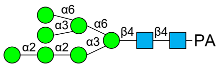	12.973	1.122	✓		
28.13	5.94		34.95		1231.42	1231.45	H+	1230.44	H7	**Glc7**	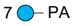	0.393	0.034	✓		
28.13	5.94		34.95		1106.51	1107.42	2H+	2212.83	N1H5Hn5	N-**BIBs**/ga	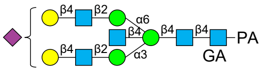					
29.30	6.16	6.11	36.08	34.37	737.81	738.28	2H+	1474.54	H6Hn2	**M6B**	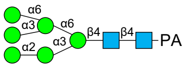	52.975	4.580	✓		
30.94	6.49	6.48	31.75		899.90	900.33	2H+	1798.65	H8Hn2	**M8C**	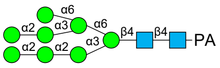	0.445	0.038			
31.92	6.69	6.72	38.60		1004.70	1005.88	2H+	2009.75	N1H5Hn4	06N-**BI**/ga	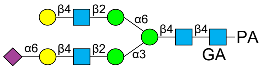			✓		
32.68	6.86		37.66		1005.32	1005.88	2H+	2009.75	N1H5Hn4	06N-**BI**/epi	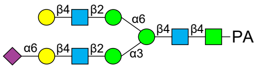	0.247	0.021			
32.68	6.86		37.66		1135.33	1135.45	H+	1134.44	H3Hn2dH1	**M3B**F(6)/epi	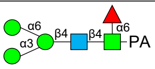	0.163	0.014			
32.68	6.86	6.80	37.66	37.73	818.59	819.31	2H+	1636.60	H7Hn2	**M7D**	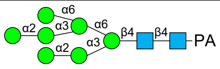	0.130	0.011			
32.68	6.86	7.08	37.66	38.72	989.26	989.39	H+	988.39	H3Hn2	**M3B**						
33.35	7.01	7.00	40.00	38.72	1313.32	1313.50	H+	1312.49	H5Hn2	**M5A**	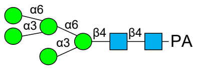	17.646	1.526	✓		
33.35	7.01	7.08	40.00	38.72	989.30	989.39	H+	988.39	H3Hn2	**M3B**	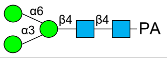	6.831	0.591			
33.35	7.01		40.00		971.24							6.114	0.529			
33.35	7.01	7.04	40.00	38.72	827.29	827.34	H+	826.33	H2Hn2	**M2B**	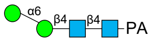	5.809	0.502	✓		
33.35	7.01		40.00		1133.26	1135.45	H+	1134.44	H3Hn2dH1	**M3B**F(6)/ga	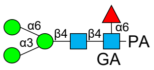					
33.35	7.01	6.72	40.00		1151.30	1151.45	H+	1150.44	H4Hn2	**M4A**	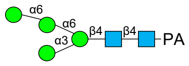	0.554	0.048			
34.04	7.16	7.06	40.73		1151.40	1151.45	H+	1150.44	H4Hn2	**M4B**	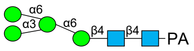	0.599	0.052	✓		
34.04	7.16		40.73		1004.58	1005.88	2H+	2009.75	N1H5Hn4	30N-**BI**/ga	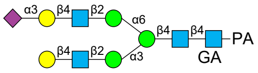			✓		
34.04	7.16		40.73		1313.34	1313.50	H+	1312.49	H5Hn2	**M5A**		0.055	0.005			
34.04	7.16	7.00	40.73		1133.23	1135.45	H+	1134.44	H3Hn2dH1	**M3B**F(6)/ga						
35.42	7.49		42.18		1150.26	1151.43	2H+	2300.84	N2H5Hn4	66N-**BI**/ga						
35.42	7.49		42.18		1004.61	1005.88	2H+	2009.75	N1H5Hn4	30N-**BI**/ga						

Table S4 continued.

RT (min)	GU experi-mental	GU std.	RP experi-mental	RP std.	m/z experi-mental	m/z theoretical	m/z ion form	MS	Composition (+PA)		Glycan structure	Quantity ratio % for top	Quantity ratio % for whole	MS²	Only	Note
35.86	7.60	7.56	42.65		1150.92	1151.43	2H+	2300.84	N2H5Hn4	66N-**BI**/ga	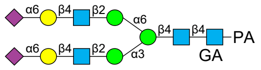			✓		
37.06	7.90	7.88	43.92		1151.33	1151.43	2H+	2300.84	N2H5Hn4	66N-**BI**/epi	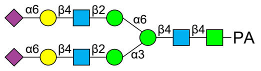	2.744	0.237	✓		
37.58	8.04		44.47		1151.28	1151.43	2H+	2300.84	N2H5Hn4	36N-**BI**/epi	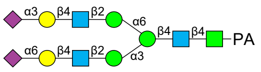	7.989	0.691	✓		
37.58	8.04	8.03	44.47		985.29	985.37	2H+	1968.72	N1H6Hn3	6N-Ln-**M5A**	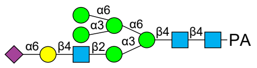	6.463	0.559	✓		
37.58	8.04	8.09	44.47	42.84	823.33	823.31	2H+	1644.61	N1H4Hn3	6N-**MO1**	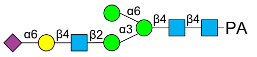	5.508	0.476	✓		
37.58	8.04		44.47		1179.28	1179.41	2H+	2356.80	P1N1H7Hn3dH1	6P-N-Ln-**M6**F(6)	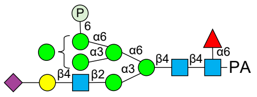	3.705	0.320			
38.16	8.19	8.30	45.08	43.68	1151.50	1151.43	2H+	2300.84	N2H5Hn4	36N-**BI**/ga	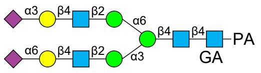					
38.16	8.19		45.08		1078.76	1078.91	2H+	2155.80	N1H5Hn4dH1	3N-F(3)-**BI**	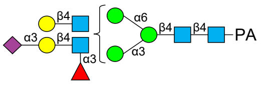	0.659	0.057	✓		SLe^x
38.16	8.19	8.25	45.08		904.40	904.34	2H+	1806.67	N1H5Hn3	6N-Ln-**M4C**	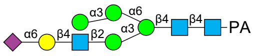	0.622	0.054	✓		
39.51	8.55		46.50		1150.49	1151.43	2H+	2300.84	N2H5Hn4	dN-**BI**/ga	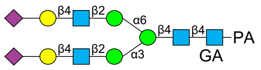					
39.51	8.55		46.50		895.14	895.34	2H+	1790.67	N1H4Hn3dH1	N-**MO**F(6)	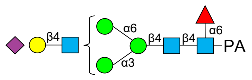	0.163	0.014			
39.51	8.55		46.50		1179.99	1179.41	2H+	2356.80	P1N1H7Hn3dH1	6P-N-Ln-**M6**F(6)	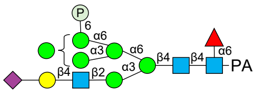	0.105	0.009			
39.65	8.59		46.65		1150.59	1151.43	2H+	2300.84	N2H5Hn4	dN-**BI**/ga	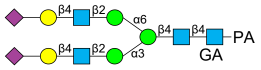			✓		
39.65	8.59	8.64	46.65		1079.11	1079.42	2H+	2156.82	H5Hn4dH3	F(2)-F(3)-**BI**F(6)	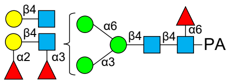	0.632	0.055			Le^y
39.65	8.59		46.65		1179.79	1179.41	2H+	2356.80	P1N1H7Hn3dH1	6P-N-Ln-**M6**F(6)	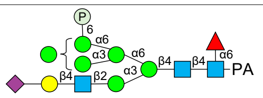	0.385	0.033			
40.44	8.82		47.49		1332.88	1333.99	2H+	2665.97	N2H6Hn5	dN-**TR**/ga	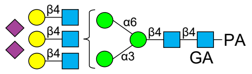					
40.44	8.82	9.13	47.49		1151.17	1151.43	2H+	2300.84	N2H5Hn4	33N-**BI**/epi						
41.48	9.12	9.13	48.58		1151.97	1151.43	2H+	2300.84	N2H5Hn4	33N-**BI**/epi	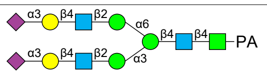	0.890	0.000			
41.48	9.12		48.58		851.12	851.33	2H+	1700.64	H4Hn4dH1	**BI**F(6)-Ga/epi	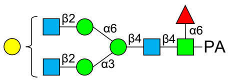	0.169	0.015			
41.48	9.12		48.58		811.30	811.35	H+	810.34	H1Hn2dH1	**M1A**F(6)	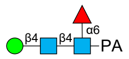	0.164	0.014	✓		
41.48	9.12		48.58		1333.52	1333.99	2H+	2665.97	N2H6Hn5	dN-**TR**						
42.07	9.29		49.21		697.81	697.25	2H+	1392.49	H8	**Glc8**	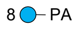	0.124	0.011			
42.07	9.29		49.21		1333.50	1333.99	2H+	2665.97	N2H6Hn5	dN-**TR**	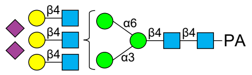	0.225	0.019			
42.07	9.29		49.21		932.55	933.36	2H+	1864.71	H5Hn4dH1	**BI**F(6)/ga	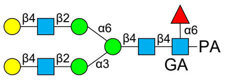					
42.07	9.29		49.21		1260.25							0.082	0.007		✓	

Table S4 continued.

RT (min)	GU experi-mental	GU std.	RP experi-mental	RP std.	*m/z* experi-mental	*m/z* theoretical	ion form	MS	Composition (+PA)	Glycan structure		Quantity ratio % for top	Quantity ratio % for whole	MS²	Only	Note
42.07	9.29	9.56	49.21		1192.31	1192.47	H+	1191.47	H3Hn3	**AG2**	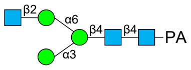	0.077	0.007	✓		
42.07	9.29		49.21		1204.59	1205.44	3H+	3613.30	N4H7Hn6	teN-**TE**/ga	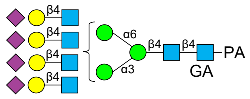	0.075	0.007		✓	
43.15	9.61		50.35		1478.09	1479.54	2H+	2957.07	N3H6Hn5	trN-F-**TR**/ga						
43.15	9.61	9.63	50.35	48.64	924.71	924.85	2H+	1847.69	N1H4Hn4	06N-**BI**-Ga2	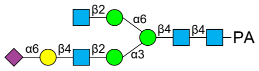	2.049	0.177			
43.15	9.61		50.35		1296.38	1297.49	2H+	2592.96	N2H5Hn4dH2	dN-F-**BI**F(6)/ga						
43.69	9.78		50.92		1296.54	1297.49	2H+	2592.96	N2H5Hn4dH2	3N-N-F(3)-**BI**F(6)/ga	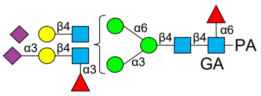			✓		SLe^x
43.69	9.78		50.92		1478.88	1479.54	2H+	2957.07	N3H6Hn5	trN-F-**TR**/ga	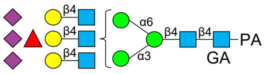					
43.69	9.78		50.92		1026.28	1026.39	2H+	2050.77	N1H4Hn5	N-**BI**Bs-Ga	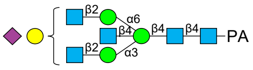					
43.69	9.78		50.92		985.92	986.70	3H+	2957.07	N3H6Hn5	trN-**TR**/ga	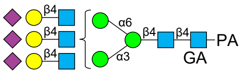					
43.69	9.78	9.63	50.92	48.64	924.71	924.85	2H+	1847.69	N1H4Hn4	06N-**BI**-Ga2						
44.18	9.93	10.05	51.43		860.32	860.33	2H+	1718.65	H5Hn4	**BI**	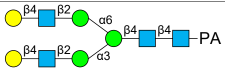	2.366	0.205	✓		
44.18	9.93	10.07	51.43		1224.46	1224.46	2H+	2446.90	N2H5Hn4dH1	N-3N-F(3)-**BI**	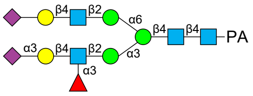	1.254	0.108			SLe^x
44.18	9.93		51.43		1135.29	1135.45	H+	1134.44	H3Hn2dH1	**M3B**F(6)						
44.18	9.93		51.43	50.51	973.41	973.40	H+	972.39	H2Hn2dH1	**M2B**F(6)						
44.86	10.15	10.07	52.15	50.51	973.42	973.40	H+	972.39	H2Hn2dH1	**M2B**F(6)	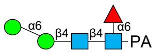	38.161	3.300	✓		
44.86	10.15		52.15		1077.94	1078.91	2H+	2155.80	N1H5Hn4dH1	06N-**BI**F(6)/ga	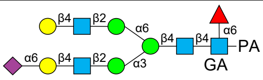					
44.86	10.15		52.15	50.87	1005.94	1005.88	2H+	2009.75	N1H5Hn4	06N-**BI**	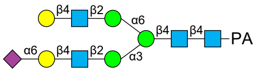	15.548	1.344	✓		
44.86	10.15	10.07	52.15	50.51	1135.44	1135.45	H+	1134.44	H3Hn2dH1	**M3B**F(6)	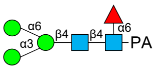	15.012	1.298			
45.95	10.50	10.55	53.25		1223.94	1224.97	2H+	2446.90	N2H5Hn4dH1	66N-**BI**F(6)/ga	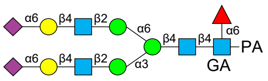			✓		
47.05	10.86		54.3579		1077.80	1078.91	2H+	2155.80	N1H5Hn4dH1	N-**BI**F(6)/ga	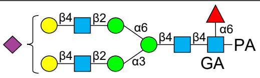			✓		[illegible]
47.05	10.86		54.36		1243.7	1243.46	3H+	3727.36	N4H5Hn6dH3	teN-dF-Ldn-**BI**F(6)	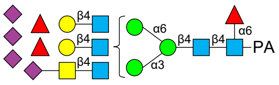	0.376	0.033	✓		N-Ldn,
47.05	10.86	10.91	54.36		1223.23	1224.46	2H+	2446.90	N2H5Hn4dH1	36N-**BI**F(6)/ga				✓		
47.05	10.86		54.36		1119.84	1119.94	2H+	2237.86	N1H3Hn6dH1	N-dLdn-**M3**F(6)	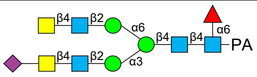	0.240	0.021	✓		N-Ldn
47.58	11.04	10.91	54.89		1223.91	1224.46	2H+	2446.90	N2H5Hn4dH1	36N-**BI**F(6)/ga	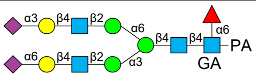			✓		
47.58	11.04	11.15	54.89		1077.85	1078.91	2H+	2155.80	N1H5Hn4dH1	03N-**BI**F(6)/ga	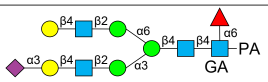					
47.58	11.04	11.73	54.89		1261.06	1261.48	2H+	2520.94	N1H6Hn5dH1	N-**TR**F(6)	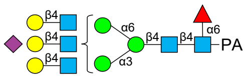	6.523	0.564	✓		

Table S4 continued.

RT (min)	GU experi-mental	GU std.	RP experi-mental	RP std.	*m/z* experi-mental	*m/z* theoretical	*m/z* ion form	MS	Composition (+PA)		Glycan structure	Quantity ratio % for top	Quantity ratio % for whole	MS^2	Only	Note
47.58	11.04		54.89		1180.84	1180.96	2H+	2359.90	H5Hn5dH3	F(2)-F(3)-**BI**BsF(6)	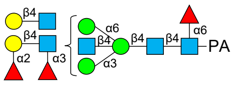	4.215	0.364			Le[y]
47.58	11.04	10.91	54.89		1034.92	1034.90	2H+	2067.79	H5Hn5dH1	F-**BI**Bs	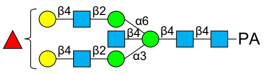	1.184	0.102			
48.15	11.24	11.49	55.47	54.54	1151.75	1151.43	2H+	2300.84	N2H5Hn4	66N-**BI**		0.190	0.016	✓		
48.15	11.24	11.65	55.47		1223.56	1224.46	2H+	2446.90	N2H5Hn4dH1	33N-**BIF**(6)/ga	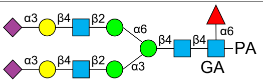			✓		
48.15	11.24		55.47		1180.59	1180.96	2H+	2359.90	H5Hn5dH3	F(2)-F(3)-**BI**BsF(6)	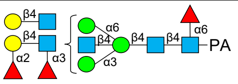	0.106	0.009	-		Le[y]
48.15	11.24		55.47		1077.62	1078.91	2H+	2155.80	N1H5Hn4dH1	66N-**BIF**(6)/ga				✓		
48.15	11.24	11.12	55.47		1034.76	1034.90	2H+	2067.79	H5Hn5dH1	**BI**BsF(6)	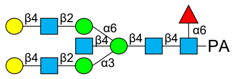	0.044	0.004			
48.15	11.24	11.33	55.47	55.68	916.97	916.86	2H+	1831.70	N1H3Hn4dH1	N-Ldn1-**M3F**(6)	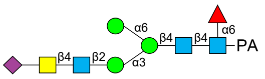	0.029	0.003			N-Ldn
48.68	11.42	11.49	56.00	54.54	1151.56	1151.43	2H+	2300.84	N2H5Hn4	66N-**BI**	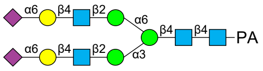	100	8.647	✓		
49.52	11.71	11.65	56.85		1223.85	1224.46	2H+	2446.90	N2H5Hn4dH1	33N-**BIF**(6)/ga	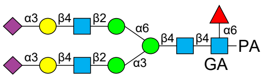			✓		
49.52	11.71	11.98	56.85	55.95	1005.84	1005.88	2H+	2009.75	N1H5Hn4	30N-**BI**	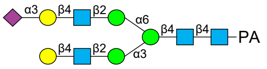	1.644	0.142	✓		
49.52	11.71	12.02	56.85	56.14	1151.85	1151.43	2H+	2300.84	N2H5Hn4	66N-**BI**				✓		
50.26	11.98	12.02	57.59	56.14	1151.32	1151.43	2H+	2300.84	N2H5Hn4	36N-**BI**	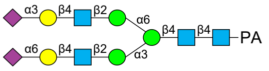	14.234	1.231	✓		
50.26	11.98	11.65	57.59		1223.21	1224.46	2H+	2446.90	N2H5Hn4dH1	33N-**BIF**(6)/ga	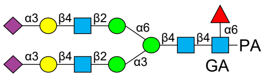			✓		
50.26	11.98	11.98	57.59	55.95	1005.51	1005.88	2H+	2009.75	N1H5Hn4	30N-**BI**	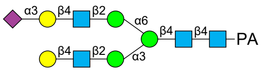	2.225	0.192	✓		
50.26	11.98	12.08	57.59	57.74	1205.30	1205.44	3H+	3613.30	N4H7Hn6	teN-**TE**	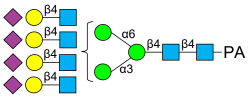	1.247	0.108		✓	
50.26	11.98	12.00	57.59	56.09	771.36	771.31	2H+	1540.60	H3Hn4dH1	**AG12**F(6)	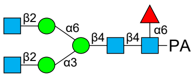	0.639	0.055			
50.26	11.98	11.68	57.59	59.36	1338.36	1338.53	H+	1337.52	H3Hn3dH1	**AG2**F(6)	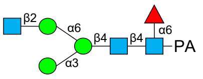	0.471	0.041			
51.87	12.56		59.21		1253.42	1253.48	2H+	2504.94	N1H5Hn5dH2	N-F-Gn-Ln-**M4**F(6)	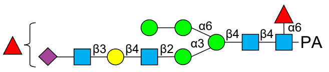	1.581	0.137	✓		
51.87	12.56		59.21		1405.91	1407.02	2H+	2812.03	N2H6Hn4dH1	dN-F-**TR**				✓		
51.87	12.56	12.59	59.21		1188.19	1188.45	2H+	2374.88	N1H6Hn5	N-**TR**	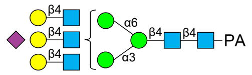	0.208	0.018			
51.87	12.56		59.21		1551.50	1552.57	2H+	3103.13	N3H6Hn5dH1	trN-F-**TR**	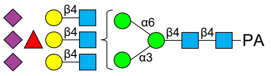	0.084	0.007			
51.87	12.56		59.21		938.14	938.35	3H+	2812.03	N2H6Hn4dH1	dN-**TR**F(6)	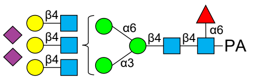	0.020	0.002		✓	
52.21	12.69		59.56		1406.69	1407.02	2H+	2812.03	N2H6Hn4dH1	dN-F-**TR**	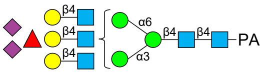	4.374	0.378	✓		N-dLn, Lex
52.21	12.69		59.56		1224.38	1224.46	2H+	2446.90	N2H5Hn4dH1	33N-**BIF**(6)/epi	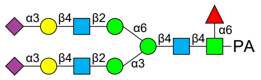	3.584	0.310	✓		

Table S4 continued.

RT (min)	GU experi-mental	GU std.	RP experi-mental	RP std.	*m/z* experi-mental	*m/z* theoretical	*m/z* ion form	MS	Composition (+PA)	Glycan structure	Quantity ratio % for top	Quantity ratio % for whole	MS^2	Only	Note
52.21	12.69	13.11	59.56	60.83	852.12	852.33	2H+	1702.66	H4Hn4dH1	**BI**F(6)-Ga2 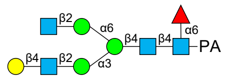	3.075	0.266	✓		
52.21	12.69	13.08	59.56	60.45	1551.83	1552.57	2H+	3103.13	N3H6Hn5dH1	trN-F-**TR**					
52.21	12.69		59.56		1156.37	1157.09	3H+	3468.26	N3H7Hn6dH1	trN-F-**TE** 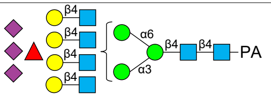	1.210	0.105		✓	
52.82	12.92	13.08	60.17	60.45	1551.82	1552.57	2H+	3103.13	N3H6Hn5dH1	trN-F-**TR** 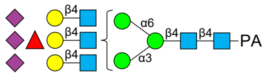	2.797	0.242	-		
52.82	12.92		60.17		1405.86	1407.02	2H+	2812.03	N2H6Hn4dH1	dN-F-**TR**/ga 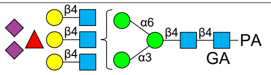					
52.82	12.92		60.17		1034.53	1034.90	2H+	2067.79	H5Hn5dH1	Gn-**BI**F(6) 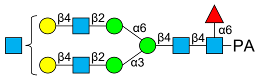	0.328	0.028			
52.82	12.92	13.35	60.17		1151.16	1151.43	2H+	2300.84	N2H5Hn4	63N-**BI** 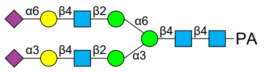	0.238	0.021			
54.54	13.59	13.61	61.91	60.83	933.21	933.36	2H+	1864.71	H5Hn4dH1	**BI**F(6) 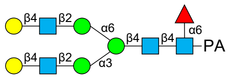	4.996	0.432	✓		
54.54	13.59	13.59	61.91	61.78	1205.68	1205.44	3H+	3613.30	N4H7Hn6	teN-**TE** 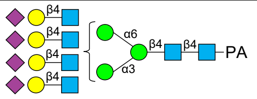	4.641	0.401		✓	
54.54	13.59	13.37	61.91		1297.10	1297.49	2H+	2592.96	N2H5Hn4dH2	dN-F(3)2-**BI**F(6) 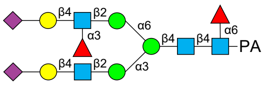	3.117	0.270	✓		SLe^x
54.54	13.59	14.01	61.91		1333.90	1333.99	2H+	2665.97	N2H6Hn5	dN-Ln-**BI** 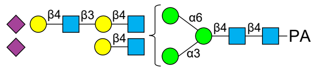	2.931	0.253	-		
54.54	13.59		61.91		1099.39	1099.42	2H+	2196.83	N1H4Hn5dH1	N-F-Ldn-**MO** 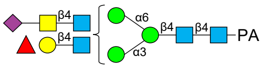	7.152	0.618	✓		N-Ldn
54.54	13.59	13.35	61.91		1151.24	1151.43	2H+	2300.84	N2H5Hn4	63N-**BI** 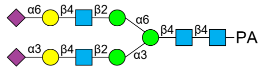	1.662	0.144			
54.54	13.59		61.91		1406.52	1407.02	2H+	2812.03	N2H6Hn4dH1	dN-Ln-**BI**F(6) 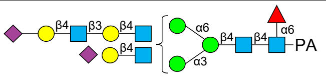	1.326	0.115			N-dLn
54.54	13.59	13.72	61.91		997.96	997.88	2H+	1993.75	N1H4Hn4dH1	06N-**BI**F(6)-Ga2 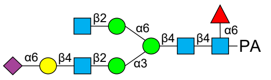	0.899	0.078			
54.97	13.76	14.01	62.34		1333.86	1333.99	2H+	2665.97	N2H6Hn5	dN-Ln-**BI** 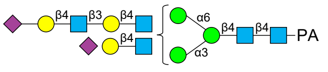	37.937	3.280	✓		N-dLn
54.97	13.76	13.80	62.34		1552.26	1552.57	2H+	3103.13	N3H6Hn5dH1	3N-dN-F(3)-**TR** 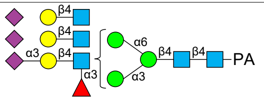	12.538	1.084	✓		SLe^x
54.97	13.76		62.34		1099.50	1099.42	2H+	2196.83	N1H4Hn5dH1	N-Ldn-**MO**F(6)			✓		N-Ldn
54.97	13.76		62.34		1369.85	1370.00	2H+	2737.99	N3H5Hn4dH1	6N-3N-N-**BI**F(6) 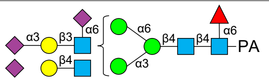	2.518	0.218	✓		dN-Ln
54.97	13.76		62.34		1205.48	1205.44	3H+	3613.30	N4H7Hn6	teN-**TE**					
55.90	14.14	14.12	63.28	62.25	1078.63	1078.91	2H+	2155.80	N1H5Hn4dH1	06N-**BI**F(6) 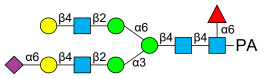	56.730	4.905	✓		
55.90	14.14		63.28		1334.32	1333.99	2H+	2665.97	N2H6Hn5	dN-**TR** 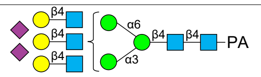	4.235	0.366			
55.90	14.14	14.23	63.28	62.56	1151.27	1151.43	2H+	2300.84	N2H5Hn4	33N-**BI** 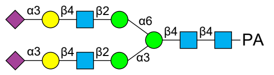	3.492	0.302	✓		
55.90	14.14		63.28		1179.32	1180.45	2H+	2358.88	N1H5Hn5dH1	06N-**BI**BsF(6)/ga 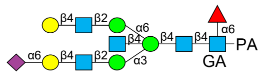	2.268	0.196			

Table S4 continued.

RT (min)	GU experi-mental	GU std.	RP experi-mental	RP std.	*m/z* experi-mental	*m/z* theoretical	*m/z* ion form	MS	Composition (+PA)		Glycan structure	Quantity ratio % for top	Quantity ratio % for whole	MS^2	Only	Note
55.90	14.14		63.28		1033.94	1035.38	3H+	3103.13	N3H6Hn5dH1	trN-**TR**F(6)/ga	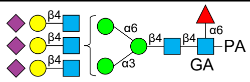					
56.95	14.57	14.99	64.33	65.17	1479.36	1479.54	2H+	2957.07	N3H6Hn5	trN-**TR**	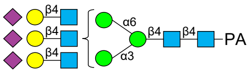	23.657	2.046			
56.95	14.57		64.33		1552.36	1552.57	2H+	3103.13	N3H6Hn5dH1	trN-F-**TR**	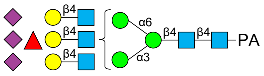	16.054	1.388			
56.95	14.57	14.48	64.33		1026.53	1026.90	2H+	2051.79	H4Hn5dH2	F-**BI**BsF(6)-Ga	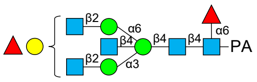	9.230	0.798			
56.95	14.57		64.33		1244.56	1244.97	2H+	2487.93	N2H4Hn5dH1	dN-Ldn-**MO**F(6)	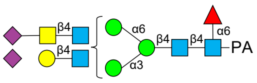	5.703	0.493			N-Ldn
56.95	14.57	14.96	64.33		1107.17	1107.42	2H+	2212.83	N1H5Hn5	06N-**BI**Bs	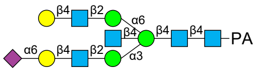	4.351	0.376			
56.95	14.57	14.87	64.33	64.27	1224.36	1224.46	2H+	2446.90	N2H5Hn4dH1	66N-**BI**F(6)						
58.11	15.07	14.87	65.50	64.27	1224.56	1224.46	2H+	2446.90	N2H5Hn4dH1	66N-**BI**F(6)	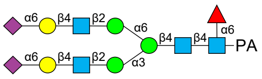	92.033	7.958	✓		
58.11	15.07	14.79	65.50		1119.90	1119.94	2H+	2237.86	N1H3Hn6dH1	06N-dLdn12-**M3**F(6)	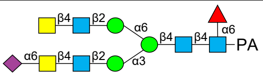	7.261	0.628	✓		N-Ldn
58.11	15.07		65.50		1629.32							4.148	0.359		✓	
58.11	15.07	15.35	65.50		1138.97	1139.40	2H+	2276.79	S1N1H4 Hn5dH1	S-N-Ldn1-**MO2**F(6)	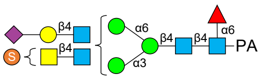	3.930	0.340			S-Ldn
58.11	16.07		65.50		1054.28	1054.34	2H+	2106.67	S2H3Hn6dH1	dS-dLdn-**M3**F(6)		1.023	0.088			
58.86	15.40	15.57	66.26	66.49	1479.44	1479.54	2H+	2957.07	N3H6Hn5	trN-Ln-**BI**	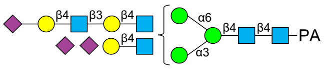	7.885	0.682	✓		N-dLn
58.86	15.40	15.86	66.26	65.53	1078.76	1078.91	2H+	2155.80	N1H5Hn4dH1	60N-**BI**F(6)	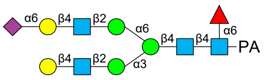	3.832	0.331	✓		
58.86	15.40		66.26		974.12	974.39	2H+	1946.76	H3Hn6dH1	dLdn12-**M3**F(6)	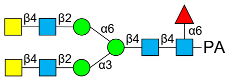	2.128	0.184			Ldn
58.86	15.40	14.87	66.26	64.27	1224.39	1224.46	2H+	2446.90	N2H5Hn4dH1	66N-**BI**F(6)						
58.86	15.40		66.26		1054.07	1054.34	2H+	2106.67	S2H3Hn6dH1	(dS-dLdn)-**M3**F(6)	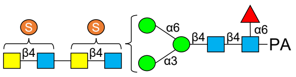	2.202	0.190			S-Ldn
58.86	15.40		66.26		1244.82	1244.97	2H+	2487.93	N2H4Hn5dH1	36N-Ldn2-**MO1**F(6)						
59.66	15.76	15.48	67.06		1245.20	1244.97	2H+	2487.93	N2H4Hn5dH1	36N-Ldn2-**MO1**F(6)	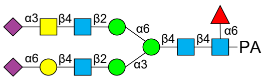	2.567	0.222	✓		N-Ldn
59.66	15.76	15.85	67.06		1156.95	1157.09	3H+	3468.26	N3Hn7H6dH1	trN-**TE**F(6)	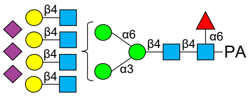	0.495	0.043			
59.66	15.76	16.73	67.06		961.86	961.87	2H+	1921.73	H5Hn5	**BI**Bs	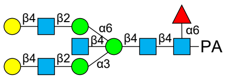	0.037	0.003	✓		
60.98	16.38		68.36		1224.26	1224.46	2H+	2446.90	N2H5Hn4dH1	36N-**BI**F(6)				✓		
60.98	16.38	16.26	68.36		1107.80	1107.93	2H+	2213.85	H5Hn5dH2	F(3)-**BI**BsF(6)	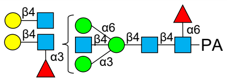	0.290	0.025			Le^x
60.98	16.38		68.36		1261.45	1261.48	2H+	2520.94	N1H6Hn5dH1	N-Ln-**BI**F(6)	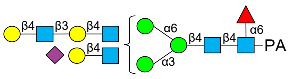	0.268	0.023	✓	✓	dLn
60.98	16.38	16.73	68.36		961.86	961.87	2H+	1921.73	H5Hn5	**BI**Bs	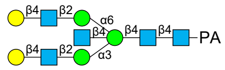	0.178	0.015	✓		
60.98	16.38		68.36		1157.24	1157.09	3H+	3468.26	N3Hn7H6dH1	trN-**TE**F(6)	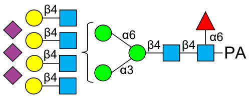	0.142	0.012		✓	

Table S4 continued.

RT (min)	GU		RP		*m/z*			MS	Composition (+PA)	Glycan structure		Quantity ratio		MS^2	Only	Note
	experi-mental	std.	experi-mental	std.	experi-mental	theoretical	ion form					% for top	% for whole			
60.98	16.38		68.36		1552.45	1552.57	2H+	3103.13	N3H6Hn5dH1	trN-F-**TR**	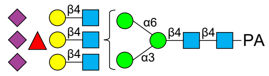	0.122	0.011		✓	
61.48	16.62	16.30	68.84	67.84	1224.43	1224.46	2H+	2446.90	N2H5Hn4dH1	36N-**BI**F(6)	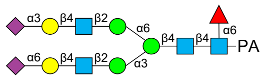	39.709	3.433	✓		
61.48	16.62	17.28	68.84		1078.54	1078.91	2H+	2155.80	N1H5Hn4dH1	03N-**BI**F(6)	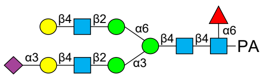	13.644	1.180	✓		
61.48	16.62		68.84		1254.22	1254.13	3H+	3759.35	N4H7Hn6dH1	trN-F-**TE**	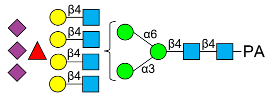	4.059	0.351	✓	✓	
61.48	16.62	16.96	68.84		1479.51	1479.54	2H+	2957.07	N3H6Hn5	trN-**TR**	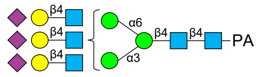	2.556	0.221			
62.23	16.99	17.66	69.56	68.72	1078.82	1078.91	2H+	2155.80	N1H5Hn4dH1	30N-**BI**F(6)	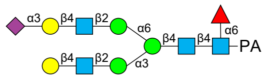	1.961	0.170	✓		
62.23	16.99		69.56		1325.45	1326.00	2H+	2649.98	N2H5Hn5dH1	dN-Gn-**BI**F(6)	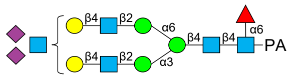	0.680	0.059			
62.23	16.99	17.43	69.56		1224.25	1224.46	2H+	2446.90	N2H5Hn4dH1	36N-**BI**F(6)				✓		
63.38	17.57		70.66		1261.30	1261.48	2H+	2520.94	N1H6Hn5dH1	N-**TR**F(6)	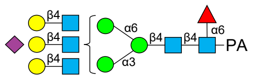	3.316	0.287			
63.38	17.57		70.66		1321.25	1321.82	3H+	3962.43	N4H7Hn7dH1	teN-**TE**BsF(6)	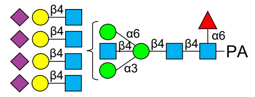	1.983	0.172		✓	
63.38	17.57		70.66		1156.94	1157.43	3H+	3469.28	N2H7Hn6dH3	dN-trF-**TE**	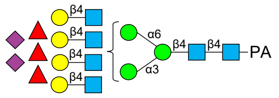	1.621	0.140		✓	
63.38	17.57	17.70	70.66		1735.42	1735.14	2H+	3468.26	N3Hn7H6dH1	trN-**TE**F(6)	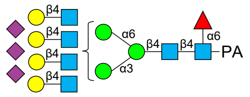	1.070	0.092		✓	
63.38	17.57		70.66		1099.15	1099.42	2H+	2196.83	N1H4Hn5dH1	N-Ldn-**MO**F(6)	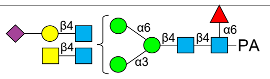	0.853	0.074	✓		Ldn
63.38	17.57	17.43	70.66	70.68	1224.05	1224.46	2H+	2446.90	N2H5Hn4dH1	63N-**BI**F(6)	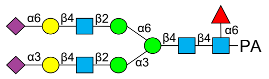	0.435	0.038			
64.89	18.38		72.11		1253.84	1254.13	3H+	3759.35	N4H7Hn6dH1	6N-3N-3N-N-F(3)-Ln-**TR**	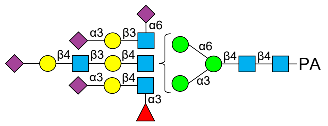	42.161	3.645	✓		N-dLn, dN-Ln, SLe^x
64.89	18.38		72.11		1157.36	1157.43	3H+	3469.28	N2H7Hn6dH3	dN-trF-**TE**	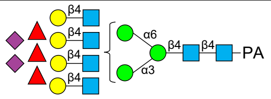	6.957	0.602			
64.89	18.38		72.11		1151.82	1151.94	2H+	2301.86	N1H5Hn4dH2	3N-F(3)-**BI**F(6)	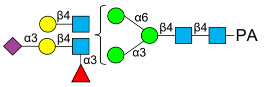	3.980	0.344	✓		SLe^x
66.09	19.06		73.26		1157.35	1157.43	3H+	3469.28	N3Hn7H6dH1	trN-**TE**F(6)	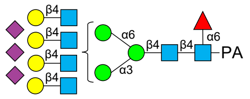	0.867	0.075		✓	
66.09	19.06		73.26		1406.93	1407.02	2H+	2812.03	N2H6Hn4dH1	dN-**TR**F(6)	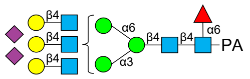	0.703	0.061			
66.09	19.06		73.26		1479.57	1479.54	2H+	2957.07	N3H6Hn5	trN-**TR**	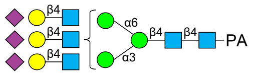	0.578	0.050		✓	
66.09	19.06		73.26		1289.17	1289.99	2H+	2577.96	N1H6Hn6	N-**TR**Bs	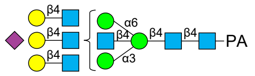	0.210	0.018			

Table S4 continued.

RT (min)	GU		RP		*m/z*			MS	Composition (+PA)		Glycan structure	Quantity ratio		MS^2	Only	Note
	experi-mental	std.	experi-mental	std.	experi-mental	theoretical	ion form					% for top	% for whole			
66.09	19.06		73.26		1099.10	1099.42	2H+	2196.83	N1H4Hn5dH1	N-Ldn-**MOF**(6)	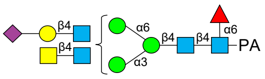	0.075	0.006			Ldn
67.03	19.62	19.43	74.17	73.91	1224.78	1224.46	2H+	2446.90	N2H5Hn4dH1	33N-**BIF**(6)	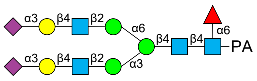	38.470	3.326	✓		
67.03	19.62		74.17		1406.76	1407.02	2H+	2812.03	N2H6Hn5dH1	dN-**TRF**(6)	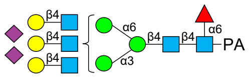	13.460	1.164	✓		
67.03	19.62		74.17		1552.71	1552.57	2H+	3103.13	N3H6Hn5dH1	trN-**TRF**(6)	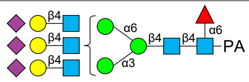	5.054	0.437			
67.03	19.62		74.17		1099.1	1099.42	2H+	2196.83	N1H4Hn5dH1	N-Ldn-**MOF**(6)	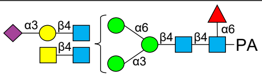	0.657	0.057		✓	Ldn
67.03	19.62		74.17		1216.62							0.270	0.023		✓	
68.46	20.52	21.99	75.54	77.61	872.83	872.85	2H+	1743.68	H3Hn5dH1	**AG12**BsF(6)	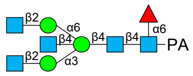	1.865	0.161	✓		
68.46	20.52		75.54		1407.04	1407.02	2H+	2812.03	N2H6Hn5dH1	dN-**TRF**(6)	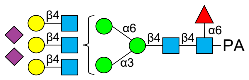	0.816	0.071		✓	
68.46	20.52		75.54		1216.08							0.528	0.046			
69.08	20.93		76.13		1381.14							1.444	0.125		✓	
69.08	20.93		76.13		1406.67	1407.02	2H+	2812.03	N2H6Hn5dH1	dN-**TRF**(6)	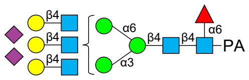	1.370	0.118		✓	
69.08	20.93		76.13		1170.69	1170.77	3H+	3509.29	N3H6Hn7dH1	trN-**TE**BsF(6)-Ga	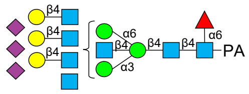	0.565	0.049		✓	
69.95	21.52	20.35	76.97		1552.38	1552.57	2H+	3103.13	N3H6Hn5dH1	trN-**TRF**(6)	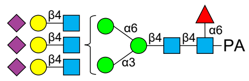	25.760	2.227	✓		
71.41	22.59	21.99	78.37	77.61	1180.35	1180.45	2H+	2358.88	N1H5Hn5dH1	06N-**BI**BsF(6)	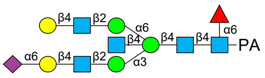	5.536	0.479	✓		
71.41	22.59	22.02	78.37		1552.05	1552.57	2H+	3103.13	N3H6Hn5dH1	trN-F-**TR**	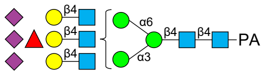	3.254	0.281			
71.41	22.59		78.37		1261.28	1261.48	2H+	2520.94	N1H6Hn5dH1	N-**TRF**(6)	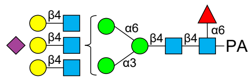	1.406	0.122			
71.41	22.59	22.47	78.37	78.21	953.59	953.87	2H+	1905.73	H4Hn5dH1	**BI**BsF(6)-Ga2	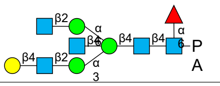	1.385	0.120	✓		
71.41	22.59		78.37		1406.53	1407.02	2H+	2812.03	N2H6Hn5dH1	dN-**TRF**(6)	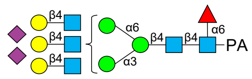	0.808	0.070			
72.09	23.11	23.53	79.02		1325.47	1326.00	2H+	2649.98	N2H5Hn5dH1	66N-**BI**BsF(6)	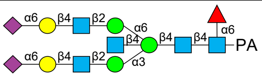	4.427	0.383	✓		
72.09	23.11	23.29	79.02		1406.71	1407.02	2H+	2812.03	N2H6Hn5dH1	dN-**TRF**(6)	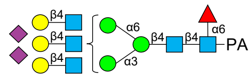	2.552	0.221			dLn
72.09	23.11		79.02		1454.89							1.367	0.118			
72.09	23.11	23.35	79.02	78.21	953.51	953.87	2H+	1905.73	H4Hn5dH1	**BI**BsF(6)-Ga1	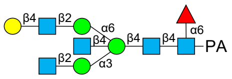	0.733	0.063	✓		
72.09	23.11	22.00	79.02		1132.80	1132.41	3H+	3394.22	N4H6Hn5dH1	teN-**TRF**(6)	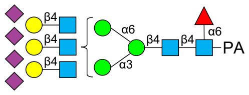	0.342	0.030		✓	
73.46	24.23	23.84	80.34	80.00	1034.55	1034.90	2H+	2067.79	H5Hn5dH1	**BI**BsF(6)	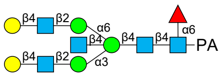	12.226	1.057	✓		
73.46	24.23	23.29	80.34		1406.66	1407.02	2H+	2812.03	N2H6Hn5dH1	dN-**TRF**(6)	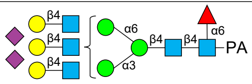	6.237	0.539	✓		

Table S4 continued.

RT (min)	GU		RP		*m/z*			MS	Composition (+PA)		Glycan structure	Quantity ratio		MS2	Only	Note
	experi-mental	std.	experi-mental	std.	experi-mental	theoretical	ion form					% for top	% for whole			
73.46	24.23		80.34		1217.04	1217.47	2H+	2432.92	H6Hn6dH1	**TR**BsF(6)	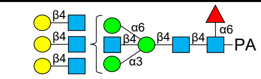	0.759	0.066			
75.40	25.97	25.99	82.20		1325.56	1326.00	2H+	2649.98	N2H5Hn5dH1	36N-**BI**BsF(6)	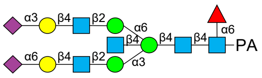	1.131	0.098			
75.40	25.97		82.20		1406.60	1407.02	2H+	2812.03	N2H6Hn4dH1	dN-**TRF**(6)	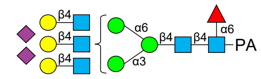	0.941	0.081			
75.40	25.97		82.20		1099.28	1099.42	2H+	2196.83	N1H4Hn5dH1	N-**BI**BsF(6)-Ga	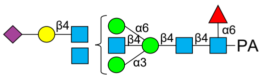	0.292	0.025			
75.54	26.11		82.33		1552.71	1552.57	2H+	3103.13	N3H6Hn5dH1	trN-**TRF**(6)	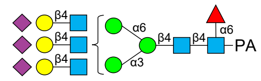	10.835	0.937	✓		
75.54	26.11	24.25	82.33		1407.25	1407.02	2H+	2812.03	N2H6Hn5dH1	dN-**TRF**(6)	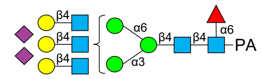	1.979	0.171			
75.54	26.11	26.99	82.33		1144.34	1144.44	2H+	2286.86	H6Hn6	**TR**Bs	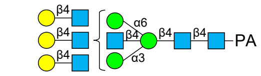	0.831	0.072	✓		
75.54	26.11		82.33		1326.30	1326.00	2H+	2649.98	N2H5Hn5dH1	dN-**BI**BsF(6)	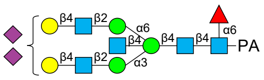	0.281	0.024			
75.54	26.11		82.33		1099.00	1099.42	2H+	2196.83	N1H4Hn5dH1	N-**BI**BsF(6)-Ga	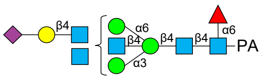	0.130	0.011		✓	
76.79	27.35	27.33	83.53	83.11	1180.41	1180.45	2H+	2358.88	N1H5Hn5dH1	30N-**BI**BsF(6)	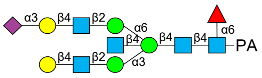	1.024	0.089	✓		
77.45	28.05	27.52	84.16		1132.56	1132.41	3H+	3394.22	N4H6Hn5dH1	teN-**TRF**(6)	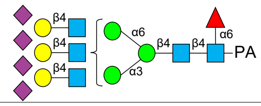	0.691	0.060			
77.45	28.05		84.16		1552.71	1552.57	2H+	3103.13	N3H6Hn5dH1	trN-Ln-**BI**F(6)	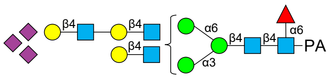	0.215	0.019			
77.45	28.05		84.16		1107.63	1107.93	2H+	2213.85	H5Hn5dH2	F-**BI**BsF(6)	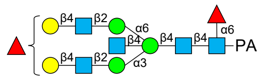	0.121	0.010			
78.04	28.70		84.73		1099.06	1099.42	2H+	2196.83	N1H4Hn5dH1	N-**BI**BsF(6)-Ga	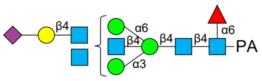	0.277	0.024			
79.16	29.99		85.80		1180.58	1180.96	2H+	2359.90	H5Hn5dH3	dF-**BI**BsF(6)	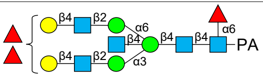	0.687	0.059			
79.16	29.99		85.80		1362.99	1363.01	2H+	2724.02	N1H6Hn6dH1	N-**TR**BsF(6)	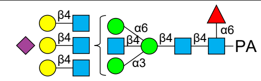	0.217	0.019			
80.16	31.23		86.76		1325.66	1326.00	2H+	2649.98	N2H5Hn5dH1	63N-**BI**BsF(6)	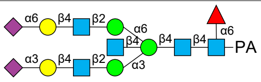	2.918	0.252	✓		
81.19	32.59		87.75		1180.68	1180.96	2H+	2359.90	H5Hn5dH3	dF-**BI**BsF(6)	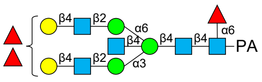	10.262	0.887		✓	
82.68	34.72	34.05	89.18		1290.14	1290.50	2H+	2578.98	H6Hn6dH2	F(3)-**TR**BsF(6)	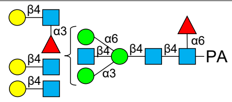	1.740	0.150	-		Le^x
84.37	37.40		90.80		1217.37	1217.47	2H+	2432.92	H6Hn6dH1	**TR**BsF(6)	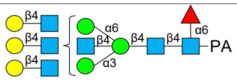	2.149	0.186	-		
87.15	42.50		93.47		1435.51	1435.53	2H+	2870.07	N1H6Hn6dH2	N-F-**TR**BsF(6)	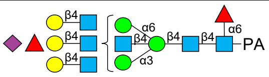	1.265	0.109			
87.15	42.50		93.47		1230.54	1229.79	3H+	3686.34	N4H6Hn5dH3	teN-dF-**TRF**(6)	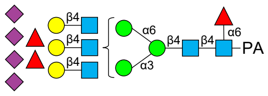	0.198	0.017		✓	

Table S5. *N*-glycans from human urine M2 analyzed using the improved method.

RT (min)	GU experi-mental	GU std.	RP experi-mental	RP std.	m/z experi-mental	m/z theoretical	m/z ion form	MS	Composition (+PA)		Glycan structure	Quantity ratio % for top	Quantity ratio % for whole	MS²	Only	Note
23.69	5.18	5.11	30.67	29.07	899.72	900.33	2H+	1798.65	H8Hn2	**M8A**	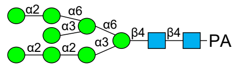	2.953	0.462	✓		
23.69	5.18	5.14	30.67		1049.53	1049.89	2H+	2097.76	N2H5Hn4	66N-**BI'**	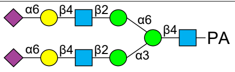	0.988	0.155		✓	
23.69	5.18		30.67		1186.37		2H+		N2H2Hn2+1059			0.443	0.069			
24.81	5.36	5.31	31.75	30.36	818.71	819.31	2H+	1636.60	H7Hn2	**M7A**	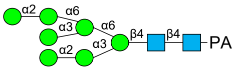	2.762	0.432	✓		
25.29	5.44		32.22		1069.32	1069.39	H+	1068.39	H6	**Glc6**	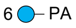	1.714	0.268	✓	✓	
25.29	5.44		32.22		1041.22	1041.89	2H+	2081.77	N2H4Hn3dH1	dN-F-Ln-**M2'**	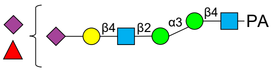	0.239	0.037			
25.29	5.44		32.22		818.79	819.31	2H+	1636.60	H7Hn2	**M7A**						
26.02	5.56	5.50	32.92	31.33	980.83	981.36	2H+	1960.70	H9Hn2	**M9A**	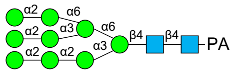	1.319	0.206	✓		
26.02	5.56		32.92		1186.02		2H+					0.313	0.049			
26.84	5.70		33.71		1231.35	1231.45	H+	1230.44	H7	**Glc7**	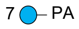	4.132	0.646	✓		
27.44	5.81	5.76	34.29	32.76	899.39	900.33	2H+	1798.65	H8Hn2	**M8B**	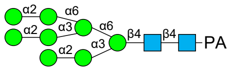	0.322	0.050	✓		
28.08	5.93	5.88	34.90	33.44	818.97	819.31	2H+	1636.60	H7Hn2	**M7B**	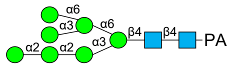	3.736	0.584	✓		
28.08	5.93		34.90		1231.42	1231.45	H+	1230.44	H7	**Glc7**	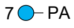	2.911	0.455	✓		
28.08	5.93		34.90		1106.51	1107.42	2H+	2212.83	N1H5Hn5	N-**BI**Bs/ga	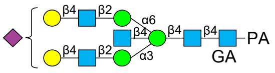					
29.32	6.16	6.11	36.10	34.37	737.68	738.28	2H+	1474.54	H6Hn2	**M6B**	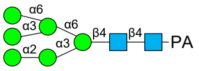	13.093	2.048	✓		
30.36	6.37		37.10		1393.36	1393.50	H+	1392.49	H8	**Glc8**	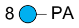	0.288	0.045	✓	✓	
30.99	6.50	6.48	37.71		899.90	900.33	2H+	1798.65	H8Hn2	**M8C**	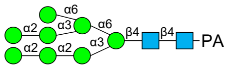	0.436	0.068			
31.84	6.67	6.72	38.52		1004.70	1005.88	2H+	2009.75	N1H5Hn4	06N-**BI**/ga	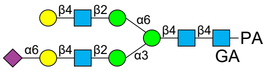					
32.71	6.86		39.36		1004.56	1005.88	2H+	2009.75	N1H5Hn4	06N-**BI**/epi	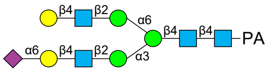			✓		
32.71	6.86	6.80	39.36	37.73	818.72	819.31	2H+	1636.60	H7Hn2	**M7D**	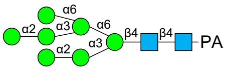	0.406	0.064			
32.71	6.86		39.36		1135.34	1135.45	H+	1134.44	H3Hn2dH1	**M3B**F(6)/epi	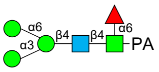			✓		
32.71	6.86	7.08	39.36	38.72	989.31	989.39	H+	988.39	H3Hn2	**M3B**				✓		
33.34	7.00	7.00	39.99	38.72	1313.32	1313.50	H+	1312.49	H5Hn2	**M5A**	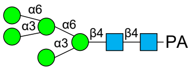	17.833	2.790	✓		
33.34	7.00	7.08	39.99	38.72	989.30	989.39	H+	988.39	H3Hn2	**M3B**	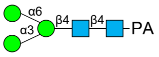	12.538	1.962	✓		
33.34	7.00	7.04	39.99	38.72	827.28	827.34	H+	826.33	H2Hn2	**M2B**	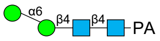	7.424	1.162	✓		
33.34	7.00		39.99		1133.25	1135.45	H+	1134.44	H3Hn2dH1	**M3B**F(6)/ga	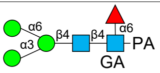			✓		
33.34	7.00	6.72	39.99		1151.29	1151.45	H+	1150.44	H4Hn2	**M4A**	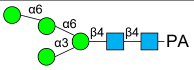	1.581	0.247	✓		

Table S5 continued.

RT (min)	GU experimental	GU std.	RP experimental	RP std.	*m/z* experimental	*m/z* theoretical	*m/z* ion form	MS	Composition (+PA)	Glycan structure		Quantity ratio % for top	Quantity ratio % for whole	MS^2	Only	Note
34.04	7.16	7.06	40.73		1151.31	1151.45	H+	1150.44	H4Hn2	**M4B**	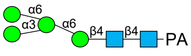	0.440	0.069	✓		
34.04	7.16		40.73		1004.84	1005.88	2H+	2009.75	N1H5Hn4	30N-**BI**/ga	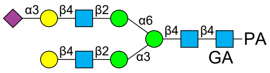					
34.04	7.16		40.73		899.75	900.33	2H+	1798.65	H8Hn2	**M8**	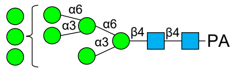	0.100	0.016		✓	
34.04	7.16		40.73		1313.39	1313.50	H+	1312.49	H5Hn2	**M5A**						
34.04	7.16	7.00	40.73		1133.16	1135.45	H+	1134.44	H3Hn2dH1	**M3B**F(6)/ga						
35.25	7.45		42.00		859.14							0.535	0.084	-		
35.25	7.45		42.00		1004.55	1005.88	2H+	2009.75	N1H5Hn4	30N-**BI**/ga				✓		
35.25	7.45		42.00		1150.91	1151.43	2H+	2300.84	N2H5Hn4	66N-**BI**/ga						
35.85	7.60	7.56	42.64		1150.40	1151.43	2H+	2300.84	N2H5Hn4	66N-**BI**/ga	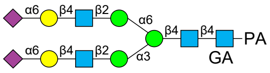			✓		
37.05	7.90	7.88	43.91		1151.24	1151.43	2H+	2300.84	N2H5Hn4	66N-**BI**/epi	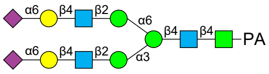	3.821	0.598	✓		
37.64	8.05		44.53		1151.31	1151.43	2H+	2300.84	N2H5Hn4	36N-**BI**/epi	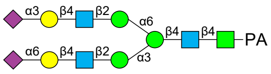	5.873	0.919	✓		
37.64	8.05	8.03	44.53		985.09	985.37	2H+	1968.72	N1H6Hn3	6N-Ln-**M5A**	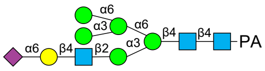	3.738	0.585	✓		
37.64	8.05	8.09	44.53	42.84	823.18	823.31	2H+	1644.61	N1H4Hn3	6N-**MO1**	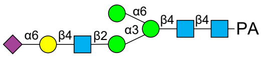	3.702	0.579	✓		
37.64	8.05		44.53		1179.11	1179.41	2H+	2356.80	P1 N1H7Hn3dH1	6P-N-Ln-**M6F**(6)	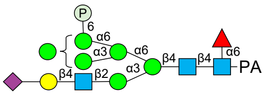	0.767	0.120			
38.20	8.20	8.30	45.12		1150.95	1151.43	2H+	2300.84	N2H5Hn4	36N-**BI**/ga	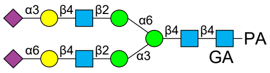			✓		
38.20	8.20	8.25	45.12	43.68	904.19	904.34	2H+	1806.67	N1H5Hn3	6N-Ln-**M4C**	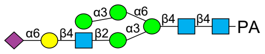	0.791	0.124	✓		
38.20	8.20		45.12		1078.78	1078.91	2H+	2155.80	N1H5Hn4dH1	3N-F(3)-**BI**	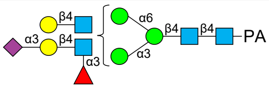	0.259	0.041			SLe^x
39.44	8.54		46.43		1179.86	1179.41	2H+	2356.80	P1 N1H7Hn3dH1	6P-N-Ln-**M6F**(6)	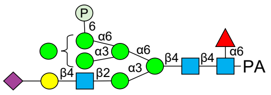	0.244	0.038			
39.44	8.54		46.43		879.51							0.176	0.028			
39.44	8.54		46.43		1150.39	1151.43	2H+	2300.84	N2H5Hn4	dN-**BI**/ga	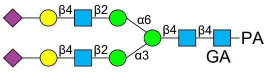					
39.44	8.54	8.67	46.43		770.64	771.31	2H+	1540.60	H3Hn4dH1	**AG12**F(6)/ga	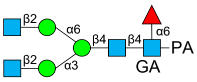					
39.74	8.62		46.75		1179.54	1179.41	2H+	2356.80	P1 N1H7Hn3dH1	6P-N-Ln-**M6F**(6)	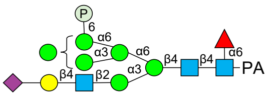	0.784	0.123			
39.74	8.62		46.75		1150.85	1151.43	2H+	2300.84	N2H5Hn4	dN-**BI**/ga	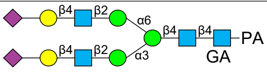					
39.74	8.62	8.64	46.75		1079.38	1079.42	2H+	2156.82	H5Hn4dH3	F(2)-F(3)-**BI**F(6)	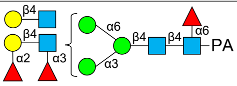	0.564	0.088			Le^y
40.57	8.85		47.62		851.26	851.33	2H+	1700.64	H4Hn4dH1	**BI**F(6)-Ga/epi	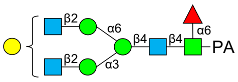	0.254	0.040		✓	
40.57	8.85		47.62		1332.88	1333.99	2H+	2665.97	N2H6Hn5	dN-**TR**/ga	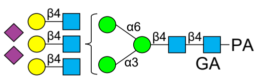					

Table S5 continued.

RT (min)	GU experi-mental	GU std.	RP experi-mental	RP std.	*m/z* experi-mental	*m/z* theoretical	*m/z* ion form	MS	Composition (+PA)	Glycan structure		Quantity ratio % for top	Quantity ratio % for whole	MS^2	Only	Note
40.57	8.85	9.13	47.62		1150.62	1151.43	2H+	2300.84	N2H5Hn4	33N-**BI**/epi						
41.57	9.14		48.68		1551.38	1552.57	2H+	3103.13	N3H6Hn5dH1	trN-F-**TR**	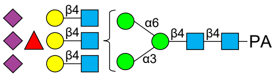	0.138	0.022		✓	
41.57	9.14		48.68		851.05	851.33	2H+	1700.64	H4Hn4dH1	**BI**F(6)-Ga/epi		0.128	0.020			
41.57	9.14	9.09	48.68		985.05	985.37	2H+	1968.72	N1H6Hn3	N-Ln-**M5A**	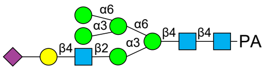	0.087	0.014		✓	
41.57	9.14	9.13	48.68		1151.32	1151.43	2H+	2300.84	N2H5Hn4	33N-**BI**/epi	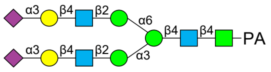	0.076	0.012			
41.57	9.14		48.68		811.26	811.35	H+	810.34	H1Hn2dH1	**M1A**F(6)	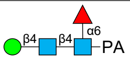	0.028	0.004			
42.04	9.28		49.17		697.81	697.25	2H+	1392.49	H8	**Glc8**	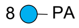	3.279	0.513	-		
42.04	9.28	9.56	49.17		1192.31	1192.47	H+	1191.47	H3Hn3	**AG2**	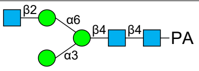	1.309	0.205	✓		
42.04	9.28		49.17		1551.12	1552.57	2H+	3103.13	N3H6Hn5dH1	trN-F-**TR**/ga	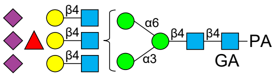				✓	
42.04	9.28		49.17		932.55	933.36	2H+	1864.71	H5Hn4dH1	**BI**F(6)/ga	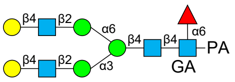					
42.04	9.28		49.17		1333.50	1333.99	2H+	2665.97	N2H6Hn5	dN-**TR**	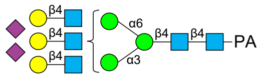	0.425	0.066			
42.99	9.57	9.63	50.18	48.64	924.71	924.85	2H+	1847.69	N1H4Hn4	06N-**BI**-Ga2	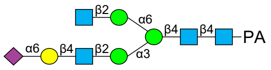	0.397	0.062	✓		
42.99	9.57		50.18		1478.09	1479.54	2H+	2957.07	N3H6Hn5	trN-F-**TR**/ga						
42.99	9.57		50.18		1297.06	1297.49	2H+	2592.96	N2H5Hn4dH2	dN-F-**BI**F(6)/ga						
43.40	9.69		50.61		1478.53	1479.54	2H+	2957.07	N3H6Hn5	trN-F-**TR**/ga	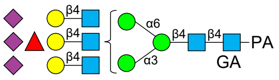					
43.40	9.69		50.61		1026.08	1026.39	2H+	2050.77	N1H4Hn5	N-**BI**Bs-Ga	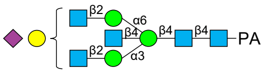	0.665	0.104			
43.40	9.69		50.61		985.96	986.70	3H+	2957.07	N3H6Hn5	trN-**TR**/ga	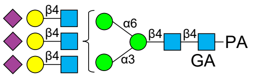					
43.40	9.69		50.61		1296.57	1297.49	2H+	2592.96	N2H5Hn4dH2	N-3N-F(3)-**BI**F(6)/ga	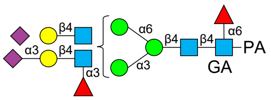					SLe^x
43.40	9.69	9.63	50.61	48.64	924.54	924.85	2H+	1847.69	N1H4Hn4	06N-**BI**-Ga2		0.169	0.027			
44.28	9.96	10.07	51.54		1224.24	1224.46	2H+	2446.90	N2H5Hn4dH1	N-3N-F(3)-**BI**	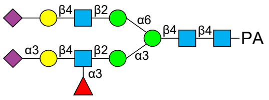	2.329	0.364	✓		SLe^x
44.28	9.96	10.05	51.54		860.19	860.33	2H+	1718.65	H5Hn4	**BI**	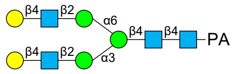	1.680	0.263	✓		
44.28	9.96	10.07	51.54	50.51	973.33	973.40	H+	972.39	H2Hn2dH1	**M2B**F(6)						
44.92	10.17		52.21	50.87	1005.87	1005.88	2H+	2009.75	N1H5Hn4	06N-**BI**	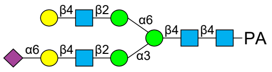	17.620	2.757	✓		
44.92	10.17	10.07	52.21	50.51	1135.33	1135.45	H+	1134.44	H3Hn2dH1	**M3B**F(6)	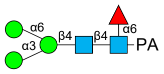	13.063	2.044	✓		
44.92	10.17		52.21		1077.95	1078.91	2H+	2155.80	N1H5Hn4dH1	06N-**BI**F(6)/ga	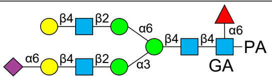			✓		
44.92	10.17	10.07	52.21	50.51	973.42	973.40	H+	972.39	H2Hn2dH1	**M2B**F(6)	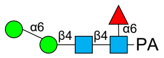	8.431	1.319	✓		

Table S5 continued.

RT (min)	GU		RP		*m/z*			MS	Composition (+PA)	Glycan structure		Quantity ratio		MS^2	Only	Note
	experi-mental	std.	experi-mental	std.	experi-mental	theoretical	ion form					% for top	% for whole			
46.09	10.54	10.55	53.39		1223.41	1224.97	2H+	2446.90	N2H5Hn4dH1	66N-**BI**F(6)/ga	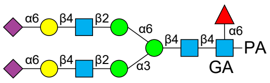					
44.92	10.17		52.21		1118.92	1118.89	2H+	2235.76	S1N1H5Hn4dH	S-N-**BI**F(6)					✓	
46.09	10.54	10.55	53.39		1223.41	1224.97	2H+	2446.90	N2H5Hn4dH1	66N-**BI**F(6)/ga				✓		
46.09	10.54		53.39		1118.92	1118.89	2H+	2235.76	S1N1H5Hn4d H1	S-N-**BI**F(6)	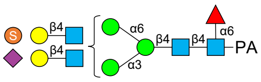	3.285	0.514	✓	✓	N-Ldn
46.09	10.54		53.39		1333.69	1333.99	2H+	2665.97	N2H6Hn5	dN-**TR**	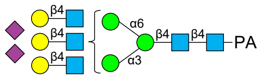	1.827	0.286			
47.01	10.85		54.32		1243.88	1243.46	3H+	3727.36	N4H5Hn6dH3	teN-dF-Ldn-**BI**F(6)	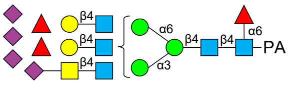	0.154	0.024	✓		N-Ldn
47.01	10.85		54.32		1078.29	1078.91	2H+	2155.80	N1H5Hn4dH1	N-**BI**F(6)/ga	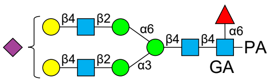			✓		
47.01	10.85		54.32		1119.84	1119.94	2H+	2237.86	N1H3Hn6dH1	N-dLdn-**M3**F(6)	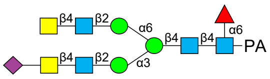	0.081	0.013	✓		N-Ldn
47.01	10.85	10.91	54.32		1223.13	1224.46	2H+	2446.90	N2H5Hn4dH1	36N-**BI**F(6)/ga						
47.61	11.05	10.91	54.92		1223.91	1224.46	2H+	2446.90	N2H5Hn4dH1	36N-**BI**F(6)/ga	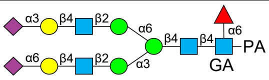			✓		
47.61	11.05	11.15	54.92		1077.85	1078.91	2H+	2155.80	N1H5Hn4dH1	03N-**BI**F(6)/ga	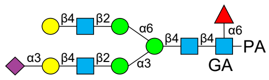			✓		
47.61	11.05		54.92		1180.84	1180.96	2H+	2359.90	H5Hn5dH3	F(2)-F(3)-**BI**BsF(6)	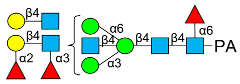	2.604	0.407			Le^y
47.61	11.05	11.73	54.92		1261.06	1261.48	2H+	2520.94	N1H6Hn5dH1	N-**TR**F(6)	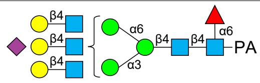	1.721	0.269			
47.61	11.05	10.91	54.92		1034.92	1034.90	2H+	2067.79	H5Hn5dH1	F-**BI**Bs	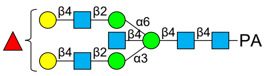	0.883	0.138			
48.02	11.19	11.49	55.34	54.54	1151.44	1151.43	2H+	2300.84	N2H5Hn4	66N-**BI**				✓		
48.02	11.19		55.34		1180.50	1180.96	2H+	2359.90	H5Hn5dH3	F(2)-F(3)-**BI**BsF(6)	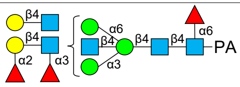	0.129	0.020	✓		Le^y
48.02	11.19	11.12	55.34		1034.64	1034.90	2H+	2067.79	H5Hn5dH1	**BI**BsF(6)	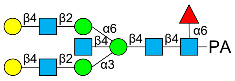	0.071	0.011			
48.02	11.19	11.33	55.34	55.68	916.81	916.86	2H+	1831.70	N1H3Hn4dH1	N-Ldn1-**M3**F(6)	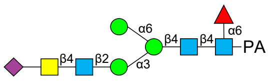	0.059	0.009			N-Ldn
48.02	11.19	11.65	55.34		1223.87	1224.46	2H+	2446.90	N2H5Hn4dH1	33N-**BI**F(6)/ga	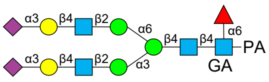					
48.02	11.19	11.06	55.34		1077.58	1078.91	2H+	2155.80	N1H5Hn4dH1	66N-**BI**F(6)/ga						
48.67	11.41	11.49	55.99	54.54	1151.33	1151.43	2H+	2300.84	N2H5Hn4	66N-**BI**	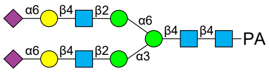	100	15.575	✓		
49.52	11.71	11.65	56.85		1223.20	1224.46	2H+	2446.90	N2H5Hn4dH1	33N-**BI**F(6)/ga	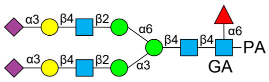			✓		
49.52	11.71	11.98	56.85	55.95	1005.86	1005.88	2H+	2009.75	N1H5Hn4	30N-**BI**	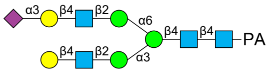	0.808	0.126	✓		
49.52	11.71	12.02	56.85	56.14	1151.42	1151.43	2H+	2300.84	N2H5Hn4	66N-**BI**				✓		
50.21	11.96	12.02	57.54	56.14	1151.07	1151.43	2H+	2300.84	N2H5Hn4	36N-**BI**	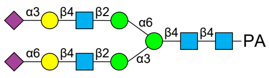	10.353	1.620	✓		
50.21	11.96	11.65	57.54		1223.22	1224.46	2H+	2446.90	N2H5Hn4dH1	33N-**BI**F(6)/ga				✓		

Table S5 continued.

RT (min)	GU		RP		*m/z*			MS	Composition (+PA)	Glycan structure		Quantity ratio		MS²	Only	Note
	experi-mental	std.	experi-mental	std.	experi-mental	theoretical	ion form					% for top	% for whole			
50.21	11.96	11.98	57.54	55.95	1005.48	1005.88	2H+	2009.75	N1H5Hn4	30N-**BI**	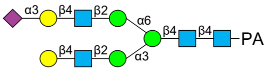	1.740	0.272	✓		
50.21	11.96	12.00	57.54	56.09	771.75	771.31	2H+	1540.60	H3Hn4dH1	**AG12**F(6)	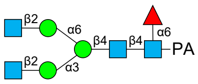	1.092	0.171	✓		
50.21	11.96	11.68	57.54	59.36	1338.32	1338.53	H+	1337.52	H3Hn3dH1	**AG2**F(6)	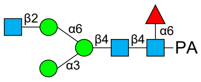	0.513	0.080			
51.90	12.58		59.25		1253.13	1253.48	2H+	2504.94	N1H5Hn5dH2	N-F-Gn-Ln-**M4**F(6)	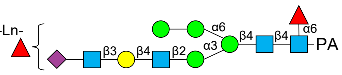	0.557	0.087			
51.90	12.58		59.25		1406.10	1407.02	2H+	2812.03	N2H6Hn4dH1	dN-F-**TR**						
51.90	12.58		59.25		851.97	852.33	2H+	1702.66	H4Hn4dH1	**BI**F(6)-Ga2					✓	
51.90	12.58		59.25		1551.86	1552.57	2H+	3103.13	N3H6Hn5dH1	trN-F-**TR**/ga	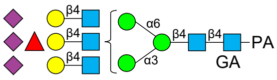					
51.90	12.58		59.25		1188.33	1188.45	2H+	2374.88	N1H6Hn5	N-**TR**	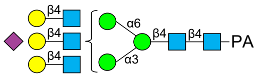	0.172	0.027			
52.21	12.69	13.11	59.56	60.83	852.29	852.33	2H+	1702.66	H4Hn4dH1	**BI**F(6)-Ga2	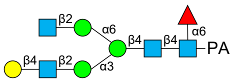	5.561	0.870	✓		
52.21	12.69		59.56		1224.04	1224.46	2H+	2446.90	N2H5Hn4dH1	33N-**BI**F(6)/epi	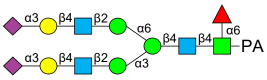	1.534	0.240			
52.21	12.69		59.56		1406.98	1407.02	2H+	2812.03	N2H6Hn4dH1	dN-F-**TR**	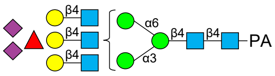	1.325	0.207			
52.21	12.69	13.08	59.56	60.45	1551.82	1552.57	2H+	3103.13	N3H6Hn5dH1	trN-F-**TR**						
52.82	12.92	13.08	60.17	60.45	1551.82	1552.57	2H+	3103.13	N3H6Hn5dH1	trN-F-**TR**	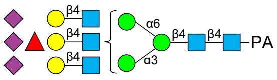	1.189	0.186			
52.82	12.92		60.17		1018.16	1018.40	2H+	2034.78	N1H3Hn5dH1	N-F-Gn-Ldn-**M3**	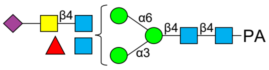	0.358	0.056		✓	N−Ldn
52.82	12.92		60.17		1405.86	1407.02	2H+	2812.03	N2H6Hn4dH1	dN-F-**TR**/ga	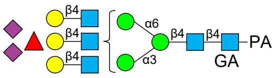					
52.82	12.92		60.17		1034.53	1034.90	2H+	2067.79	H5Hn5dH1	Gn-**BI**F(6)	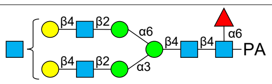	0.278	0.044			
52.82	12.92	13.35	60.17		1151.16	1151.43	2H+	2300.84	N2H5Hn4	63N-**BI**	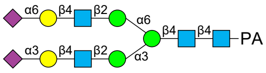	0.218	0.034			
52.82	12.92		60.17		852.27	852.33	2H+	1702.66	H4Hn4dH1	**BI**F(6)-Ga2		0.159	0.025			
54.34	13.51	13.61	61.70	60.83	933.11	933.36	2H+	1864.71	H5Hn4dH1	**BI**F(6)	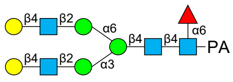	5.748	0.899	✓		
54.34	13.51		61.70		1099.59	1099.42	2H+	2196.83	N1H4Hn5dH1	N-Ldn-**MO**F(6)	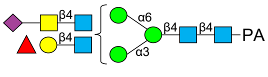	5.102	0.798	✓		N-Ldn
54.34	13.51		61.70		1552.76	1552.57	2H+	3103.13	N3H6Hn5dH1	trN-F(3)-**TR**				✓		SLex
54.34	13.51	14.01	61.70		1334.40	1333.99	2H+	2665.97	N2H6Hn5	dN-**TR**	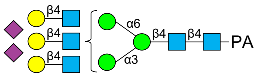	1.048	0.164			
54.34	13.51		61.70		1406.41	1407.02	2H+	2812.03	N2H6Hn4dH1	dN-Ln-**BI**F(6)	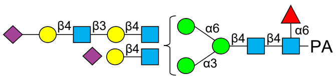	0.912	0.143			N-dLn
54.34	13.51	13.72	61.70		997.81	997.88	2H+	1993.75	N1H4Hn4dH1	06N-**BI**F(6)-Ga2	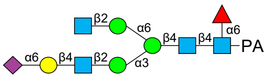	0.791	0.124			
54.34	13.51	13.35	61.70		1151.02	1151.43	2H+	2300.84	N2H5Hn4	63N-**BI**	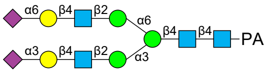	0.435	0.068			

Table S5 continued.

RT (min)	GU experi-mental	GU std.	RP experi-mental	RP std.	*m/z* experi-mental	*m/z* theoretical	*m/z* ion form	MS	Composition (+PA)	Glycan structure	Quantity ratio % for top	Quantity ratio % for whole	MS^2	Only	Note
54.34	13.51	13.37	61.70		1296.97	1297.49	2H+	2592.96	N2H5Hn4dH2	dN-F(3)2-**BI**F(6) 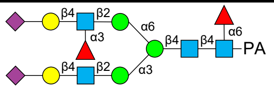	0.361	0.056			SLe^x
55.09	13.81	13.80	62.46		1552.3	1552.57	2H+	3103.13	N3H6Hn5dH1	3N-dN-F(3)-**TR** 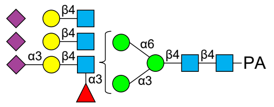	17.297	2.706	-		SLe^x
55.09	13.81		62.46		1333.92	1333.99	2H+	2665.97	N2H6Hn5	dN-Ln-**BI** 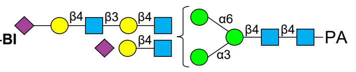	3.244	0.507			N-dLn
55.09	13.81	14.08	62.46		1324.56	1326.00	2H+	2649.98	N2H5Hn5dH1	66N-**BI**BsF(6)/ga 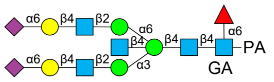					
55.09	13.81		62.46		1369.61	1370.00	2H+	2737.99	N3H5Hn4dH1	6N-3N-N-**BI**F(6) 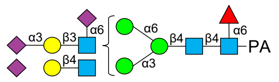	2.681	0.419			dN-Ln
55.09	13.81	14.12	62.46	62.25	1078.83	1078.91	2H+	2155.80	N1H5Hn4dH1	06N-**BI**F(6)			✓		
55.09	13.81		62.46		1099.35	1099.42	2H+	2196.83	N1H4Hn5dH1	N-Ldn-**MO**F(6)					
55.82	14.11	14.12	63.20	62.25	1078.87	1078.91	2H+	2155.80	N1H5Hn4dH1	06N-**BI**F(6) 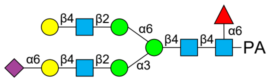	32.384	5.067	✓		
55.82	14.11	14.12	63.20		1552.42	1552.57	2H+	3103.13	N3H6Hn5dH1	3N-dN-F(3)-**TR** 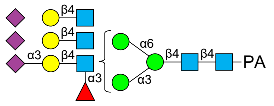	3.327	0.521	-		SLe^x
55.82	14.11	14.23	63.20	62.56	1151.54	1151.43	2H+	2300.84	N2H5Hn4	33N-**BI** 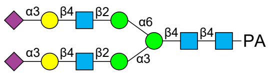	1.799	0.282	✓		
55.82	14.11		63.20		1179.44	1180.45	2H+	2358.88	N1H5Hn5dH1	06N-**BI**BsF(6)/ga 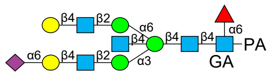					
55.82	14.11	14.01	63.20		1333.82	1333.99	2H+	2665.97	N2H6Hn5	dN-Ln-**BI** 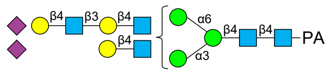	0.940	0.147			dLn
56.96	14.58	14.99	64.34	65.17	1479.86	1479.54	2H+	2957.07	N3H6Hn5	trN-**TR** 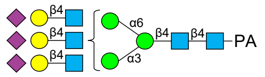	29.488	4.613	✓		
56.96	14.58	14.96	64.34		1107.08	1107.42	2H+	2212.83	N1H5Hn5	06N-**BI**Bs 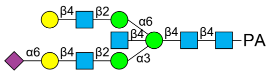	6.337	0.991	✓		
56.96	14.58		64.34		1552.35	1552.57	2H+	3103.13	N3H6Hn5dH1	trN-F-**TR** 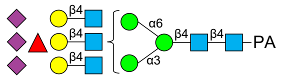	5.400	0.845			
56.96	14.58		64.34		1232.63	1232.96	2H+	2463.91	N1H6Hn4dH2	N-Ga-dF-**BI** 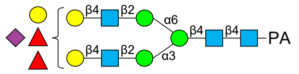	5.385	0.843	✓		
56.96	14.58		64.34		1244.47	1244.97	2H+	2487.93	N2H4Hn5dH1	dN-Ldn-**MO**F(6) 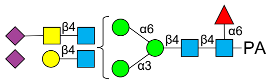	2.810	0.440	✓		N−Ldn
56.96	14.58	14.48	64.34		1026.94	1026.90	2H+	2051.79	H4Hn5dH2	F-**BI**BsF(6)-Ga 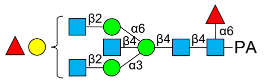	2.400	0.375			
58.09	15.06	14.87	65.48	64.27	1224.36	1224.46	2H+	2446.90	N2H5Hn4dH1	66N-**BI**F(6) 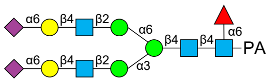	46.135	7.218	✓		
58.09	15.06	14.79	65.48		1119.88	1119.94	2H+	2237.86	N1H3Hn6dH1	06N-dLdn12-**M3**F(6) 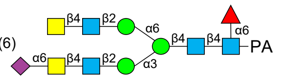	13.557	2.121			N-Ldn
58.09	15.06	15.35	65.48		1138.87	1139.40	2H+	2276.79	S1N1H4 Hn5dH1	S-N-Ldn1-**MO2**F(6) 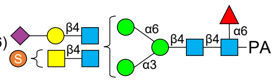	3.767	0.589			S-Ldn
58.09	15.06		65.48		1054.01	1054.34	2H+	2106.67	S2H3Hn6dH1	dS-dLdn-**M3**F(6)					
58.78	15.37	15.57	66.18	66.49	1479.32	1479.54	2H+	2957.07	N3H6Hn5	trN-Ln-**BI** 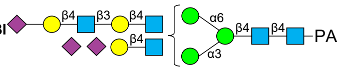	2.518	0.394	✓		N-dLn

Table S5 continued.

RT (min)	GU		RP		*m/z*			MS	Composition (+PA)	Glycan structure	Quantity ratio		MS²	Only	Note
	experi-mental	std.	experi-mental	std.	experi-mental	theoretical	ion form				% for top	% for whole			
58.78	15.37	15.86	66.18	65.53	1078.62	1078.91	2H+	2155.80	N1H5Hn4dH1	60N-**BIF**(6) 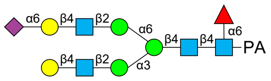	1.775	0.278	✓		
58.78	15.37		66.18		1244.51	1244.97	2H+	2487.93	N2H4Hn5dH1	36N-Ldn2-**MO1**F(6)					
58.78	15.37		66.18		974.29	974.39	2H+	1946.76	H3Hn6dH1	dLdn12-**M3**F(6) 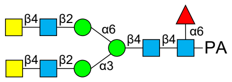	1.108	0.173			Ldn
58.78	15.37		66.18		1053.81	1054.34	2H+	2106.67	S2H3Hn6dH1	dS-dLdn-**M3**F(6) 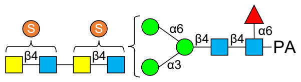	1.846	0.289			S-Ldn
58.78	15.37	14.79	66.18		1119.55	1119.94	2H+	2237.86	N1H3Hn6dH1	06N-dLdn12-**M3**F(6) 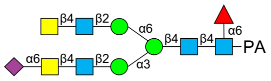	0.675	0.106	✓		N-Ldn
58.78	15.37	14.87	66.18	64.27	1224.25	1224.46	2H+	2446.90	N2H5Hn4dH1	66N-**BI**F(6)					
59.74	15.80	15.48	67.15		1244.86	1244.97	2H+	2487.93	N2H4Hn5dH1	36N-Ldn2-**MO1**F(6) 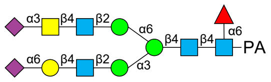	4.042	0.632	✓		N-Ldn
59.74	15.80	15.85	67.15		1157.22	1157.09	3H+	3468.26	N3Hn7H6dH1	trN-**TE**F(6) 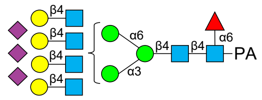	0.681	0.107			
59.74	15.80	16.73	67.15		961.52	961.87	2H+	1921.73	H5Hn5	**BI**Bs 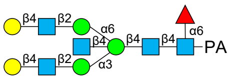	0.048	0.007			
61.04	16.41	16.30	68.42	67.84	1224.08	1224.46	2H+	2446.90	N2H5Hn4dH1	36N-**BI**F(6)					
61.04	16.41	16.26	68.42		1107.86	1107.93	2H+	2213.85	H5Hn5dH2	F(3)-**BI**BsF(6) 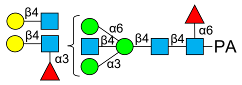	0.273	0.043			Le^x
61.04	16.41	16.73	68.42		961.46	961.87	2H+	1921.73	H5Hn5	**BI**Bs 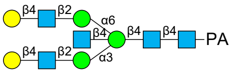	0.115	0.018			
61.46	16.61	16.30	68.82	67.84	1224.12	1224.46	2H+	2446.90	N2H5Hn4dH1	36N-**BI**F(6) 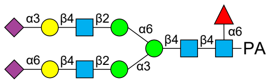	16.028	2.508	✓		
61.46	16.61	17.28	68.82		1078.48	1078.91	2H+	2155.80	N1H5Hn4dH1	03N-**BI**F(6) 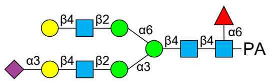	10.911	1.707	✓		
61.46	16.61	16.96	68.82		1479.08	1479.54	2H+	2957.07	N3H6Hn5	trN-**TR** 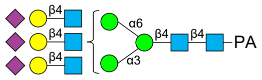	3.570	0.559			
62.14	16.94	17.66	69.47	68.72	1078.56	1078.91	2H+	2155.80	N1H5Hn4dH1	30N-**BI**F(6) 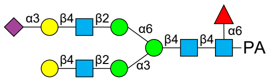	1.339	0.210	✓		
62.14	16.94		69.47		1261.28	1261.48	2H+	2520.94	N1H6Hn5dH1	N-**TR**F(6) 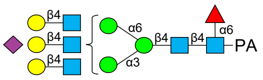	0.368	0.058			
63.34	17.55		70.63		1325.65	1326.00	2H+	2649.98	N2H5Hn5dH1	dN-Gn-**BI**F(6) 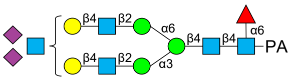	2.008	0.314			
63.34	17.55		70.63		1099.06	1099.42	2H+	2196.83	N1H4Hn5dH1	N-Ldn-**MO**F(6) 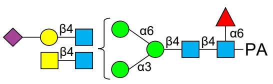	1.322	0.207			Ldn
63.34	17.55	17.43	70.63	70.68	1224.19	1224.46	2H+	2446.90	N2H5Hn4dH1	63N-**BI**F(6) 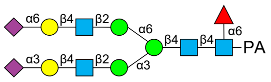	0.591	0.092			
64.94	18.41		72.16		1253.96	1254.13	3H+	3759.35	N4H7Hn6dH1	6N-3N-dN-F(3)-Ln-**TR** 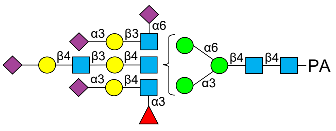	7.912	1.238			dN-Ln, N-dLn, SLe^x
64.94	18.41		72.16		1151.82	1151.94	2H+	2301.86	N1H5Hn4dH2	3N-F(3)-**BI**F(6) 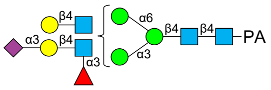	1.363	0.213			SLe^x

Table S5 continued.

RT (min)	GU experi-mental	GU std.	RP experi-mental	RP std.	*m/z* experi-mental	*m/z* theoretical	*m/z* ion form	MS	Composition (+PA)		Glycan structure	Quantity ratio % for top	Quantity ratio % for whole	MS^2	Only	Note
64.94	18.41		72.16		1156.95	1157.43	3H+	3469.28	N2H7Hn6dH3	dN-trF-**TE**	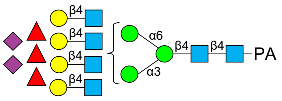	1.173	0.184			
65.86	18.93		73.04		1289.15	1289.99	2H+	2577.96	N1H6Hn6	N-**TR**Bs	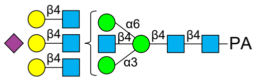	0.450	0.070			
65.86	18.93		73.04		1407.02	1407.02	2H+	2812.03	N2H6Hn4dH1	dN-**TRF**(6)	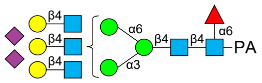	0.408	0.064			
65.86	18.93		73.04		1099.55	1099.42	2H+	2196.83	N1H4Hn5dH1	N-Ldn-**MO**F(6)	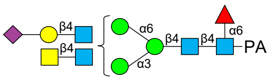	0.155	0.024			Ldn
67.01	19.61	19.43	74.15	73.91	1224.17	1224.46	2H+	2446.90	N2H5Hn4dH1	33N-**BI**F(6)	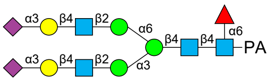	24.252	3.794	✓		
67.01	19.61		74.15		1407.24	1407.02	2H+	2812.03	N2H6Hn5dH1	dN-**TRF**(6)	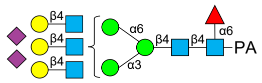	5.610	0.878			
67.01	19.61		74.15		1552.33	1552.57	2H+	3103.13	N3H6Hn5dH1	trN-**TRF**(6)	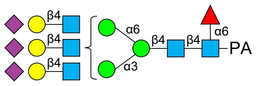	0.872	0.136			
68.11	20.29	21.99	75.20	77.61	872.62	872.85	2H+	1743.68	H3Hn5dH1	**AG12**BsF(6)	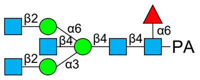	1.618	0.253	✓		
68.11	20.29		75.20		1216.04							0.213	0.033			
69.92	21.50	20.35	76.94		1552.33	1552.57	2H+	3103.13	N3H6Hn5dH1	trN-**TRF**(6)	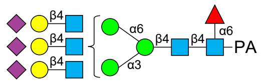	9.222	1.443			
71.41	22.59	21.99	78.37	77.61	1180.37	1180.45	2H+	2358.88	N1H5Hn5dH1	06N-**BI**BsF(6)	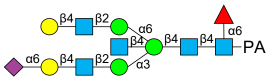	3.832	0.600	✓		
71.41	22.59	22.47	78.37	78.21	953.83	953.87	2H+	1905.73	H4Hn5dH1	**BI**BsF(6)-Ga2	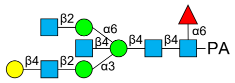	1.255	0.196	✓		
71.41	22.59	22.02	78.37		1552.85	1552.57	2H+	3103.13	N3H6Hn5dH1	trN-F-**TR**	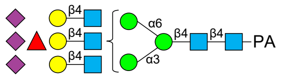	1.114	0.174			
71.41	22.59		78.37		1261.42	1261.48	2H+	2520.94	N1H6Hn5dH1	N-**TRF**(6)	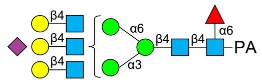	0.577	0.090			
71.41	22.59		78.37		1406.83	1407.02	2H+	2812.03	N2H6Hn5dH1	dN-**TRF**(6)	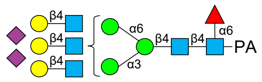	0.338	0.053			
72.00	23.04	23.53	78.94		1325.65	1326.00	2H+	2649.98	N2H5Hn4dH1	66N-**BI**BsF(6)	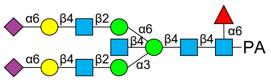	2.850	0.446	✓		
72.00	23.04	23.29	78.94		1406.89	1407.02	2H+	2812.03	N2H6Hn5dH1	dN-**TRF**(6)	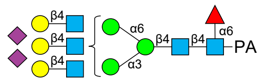	1.517	0.237			
72.00	23.04	23.35	78.94	78.21	953.60	953.87	2H+	1905.73	H4Hn5dH1	**BI**BsF(6)-Ga1	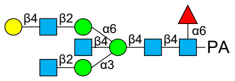	0.502	0.079	✓		
72.00	23.04		78.94		1454.82							0.343	0.054			
73.45	24.22	23.84	80.33	80.00	1034.79	1034.90	2H+	2067.79	H5Hn5dH1	**BI**BsF(6)	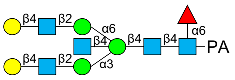	8.156	1.276	✓		
73.45	24.22	23.29	80.33		1407.20	1407.02	2H+	2812.03	N2H6Hn5dH1	dN-**TRF**(6)	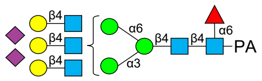	2.486	0.389			
73.45	24.22		80.33		1363.01	1363.01	2H+	2724.02	N1H6Hn6dH1	N-**TR**BsF(6)	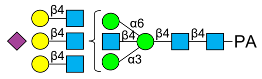	1.137	0.178		✓	

Table S5 continued.

RT (min)	GU experi-mental	GU std.	RP experi-mental	RP std.	*m/z* experi-mental	*m/z* theoretical	*m/z* ion form	MS	Composition (+PA)		Glycan structure	Quantity ratio % for top	Quantity ratio % for whole	MS^2	Only	Note
73.45	24.22		80.33		1217.33	1217.47	2H+	2432.92	H6Hn6dH1	**TR**BsF(6)	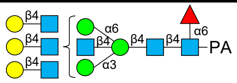	0.572	0.089			
75.17	25.76	25.99	81.98		1325.85	1326.00	2H+	2649.98	N2H5Hn5dH1	36N-**BI**BsF(6)	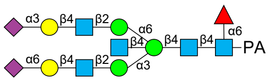	0.835	0.131			
75.17	25.76		81.98		1099.47	1099.42	2H+	2196.83	N1H4Hn5dH1	N-**BI**BsF(6)-Ga	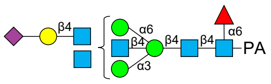	0.328	0.051			
75.17	25.76		81.98		1407.08	1407.02	2H+	2812.03	N2H6Hn4dH1	dN-**TR**F(6)	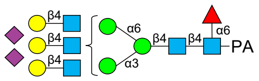	0.296	0.046			
75.55	26.12		82.34		1552.71	1552.57	2H+	3103.13	N3H6Hn5dH1	trN-**TR**F(6)	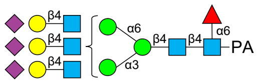	5.056	0.791	-		
75.55	26.12	24.25	82.34		1407.25	1407.02	2H+	2812.03	N2H6Hn5dH1	dN-**TR**F(6)	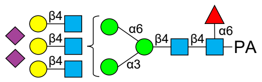	0.715	0.112			
75.55	26.12		82.34		1326.30	1326.00	2H+	2649.98	N2H5Hn5dH1	dN-**BI**BsF(6)	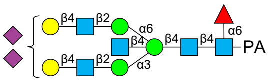	0.240	0.038			
75.55	26.12	26.99	82.34		1144.34	1144.44	2H+	2286.86	H6Hn6	**TR**Bs	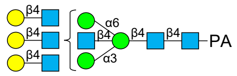	0.229	0.036			
76.76	27.32	27.33	83.50	83.11	1180.13	1180.45	2H+	2358.88	N1H5Hn5dH1	30N-**BI**BsF(6)	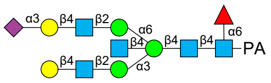	0.607	0.095			
77.48	28.08		84.19		1552.84	1552.57	2H+	3103.13	N3H6Hn5dH1	trN-Ln-**BI**F(6)	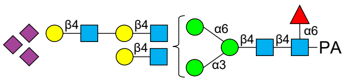	0.324	0.051			dLn
77.48	28.08	27.52	84.19		1132.17	1132.41	3H+	3394.22	N4H6Hn5dH1	teN-**TR**F(6)	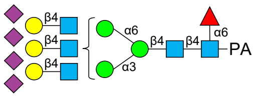	0.167	0.026			
77.48	28.08		84.19		1107.71	1107.93	2H+	2213.85	H5Hn5dH2	F-**BI**BsF(6)	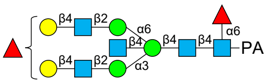	0.160	0.025			
78.15	28.82		84.84		1098.85	1099.42	2H+	2196.83	N1H4Hn5dH1	N-**BI**BsF(6)-Ga	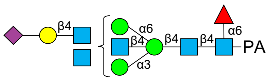	0.262	0.041			
79.17	30.00		85.81		1180.58	1180.96	2H+	2359.90	H5Hn5dH3	dF-**BI**BsF(6)	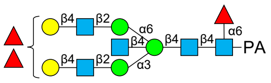	0.352	0.055			
79.17	30.00		85.81		1363.17	1363.01	2H+	2724.02	N1H6Hn6dH1	N-**TR**BsF(6)	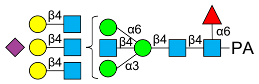	0.107	0.017			
80.28	31.38		86.88		1326.12	1326.00	2H+	2649.98	N2H5Hn5dH1	63N-**BI**BsF(6)	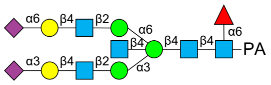	1.522	0.238			
81.33	32.78		87.89		1209.29							8.837	1.383		✓	
82.71	34.77	34.05	89.21		1290.73	1290.50	2H+	2578.98	H6Hn6dH2	F(3)-**TR**BsF(6)	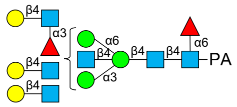	1.984	0.310	-		Le^x
84.44	37.52		90.87		1217.16	1217.47	2H+	2432.92	H6Hn6dH1	**TR**BsF(6)	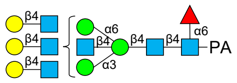	0.986	0.154			
87.27	42.74		93.59		1435.69	1435.53	2H+	2870.07	N1H6Hn6dH2	N-F-**TR**BsF(6)	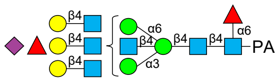	0.861	0.135			

Table S6. *N*-glycans from CHO-K1 analyzed using the improved method.
In the "Note" column, "pep" denotes peptide-like peaks based on MS/MS data.

RT (min)	GU experi-mental	GU std.	RP experi-mental	RP std.	*m/z* experi-mental	*m/z* theoretical	*m/z* ion form	MS	Composition (+PA)	Glycan structure		Quantity ratio % for top	Quantity ratio % for whole	MS^2	Note
23.51	5.09	5.11	29.89	29.07	899.87	900.33	2H+	1798.65	H8Hn2	**M8A**	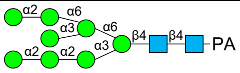	50.58	8.47	✓	
23.51	5.09		29.89		651.12		H+							✓	pep
24.74	5.29	5.31	31.07	30.36	818.82	819.31	2H+	1636.60	H7Hn2	**M7A**	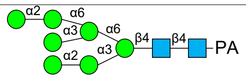	18.44	3.09	✓	
25.87	5.48	5.54	32.15	31.33	980.97	981.36	2H+	1960.70	H9Hn2	**M9A**	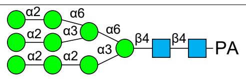	17.95	3.01	✓	
25.87	5.48		32.15		652.15		H+							✓	pep
27.41	5.75		33.63		635.04		H+							✓	pep
27.41	5.75	5.76	33.63	32.76	899.96	900.33	2H+	1798.65	H8Hn2	**M8B**	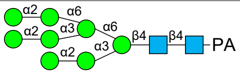	2.99	0.50	-	
28.05	5.87	5.88	34.25	33.44	818.95	819.31	2H+	1636.60	H7Hn2	**M7B**	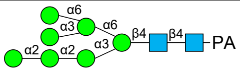	11.36	1.90	✓	
28.05	5.87		34.25		907.81		3H+					0.68	0.11		
28.74	6.00		34.91		636.14		H+							✓	pep
28.74	6.00		34.91		629.11		2H+							✓	pep
28.74	6.00	6.11	34.91	34.37	738.07	738.28	2H+	1474.54	H6Hn2	**M6B**					
29.29	6.11	6.11	35.44	34.37	737.95	738.28	2H+	1474.54	H6Hn2	**M6B**	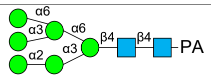	100	16.75	✓	
29.29	6.11	6.15	35.44		980.98	981.36	2H+	1960.70	H9Hn2	**G1M8A**	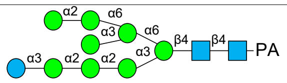	0.85	0.14		
29.29	6.11		35.44		636.19		H+							✓	pep
29.29	6.11	6.72	35.44		1151.41	1151.45	H+	1150.44	H4Hn2	**M4A**	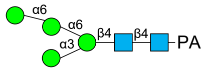	0.17	0.03		
29.90	6.23		36.02		737.86	738.28	2H+	1474.54	H6Hn2	**M6B**					
31.03	6.46	6.48	37.11	36.27	899.90	900.33	2H+	1798.65	H8Hn2	**M8C**	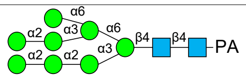	2.58	0.43	✓	
31.95	6.66	6.70	37.99		1062.20	1062.39	2H+	2122.76	H10Hn2	**G1M9A**	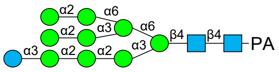	5.72	0.96	✓	
31.95	6.66		37.99		669.11		H+					0.58	0.10		
32.56	6.80	6.80	38.58	37.73	818.86	819.31	2H+	1636.60	H7Hn2	**M7D**	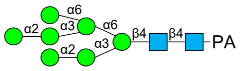	0.51	0.09	✓	
33.55	7.03	7.00	39.54	38.72	1313.49	1313.50	H+	1312.49	H5Hn2	**M5A**	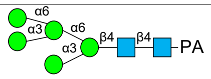	45.54	7.63	✓	
33.55	7.03	7.08	39.54	38.72	989.42	989.39	H+	988.39	H3Hn2	**M3B**	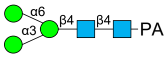	15.34	2.57	✓	
33.55	7.03	7.04	39.54	38.72	827.41	827.34	H+	826.33	H2Hn2	**M2B**	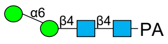	12.35	2.07	✓	
33.55	7.03		39.54		652.06		H+							✓	pep
33.55	7.03	7.06	39.54	38.72	1151.45	1151.45	H+	1150.44	H4Hn2	**M4B**	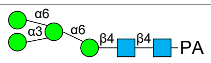	5.82	0.97	✓	
35.33	7.46		41.43		916.14		H+							✓	pep
35.33	7.46		41.43		1004.88	1005.88	2H+	2009.75	N1H5Hn4	N-**BI**/ga	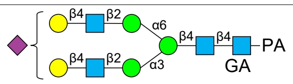				
35.58	7.53	7.52	41.70		839.81	839.82	2H+	1677.62	H6Hn3	Ln-**M5A**	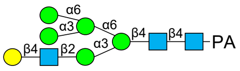	3.91	0.65		
37.04	7.91		43.25		659.09		H+							✓	pep
37.04	7.91		43.25		653.15		H+								pep
37.04	7.91		43.25		917.12		H+							✓	pep
38.03	8.18		44.30		675.00		H+							✓	pep
38.03	8.18		44.30		691.04		H+							✓	pep
38.03	8.18		44.30		669.09		H+					1.54	0.26		

Table S6 continued.

RT (min)	GU experi-mental	GU std.	RP experi-mental	RP std.	*m/z* experi-mental	*m/z* theoretical	*m/z* ion form	MS	Composition (+PA)		Glycan structure	Quantity ratio % for top	Quantity ratio % for whole	MS²	Note
38.03	8.18		44.30		653.10		?					0.67	0.11		
38.03	8.18		44.30		956.12		?					0.00	0.00		
39.98	8.73		46.37		917.15		H+							✓	pep
39.98	8.73	9.71	46.37		941.18	941.36	2H+	1880.70	H6Hn4	Ln-**M5A**Bs	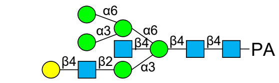	1.87	0.31		
41.63	9.22		48.13		956.14		H+							✓	pep
41.63	9.22	9.09	48.13		985.37	985.37	2H+		N1H6Hn3	3N-Ln-**M5A**	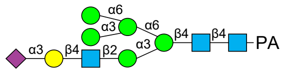	3.67	0.61	✓	
41.63	9.22		48.13		918.10		H+							✓	pep
42.34	9.44	9.54	48.88		932.38	933.36	2H+	1864.71	H5Hn4dH1	**BI**F(6)/ga	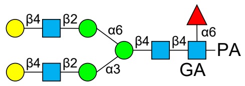			✓	
42.34	9.44	9.26	48.88		823.09	823.31	2H+	1644.61	N1H4Hn3	3N-**MO1**	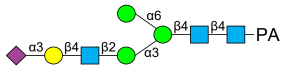	3.80	0.64	✓	
42.83	9.59	9.43	49.40		904.20	904.34	2H+	1806.67	N1H5Hn3	3N-Ln-**M4C**	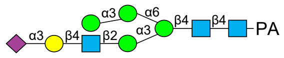	0.63	0.11	✓	
42.83	9.59	9.54	49.40		932.37	933.36	2H+	1864.71	H5Hn4dH1	**BI**F(6)/ga					
43.35	9.75	9.71	49.95		889.91	889.67	3H+	2665.97	N2H6Hn5	33N-**TR**	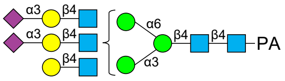	1.12	0.19	-	
43.35	9.75		49.95		980.13		H+					0.00	0.00	✓	pep
44.48	10.11	10.05	51.15		860.44	860.33	2H+	1718.65	H5Hn4dH1	**BI**	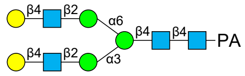	6.88	1.15	✓	
44.48	10.11		51.15		693.09		2H+							✓	pep
44.48	10.11		51.15		942.09		H+					0.26	0.04		
44.48	10.11	10.15	51.15		973.40	973.40	H+	972.39	H2Hn2dH1	**M2B**F(6)					
44.48	10.11		51.15		957.14		H+							✓	pep
44.99	10.28	10.15	51.69		973.42	973.40	H+	972.39	H2Hn2dH1	**M2B**F(6)	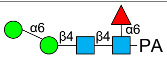	18.23	3.05	✓	
44.99	10.28	10.15	51.69	50.51	1135.46	1135.45	H+	1134.44	H3Hn2dH1	**M3B**F(6)	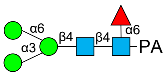	9.82	1.65	✓	
44.99	10.28	10.12	51.69		1115.66	1115.93	2H+	2229.84	H6H5dH1	**TR**F(6)	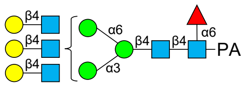	0.41	0.07	✓	
44.99	10.28		51.69		693.15		H+							✓	pep
46.36	10.73		53.04		986.92	986.70	3H+	2957.07	N3H6Hn5	trN-**TR**	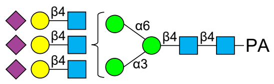	2.78	0.47		
46.36	10.73		53.04		693.15		H+							✓	pep
46.36	10.73		53.04		957.16		H+							✓	pep
46.36	10.73	10.95	53.04		1078.19	1078.91	2H+	2155.80	N1H5Hn4dH1	03N-**BI**F(6)/ga					
46.62	10.81	10.91	53.30		1034.94	1034.90	2H+	2067.79	H5Hn5dH1	F(3)-**BI**Bs	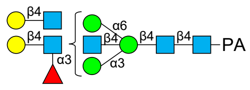	14.09	2.36		Le^x
47.62	11.15	10.95	54.28		1077.94	1078.91	2H+	2155.80	N1H5Hn4dH1	03N-**BI**F(6)/ga	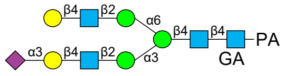			✓	
47.62	11.15		54.28		980.10		H+							✓	pep
48.49	11.45		55.14		981.12		H+							✓	pep
48.49	11.45		55.14		941.11		H+							✓	pep
48.49	11.45		55.14		709.11		H+							✓	pep
48.49	11.45	11.15	55.14		924.90	924.85	2H+	1847.69	N1H4Hn4	03N-**BI**-Ga2	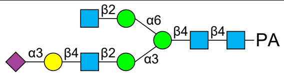	0.06	0.01		
50.17	12.03	11.68	56.79	56.09	771.34	771.31	2H+	1540.60	H3Hn4dH1	**AG12**F(6)	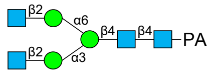	3.57	0.60	✓	

Table S6 continued.

RT (min)	GU experi-mental	GU std.	RP experi-mental	RP std.	*m/z* experi-mental	*m/z* theoretical	*m/z* ion form	MS	Composition (+PA)	Glycan structure		Quantity ratio % for top	Quantity ratio % for whole	MS^2	Note
50.17	12.03	11.65	56.79		1223.49	1224.46	2H+	2446.90	N2H5Hn4dH1	33N-**BI**F(6)/ga	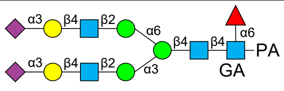	2.92	0.49	✓	
50.17	12.03		56.79		1261.40	1261.48	2H+	2520.94	N1H6Hn5dH1	N-**TR**F(6)	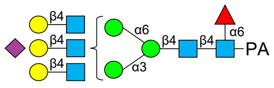	1.46	0.24	✓	
50.17	12.03		56.79		980.10		H+							✓	pep
50.17	12.03		56.79		611.18		?					0.39	0.06		
50.17	12.03	11.98	56.79		1006.35	1005.88	2H+	2009.75	N1H5Hn4	30N-**BI**					
50.69	12.22	11.98	57.30		1005.82	1005.88	2H+	2009.75	N1H5Hn4	30N-**BI**	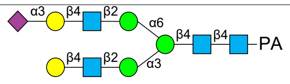	19.97	3.34	✓	
50.69	12.22		57.30		996.14		H+							✓	pep
50.69	12.22		57.30		1028.06		?					0.13	0.02		
51.77	12.61		58.36		693.08		H+							✓	pep
51.77	12.61	12.27	58.36		1005.86	1005.88	2H+	2009.75	N1H5Hn4	03N-**BI**	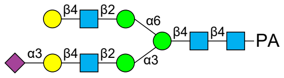	1.66	0.28	✓	
51.77	12.61		58.36		841.25	841.32	3H+	2520.94	N1H6Hn5dH1	N-**TR**F(6)	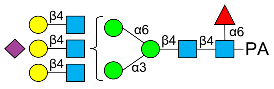	0.55	0.09	✓	
52.22	12.77	12.59	58.81		1224.85	1224.46	2H+	2446.90	N2H5Hn4dH1	33N-**BI**F(6)/epi	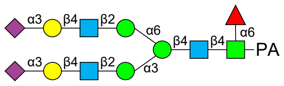	5.27	0.88	✓	
52.22	12.77		58.81		965.13		H+							✓	pep
53.13	13.10		59.70		997.14	997.88	2H+	1993.75	N1H4Hn4dH1	N-**BI**F(6)-Ga	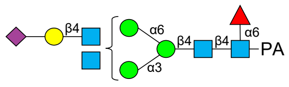	0.47	0.08		
53.13	13.10		59.70		980.16		H+							✓	pep
53.13	13.10		59.70		965.08		H+							✓	pep
53.13	13.10		59.70		957.05		H+							✓	pep
54.00	13.42		60.56		631.10		2H+							✓	pep
54.00	13.42		60.56		938.33	938.35	3H+	2812.03	N2H6Hn4dH1	dN-Ln-**BI**F(6)	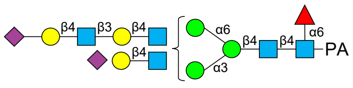	2.74	0.46	✓	N-dLn
54.55	13.63	13.61	61.10	60.83	933.37	933.36	2H+	1864.71	H5Hn4dH1	**BI**F(6)	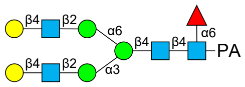	20.95	3.51	✓	
54.55	13.63		61.10		502.73		2H+							✓	pep
54.55	13.63		61.10		611.69		?					2.24	0.37		
54.55	13.63		61.10		1004.17		H+							✓	pep
55.50	13.99	11.79	62.03	60.83	623.25		2H+							✓	pep
55.50	13.99		62.03		933.46	933.36	2H+	1864.71	H5Hn4dH1	**BI**F(6)					
56.34	14.31	14.13	62.86	62.56	1151.28	1151.43	2H+	2300.84	N2H5Hn4	33N-**BI**	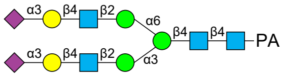	22.42	3.76	✓	
56.34	14.31	14.36	62.86		1035.58	1035.38	3H+	3103.13	N3H6Hn5dH1	trN-F-**TR**	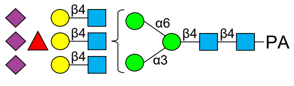	12.85	2.15	✓	
56.34	14.31		62.86		631.22		2H+							✓	pep
56.34	14.31		62.86		623.14		2H+							✓	pep
56.34	14.31		62.86		643.15		?							✓	pep
58.18	15.04		64.67		643.14		?					0.76	0.13		
58.18	15.04		64.67		1004.11		H+							✓	pep
58.18	15.04	14.99	64.67		986.96	986.70	3H+	2957.07	N3H6Hn5	trN-**TR**	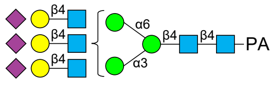	0.06	0.01		
59.03	15.38	15.35	65.50		986.71	986.70	3H+	2957.07	N3H6Hn5	trN-**TR**	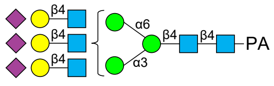	4.18	0.70		

Table S6 continued.

RT (min)	GU experi-mental	GU std.	RP experi-mental	RP std.	*m/z* experi-mental	*m/z* theoretical	*m/z* ion form	MS	Composition (+PA)	Glycan structure		Quantity ratio % for top	Quantity ratio % for whole	MS^2	Note
59.03	15.38		65.50		1060.18	1060.06	3H+	3177.16	N2H7Hn6dH1	dN-**TE**F(6)	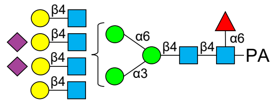	2.03	0.34		
61.42	16.38		67.85		643.26		2H+							✓	pep
61.42	16.38		67.85		651.11		2H+					1.69	0.28		
61.42	16.38		67.85		1157.18	1157.09	3H+	3468.26	N3Hn7H6dH1	trN-F-**TE**	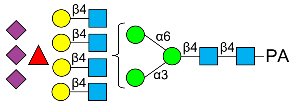	0.56	0.09		
61.42	16.38		67.85		631.38		2H+					0.21	0.03		
62.04	16.65	17.28	68.41		1078.97	1078.91	2H+	2155.80	N1H5Hn4dH1	03N-**BI**F(6)	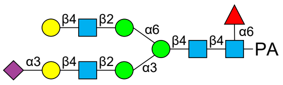	37.94	6.35	✓	
62.04	16.65	16.39	68.41		1157.24	1157.09	3H+	3468.26	N3H7Hn6dH1	trN-**TE**F(6)	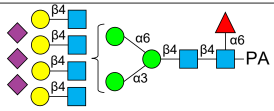	0.41	0.07		
62.04	16.65		68.41		1060.12	1060.06	3H+	3177.16	N2H7Hn6dH1	dN-dLn-**BI**F(6)	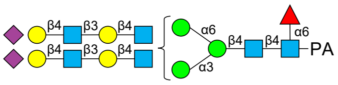	0.18	0.03		
62.63	16.91	17.66	68.94		1078.94	1078.91	2H+	2155.80	N1H5Hn4dH1	30N-**BI**F(6)	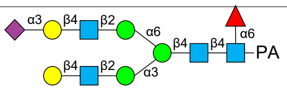	5.75	0.96	✓	
63.20	17.17		69.46		1157.06	1157.09	3H+	3468.26	N3Hn7H6dH1	trN-F-**TE**	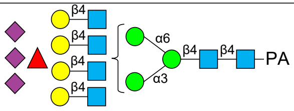	2.90	0.49		
63.20	17.17		69.46		651.12		?					2.05	0.34		
63.20	17.17		69.46		1078.94	1078.91	2H+	2155.80	N1H5Hn4dH1	30N-**BI**F(6)					
64.25	17.65		70.40		1232.56	1232.45	2H+	2462.89	Ng1N1H5Hn4dH1	Ng-N-**BI**F(6)	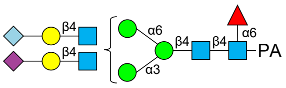	1.74	0.29	✓	Ng-Ln, N-Ln
64.25	17.65		70.40		635.06		2H+							✓	pep
65.13	18.07		71.20		1254.33	1254.13	3H+	3759.35	N4H7Hn6dH1	teN-Ln-**TR**F(6)	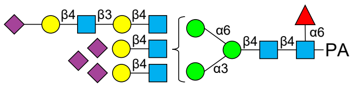	8.85	1.48	✓	N-dLn
65.13	18.07		71.20		635.25		2H+							✓	pep
65.13	18.07		71.20		643.12		2H+							✓	pep
65.13	18.07	17.70	71.20		1157.23	1157.09	3H+	3468.26	N3Hn7H6dH1	trN-**TE**F(6)	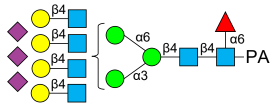	1.65	0.28		
66.46	18.73		72.40		655.26		?					1.07	0.18		
66.46	18.73		72.40		643.10		3H+					0.86	0.14		
66.46	18.73		72.40		1278.78		3H+					0.29	0.05		
66.46	18.73	18.74	72.40		1157.23	1157.09	3H+	3468.26	N3Hn7H6dH1	trN-**TE**F(6)	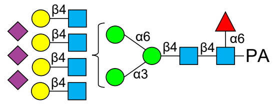	0.08	0.01		
67.46	19.25	19.41	73.31	73.91	1224.19	1224.46	2H+	2446.90	N2H5Hn4dH1	33N-**BI**F(6)	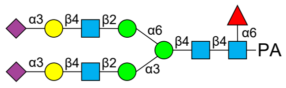	42.42	7.10	✓	
67.46	19.25		73.31		651.22		3H+					1.51	0.25		
67.46	19.25		73.31		663.17		3H+					0.56	0.09		
69.56	20.42		75.21		1375.82	1375.53	2H+	2749.05	N1H4Hn7dH2	N-F-dLdn-**MO**F(6)	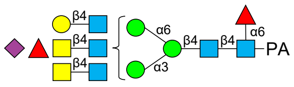	3.33	0.56		Ldn
71.80	21.81	22.65	77.23		938.40	938.35	3H+	2812.03	N2H6Hn4dH1	33N-Ln-**BI**F(6)	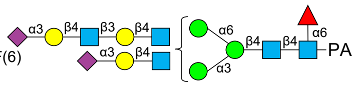	3.25	0.54		3N-dLn
72.95	22.59		78.27		655.22		H+					1.01	0.17		
72.95	22.59		78.27		1123.71		?					0.16	0.03		
73.72	23.14		78.97		655.13		3H+					3.34	0.56		

Table S6 continued.

RT (min)	GU experi-mental	GU std.	RP experi-mental	RP std.	*m/z* experi-mental	*m/z* theoretical	*m/z* ion form	MS	Composition (+PA)	Glycan structure	Quantity ratio % for top	Quantity ratio % for whole	MS[2]	Note
73.72	23.14		78.97		775.94		3H+				0.61	0.10		
73.72	23.14	23.07	78.97		938.24	938.35	3H+	2812.03	N2H6Hn4dH1	33N-Ln-**BI**F(6) 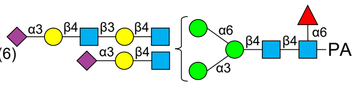	0.53	0.09		3N-dLn
74.27	23.55		79.47		663.26		H+				0.47	0.08		
75.73	24.70		80.79		1035.53	1035.38	3H+	3103.13	N3H6Hn5dH1	trN-F-**TR** 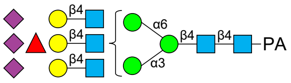	3.06	0.51		
75.73	24.70		80.79		655.66		3H+				2.25	0.38		
75.73	24.70		80.79		783.85		2H+				0.62	0.10		
76.85	25.67		81.80		659.19		H+						✓	pep
76.85	25.67		81.80		783.78		2H+				0.11	0.02		
76.85	25.67		81.80		667.18		3H+				0.07	0.01		
76.85	25.67		81.80		635.73		2H+				0.07	0.01		
77.69	26.43		82.56		667.16		3H+				1.13	0.19		

S1 Table. *N*-glycans from human serum pool analyzed by RP-LC-MS/MS.

For each sample, the relative yield of each glycan is shown, with the yield of 66N-BI normalized to 100.

Fig 1 No.	RT (min)	*m/z* experi-mental	*m/z* theoretical	*m/z* ion form	MS	Composition (+PA)		Glycan structure	Hydrazinolysis & Conventional 1	2	3	4	PNGase F & Conventional 1	2	3	Rapid PNGase F & Conventional 1	2	3	4	5	RapidPNGase F & BlotGlyco 1	2	3	4
5	23.62	889.75	900.33	2H+	1798.65	H8Hn2	**M8A**	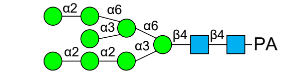	1.028	1.032	0.824	0.829	0.565	0.682	0.639	0.516	0.504	0.568	0.573	0.513	0.680	0.607	0.649	0.639
4	24.50	818.94	819.31	2H+	1636.60	H7Hn2	**M7A**	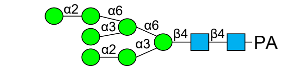	0.307	0.324	0.370	0.462	0.388	0.356	0.419	0.455	0.391	0.352	0.295	0.378	0.585	0.595	0.624	0.620
7	25.60	980.84	981.36	2H+	1960.70	H9Hn2	**M9A**	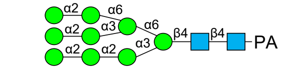	0.676	0.340	0.531	0.610	0.581	0.704	0.587	0.464	0.636	0.485	0.502	0.475	0.644	0.679	0.602	0.608
6	27.20	899.86	900.33	2H+	1798.65	H8Hn2	**M8B**	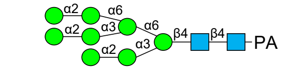	0.692	0.784	0.824	0.798	0.312	0.538	0.473	0.352	0.313	0.518	0.591	0.643	0.429	0.365	0.317	0.367
3	29.05	737.81	738.28	2H+	1474.54	H6Hn2	**M6B**	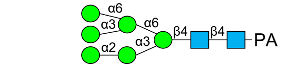	1.289	1.276	1.652	1.573	2.322	2.347	2.344	2.409	2.513	2.437	2.311	2.358	2.524	2.494	2.445	2.516
32	32.48	1005.84	1005.88	2H+	2009.75	N1H5Hn4	06N-**BI**/epi	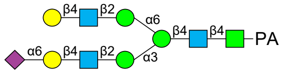	0.645	0.824	0.707	0.777	2.896	2.234	1.698	1.852	1.405	1.554	1.645	1.675	1.045	0.901	0.809	0.819
2	33.20	1313.44	1313.50	H+	1312.49	H5Hn2	**M5A**	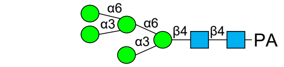	3.337	3.372	3.388	3.595	2.341	2.924	2.692	2.774	2.745	3.080	2.879	2.829	2.734	2.848	2.751	2.749
30	34.80	1150.60	1151.43	2H+	2300.84	N2H5Hn4	66N-**BI**/ga	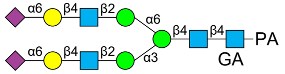	1.190	1.081	0.869	1.025	1.429	0.583	0.521	1.148	1.387	1.637	1.593	1.599	2.185	1.506	1.919	1.881
31	36.42	1151.44	1151.43	2H+	2300.84	N2H5Hn4	66N-**BI**/epi	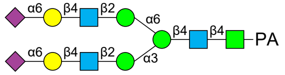	6.259	6.114	5.892	6.213	16.825	14.679	13.195	11.348	9.091	12.615	12.541	12.175	6.017	6.273	5.846	5.716
	37.04	1150.47	1151.43	2H+	2300.84	N2H5Hn4	36N-**BI**/ga	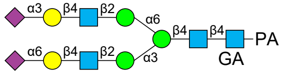	0.060	0.094	0.123	0.162	0.060	0.019	0.064	0.024	0.024	0.166	0.230	0.218	0.032	0.030	0.019	0.015
	37.04	985.30	985.37	2H+	1968.72	N1H6Hn3	6N-Ln-**M5A**	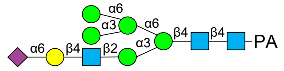	0.275	0.274	0.218	0.209	0.054	0.032	0.082	0.042	0.027	0.045	0.065	0.051	0.072	0.087	0.046	0.031
	37.04	823.34	823.31	2H+	1644.61	N1H4Hn3	6N-**MO1**	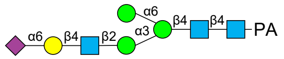	0.368	0.381	0.495	0.475	0.026	0.016	0.032	0.026	0.014	0.018	0.030	0.024	0.036	0.047	0.022	0.015
14	37.90	904.16	904.34	2H+	1806.67	N1H5Hn3	6N-Ln-**M4C**	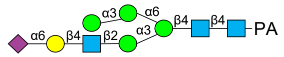	0.827	0.930	0.894	0.724	2.974	2.081	2.201	2.174	2.039	2.226	2.069	2.001	2.245	2.013	2.030	1.855
15	42.63	924.87	924.85	2H+	1847.69	N1H4Hn4	06N-**BI**-Ga2	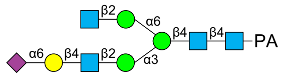	1.871	1.615	2.385	2.402	1.815	1.330	1.394	0.986	1.449	0.921	0.806	0.674	0.975	1.291	1.034	1.168
22	43.74	1224.50	1224.46	2H+	2446.90	N2H5Hn4dH1	N-3N-F(3)-**BI**	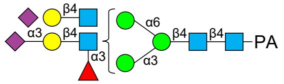	1.090	1.185	0.926	1.039	1.112	0.997	1.122	1.135	0.648	0.727	0.722	0.768	0.898	0.905	1.072	0.919

S1 Table continued.

Fig 1 No.	RT (min)	*m/z* experi-mental	*m/z* theoretical	*m/z* ion form	MS	Composition (+PA)	Glycan structure		Hydrazinolysis & Conventional 1	2	3	4	PNGase F & Conventional 1	2	3	Rapid PNGase F & Conventional 1	2	3	4	5	RapidPNGase F & BlotGlyco 1	2	3	4
	43.74	986.66	986.70	3H+	2957.07	N3H6Hn5	trN-**TR**	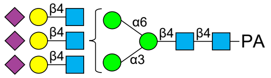	N.D.	N.D.	N.D.	N.D.	2.874	2.083	2.110	2.030	1.726	1.921	1.791	1.893	0.769	0.717	0.662	0.498
12	43.74	860.43	860.33	2H+	1718.65	H5Hn4	**BI**	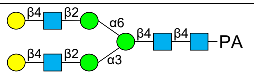	0.930	0.959	1.053	1.227	0.515	0.512	0.552	0.550	0.365	0.542	0.482	0.469	1.309	1.415	1.275	1.283
16	44.62	1006.01	1005.88	2H+	2009.75	N1H5Hn4	06N-**BI**	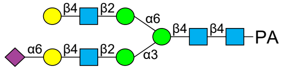	19.047	18.589	19.119	20.294	18.127	17.063	17.733	18.275	20.014	16.440	16.368	16.401	17.690	18.960	18.027	18.845
1	48.17	1151.78	1151.43	2H+	2300.84	N2H5Hn4	66N-**BI**	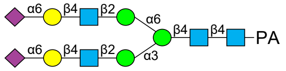	100	100	100	100	100	100	100	100	100	100	100	100	100	100	100	100
20	49.64	1151.51	1151.43	2H+	2300.84	N2H5Hn4	36N-**BI**	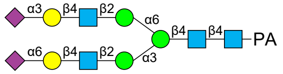	9.459	8.354	7.266	8.685	9.895	9.764	9.539	10.281	10.605	9.417	9.173	9.929	8.441	8.804	8.312	7.844
9	49.64	771.37	771.31	2H+	1540.60	H3Hn4dH1	**AG12**F(6)	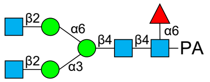	9.210	8.717	10.046	10.648	7.879	6.848	7.205	7.480	8.079	9.109	9.351	8.622	9.055	8.633	8.883	9.341
8	49.64	1338.48	1338.53	H+	1337.52	H3Hn3dH1	**AG2**F(6)	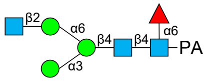	3.286	3.059	3.317	2.734	2.890	2.728	2.668	3.558	3.072	2.646	2.928	2.686	2.880	2.694	2.925	2.786
29	49.64	1205.59	1205.44	3H+	3613.30	N4H7Hn6	teN-**TE**	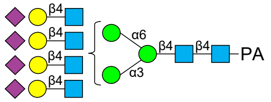	N.D.	N.D.	N.D.	N.D.	0.655	0.726	0.614	0.652	0.692	0.550	0.571	0.609	0.545	0.632	0.703	0.589
10	51.71	852.38	852.33	2H+	1702.66	H4Hn4dH1	**BI**F(6)-Ga2	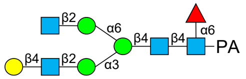	16.080	17.794	18.397	19.257	16.958	15.526	15.246	16.250	15.950	14.522	14.641	14.590	16.067	15.750	15.555	14.780
11	53.92	933.49	933.36	2H+	1864.71	H5Hn4dH1	**BI**F(6)	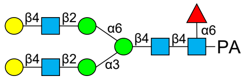	8.765	10.004	9.088	8.636	6.931	6.974	7.150	6.107	6.422	7.317	6.906	7.903	7.054	7.137	6.898	6.806
25	53.92	889.73	889.67	3H+	2665.97	N2H6Hn5	dN-**TR**	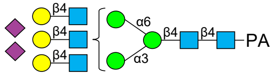	1.361	1.581	1.623	1.836	1.626	1.535	1.445	1.816	2.213	1.846	1.392	1.595	1.930	2.021	1.818	2.104
21	53.92	1151.16	1151.43	2H+	2300.84	N2H5Hn4	63N-**BI**	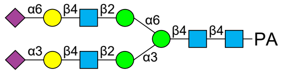	0.432	0.673	0.531	0.581	0.593	0.576	0.578	0.582	0.590	0.701	0.587	0.562	0.416	0.468	0.507	0.441
28	53.92	1035.51	1035.38	3H+	3103.13	N3H6Hn5dH1	dN-3N-F(3)-**TR**	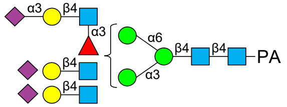	7.385	7.044	6.425	7.141	8.786	9.020	8.156	8.677	7.364	7.562	8.544	8.204	7.850	6.625	6.773	6.519
17	55.15	1078.94	1078.91	2H+	2155.80	N1H5Hn4dH1	06N-**BI**F(6)	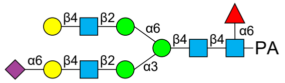	13.851	13.251	14.314	14.023	10.292	9.840	9.806	10.265	10.089	10.147	9.804	9.587	11.521	11.565	13.588	12.870

S1 Table continued.

Fig 1 No.	RT (min)	*m/z* experi-mental	*m/z* theoretical	*m/z* ion form	MS	Composition (+PA)	Glycan structure	Hydrazinolysis & Conventional 1	2	3	4	PNGase F & Conventional 1	2	3	Rapid PNGase F & Conventional 1	2	3	4	5	RapidPNGase F & BlotGlyco 1	2	3	4
23	56.96	1224.51	1224.46	2H+	2446.90	N2H5Hn4dH1	66N-**BIF**(6) 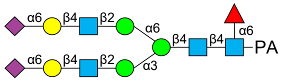	10.976	10.076	9.505	8.619	11.761	11.458	12.316	9.199	9.702	10.151	9.202	8.358	9.063	7.398	10.475	9.534
26	56.96	986.86	986.70	3H+	2957.07	N3H6Hn5	trN-**TR** 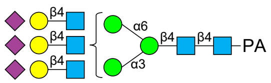	10.706	10.858	8.995	9.169	10.162	11.566	10.694	15.435	14.111	13.997	15.586	16.529	14.173	14.606	11.493	11.561
27	60.96	986.83	986.70	3H+	2957.07	N3H6Hn5	trN-**TR** 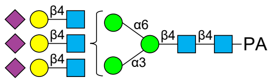	5.389	5.030	5.642	5.111	5.308	5.603	5.400	5.683	5.411	6.184	6.101	6.085	5.507	5.408	5.431	5.607
19	60.96	1078.88	1078.91	2H+	2155.80	N1H5Hn4dH1	03N-**BIF**(6) 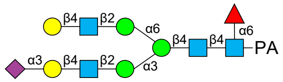	0.877	0.722	0.572	0.621	0.668	0.833	0.985	0.705	0.700	0.343	0.419	0.433	0.641	0.654	0.630	0.558
18	70.52	1180.45	1180.45	2H+	2358.88	N1H5Hn5dH1	06N-**BI**BsF(6) 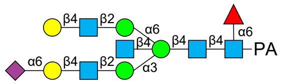	4.813	5.270	4.680	4.278	4.636	4.514	4.821	5.348	5.267	4.830	4.860	4.217	4.564	4.370	4.637	4.615
24	71.43	884.53	884.33	3H+	2649.98	N2H5Hn5dH1	66N-**BI**BsF(6) 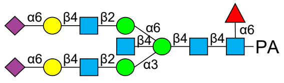	6.904	7.340	6.439	6.439	6.903	7.120	6.964	6.551	6.650	6.295	6.614	6.470	6.764	6.819	6.466	6.534
13	73.03	1035.29	1034.90	2H+	2067.79	H5Hn5dH1	**BI**BsF(6) 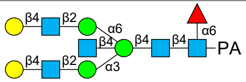	1.474	1.428	1.582	1.568	1.264	1.298	1.273	1.337	1.261	1.153	1.128	1.091	1.499	1.504	1.501	1.445
33	76.94	1172.13	1172.43	2H+	2342.85	N2H5Hn4Ac1	Ac-66N-**BI** 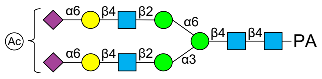	1.857	1.713	1.819	1.618	0.721	0.936	1.059	0.802	0.675	0.763	0.825	0.689	1.365	1.272	1.557	1.626
34	82.37	1172.13	1172.43	2H+	2342.85	N2H5Hn4Ac1	Ac-66N-**BI** 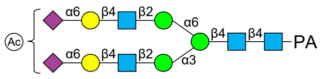	0.983	0.950	1.058	1.053	0.387	0.439	0.520	0.441	0.354	0.388	0.396	0.345	0.851	0.774	0.934	1.056
35	83.42	1172.13	1172.43	2H+	2342.85	N2H5Hn4Ac1	Ac-66N-**BI** 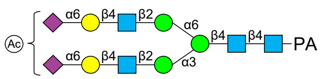	0.982	0.872	0.879	0.831	0.278	0.344	0.416	0.392	0.329	0.288	0.345	0.309	0.706	0.604	0.757	0.862
36	85.09	1172.13	1172.43	2H+	2342.85	N2H5Hn4Ac1	Ac-66N-**BI** 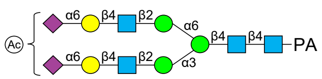	1.000	1.252	0.917	0.855	0.313	0.346	0.387	0.322	0.330	0.281	0.284	0.309	0.769	0.591	0.850	0.910

S2-1 Table. DEAE A3 fraction of *N*-glycans prepared from human urine using the improved method. Peak numbers correspond to those shown in Fig. 2. GU and RP indicate the normalized elution times based on PA-isomaltooligosaccharides and PA-*N*-glycan core structures, respectively. “Experimental” refers to the measured values. “Composition” indicates the presence of the following components: S (sulfate group), P (phosphate group), N (NeuAc), H (Hex), Hn (HexNAc), and dH (deoxyHex). “% for top” indicates the yield relative to the most abundant glycan in the fraction, while “% for whole” indicates the yield as a proportion of the entire fraction. In the MS^2 column, a check mark (✓) indicates glycan structures for which MS/MS data were obtained, while a dash (–) indicates that MS/MS data were acquired but insufficient for structural assignment. The “Note” column highlights characteristic substructures. “Hydrazine RT” indicates the retention times of peaks also observed in samples prepared by hydrazinolysis and conventional methods.

Peak No.	RT (min)	GU	RP	*m/z*			MS	Composition (+PA)	Glycan structure	Quantity ratio		MS^2	Note	Hydrazine
		experi-mental	experi-mental	experi-mental	theoretical	ion form				% for top	% for whole			RT (min)
1	47.26	10.93	54.57	1035.01	1035.38	3H+	3103.13	N3H6Hn5dH1	trN-**TRF**(6) 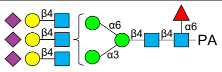	9.49	1.14			
	47.26	10.93	54.57	1066.18	1066.37	2H+	2130.73	S1N1H4Hn5	S-N-Ldn-**MO** 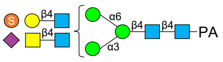	6.02	0.72		Ldn	47.16
2	48.19	11.25	55.51	1108.33	1108.41	3H+	3322.20	N3H7Hn6	trN-**TE** 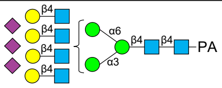	7.58	0.91			48.07
3	49.73	11.79	57.06	1108.68	1108.41	3H+	3322.20	N3H7Hn6	trN-**TE** 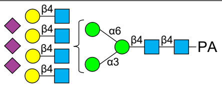	4.71	0.57			49.62
4	50.62	12.11	57.96	1156.74	1157.09	3H+	3468.26	N3H7Hn6dH1	trN-F-**TE**/ga 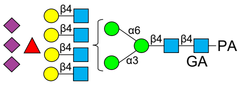					
	50.62	12.11	57.96	1086.84	1087.08	3H+	3258.22	N3H3Hn6dH4	trN-trF-dLdn-**M3**F(6) 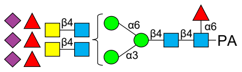	8.92	1.07		Ldn	50.58
5	52.21	12.69	59.56	1551.13	1552.57	2H+	3103.13	N3H6Hn5dH1	trN-F-**TR** 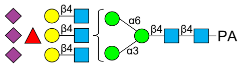	9.92	1.19			51.93
	52.21	12.69	59.56	1156.62	1157.09	3H+	3468.26	N3H7Hn6dH1	trN-**TE**F(6)/ga 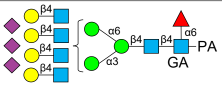					51.93
	52.21	12.69	59.56	1108.24	1108.41	3H+	3322.20	N3H7Hn6	trN-**TE** 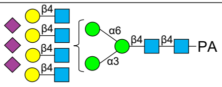	1.72	0.21			51.93
6	53.20	13.07	60.56	1551.11	1552.57	2H+	3103.13	N3H6Hn5dH1	trN-F-**TR** 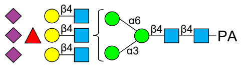	21.33	2.56			52.86
7	55.00	13.77	62.37	1551.91	1552.57	2H+	3103.13	N3H6Hn5dH1	trN-F-**TR**/ga 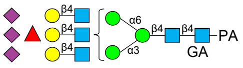					54.72
	55.00	13.77	62.37	1108.31	1108.41	3H+	3322.20	N3H7Hn6	trN-**TE** 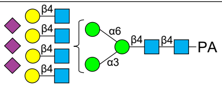	61.92	7.43			54.72
	56.48	14.38	63.86	1552.17	1552.57	2H+	3103.13	N3H6Hn5dH1	trN-F(3)-**TR**/ga 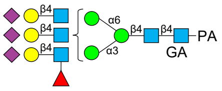			✓	Lex	56.28
8	57.81	14.94	65.20	1479.44	1479.54	2H+	2957.07	N3H6Hn5	trN-**TR** 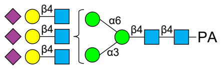	94.48	11.34	✓		57.55
9	58.81	15.38	66.21	1139.01	1139.40	2H+	2276.79	S1N1H4Hn5dH 1	S-N-F-Ldn-**MO** 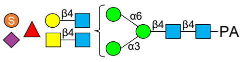	59.51	7.14		Ldn	58.64
	58.81	15.38	66.21	1479.68	1479.54	2H+	2957.07	N3H6Hn5	trN-**TR** 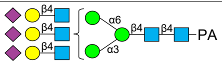	31.48	3.78			58.64
	58.81	15.38	66.21	1241.15	1240.79	3H+	3719.35	N3H8Hn5dH3	trN-dGa-trF-**TR** 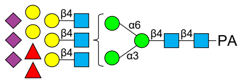	4.77	0.57			
10	60.03	15.93	67.44	1156.94	1157.09	3H+	3468.26	N3H7Hn6dH1	trN-**TE**F(6) 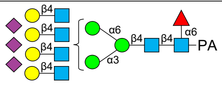	11.18	1.34			59.57
11	61.04	16.41	68.42	1156.76	1157.09	3H+	3468.26	N3H7Hn6dH1	trN-**TE**F(6) 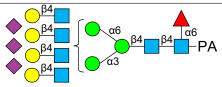	4.54	0.55			60.76

S2-1 Table continued.

Peak No.	RT (min)	GU experi-mental	RP experi-mental	*m/z* experi-mental	*m/z* theoretical	*m/z* ion form	MS	Composition (+PA)	Glycan structure	Quantity ratio % for top	Quantity ratio % for whole	MS^2	Note	Hydrazine RT (min)
12	61.88	16.81	69.23	1479.17	1479.54	2H+	2957.07	N3H6Hn5	trN-**TR** 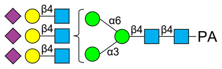	46.58	5.59	-		61.63
13	63.58	17.68	70.86	1156.84	1157.09	3H+	3468.26	N3H7Hn6dH1	trN-**TE**F(6) 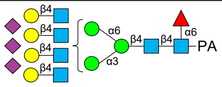	39.46	4.74			63.33
	63.58	17.68	70.86	1278.53	1278.80	3H+	3833.39	N3H8Hn7dH1	trN-**PE**F(6) 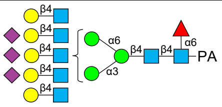	3.79	0.45			
14	65.38	18.65	72.58	1157.05	1157.09	3H+	3468.26	N3H7Hn6dH1	trN-**TE**F(6) 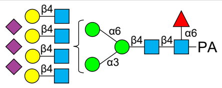	69.88	8.39			65.20
15	66.39	19.24	73.55	1156.89	1157.09	3H+	3468.26	N3H7Hn6dH1	trN-**TE**F(6) 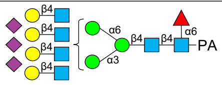	33.48	4.02			66.24
16	67.91	20.17	75.01	1552.05	1552.57	2H+	3103.13	N3H6Hn5dH1	trN-**TR**F(6) 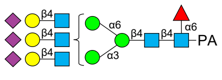	65.60	7.88	-		67.73
17	69.71	21.36	76.74	1170.71	1170.77	3H+	3509.29	N3H6Hn7dH1	trN-**TE**BsF(6)-Ga 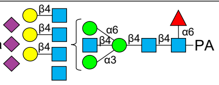	0.31	0.04			
	69.71	21.36	76.74	1176.13	1175.92	2H+	2349.83	S1N1H3Hn2dH4	S-N-trF-Ldn-**MO**F(6) 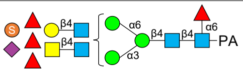	0.75	0.09		Ldn	69.61
	69.71	21.36	76.74	1278.95	1278.80	3H+	3833.39	N3H8Hn7dH1	trN-**PE**F(6) 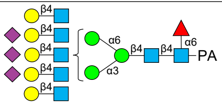	0.90	0.11			
18	70.27	21.75	77.28	1552.05	1552.57	2H+	3103.13	N3H6Hn5dH1	trN-**TR**F(6) 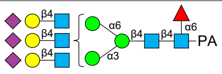	100	12.00	✓		70.11
19	71.46	22.62	78.42	1552.05	1552.57	2H+	3103.13	N3H6Hn5dH1	trN-**TR**F(6) 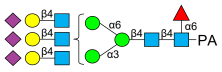	10.23	1.23			71.35
	71.46	22.62	78.42	1176.21	1176.10	3H+	3525.28	N3H7Hn5	trN-**TE**Bs 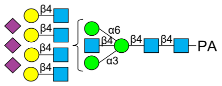	0.65	0.08			71.35
20	73.62	24.37	80.49	1552.05	1552.57	2H+	3103.13	N3H6Hn5dH1	trN-**TR**F(6) 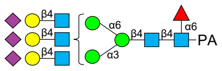	3.98	0.48			73.66
21	75.64	26.20	82.43	1552.34	1552.57	2H+	3103.13	N3H6Hn5dH1	trN-**TR**F(6) 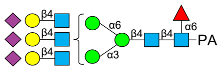	8.66	1.04			75.62
22	77.73	28.35	84.43	1552.05	1552.57	2H+	3103.13	N3H6Hn5dH1	trN-**TR**F(6) 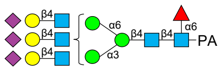	53.53	6.43			
23	83.61	36.16	90.07	1230.63	1230.80	3H+	3689.39	N3H3Hn9	trN-teLdn-**M3**Bs 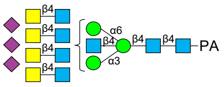	1.52	0.18		Ldn	
	83.61	36.16	90.07	1278.65	1278.80	3H+	3833.39	N3H8Hn7dH1	trN-**PE**F(6) 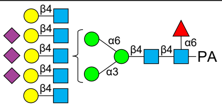	2.05	0.25			
24	87.09	42.38	93.41	1230.76	1230.80	3H+	3689.39	N3H3Hn9	trN-teLdn-**M3**Bs 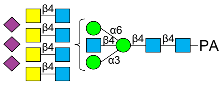	5.41	0.65		Ldn	
25	90.37	49.67	96.56	1279.50	1278.80	3H+	3833.39	N3H8Hn7dH1	trN-**PE**F(6) 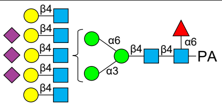	7.60	0.91			
26	92.05	54.04	98.17	1279.20	1278.80	3H+	3833.39	N3H8Hn7dH1	trN-**PE**F(6) 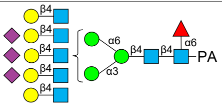	13.39	1.61			
27	93.46	58.08	99.52	1279.48	1278.80	3H+	3833.39	N3H8Hn7dH1	trN-**PE**F(6) 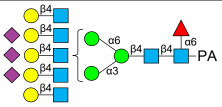	9.72	1.17			

S2-1 Table continued.

Peak No.	RT (min)	GU experi-mental	RP experi-mental	*m/z* experi-mental	*m/z* theoretical	*m/z* ion form	MS	Composition (+PA)	Glycan structure		Quantity ratio % for top	Quantity ratio % for whole	MS^2	Note	Hydrazine RT (min)
28	94.04	59.85	100.08	1279.42	1278.80	3H+	3833.39	N3H8Hn7dH1	trN-**PE**F(6)	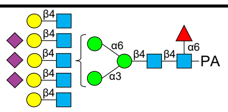	10.73	1.29			94.10
29	97.16	70.54	103.07	1279.16	1278.80	3H+	3833.39	N3H8Hn7dH1	trN-**PE**F(6)	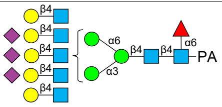	12.17	0.87			

S2-2 Table. DEAE A4 fraction of *N*-glycans prepared from human urine using the improved method.

Peak No.	RT (min)	GU	RP	*m/z*			MS	Composition (+PA)		Glycan structure	Quantity ratio		MS²	Note	Hydrazine
		experi-mental	experi-mental	experi-mental	theoretical	ion form					% for top	% for whole			RT (min)
30	28.23	5.96	35.05	1106.12	1106.38	2H+	2210.74	P1N1H7Hn3	6P-N-Ln-**M6**	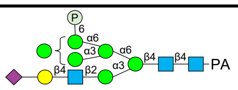	3.30	0.48			
31	29.50	6.20	36.27	1106.00	1106.38	2H+	2210.74	P1N1H7Hn3	6P-N-Ln-**M6**	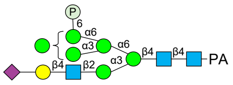	0.32	0.08			
	29.50	6.20	36.27	1178.10	1179.41	2H+	2356.80	P1N1H7Hn3dH1	6P-N-Ln-**M6**F(6)	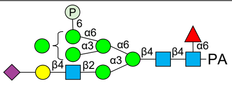	0.35	0.09			
32	37.64	8.05	44.53	1179.04	1179.41	2H+	2356.80	P1N1H7Hn3dH1	6P-N-F-Ln-**M6**	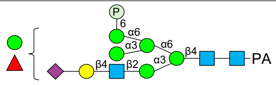	4.11	1.02	✓		
	40.41	8.81	47.45	1204.57	1205.44	3H+	3613.30	N4H7Hn6	teN-**TE**/ga	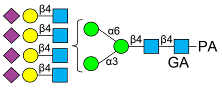					
33	42.11	9.30	49.25	1205.18	1205.44	3H+	3613.30	N4H7Hn6	teN-**TE**	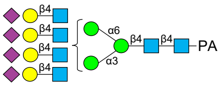	1.39	0.34			
	42.11	9.30	49.25	1062.57	1062.86	H+NH4+	2106.67	S2H3Hn6dH1	dS-dLdn12-**M3**F(6)						
34	42.71	9.48	49.88	1053.77	1054.34	2H+	2106.67	S2H3Hn6dH1	dS-dLdn12-**M3**F(6)	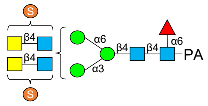	3.07	0.76		Ldn	
35	46.13	10.56	53.43	1191.71	1191.41	2H+	2380.80	S1N2H5Hn4	S-3N-6N-**BI**	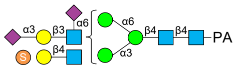	1.76	0.44		dN-Ln	
	47.49	11.01	54.80	1253.42	1254.13	3H+	3777.39	N4H7Hn6dH1	teN-**TE**F(6)/ga	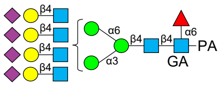	3.01	0.75			
	47.49	11.01	54.80	1368.70	1370.00	2H+	2737.99	N3H5Hn4dH1	trN-**BI**F(6)/ga	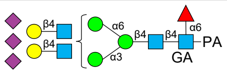	2.68	0.66			
36	49.41	11.67	56.74	981.16	981.32	2H+	1960.62	S2H3Hn6	dS-dLdn-**M3**	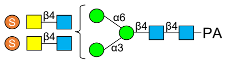	3.81	0.94		Ldn	
	49.41	11.67	56.74	1445.46							2.97	0.74			
	49.41	11.67	56.74	1253.54	1254.13	3H+	3777.39	N4H7Hn6dH1	teN-**TE**F(6)						
	49.41	11.67	56.74	1205.25	1205.44	3H+	3631.33	N4H7Hn6	teN-**TE**						
37	50.58	12.09	57.91	1205.32	1205.44	3H+	3631.33	N4H7Hn6	teN-**TE**	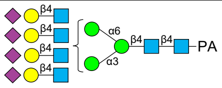	11.99	2.97			50.72
	50.58	12.09	57.91	1253.83	1254.13	3H+	3777.39	N4H7Hn6dH1	teN-**TE**F(6)	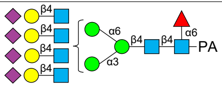	6.51	1.61			
	50.58	12.09	57.91	1446.41							1.61	0.40			
38	51.60	12.46	58.94	1253.72	1254.13	3H+	3777.39	N4H7Hn6dH1	teN-**TE**F(6)	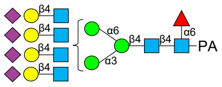	14.28	3.54			51.7
39	52.79	12.91	60.14	1253.72	1254.13	3H+	3777.39	N4H7Hn6dH1	teN-**TE**F(6)						
	52.79	12.91	60.14	1369.46	1370.00	2H+	2737.99	N3H5Hn4dH1	trN-**BI**F(6)	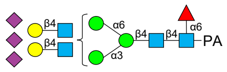	1.83	0.45			
40	54.54	13.59	61.91	1205.45	1205.44	3H+	3631.33	N4H7Hn6	teN-**TE**	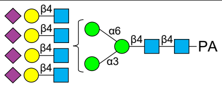	32.80	8.13			54.67
	54.54	13.59	61.91	1369.93	1370.00	2H+	2737.99	N3H5Hn4dH1	trN-**BI**F(6)	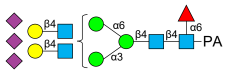	2.59	0.64			54.67
	54.54	13.59	61.91	1033.65							2.50	0.62			
41	55.75	14.08	63.12	1369.65	1370.50	3H+	4108.49	N4H7Hn7dH2	teN-F-**TE**BsF(6)	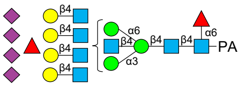	4.72	1.17	✓	dN-Ln	55.65
	55.75	14.08	63.12	1264.11	1264.44	2H+	2526.82	S1N2H5Hn4dH1	S-dN-**BI**F(6)	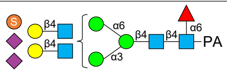	3.83	0.95			
	55.75	14.08	63.12	1253.85	1254.13	3H+	3759.35	N4H7Hn6dH1	teN-**TE**F(6)		0.88	0.22			

S2-2 Table continued.

Peak No.	RT (min)	GU	RP	*m/z*			MS	Composition (+PA)	Glycan structure		Quantity ratio		MS²	Note	Hydrazine
		experi-mental	experi-mental	experi-mental	theoretical	ion form					% for top	% for whole			RT (min)
42	56.54	14.40	63.92	1525.68							18.09	4.48			56.76
	56.54	14.40	63.92	1462.68	1463.04	2H+	2924.06	N4H4Hn5	teN-Ldn-**BI**	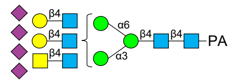	3.74	0.93		Ldn	
	56.54	14.40	63.92	1254.08	1254.13	3H+	3777.39	N4H7Hn6dH1	teN-**TE**F(6)	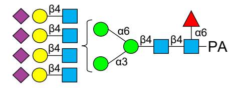	3.74	0.93			
	56.54	14.40	63.92	1525.07							1.51	0.37			
43	58.13	15.08	65.52	1263.97	1264.44	2H+	2526.82	S1N2H5Hn4dH1	S-dN-**BI**F(6)	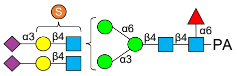	3.42	0.85		S-N-Ln	
44	58.76	15.36	66.16	1053.94	1054.34	2H+	2106.67	S2H3Hn6dH1	dS-dLdn-**M3**F(6)	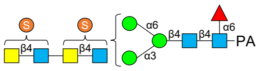	42.21	10.46	-	Ldn	58.95
45	60.37	16.09	67.78	1327.36	1327.83	3H+	3980.48	N4H3Hn11dH4	teN-teLdn-**M3**Bs	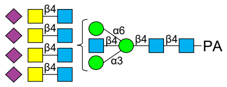	2.37	0.59		Ldn	
46	61.49	16.62	68.85	1253.91	1254.13	3H+	3777.39	N4H7Hn6dH1	trN-**TE**F(6)	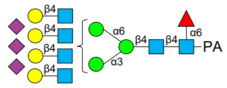	58.22	14.43			61.73
47	62.23	16.99	69.56	1322.21	1321.82	3H+	3962.43	N4H7Hn7dH1	teN-**TE**BsF(6)	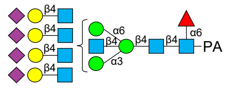	1.26	0.31			
	62.23	16.99	69.56	1254.13	1254.13	3H+	3777.39	N4H7Hn6dH1	trN-**TE**F(6)		0.54	0.13			
48	63.63	17.70	70.90	1321.92	1321.82	3H+	3962.43	N4H7Hn7dH1	teN-**TE**BsF(6)	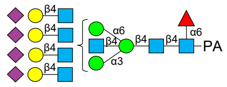	12.61	3.12			63.71
49	65.08	18.49	72.30	1253.86	1254.13	3H+	3777.39	N4H7Hn6dH1	trN-**TE**F(6)	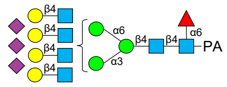	100	24.78	✓		65.29
50	66.23	19.14	73.40	1446.40	1447.04	2H+	2883.03	N4H5Hn7dH2	teN-F-Ldn-**BI**BsF(6)	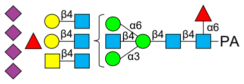	2.75	0.68		Ldn	
	66.23	19.14	73.40	1376.14	1376.52	3H+	4126.54	N4H3Hn11dH1	teN-teLdn-**M3**BsF(6)	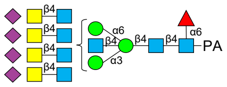	2.05	0.51		Ldn	
	66.23	19.14	73.40	1253.91	1254.13	3H+	3777.39	N4H7Hn6dH1	trN-**TE**F(6)		1.21	0.30			
51	67.95	20.19	75.05	1446.64	1447.04	2H+	2883.03	N4H5Hn7dH2	teN-F-Ldn-**BI**BsF(6)	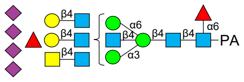	6.60	1.64		Ldn	68.10
	67.95	20.19	75.05	1203.51	1203.09	3H+	3606.26	S1N2H7Hn7dH2	S-dN-F-**TE**BsF(6)	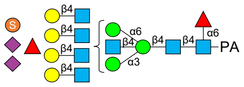	1.97	0.49			
52	69.86	21.46	76.88	1467.23	1467.51	2H+	2933.01	S1N2H5Hn6dH	S-dN-Ldn-**BI**F(6)	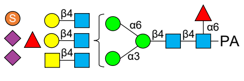	16.02	3.97		Ldn	70.06
	69.86	21.46	76.88	1375.92	1376.52	3H+	4144.57	N4H3Hn11dH1	teN-teLdn-**M3**BsF(6)	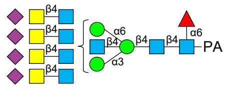	6.49	1.61		Ldn	
53	72.35	23.32	79.27	1446.69	1447.04	2H+	2883.03	N4H5Hn7dH2	teN-F-Ldn-**BI**BsF(6)	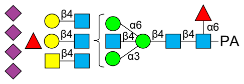	9.07	2.25		Ldn	72.34
54	73.42	24.20	80.30	1447.36	1447.04	2H+	2883.03	N4H5Hn7dH2	teN-F-Ldn-**BI**BsF(6)	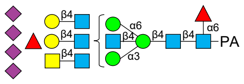	1.61	0.40		Ldn	
55	75.94	26.49	82.72	1446.90	1447.04	2H+	2883.03	N4H5Hn7dH2	teN-F-Ldn-**BI**BsF(6)	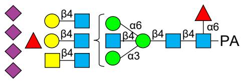	1.77	0.44		Ldn	

S2-3 Table. DEAE A5 fraction of *N*-glycans prepared from human urine using the improved method.

Peak No.	RT (min)	GU experi-mental	RP experi-mental	*m/z* experi-mental	*m/z* theoretical	*m/z* ion form	MS	Composition (+PA)	Glycan structure		Quantity ratio % for top	Quantity ratio % for whole	MS^2	Note	Hydrazine RT (min)
56	45.83	10.46	53.13	1191.28	1191.41	2H+	2380.80	S1N2H5Hn4	S-dN-**BI**	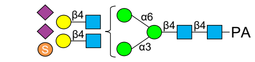	35.97	11.07			46.10
57	54.52	13.58	61.89	1291.36	1291.86	3H+	3872.40	N5H5Hn6dH2	peN-F-Ldn-**BI**F(6)	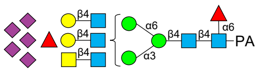	62.71	19.30			
58	58.92	15.43	66.32	1183.88	1184.09	3H+	3549.24	S1N2H7Hn6dH3	S-dN-dF-**TE**F(6)	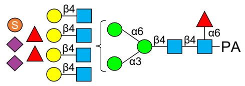	100	30.78			59.31
59	61.40	16.58	68.76	1184.18	1184.09	3H+	3549.24	S1N2H7Hn6dH3	S-dN-dF-**TE**F(6)	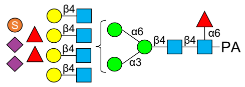	51.21	15.76			
60	64.50	18.17	71.74	1261.14	1260.92	2H+	2519.82	S2H5Hn5dH3	dS-dF-**BI**BsF(6)	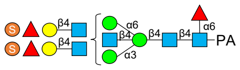	13.75	4.23			
61	68.11	20.29	75.20	1358.09	1357.98	2H+	2713.94	S1N2H4Hn5dH2	S-dN-F-Ldn-**MO**F(6)	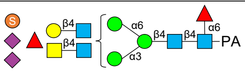	19.52	6.01			
	69.45	21.18	76.49	1340.86							25.04	7.71			
62	71.82	22.90	78.76	1132.17	1132.41	3H+	3412.26	N4H6Hn5dH1	teN-**TR**F(6)	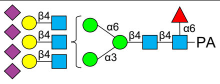	9.44	2.91			
63	77.48	28.08	84.19	1132.24	1132.41	3H+	3412.26	N4H6Hn5dH1	teN-**TR**F(6)	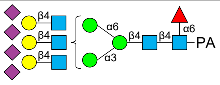	7.24	2.23			

S3 Table. *N*-glycans from human serum pool analyzed using the improved method.
Structures highlighted in blue span two fluorescence peaks with a lower abundance ratio; their area values were combined with the corresponding peak having the higher ratio. GU and RP indicate the normalized elution times based on PA-isomaltooligosaccharides and PA-*N*-glycan core structures, respectively. "Experimental" refers to the measured values, and "std." indicates standard values listed in SugarScan. "Composition" indicates the following components: Ac (acetyl group), S (sulfate group), P (phosphate group), N (NeuAc), H (Hex), Hn (HexNAc), and dH (deoxyHex). The "Quantity ratio" refers to the relative yield calculated from fluorescence peak areas. "% for top" indicates the yield relative to the most abundant glycan in the sample, while "% for whole" indicates the yield as a proportion of the entire sample. In the MS^2 column, a check mark (✓) indicates glycan structures for which MS/MS data were obtained, while a dash (–) indicates that MS/MS data were acquired but insufficient for structural assignment. The "Note" column highlights characteristic substructures.

RT (min)	GU		RP		*m/z*			MS	Composition (+PA)		Glycan structure	Quantity ratio		MS^2	Note
	experi-mental	std.	experi-mental	std.	experi-mental	theoretical	ion form					% for top	% for whole		
23.57	5.10	5.11	29.95	29.07	889.75	900.33	2H+	1798.65	H8Hn2	**M8A**	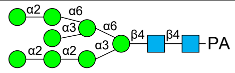	1.348	0.477	✓	
23.57	5.10		29.95		1186.44		2H+	2371.88	N2H2Hn2+1059.16			0.099	0.035	✓	
24.52	5.25	5.31	30.86	30.36	818.93	819.31	2H+	1636.60	H7Hn2	**M7A**					
24.75	5.29	5.31	31.08	30.36	818.94	819.31	2H+	1636.60	H7Hn2	**M7A**	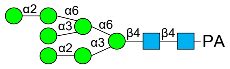	1.263	0.447		
24.75	5.29		31.08		903.07	904.34	2H+	1806.67	N1H5Hn3	6N-Ln-**M4C**/ga	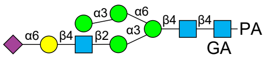			✓	
24.75	5.29	5.82	31.08		985.28	985.37	2H+	1968.72	N1H6Hn3	6N-Ln-**M5A**/epi	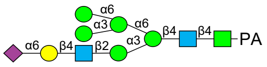	0.009	0.003		
25.92	5.49	5.50	32.20	31.33	980.84	981.36	2H+	1960.70	H9Hn2	**M9A**	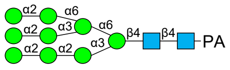	0.814	0.288	✓	
27.41	5.75	5.76	33.63	32.76	899.86	900.33	2H+	1798.65	H8Hn2	**M8B**	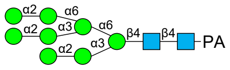	0.368	0.130		
28.12	5.88	5.88	34.31	33.44	818.85	819.31	2H+	1636.60	H7Hn2	**M7B**	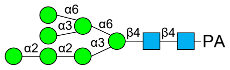	0.347	0.123		
28.12	5.88		34.31		1106.58	1107.42	2H+	2212.83	N1H5Hn5	N-**BIBs**/ga	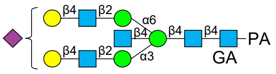				
29.34	6.12	6.11	35.48	34.37	737.81	738.28	2H+	1474.54	H6Hn2	**M6B**	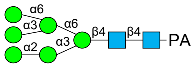	2.818	0.997	✓	
29.89	6.23		36.01		859.24	859.99	3H+	2576.96	N2H4Hn4dH3	N-**TRBs**/ga	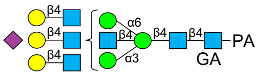				
29.89	6.23		36.01		1142.83		2H+					0.084	0.030	✓	
29.89	6.23	6.11	36.01	34.37	738.03	738.28	2H+	1474.54	H6Hn2	**M6B**					
31.96	6.67	6.72	38.00		1004.97	1005.88	2H+	2009.75	N1H5Hn4	06N-**BI**/ga	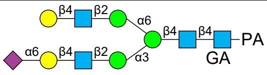			✓	
32.62	6.81	6.72	38.63		1005.12	1005.88	2H+	2009.75	N1H5Hn4	06N-**BI**/ga	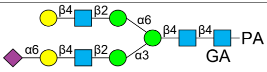			✓	
32.62	6.81		38.63		1223.49	1224.46	2H+	2446.90	N2H5Hn4dH1	66N-**BI**F(6)/epi	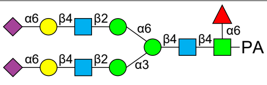	0.079	0.028		
32.62	6.81	6.80	38.63	37.73	818.88	819.31	2H+	1636.60	H7Hn2	**M7D**	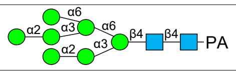	0.045	0.016		
33.12	6.93	6.92	39.11		1005.84	1005.88	2H+	2009.75	N1H5Hn4	06N-**BI**/epi	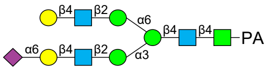	0.516	0.183	✓	
33.12	6.93	7.02	39.11	38.72	656.95	657.25	2H+	1312.49	H5Hn2	**M5A**					
33.58	7.04	7.01	39.58	38.72	1313.44	1313.50	H+	1312.49	H5Hn2	**M5A**	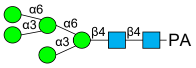	3.480	1.232	✓	
33.58	7.04	6.92	39.58		1005.96	1005.88	2H+	2009.75	N1H5Hn4	06N-**BI**/epi				✓	
34.31	7.21		40.35		1004.93	1005.88	2H+	2009.75	N1H5Hn4	30N-**BI**/ga	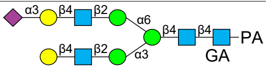			✓	

S3 Table continued.

RT (min)	GU experi-mental	GU std.	RP experi-mental	RP std.	*m/z* experi-mental	*m/z* theoretical	ion form	MS	Composition (+PA)		Glycan structure	Quantity ratio % for top	Quantity ratio % for whole	MS[2]	Note
35.68	7.55	7.56	41.81		1150.60	1151.43	2H+	2300.84	N2H5Hn4	66N-**BI**/ga	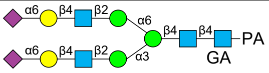			✓	
37.08	7.92	7.88	43.29		1151.44	1151.43	2H+	2300.84	N2H5Hn4	66N-**BI**/epi	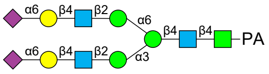	6.479	2.293	✓	
37.08	7.92	7.90	43.29		737.92	738.28	2H+	1474.54	H6Hn2	**M6C**	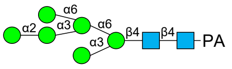	0.309	0.109	✓	
37.08	7.92	7.93	43.29		1188.47	1188.45	2H+	2374.88	N1H6Hn5	006N-**TR124**	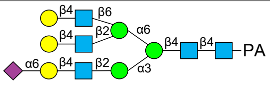	0.127	0.045	✓	
37.60	8.06	8.30	43.85		1150.47	1151.43	2H+	2300.84	N2H5Hn4	36N-**BI**/ga	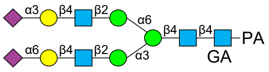			✓	
37.60	8.06	8.03	43.85	42.84	985.30	985.37	2H+	1968.72	N1H6Hn3	6N-Ln-**M5A**	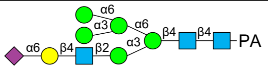	0.359	0.127	✓	
37.60	8.06	8.09	43.85	42.84	823.34	823.31	2H+	1644.61	N1H4Hn3	6N-**MO1**	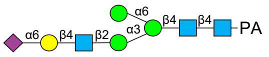	0.122	0.043		
38.20	8.22	8.25	44.48	43.68	904.16	904.34	2H+	1806.67	N1H5Hn3	6N-Ln-**M4C**	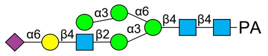	6.295	2.228	✓	
38.20	8.22	8.28	44.48		771.08	771.31	2H+	1540.60	H3Hn4dH1	**AG12**F(6)/epi	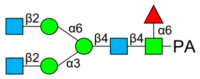	0.935	0.331	✓	
38.20	8.22		44.48		1150.51	1151.43	2H+	2300.84	N2H5Hn4	36N-**BI**/ga				✓	
38.60	8.34	8.30	44.91		1151.39	1151.43	2H+	2300.84	N2H5Hn4	36N-**BI**/epi	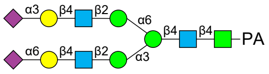	0.259	0.092		
38.60	8.34	8.25	44.91	43.68	904.42	904.34	2H+	1806.67	N1H5Hn3	6N-Ln-**M4C**					
38.60	8.34		44.91		1107.82	1108.41	3H+	3322.20	N3H6Hn5	trN-**TE**/ga	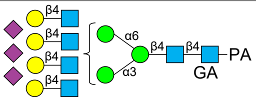				
39.73	8.66	8.67	46.11		770.33	771.31	2H+	1540.60	H3Hn4dH1	**AG12**F(6)/ga	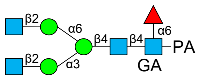			✓	
39.73	8.66	8.66	46.11		852.39	852.33	2H+	1702.66	H4Hn4dH1	**BI**F(6)-Ga/epi	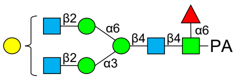	0.632	0.224	✓	
39.73	8.66	9.00	46.11		889.10	889.67	3H+	2665.97	N2H6Hn5	dN-**TR**	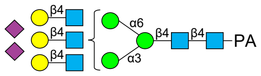	0.119	0.042		
39.73	8.66		46.11		1205.02	1205.44	3H+	3613.30	N4H7Hn6	teN-**TE**	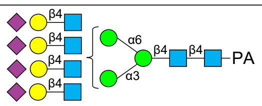	0.073	0.026		
39.73	8.66		46.11		938.03	938.35	3H+	2812.03	N2H6Hn4dH1	dN-F-**TR**/ga	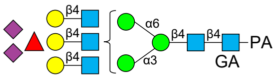				
39.73	8.66		46.11		792.60	792.63	3H+	2374.88	N1H6Hn5	N-**TR**	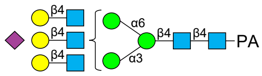	0.026	0.009		
41.08	9.05	9.02	47.54		851.49	852.33	2H+	1702.66	H4Hn4dH1	**BI**F(6)-Ga/ga	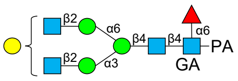			✓	
41.08	9.05		47.54		1034.82	1035.38	3H+	3103.13	N3H6Hn5dH1	trN-**TR**F(6)/ga	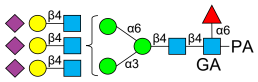			✓	
41.08	9.05	9.00	47.54		889.94	889.67	3H+	2665.97	N2H6Hn5	dN-**TR**	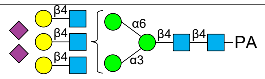	0.697	0.247	✓	
41.08	9.05		47.54		1106.30	1106.38	3H+	3016.13	N1H6Hn6dH3	N-trF-**TR**Bs	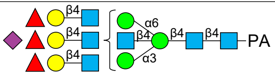	0.246	0.087		
42.06	9.35		48.58		986.06	986.70	3H+	2957.07	N3H6Hn5	trN-**TR**/ga	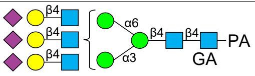			✓	
42.06	9.35		48.58		1035.51	1035.38	3H+	3103.13	N3H6Hn5dH1	trN-F-**TR**	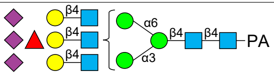	0.703	0.249		

S3 Table continued.

RT (min)	GU		RP		*m/z*			MS	Composition (+PA)	Glycan structure		Quantity ratio		MS[2]	Note
	experi-mental	std.	experi-mental	std.	experi-mental	theoretical	ion form					% for top	% for whole		
42.06	9.35	9.34	48.58		932.41	933.36	2H+	1864.71	H5Hn4dH1	**BI**F(6)/ga	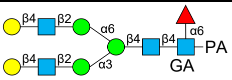			✓	
42.06	9.35	9.30	48.58		889.36	889.67	3H+	2665.97	N2H6Hn5	dN-**TR**	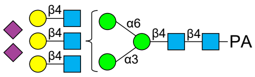	0.619	0.219		
42.06	9.35	9.33	48.58		779.18	779.31	3H+	1556.60	H4Hn2	**BI**-Ga2	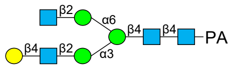	0.265	0.094	✓	
43.12	9.68		49.71		986.19	986.70	3H+	2957.07	N3H6Hn5	trN-**TR**/ga	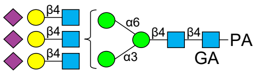			✓	
43.12	9.68	9.63	49.71	48.64	924.87	924.85	2H+	1847.69	N1H4Hn4	06N-**BI**-Ga2	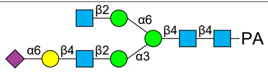	0.828	0.293	✓	
43.12	9.68	9.71	49.71		889.86	889.67	3H+	2665.97	N2H6Hn5	33N-**TR**	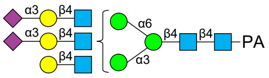	0.034	0.012	✓	
43.12	9.68		49.71		1205.54	1205.44	3H+	3613.30	N4H7Hn6	teN-**TE**	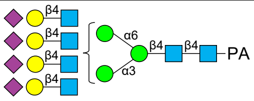	0.009	0.003		
43.55	9.81		50.17		986.11	986.70	3H+	2957.07	N3H6Hn5	trN-**TR**/ga					
43.55	9.81		50.17		914.44	914.35	3H+	2740.01	N1H7Hn6	N-**TE**	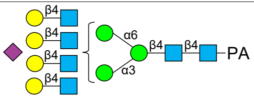	0.086	0.030		
43.55	9.81		50.17		1060.12	1060.06	3H+	3177.16	N2H7Hn6dH1	dN-F-**TE**	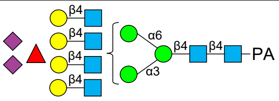	0.062	0.022		
44.37	10.08	9.98	51.04		1224.50	1224.46	2H+	2446.90	N2H5Hn4dH1	?3N-F(3)1-**BI**	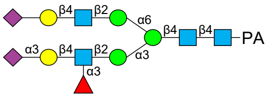	0.308	0.109	✓	SLe[x]
44.37	10.08		51.04		986.66	986.70	3H+	2957.07	N3H6Hn5	trN-**TR**	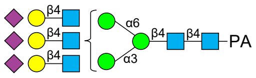	0.268	0.095	✓	
44.37	10.08	10.05	51.04		860.43	860.33	2H+	1718.65	H5Hn4	**BI**	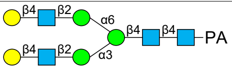	0.187	0.066	✓	
44.37	10.08	10.14	51.04		1077.70	1078.91	2H+	2155.80	N1H5Hn4dH1	06N-**BI**F(6)/ga				✓	
45.18	10.34	10.29	51.88	50.87	1006.01	1005.88	2H+	2009.75	N1H5Hn4	06N-**BI**	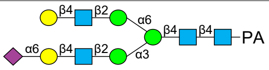	19.649	6.954	✓	
45.18	10.34		51.88		1354.48	1354.53	H+	1353.52	H4Hn3	**MO**/fr				✓	
45.18	10.34	10.14	51.88		1078.02	1078.91	2H+	2155.80	N1H5Hn4dH1	06N-**BI**F(6)/ga	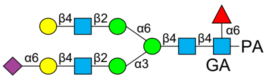			✓	
45.18	10.34	9.82	51.88		1340.68	1341.49	2H+	1340.49	H5Hn2Fo1	Fo-**M5A**	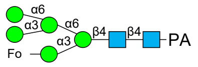	0.186	0.066		
45.95	10.59	10.55	52.64		816.11	816.64	3H+	2446.90	N2H5Hn4dH1	66N-**BI**F(6)/epi	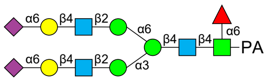	1.297	0.459	✓	
45.95	10.59	10.55	52.64		1011.63	1011.38	3H+	3031.11	N1H7Hn6dH2	dN-**TE**	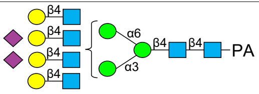	0.550	0.195	✓	
45.95	10.59		52.64		986.14	986.70	3H+	2957.07	N3H6Hn5	trN-**TR**/ga	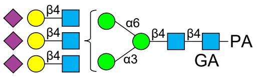			✓	
45.95	10.59	10.29	52.64	50.87	1005.93	1005.88	2H+	2009.75	N1H5Hn4	06N-**BI**				✓	
45.95	10.59		52.64		1157.31	1157.09	3H+	3468.26	N3Hn7H6dH1	trN-F-**TE**	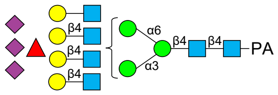	0.020	0.007		
47.05	10.96		53.72		986.72	986.70	3H+	2957.07	N3H6Hn5	trN-**TR**	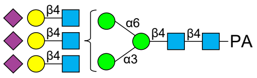	0.454	0.161	✓	
47.05	10.96	10.91	53.72		816.22	816.64	3H+	2446.90	N2H5Hn4dH1	36N-**BI**F(6)/ga	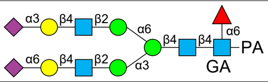			✓	

S3 Table continued.

RT (min)	GU experi-mental	GU std.	RP experi-mental	RP std.	*m/z* experi-mental	*m/z* theoretical	*m/z* ion form	MS	Composition (+PA)	Glycan structure	Quantity ratio % for top	Quantity ratio % for whole	MS^2	Note
47.05	10.96	10.55	53.72		1011.54	1011.38	3H+	3031.11	N2H7Hn6	dN-**TE**			✓	
47.05	10.96		53.72		1254.35	1254.13	3H+	3759.35	N4H7Hn6dH1	teN-F-**TE**				
47.54	11.12		54.20		1254.11	1254.13	3H+	3759.35	N4H7Hn6dH1	teN-F-**TE** 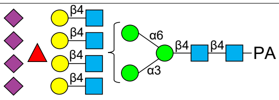	0.861	0.305		
47.54	11.12	11.06	54.20		816.74	816.64	3H+	2446.90	N2H5Hn4dH1	66N-**BI**F(6)/epi 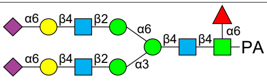	0.744	0.263	✓	
47.54	11.12		54.20		1108.65	1108.41	3H+	3322.20	N3H7Hn6	N-3N-6N-Ln-**TR** 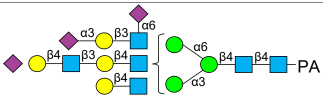	0.219	0.077	✓	dN-Ln, dLn
47.54	11.12	10.91	54.20		1077.84	1078.91	2H+	2155.80	N1H5Hn4dH1	30N-**BI**-Ga1 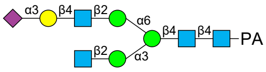	0.044	0.015		
47.54	11.12	10.55	54.20		1011.53	1011.38	3H+	3031.11	N2H7Hn6	dN-**TE** 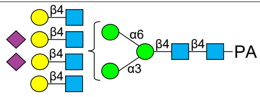	0.021	0.007		
47.54	11.12		54.20		986.91	986.70	3H+	2957.07	N3H6Hn5	trN-**TR** 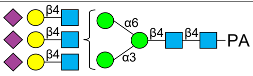	0.018	0.006		
48.35	11.40		55.00		1108.51	1108.41	3H+	3322.20	N3H7Hn6	N-3N-6N-Ln-**TR** 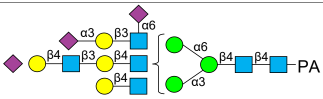	0.344	0.122	✓	dN-Ln, dLn
48.35	11.40	11.49	55.00	54.54	1151.75	1151.43	2H+	2300.84	N2H5Hn4	66N-**BI**				
48.35	11.40	10.55	55.00		1011.69	1011.38	3H+	3031.11	N2H7Hn6	dN-**TE**				
49.07	11.65	11.49	55.71	54.54	1151.78	1151.43	2H+	2300.84	N2H5Hn4	66N-**BI** 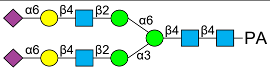	100	35.391	✓	
49.07	11.65		55.71		1108.74	1108.41	3H+	3322.20	N3H7Hn6	N-3N-6N-Ln-**TR** 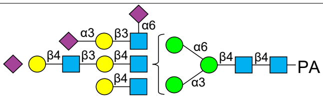	0.095	0.034	✓	dN-Ln, dLn
50.55	12.17	12.02	57.16	56.14	1151.51	1151.43	2H+	2300.84	N2H5Hn4	36N-**BI** 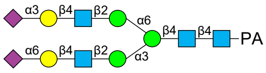	11.871	4.201	✓	
50.55	12.17	12.00	57.16	56.09	771.37	771.31	2H+	1540.60	H3Hn4dH1	**AG12**F(6) 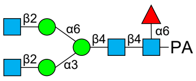	7.154	2.532	✓	
50.55	12.17	13.10	57.16	59.36	1338.48	1338.53	H+	1337.52	H3Hn3dH1	**AG2**F(6) 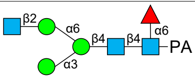	2.529	0.895	✓	
50.55	12.17	11.98	57.16	55.95	1005.98	1005.88	2H+	2009.75	N1H5Hn4	30N-**BI** 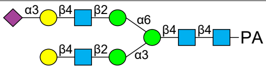	1.882	0.666	✓	
50.55	12.17	12.08	57.16	57.74	1205.59	1205.44	3H+	3613.30	N4H7Hn6	teN-**TE** 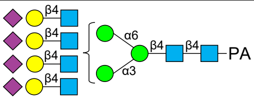	1.274	0.451	✓	
52.10	12.73	13.08	58.69		1034.86	1035.38	3H+	3103.13	N3H6Hn5dH1	trN-F-**TR** 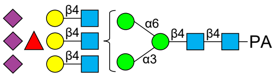	0.106	0.038		
52.10	12.73	12.27	58.69	56.80	1005.79	1005.88	2H+	2009.75	N1H5Hn4	03N-**BI** 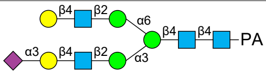	0.101	0.036		
52.10	12.73	12.73	58.69		1188.27	1188.45	2H+	2374.88	N1H6Hn5	N-Ldn-**BI** 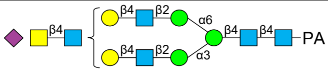	0.053	0.019		Ldn
52.10	12.73	13.08	58.69		852.02	852.33	2H+	1702.66	H4Hn4dH1	**BI**F(6)-Ga2				
52.76	12.97	13.08	59.34		852.38	852.33	2H+	1702.66	H4Hn4dH1	**BI**F(6)-Ga2 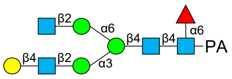	14.543	5.147	✓	
52.76	12.97	13.02	59.34		938.61	938.35	3H+	2812.03	N2H6Hn4dH1	dN-**TR**F(6) 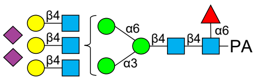	0.158	0.056		
52.76	12.97		59.34		874.74		2H+		Hn-H-Hn-(S-Hn-H-)+1017.13		0.147	0.052	✓	
53.64	13.29	13.42	60.20		1026.26	1026.39	2H+	2050.77	N1H4Hn5	06N-**BI**Bs-Ga2 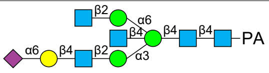	0.497	0.176	✓	

S3 Table continued.

RT (min)	GU experi-mental	GU std.	RP experi-mental	RP std.	*m/z* experi-mental	*m/z* theoretical	*m/z* ion form	MS	Composition (+PA)		Glycan structure	Quantity ratio % for top	Quantity ratio % for whole	MS²	Note
53.64	13.29	13.02	60.20		938.49	938.35	3H+	2812.03	N2H6Hn4dH1	dN-**TRF**(6)	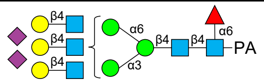	0.035	0.012		
53.64	13.29	13.59	60.20	61.78	1205.50	1205.44	3H+	3613.30	N4H7Hn6	teN-**TE**	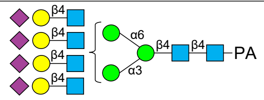	0.014	0.005		
53.64	13.29		60.20		852.42	852.33	2H+	1702.66	H4Hn4dH1	**BI**F(6)-Ga2					
55.12	13.85	13.61	61.66	60.83	933.49	933.36	2H+	1864.71	H5Hn4dH1	**BI**F(6)	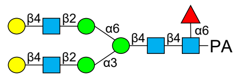	10.367	3.669	✓	
55.12	13.85	13.80	61.66	62.30	1035.19	1035.38	3H+	3103.13	N3H6Hn5dH1	trN-F-**TR**				✓	SLe^x
55.12	13.85		61.66		883.96	884.33	3H+	2649.98	N2H5Hn5dH1	66N-**BI**BsF(6)/epi				✓	
55.12	13.85		61.66		889.73	889.67	3H+	2665.97	N2H6Hn5	dN-**TR**	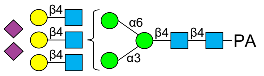	0.067	0.024		
55.12	13.85	13.35	61.66	60.99	1151.16	1151.43	2H+	2300.84	N2H5Hn4	63N-**BI**	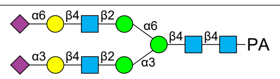	0.044	0.016		
55.12	13.85	13.72	61.66		997.73	997.88	2H+	1993.75	N1H4Hn4dH1	06N-**BI**F(6)-Ga2	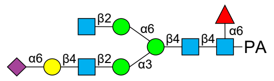	0.025	0.009		
55.46	13.97	13.80	61.99		1035.51	1035.38	3H+	3103.13	N3H6Hn5dH1	dN-3N-F(3)-**TR**	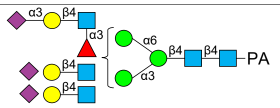	6.937	2.455	✓	SLe^x
55.46	13.97		61.99		883.94	884.33	3H+	2649.98	N2H5Hn5dH1	66N-**BI**BsF(6)/epi	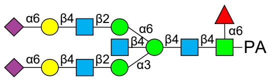	1.238	0.438	✓	
55.46	13.97	14.01	61.99		889.73	889.67	3H+	2665.97	N2H6Hn5	66N-Ln-**BI**	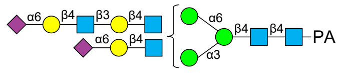	0.752	0.266	✓	N-dLn
55.46	13.97	13.61	61.99	60.83	933.50	933.36	2H+	1864.71	H5Hn4dH1	**BI**F(6)					
55.46	13.97	14.23	61.99	62.56	1151.94	1151.43	2H+	2300.84	N2H5Hn4	33N-**BI**	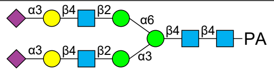	0.046	0.016		
56.65	14.43	14.52	63.16	62.25	1078.94	1078.91	2H+	2155.80	N1H5Hn4dH1	06N-**BI**F(6)	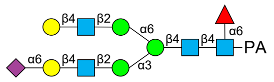	10.573	3.742	✓	
56.65	14.43	14.45	63.16		889.86	889.67	3H+	2665.97	N2H6Hn5	66N-Ln-**BI**	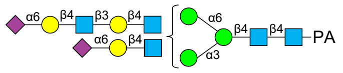	2.090	0.740	✓	N-dLn
56.65	14.43	14.36	63.16	63.69	1035.65	1035.38	3H+	3103.13	N3H6Hn5dH1	trN-F-**TR**	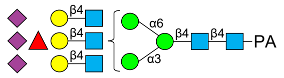	0.102	0.036		
57.85	14.91	14.96	64.34		1107.84	1107.42	2H+	2212.83	N1H5Hn5	06N-**BI**Bs	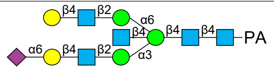	0.348	0.123	✓	
57.85	14.91	14.99	64.34	65.17	986.80	986.70	3H+	2957.07	N3H6Hn5	trN-**TR**				✓	
58.57	15.19	14.87	65.05	64.27	1224.51	1224.46	2H+	2446.90	N2H5Hn4dH1	66N-**BI**F(6)	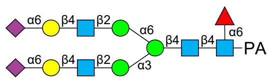	16.518	5.846	✓	
58.57	15.19	14.99	65.05	65.17	986.86	986.70	3H+	2957.07	N3H6Hn5	trN-**TR**	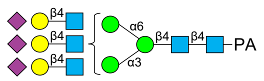	7.422	2.627	✓	
58.57	15.19		65.05		1014.18	1014.05	3H+	3039.12	N3H4Hn7	trN-dLdn-**MO**	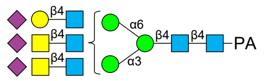	0.458	0.162		Ldn
59.83	15.71	15.57	66.29	66.49	986.85	986.70	3H+	2957.07	N3H6Hn5	trN-**TR**	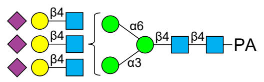	0.550	0.195	✓	
59.83	15.71	16.34	66.29		889.88	889.67	3H+	2665.97	N2H6Hn5	33N-Ln-**BI**	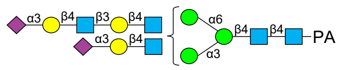	0.090	0.032		N-dLn
59.83	15.71	15.86	66.29	68.72	1079.05	1078.91	2H+	2155.80	N1H5Hn4dH1	60N-**BI**F(6)	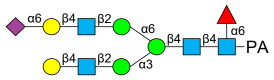	0.029	0.010		

S3 Table continued.

RT (min)	GU experi-mental	GU std.	RP experi-mental	RP std.	*m/z* experi-mental	*m/z* theoretical	*m/z* ion form	MS	Composition (+PA)	Glycan structure		Quantity ratio % for top	Quantity ratio % for whole	MS[2]	Note
59.83	15.71	16.30	66.29	67.84	1224.80	1224.46	2H+	2446.90	N2H5Hn4dH1	36N-**BI**F(6)	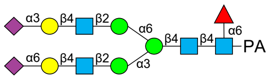	0.005	0.002		
61.53	16.43	16.73	67.94		961.74	961.87	2H+	1921.73	H5Hn5	**BI**Bs	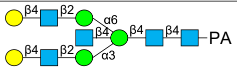	0.254	0.090		
62.84	17.01	16.96	69.13	69.36	986.83	986.70	3H+	2957.07	N3H6Hn5	trN-**TR**	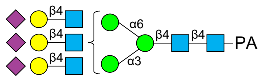	7.014	2.482	✓	
62.84	17.01	17.28	69.13		1078.88	1078.91	2H+	2155.80	N1H5Hn4dH1	03N-**BI**F(6)	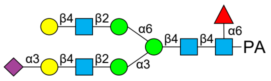	0.004	0.001		
62.84	17.01	17.43	69.13	70.68	1224.52	1224.46	2H+	2446.90	N2H5Hn4dH1	63N-**BI**F(6)	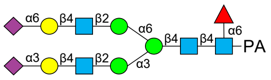	0.002	0.001		
62.84	17.01		69.13		938.40	938.35	3H+	2812.03	N2H6Hn4dH1	dN-**TR**F(6)	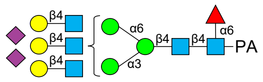	0.001	0.000		
63.94	17.51		70.12		986.78	986.70	3H+	2957.07	N3H6Hn5	trN-**TR**	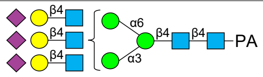	1.448	0.512		
65.37	18.19	18.39	71.42		889.83	889.67	3H+	2665.97	N2H6Hn5	dN-**TR**	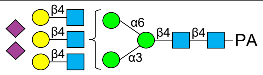	0.699	0.247		
65.37	18.19		71.42		1084.15	1084.07	3H+	3249.18	N3H6Hn5dH2	trN-dF-**TR**	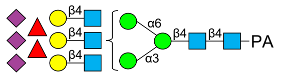	0.037	0.013		
67.03	19.03	19.64	72.92		938.45	938.35	3H+	2812.03	N2H6Hn4dH1	dN-**TR**F(6)	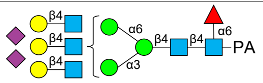	0.588	0.208		
68.50	19.82	19.43	74.25	73.91	1224.80	1224.46	2H+	2446.90	N2H5Hn4dH1	33N-**BI**F(6)	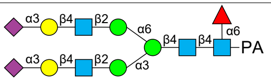	1.335	0.472		
68.50	19.82	20.35	74.25	75.07	1035.42	1035.38	3H+	3103.13	N3H6Hn5dH1	trN-F-**TR**	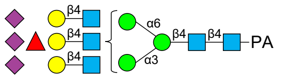	0.454	0.161		
68.50	19.82	20.65	74.25	74.28	1099.12	1099.42	2H+	2196.83	N1H4Hn5dH1	06N-**BI**BsF(6)-Ga2	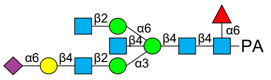	0.042	0.015		
69.47	20.37	20.29	75.13	75.27	873.18	872.85	2H+	1743.68	H3Hn5dH1	**AG12**BsF(6)	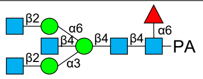	1.394	0.493	✓	
69.47	20.37	20.35	75.13		1035.65	1035.38	3H+	3103.13	N3H6Hn5dH1	trN-F-**TR**					
70.44	20.95		76.00		889.85	889.67	3H+	2665.97	N2H6Hn5	dN-**TR**	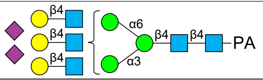	1.448	0.512		
72.25	22.11	21.99	77.64	77.61	1180.45	1180.45	2H+	2358.88	N1H5Hn5dH1	06N-**BI**BsF(6)	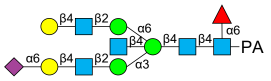	6.155	2.178	✓	
72.25	22.11	22.47	77.64		953.81	953.87	2H+	1905.73	H4Hn5dH1	**BI**BsF(6)-Ga2	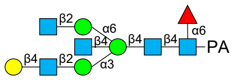	0.088	0.031	✓	
72.25	22.11	22.02	77.64	77.39	1035.55	1035.38	3H+	3103.13	N3H6Hn5dH1	trN-F-**TR**	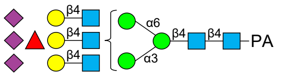	0.048	0.017		
73.13	22.72	23.29	78.44		884.53	884.33	3H+	2649.98	N2H5Hn5dH1	66N-**BI**BsF(6)	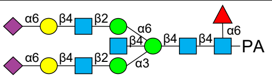	4.253	1.505	✓	
73.13	22.72	22.65	78.44		889.80	889.67	3H+	2665.97	N2H6Hn5	33N-Ln-**BI**F(6)	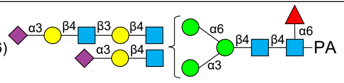	0.337	0.119		N-dLn
73.13	22.72	22.47	78.44		953.55	953.87	2H+	1905.73	H4Hn5dH1	**BI**BsF(6)-Ga2					
74.35	23.61	23.84	79.54	80.00	1035.29	1034.90	2H+	2067.79	H5Hn5dH1	**BI**BsF(6)	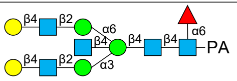	1.469	0.520	✓	
74.35	23.61	24.25	79.54		938.25	938.35	3H+	2812.03	N2H6Hn4dH1	dN-**TR**F(6)	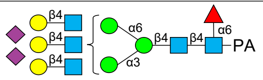	0.038	0.013		

S3 Table continued.

RT (min)	GU		RP		*m/z*			MS	Composition (+PA)	Glycan structure		Quantity ratio		MS^2	Note
	experi-mental	std.	experi-mental	std.	experi-mental	theoretical	ion form					% for top	% for whole		
75.92	24.86	25.85	80.96	81.49	1099.54	1099.42	2H+	2196.83	N1H4Hn5dH1	30N-**BI**BsF(6)-Ga1	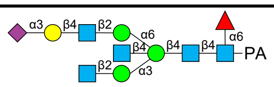	0.323	0.114		
77.36	26.13	27.33	82.26	83.11	1180.30	1180.45	2H+	2358.88	N1H5Hn5dH1	30N-**BI**BsF(6)	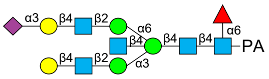	0.238	0.084		
78.26	26.98	26.16	83.07		1172.83	1172.43	2H+	2342.85	N2H5Hn4Ac1	Ac-66N-**BI**	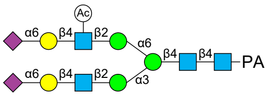	0.293	0.104		
83.00	32.42		87.36		1060.83	1060.06	3H+	3177.16	N2H7Hn6dH1	dN-**TE**F(6)	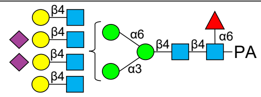	0.059	0.021		

S4 Table. *N*-glycans from human urine M1 analyzed using the improved method.

RT (min)	GU		RP		*m/z*			MS	Composition (+PA)		Glycan structure	Quantity ratio		MS^2	Only	Note
	experi-mental	std.	experi-mental	std.	experi-mental	theoretical	ion form					% for top	% for whole			
23.62	5.17	5.11	30.61	29.07	899.85	900.33	2H+	1798.65	H8Hn2	**M8A**	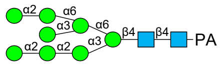	1.331	0.115	✓		
23.62	5.17		30.61		1186.09		2H+		N2H2Hn2+1059.16			1.010	0.087	✓		
24.81	5.36	5.31	31.75	30.36	818.75	819.31	2H+	1636.60	H7Hn2	**M7A**	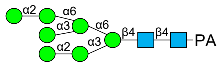	2.354	0.204	✓		
25.30	5.44		32.23		1040.79	1041.89	2H+	2081.77	N2H4Hn3dH1	dN-F-Ln-**M2'**	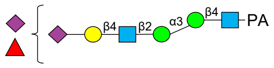	0.281	0.024			
26.04	5.57	5.50	32.94	31.33	980.83	981.36	2H+	1960.70	H9Hn2	**M9A**	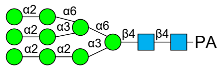	11.664	1.009	✓		
26.04	5.57		32.94		1186.02		2H+					4.370	0.378			
26.83	5.70		33.70		1231.35	1231.45	H+	1230.44	H7	**Glc7**	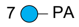	0.544	0.047	✓		
27.47	5.82	5.76	34.32	32.76	899.39	900.33	2H+	1798.65	H8Hn2	**M8B**	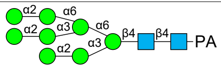	0.439	0.038	✓		
28.13	5.94	5.88	34.95	33.44	818.97	819.31	2H+	1636.60	H7Hn2	**M7B**	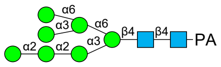	12.973	1.122	✓		
28.13	5.94		34.95		1231.42	1231.45	H+	1230.44	H7	**Glc7**	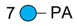	0.393	0.034	✓		
28.13	5.94		34.95		1106.51	1107.42	2H+	2212.83	N1H5Hn5	N-**BI**Bs/ga	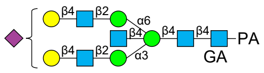					
29.30	6.16	6.11	36.08	34.37	737.81	738.28	2H+	1474.54	H6Hn2	**M6B**	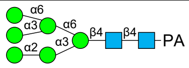	52.975	4.580	✓		
30.94	6.49	6.48	31.75		899.90	900.33	2H+	1798.65	H8Hn2	**M8C**	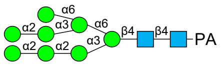	0.445	0.038			
31.92	6.69	6.72	38.60		1004.70	1005.88	2H+	2009.75	N1H5Hn4	06N-**BI**/ga	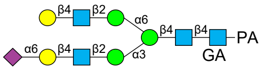			✓		
32.68	6.86		37.66		1005.32	1005.88	2H+	2009.75	N1H5Hn4	06N-**BI**/epi	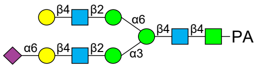	0.247	0.021			
32.68	6.86		37.66		1135.33	1135.45	H+	1134.44	H3Hn2dH1	**M3B**F(6)/epi	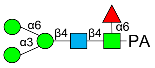	0.163	0.014			
32.68	6.86	6.80	37.66	37.73	818.59	819.31	2H+	1636.60	H7Hn2	**M7D**	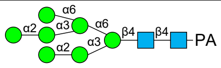	0.130	0.011			
32.68	6.86	7.08	37.66	38.72	989.26	989.39	H+	988.39	H3Hn2	**M3B**						
33.35	7.01	7.00	40.00	38.72	1313.32	1313.50	H+	1312.49	H5Hn2	**M5A**	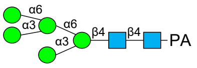	17.646	1.526	✓		
33.35	7.01	7.08	40.00	38.72	989.30	989.39	H+	988.39	H3Hn2	**M3B**	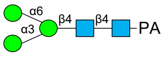	6.831	0.591			
33.35	7.01		40.00		971.24							6.114	0.529			
33.35	7.01	7.04	40.00	38.72	827.29	827.34	H+	826.33	H2Hn2	**M2B**	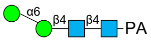	5.809	0.502	✓		
33.35	7.01		40.00		1133.26	1135.45	H+	1134.44	H3Hn2dH1	**M3B**F(6)/ga	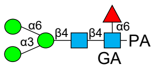					
33.35	7.01	6.72	40.00		1151.30	1151.45	H+	1150.44	H4Hn2	**M4A**	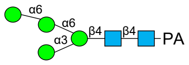	0.554	0.048			
34.04	7.16	7.06	40.73		1151.40	1151.45	H+	1150.44	H4Hn2	**M4B**	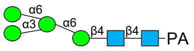	0.599	0.052	✓		
34.04	7.16		40.73		1004.58	1005.88	2H+	2009.75	N1H5Hn4	30N-**BI**/ga	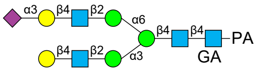			✓		
34.04	7.16		40.73		1313.34	1313.50	H+	1312.49	H5Hn2	**M5A**		0.055	0.005			
34.04	7.16	7.00	40.73		1133.23	1135.45	H+	1134.44	H3Hn2dH1	**M3B**F(6)/ga						
35.42	7.49		42.18		1150.26	1151.43	2H+	2300.84	N2H5Hn4	66N-**BI**/ga						
35.42	7.49		42.18		1004.61	1005.88	2H+	2009.75	N1H5Hn4	30N-**BI**/ga						
35.86	7.60	7.56	42.65		1150.92	1151.43	2H+	2300.84	N2H5Hn4	66N-**BI**/ga	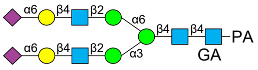			✓		

S4 Table continued.

RT (min)	GU experi-mental	GU std.	RP experi-mental	RP std.	m/z experi-mental	m/z theoretical	ion form	MS	Composition (+PA)		Glycan structure	Quantity ratio % for top	Quantity ratio % for whole	MS²	Only	Note
37.06	7.90	7.88	43.92		1151.33	1151.43	2H+	2300.84	N2H5Hn4	66N-**BI**/epi	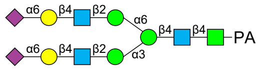	2.744	0.237	✓		
37.58	8.04		44.47		1151.28	1151.43	2H+	2300.84	N2H5Hn4	36N-**BI**/epi	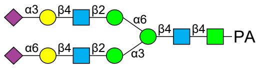	7.989	0.691	✓		
37.58	8.04	8.03	44.47		985.29	985.37	2H+	1968.72	N1H6Hn3	6N-Ln-**M5A**	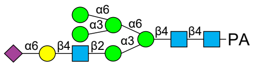	6.463	0.559	✓		
37.58	8.04	8.09	44.47	42.84	823.33	823.31	2H+	1644.61	N1H4Hn3	6N-**MO1**	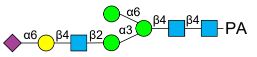	5.508	0.476	✓		
37.58	8.04		44.47		1179.28	1179.41	2H+	2356.80	P1N1H7Hn3dH1	6P-N-Ln-**M6F**(6)	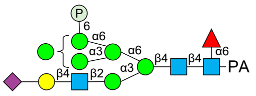	3.705	0.320			
38.16	8.19	8.30	45.08	43.68	1151.50	1151.43	2H+	2300.84	N2H5Hn4	36N-**BI**/ga	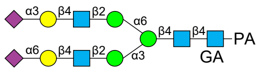					
38.16	8.19		45.08		1078.76	1078.91	2H+	2155.80	N1H5Hn4dH1	3N-F(3)-**BI**	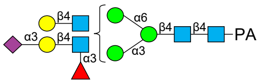	0.659	0.057	✓		SLe^{x}
38.16	8.19	8.25	45.08		904.40	904.34	2H+	1806.67	N1H5Hn3	6N-Ln-**M4C**	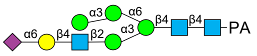	0.622	0.054	✓		
39.51	8.55		46.50		1150.49	1151.43	2H+	2300.84	N2H5Hn4	dN-**BI**/ga	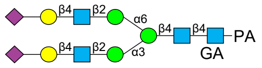					
39.51	8.55		46.50		895.14	895.34	2H+	1790.67	N1H4Hn3dH1	N-**MOF**(6)	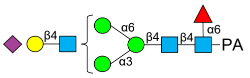	0.163	0.014			
39.51	8.55		46.50		1179.99	1179.41	2H+	2356.80	P1N1H7Hn3dH1	6P-N-Ln-**M6F**(6)	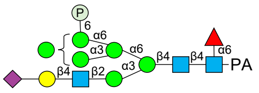	0.105	0.009			
39.65	8.59		46.65		1150.59	1151.43	2H+	2300.84	N2H5Hn4	dN-**BI**/ga	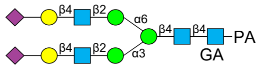			✓		
39.65	8.59	8.64	46.65		1079.11	1079.42	2H+	2156.82	H5Hn4dH3	F(2)-F(3)-**BIF**(6)	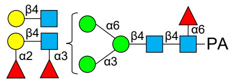	0.632	0.055			Le^{y}
39.65	8.59		46.65		1179.79	1179.41	2H+	2356.80	P1N1H7Hn3dH1	6P-N-Ln-**M6F**(6)	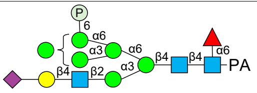	0.385	0.033			
40.44	8.82		47.49		1332.88	1333.99	2H+	2665.97	N2H6Hn5	dN-**TR**/ga	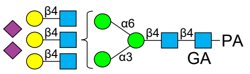					
40.44	8.82	9.13	47.49		1151.17	1151.43	2H+	2300.84	N2H5Hn4	33N-**BI**/epi						
41.48	9.12	9.13	48.58		1151.97	1151.43	2H+	2300.84	N2H5Hn4	33N-**BI**/epi	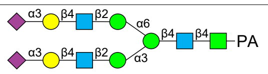	0.890	0.000			
41.48	9.12		48.58		851.12	851.33	2H+	1700.64	H4Hn4dH1	**BI**F(6)-Ga/epi	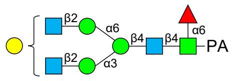	0.169	0.015			
41.48	9.12		48.58		811.30	811.35	H+	810.34	H1Hn2dH1	**M1A**F(6)	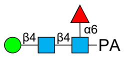	0.164	0.014	✓		
41.48	9.12		48.58		1333.52	1333.99	2H+	2665.97	N2H6Hn5	dN-**TR**						
42.07	9.29		49.21		697.81	697.25	2H+	1392.49	H8	**Glc8**	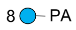	0.124	0.011			
42.07	9.29		49.21		1333.50	1333.99	2H+	2665.97	N2H6Hn5	dN-**TR**	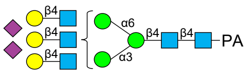	0.225	0.019			
42.07	9.29		49.21		932.55	933.36	2H+	1864.71	H5Hn4dH1	**BI**F(6)/ga	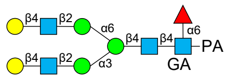					
42.07	9.29		49.21		1260.25							0.082	0.007		✓	
42.07	9.29	9.56	49.21		1192.31	1192.47	H+	1191.47	H3Hn3	**AG2**	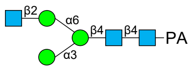	0.077	0.007	✓		

S4 Table continued.

RT (min)	GU experi-mental	GU std.	RP experi-mental	RP std.	m/z experi-mental	m/z theoretical	m/z ion form	MS	Composition (+PA)	Glycan structure	Quantity ratio % for top	Quantity ratio % for whole	MS²	Only	Note
42.07	9.29		49.21		1204.59	1205.44	3H+	3613.30	N4H7Hn6	teN-**TE**/ga 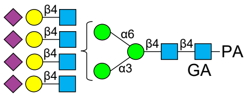	0.075	0.007		✓	
43.15	9.61		50.35		1478.09	1479.54	2H+	2957.07	N3H6Hn5	trN-F-**TR**/ga					
43.15	9.61	9.63	50.35	48.64	924.71	924.85	2H+	1847.69	N1H4Hn4	06N-**BI**-Ga2 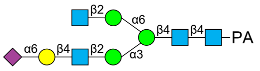	2.049	0.177			
43.15	9.61		50.35		1296.38	1297.49	2H+	2592.96	N2H5Hn4dH2	dN-F-**BI**F(6)/ga					
43.69	9.78		50.92		1296.54	1297.49	2H+	2592.96	N2H5Hn4dH2	3N-N-F(3)-**BI**F(6)/ga 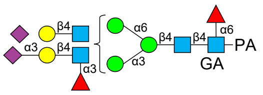			✓		SLe^x
43.69	9.78		50.92		1478.88	1479.54	2H+	2957.07	N3H6Hn5	trN-F-**TR**/ga 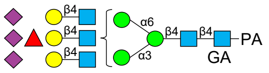					
43.69	9.78		50.92		1026.28	1026.39	2H+	2050.77	N1H4Hn5	N-**BI**Bs-Ga 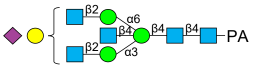					
43.69	9.78		50.92		985.92	986.70	3H+	2957.07	N3H6Hn5	trN-**TR**/ga 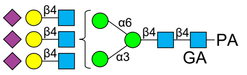					
43.69	9.78	9.63	50.92	48.64	924.71	924.85	2H+	1847.69	N1H4Hn4	06N-**BI**-Ga2					
44.18	9.93	10.05	51.43		860.32	860.33	2H+	1718.65	H5Hn4	**BI** 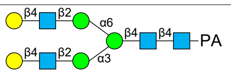	2.366	0.205	✓		
44.18	9.93	10.07	51.43		1224.46	1224.46	2H+	2446.90	N2H5Hn4dH1	N-3N-F(3)-**BI** 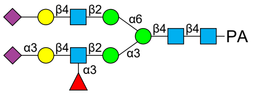	1.254	0.108			SLe^x
44.18	9.93		51.43		1135.29	1135.45	H+	1134.44	H3Hn2dH1	**M3B**F(6)					
44.18	9.93		51.43	50.51	973.41	973.40	H+	972.39	H2Hn2dH1	**M2B**F(6)					
44.86	10.15	10.07	52.15	50.51	973.42	973.40	H+	972.39	H2Hn2dH1	**M2B**F(6) 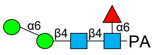	38.161	3.300	✓		
44.86	10.15		52.15		1077.94	1078.91	2H+	2155.80	N1H5Hn4dH1	06N-**BI**F(6)/ga 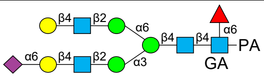					
44.86	10.15		52.15	50.87	1005.94	1005.88	2H+	2009.75	N1H5Hn4	06N-**BI** 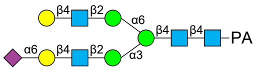	15.548	1.344	✓		
44.86	10.15	10.07	52.15	50.51	1135.44	1135.45	H+	1134.44	H3Hn2dH1	**M3B**F(6) 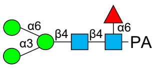	15.012	1.298			
45.95	10.50	10.55	53.25		1223.94	1224.97	2H+	2446.90	N2H5Hn4dH1	66N-**BI**F(6)/ga 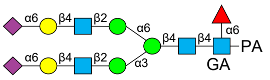			✓		
47.05	10.86		54.3579		1077.80	1078.91	2H+	2155.80	N1H5Hn4dH1	N-**BI**F(6)/ga 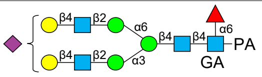			✓		
47.05	10.86		54.36		1243.7	1243.46	3H+	3727.36	N4H5Hn6dH3	teN-dF-Ldn-**BI**F(6) 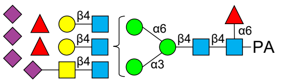	0.376	0.033	✓		N-Ldn,
47.05	10.86	10.91	54.36		1223.23	1224.46	2H+	2446.90	N2H5Hn4dH1	36N-**BI**F(6)/ga			✓		
47.05	10.86		54.36		1119.84	1119.94	2H+	2237.86	N1H3Hn6dH1	N-dLdn-**M3**F(6) 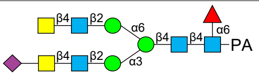	0.240	0.021	✓		N-Ldn
47.58	11.04	10.91	54.89		1223.91	1224.46	2H+	2446.90	N2H5Hn4dH1	36N-**BI**F(6)/ga 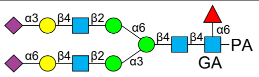			✓		
47.58	11.04	11.15	54.89		1077.85	1078.91	2H+	2155.80	N1H5Hn4dH1	03N-**BI**F(6)/ga 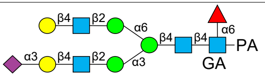					
47.58	11.04	11.73	54.89		1261.06	1261.48	2H+	2520.94	N1H6Hn5dH1	N-**TR**F(6) 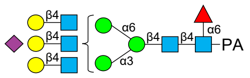	6.523	0.564	✓		
47.58	11.04		54.89		1180.84	1180.96	2H+	2359.90	H5Hn5dH3	F(2)-F(3)-**BI**BsF(6) 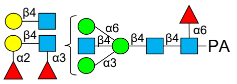	4.215	0.364			Le^y

S4 Table continued.

RT (min)	GU experi-mental	GU std.	RP experi-mental	RP std.	*m/z* experi-mental	*m/z* theoretical	ion form	MS	Composition (+PA)		Glycan structure	Quantity ratio % for top	Quantity ratio % for whole	MS^2	Only	Note
47.58	11.04	10.91	54.89		1034.92	1034.90	2H+	2067.79	H5Hn5dH1	F-**BI**Bs	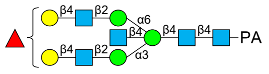	1.184	0.102			
48.15	11.24	11.49	55.47	54.54	1151.75	1151.43	2H+	2300.84	N2H5Hn4	66N-**BI**		0.190	0.016	✓		
48.15	11.24	11.65	55.47		1223.56	1224.46	2H+	2446.90	N2H5Hn4dH1	33N-**BI**F(6)/ga	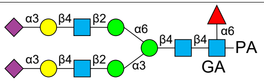			✓		
48.15	11.24		55.47		1180.59	1180.96	2H+	2359.90	H5Hn5dH3	F(2)-F(3)-**BI**BsF(6)	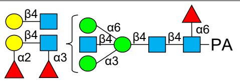	0.106	0.009	-		Le^y
48.15	11.24		55.47		1077.62	1078.91	2H+	2155.80	N1H5Hn4dH1	66N-**BI**F(6)/ga				✓		
48.15	11.24	11.12	55.47		1034.76	1034.90	2H+	2067.79	H5Hn5dH1	**BI**BsF(6)	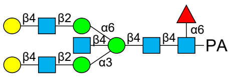	0.044	0.004			
48.15	11.24	11.33	55.47	55.68	916.97	916.86	2H+	1831.70	N1H3Hn4dH1	N-Ldn1-**M3**F(6)	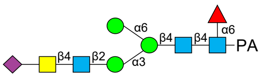	0.029	0.003			N-Ldn
48.68	11.42	11.49	56.00	54.54	1151.56	1151.43	2H+	2300.84	N2H5Hn4	66N-**BI**	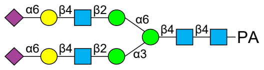	100	8.647	✓		
49.52	11.71	11.65	56.85		1223.85	1224.46	2H+	2446.90	N2H5Hn4dH1	33N-**BI**F(6)/ga	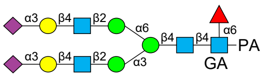			✓		
49.52	11.71	11.98	56.85	55.95	1005.84	1005.88	2H+	2009.75	N1H5Hn4	30N-**BI**	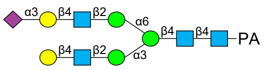	1.644	0.142	✓		
49.52	11.71	12.02	56.85	56.14	1151.85	1151.43	2H+	2300.84	N2H5Hn4	66N-**BI**				✓		
50.26	11.98	12.02	57.59	56.14	1151.32	1151.43	2H+	2300.84	N2H5Hn4	36N-**BI**	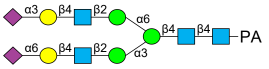	14.234	1.231	✓		
50.26	11.98	11.65	57.59		1223.21	1224.46	2H+	2446.90	N2H5Hn4dH1	33N-**BI**F(6)/ga	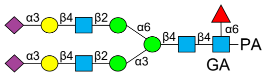			✓		
50.26	11.98	11.98	57.59	55.95	1005.51	1005.88	2H+	2009.75	N1H5Hn4	30N-**BI**	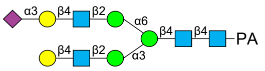	2.225	0.192	✓		
50.26	11.98	12.08	57.59	57.74	1205.30	1205.44	3H+	3613.30	N4H7Hn6	teN-**TE**	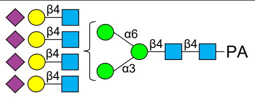	1.247	0.108		✓	
50.26	11.98	12.00	57.59	56.09	771.36	771.31	2H+	1540.60	H3Hn4dH1	**AG12**F(6)	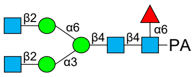	0.639	0.055			
50.26	11.98	11.68	57.59	59.36	1338.36	1338.53	H+	1337.52	H3Hn3dH1	**AG2**F(6)	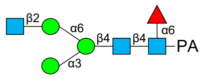	0.471	0.041			
51.87	12.56		59.21		1253.42	1253.48	2H+	2504.94	N1H5Hn5dH2	N-F-Gn-Ln-**M4**F(6)	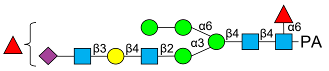	1.581	0.137	✓		
51.87	12.56		59.21		1405.91	1407.02	2H+	2812.03	N2H6Hn4dH1	dN-F-**TR**				✓		
51.87	12.56	12.59	59.21		1188.19	1188.45	2H+	2374.88	N1H6Hn5	N-**TR**	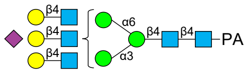	0.208	0.018			
51.87	12.56		59.21		1551.50	1552.57	2H+	3103.13	N3H6Hn5dH1	trN-F-**TR**	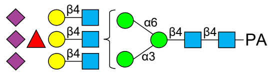	0.084	0.007			
51.87	12.56		59.21		938.14	938.35	3H+	2812.03	N2H6Hn4dH1	dN-**TR**F(6)	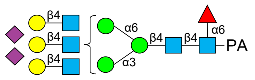	0.020	0.002		✓	
52.21	12.69		59.56		1406.69	1407.02	2H+	2812.03	N2H6Hn4dH1	dN-F-**TR**	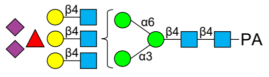	4.374	0.378	✓		N-dLn, Lex
52.21	12.69		59.56		1224.38	1224.46	2H+	2446.90	N2H5Hn4dH1	33N-**BI**F(6)/epi	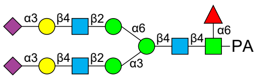	3.584	0.310	✓		
52.21	12.69	13.11	59.56	60.83	852.12	852.33	2H+	1702.66	H4Hn4dH1	**BI**F(6)-Ga2	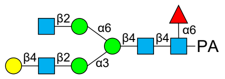	3.075	0.266	✓		
52.21	12.69	13.08	59.56	60.45	1551.83	1552.57	2H+	3103.13	N3H6Hn5dH1	trN-F-**TR**						

S4 Table continued.

RT (min)	GU experi-mental	GU std.	RP experi-mental	RP std.	m/z experi-mental	m/z theoretical	m/z ion form	MS	Composition (+PA)	Glycan structure		Quantity ratio % for top	Quantity ratio % for whole	MS^2	Only	Note
52.21	12.69		59.56		1156.37	1157.09	3H+	3468.26	N3H7Hn6dH1	trN-F-**TE**	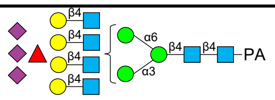	1.210	0.105		✓	
52.82	12.92	13.08	60.17	60.45	1551.82	1552.57	2H+	3103.13	N3H6Hn5dH1	trN-F-**TR**	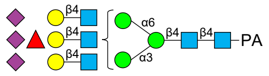	2.797	0.242	-		
52.82	12.92		60.17		1405.86	1407.02	2H+	2812.03	N2H6Hn4dH1	dN-F-**TR**/ga	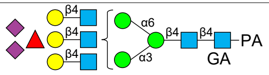					
52.82	12.92		60.17		1034.53	1034.90	2H+	2067.79	H5Hn5dH1	Gn-**BI**F(6)	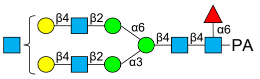	0.328	0.028			
52.82	12.92	13.35	60.17		1151.16	1151.43	2H+	2300.84	N2H5Hn4	63N-**BI**	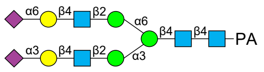	0.238	0.021			
54.54	13.59	13.61	61.91	60.83	933.21	933.36	2H+	1864.71	H5Hn4dH1	**BI**F(6)	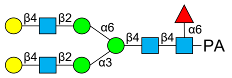	4.996	0.432	✓		
54.54	13.59	13.59	61.91	61.78	1205.68	1205.44	3H+	3613.30	N4H7Hn6	teN-**TE**	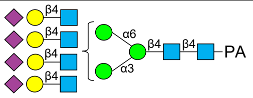	4.641	0.401		✓	
54.54	13.59	13.37	61.91		1297.10	1297.49	2H+	2592.96	N2H5Hn4dH2	dN-F(3)2-**BI**F(6)	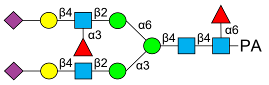	3.117	0.270	✓		SLe^x
54.54	13.59	14.01	61.91		1333.90	1333.99	2H+	2665.97	N2H6Hn5	dN-Ln-**BI**	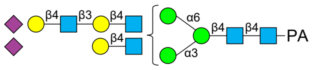	2.931	0.253	-		
54.54	13.59		61.91		1099.39	1099.42	2H+	2196.83	N1H4Hn5dH1	N-F-Ldn-**MO**	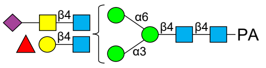	7.152	0.618	✓		N-Ldn
54.54	13.59	13.35	61.91		1151.24	1151.43	2H+	2300.84	N2H5Hn4	63N-**BI**	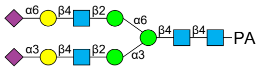	1.662	0.144			
54.54	13.59		61.91		1406.52	1407.02	2H+	2812.03	N2H6Hn4dH1	dN-Ln-**BI**F(6)	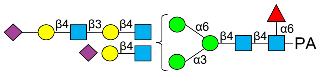	1.326	0.115			N-dLn
54.54	13.59	13.72	61.91		997.96	997.88	2H+	1993.75	N1H4Hn4dH1	06N-**BI**F(6)-Ga2	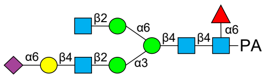	0.899	0.078			
54.97	13.76	14.01	62.34		1333.86	1333.99	2H+	2665.97	N2H6Hn5	dN-Ln-**BI**	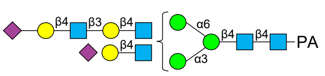	37.937	3.280	✓		N-dLn
54.97	13.76	13.80	62.34		1552.26	1552.57	2H+	3103.13	N3H6Hn5dH1	3N-dN-F(3)-**TR**	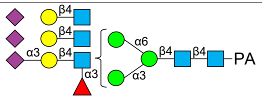	12.538	1.084	✓		SLe^x
54.97	13.76		62.34		1099.50	1099.42	2H+	2196.83	N1H4Hn5dH1	N-Ldn-**MO**F(6)				✓		N-Ldn
54.97	13.76		62.34		1369.85	1370.00	2H+	2737.99	N3H5Hn4dH1	6N-3N-N-**BI**F(6)	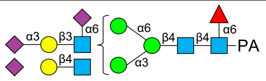	2.518	0.218	✓		dN-Ln
54.97	13.76		62.34		1205.48	1205.44	3H+	3613.30	N4H7Hn6	teN-**TE**						
55.90	14.14	14.12	63.28	62.25	1078.63	1078.91	2H+	2155.80	N1H5Hn4dH1	06N-**BI**F(6)	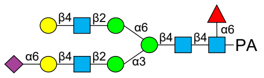	56.730	4.905	✓		
55.90	14.14		63.28		1334.32	1333.99	2H+	2665.97	N2H6Hn5	dN-**TR**	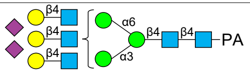	4.235	0.366			
55.90	14.14	14.23	63.28	62.56	1151.27	1151.43	2H+	2300.84	N2H5Hn4	33N-**BI**	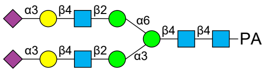	3.492	0.302	✓		
55.90	14.14		63.28		1179.32	1180.45	2H+	2358.88	N1H5Hn5dH1	06N-**BI**BsF(6)/ga	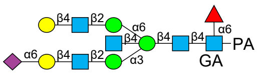	2.268	0.196			
55.90	14.14		63.28		1033.94	1035.38	3H+	3103.13	N3H6Hn5dH1	trN-**TR**F(6)/ga	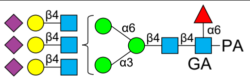					
56.95	14.57	14.99	64.33	65.17	1479.36	1479.54	2H+	2957.07	N3H6Hn5	trN-**TR**	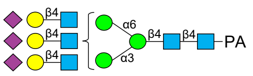	23.657	2.046			

S4 Table continued.

RT (min)	GU experi-mental	GU std.	RP experi-mental	RP std.	*m/z* experi-mental	*m/z* theoretical	*m/z* ion form	MS	Composition (+PA)	Glycan structure		Quantity ratio % for top	Quantity ratio % for whole	MS^2	Only	Note
56.95	14.57		64.33		1552.36	1552.57	2H+	3103.13	N3H6Hn5dH1	trN-F-**TR**	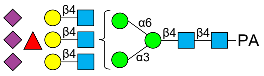	16.054	1.388			
56.95	14.57	14.48	64.33		1026.53	1026.90	2H+	2051.79	H4Hn5dH2	F-**BI**BsF(6)-Ga	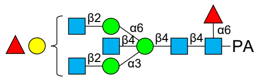	9.230	0.798			
56.95	14.57		64.33		1244.56	1244.97	2H+	2487.93	N2H4Hn5dH1	dN-Ldn-**MO**F(6)	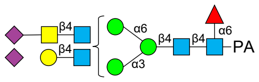	5.703	0.493			N-Ldn
56.95	14.57	14.96	64.33		1107.17	1107.42	2H+	2212.83	N1H5Hn5	06N-**BI**Bs	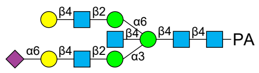	4.351	0.376			
56.95	14.57	14.87	64.33	64.27	1224.36	1224.46	2H+	2446.90	N2H5Hn4dH1	66N-**BI**F(6)						
58.11	15.07	14.87	65.50	64.27	1224.56	1224.46	2H+	2446.90	N2H5Hn4dH1	66N-**BI**F(6)	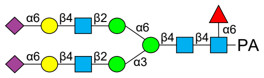	92.033	7.958	✓		
58.11	15.07	14.79	65.50		1119.90	1119.94	2H+	2237.86	N1H3Hn6dH1	06N-dLdn12-**M3**F(6)	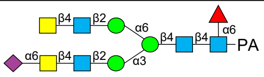	7.261	0.628	✓		N-Ldn
58.11	15.07		65.50		1629.32							4.148	0.359		✓	
58.11	15.07	15.35	65.50		1138.97	1139.40	2H+	2276.79	S1N1H4 Hn5dH1	S-N-Ldn1-**MO2**F(6)	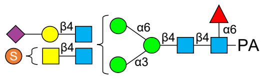	3.930	0.340			S-Ldn
58.11	16.07		65.50		1054.28	1054.34	2H+	2106.67	S2H3Hn6dH1	dS-dLdn-**M3**F(6)		1.023	0.088			
58.86	15.40	15.57	66.26	66.49	1479.44	1479.54	2H+	2957.07	N3H6Hn5	trN-Ln-**BI**	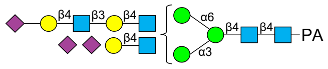	7.885	0.682	✓		N-dLn
58.86	15.40	15.86	66.26	65.53	1078.76	1078.91	2H+	2155.80	N1H5Hn4dH1	60N-**BI**F(6)	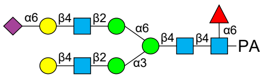	3.832	0.331	✓		
58.86	15.40		66.26		974.12	974.39	2H+	1946.76	H3Hn6dH1	dLdn12-**M3**F(6)	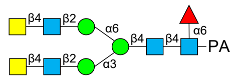	2.128	0.184			Ldn
58.86	15.40	14.87	66.26	64.27	1224.39	1224.46	2H+	2446.90	N2H5Hn4dH1	66N-**BI**F(6)						
58.86	15.40		66.26		1054.07	1054.34	2H+	2106.67	S2H3Hn6dH1	(dS-dLdn)-**M3**F(6)	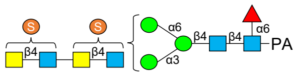	2.202	0.190			S-Ldn
58.86	15.40		66.26		1244.82	1244.97	2H+	2487.93	N2H4Hn5dH1	36N-Ldn2-**MO1**F(6)						
59.66	15.76	15.48	67.06		1245.20	1244.97	2H+	2487.93	N2H4Hn5dH1	36N-Ldn2-**MO1**F(6)	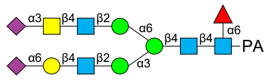	2.567	0.222	✓		N-Ldn
59.66	15.76	15.85	67.06		1156.95	1157.09	3H+	3468.26	N3Hn7H6dH1	trN-**TE**F(6)	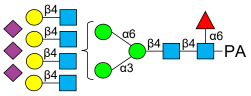	0.495	0.043			
59.66	15.76	16.73	67.06		961.86	961.87	2H+	1921.73	H5Hn5	**BI**Bs	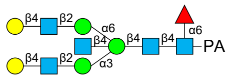	0.037	0.003	✓		
60.98	16.38		68.36		1224.26	1224.46	2H+	2446.90	N2H5Hn4dH1	36N-**BI**F(6)				✓		
60.98	16.38	16.26	68.36		1107.80	1107.93	2H+	2213.85	H5Hn5dH2	F(3)-**BI**BsF(6)	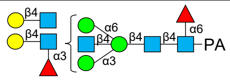	0.290	0.025			Le^x
60.98	16.38		68.36		1261.45	1261.48	2H+	2520.94	N1H6Hn5dH1	N-Ln-**BI**F(6)	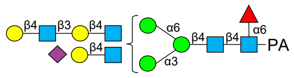	0.268	0.023	✓	✓	dLn
60.98	16.38	16.73	68.36		961.86	961.87	2H+	1921.73	H5Hn5	**BI**Bs	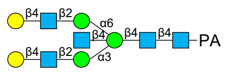	0.178	0.015	✓		
60.98	16.38		68.36		1157.24	1157.09	3H+	3468.26	N3Hn7H6dH1	trN-**TE**F(6)	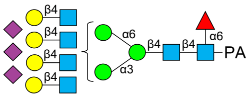	0.142	0.012		✓	
60.98	16.38		68.36		1552.45	1552.57	2H+	3103.13	N3H6Hn5dH1	trN-F-**TR**	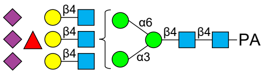	0.122	0.011		✓	
61.48	16.62	16.30	68.84	67.84	1224.43	1224.46	2H+	2446.90	N2H5Hn4dH1	36N-**BI**F(6)	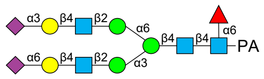	39.709	3.433	✓		

S4 Table continued.

RT (min)	GU		RP		*m/z*			MS	Composition (+PA)	Glycan structure	Quantity ratio		MS²	Only	Note
	experi-mental	std.	experi-mental	std.	experi-mental	theoretical	ion form				% for top	% for whole			
61.48	16.62	17.28	68.84		1078.54	1078.91	2H+	2155.80	N1H5Hn4dH1	03N-**BIF**(6) 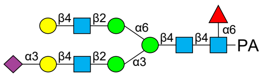	13.644	1.180	✓		
61.48	16.62		68.84		1254.22	1254.13	3H+	3759.35	N4H7Hn6dH1	trN-F-**TE** 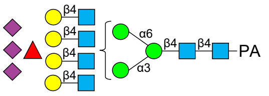	4.059	0.351	✓	✓	
61.48	16.62	16.96	68.84		1479.51	1479.54	2H+	2957.07	N3H6Hn5	trN-**TR** 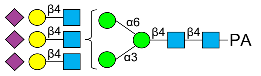	2.556	0.221			
62.23	16.99	17.66	69.56	68.72	1078.82	1078.91	2H+	2155.80	N1H5Hn4dH1	30N-**BIF**(6) 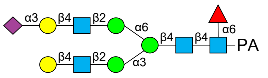	1.961	0.170	✓		
62.23	16.99		69.56		1325.45	1326.00	2H+	2649.98	N2H5Hn5dH1	dN-Gn-**BIF**(6) 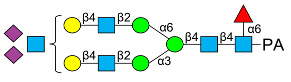	0.680	0.059			
62.23	16.99	17.43	69.56		1224.25	1224.46	2H+	2446.90	N2H5Hn4dH1	36N-**BIF**(6)			✓		
63.38	17.57		70.66		1261.30	1261.48	2H+	2520.94	N1H6Hn5dH1	N-**TRF**(6) 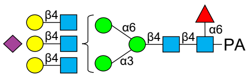	3.316	0.287			
63.38	17.57		70.66		1321.25	1321.82	3H+	3962.43	N4H7Hn7dH1	teN-**TEBs**F(6) 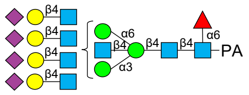	1.983	0.172		✓	
63.38	17.57		70.66		1156.94	1157.43	3H+	3469.28	N2H7Hn6dH3	dN-trF-**TE** 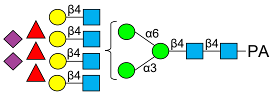	1.621	0.140		✓	
63.38	17.57	17.70	70.66		1735.42	1735.14	2H+	3468.26	N3Hn7H6dH1	trN-**TEF**(6) 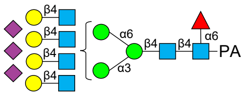	1.070	0.092		✓	
63.38	17.57		70.66		1099.15	1099.42	2H+	2196.83	N1H4Hn5dH1	N-Ldn-**MOF**(6) 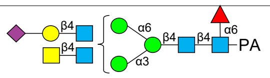	0.853	0.074	✓		Ldn
63.38	17.57	17.43	70.66	70.68	1224.05	1224.46	2H+	2446.90	N2H5Hn4dH1	63N-**BIF**(6) 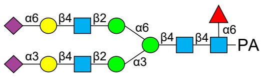	0.435	0.038			
64.89	18.38		72.11		1253.84	1254.13	3H+	3759.35	N4H7Hn6dH1	6N-3N-3N-N-F(3)-Ln-**TR** 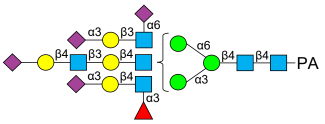	42.161	3.645	✓		N-dLn, dN-Ln, SLe^x
64.89	18.38		72.11		1157.36	1157.43	3H+	3469.28	N2H7Hn6dH3	dN-trF-**TE** 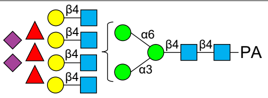	6.957	0.602			
64.89	18.38		72.11		1151.82	1151.94	2H+	2301.86	N1H5Hn4dH2	3N-F(3)-**BIF**(6) 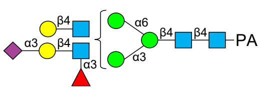	3.980	0.344	✓		SLe^x
66.09	19.06		73.26		1157.35	1157.43	3H+	3469.28	N3Hn7H6dH1	trN-**TEF**(6) 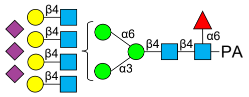	0.867	0.075		✓	
66.09	19.06		73.26		1406.93	1407.02	2H+	2812.03	N2H6Hn4dH1	dN-**TRF**(6) 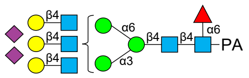	0.703	0.061			
66.09	19.06		73.26		1479.57	1479.54	2H+	2957.07	N3H6Hn5	trN-**TR** 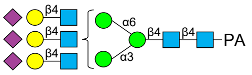	0.578	0.050		✓	
66.09	19.06		73.26		1289.17	1289.99	2H+	2577.96	N1H6Hn6	N-**TRBs** 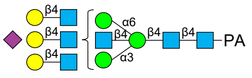	0.210	0.018			
66.09	19.06		73.26		1099.10	1099.42	2H+	2196.83	N1H4Hn5dH1	N-Ldn-**MOF**(6) 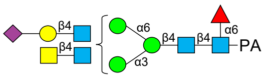	0.075	0.006			Ldn
67.03	19.62	19.43	74.17	73.91	1224.78	1224.46	2H+	2446.90	N2H5Hn4dH1	33N-**BIF**(6) 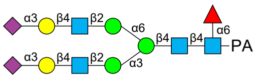	38.470	3.326	✓		

S4 Table continued.

RT (min)	GU experi-mental	GU std.	RP experi-mental	RP std.	*m/z* experi-mental	*m/z* theoretical	ion form	MS	Composition (+PA)		Glycan structure	Quantity ratio % for top	Quantity ratio % for whole	MS^2	Only	Note
67.03	19.62		74.17		1406.76	1407.02	2H+	2812.03	N2H6Hn5dH1	dN-**TRF**(6)	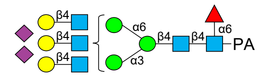	13.460	1.164	✓		
67.03	19.62		74.17		1552.71	1552.57	2H+	3103.13	N3H6Hn5dH1	trN-**TRF**(6)	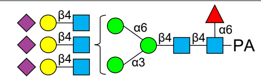	5.054	0.437			
67.03	19.62		74.17		1099.1	1099.42	2H+	2196.83	N1H4Hn5dH1	N-Ldn-**MOF**(6)	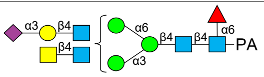	0.657	0.057		✓	Ldn
67.03	19.62		74.17		1216.62							0.270	0.023		✓	
68.46	20.52	21.99	75.54	77.61	872.83	872.85	2H+	1743.68	H3Hn5dH1	**AG12**BsF(6)	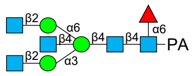	1.865	0.161	✓		
68.46	20.52		75.54		1407.04	1407.02	2H+	2812.03	N2H6Hn5dH1	dN-**TRF**(6)	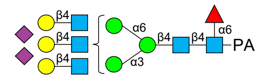	0.816	0.071		✓	
68.46	20.52		75.54		1216.08							0.528	0.046			
69.08	20.93		76.13		1381.14							1.444	0.125		✓	
69.08	20.93		76.13		1406.67	1407.02	2H+	2812.03	N2H6Hn5dH1	dN-**TRF**(6)	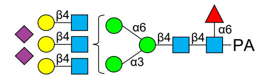	1.370	0.118		✓	
69.08	20.93		76.13		1170.69	1170.77	3H+	3509.29	N3H6Hn7dH1	trN-**TE**BsF(6)-Ga	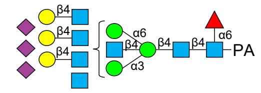	0.565	0.049		✓	
69.95	21.52	20.35	76.97		1552.38	1552.57	2H+	3103.13	N3H6Hn5dH1	trN-**TRF**(6)	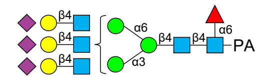	25.760	2.227	✓		
71.41	22.59	21.99	78.37	77.61	1180.35	1180.45	2H+	2358.88	N1H5Hn5dH1	06N-**BI**BsF(6)	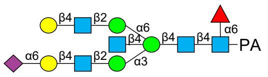	5.536	0.479	✓		
71.41	22.59	22.02	78.37		1552.05	1552.57	2H+	3103.13	N3H6Hn5dH1	trN-F-**TR**	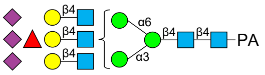	3.254	0.281			
71.41	22.59		78.37		1261.28	1261.48	2H+	2520.94	N1H6Hn5dH1	N-**TRF**(6)	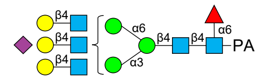	1.406	0.122			
71.41	22.59	22.47	78.37	78.21	953.59	953.87	2H+	1905.73	H4Hn5dH1	**BI**BsF(6)-Ga2	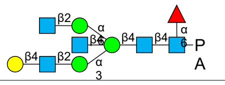	1.385	0.120	✓		
71.41	22.59		78.37		1406.53	1407.02	2H+	2812.03	N2H6Hn5dH1	dN-**TRF**(6)	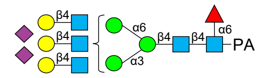	0.808	0.070			
72.09	23.11	23.53	79.02		1325.47	1326.00	2H+	2649.98	N2H5Hn5dH1	66N-**BI**BsF(6)	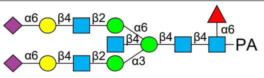	4.427	0.383	✓		
72.09	23.11	23.29	79.02		1406.71	1407.02	2H+	2812.03	N2H6Hn5dH1	dN-**TRF**(6)	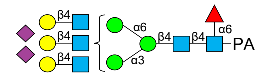	2.552	0.221			dLn
72.09	23.11		79.02		1454.89							1.367	0.118			
72.09	23.11	23.35	79.02	78.21	953.51	953.87	2H+	1905.73	H4Hn5dH1	**BI**BsF(6)-Ga1	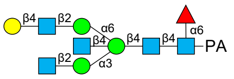	0.733	0.063	✓		
72.09	23.11	22.00	79.02		1132.80	1132.41	3H+	3394.22	N4H6Hn5dH1	teN-**TRF**(6)	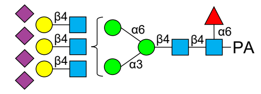	0.342	0.030		✓	
73.46	24.23	23.84	80.34	80.00	1034.55	1034.90	2H+	2067.79	H5Hn5dH1	**BI**BsF(6)	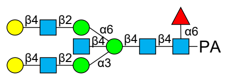	12.226	1.057	✓		
73.46	24.23	23.29	80.34		1406.66	1407.02	2H+	2812.03	N2H6Hn5dH1	dN-**TRF**(6)	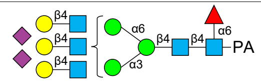	6.237	0.539	✓		
73.46	24.23		80.34		1217.04	1217.47	2H+	2432.92	H6Hn6dH1	**TR**BsF(6)	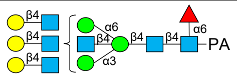	0.759	0.066			
75.40	25.97	25.99	82.20		1325.56	1326.00	2H+	2649.98	N2H5Hn5dH1	36N-**BI**BsF(6)	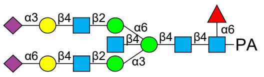	1.131	0.098			

S4 Table continued.

RT (min)	GU experi-mental	GU std.	RP experi-mental	RP std.	m/z experi-mental	m/z theoretical	ion form	MS	Composition (+PA)		Glycan structure	Quantity ratio % for top	Quantity ratio % for whole	MS[2]	Only	Note
75.40	25.97		82.20		1406.60	1407.02	2H+	2812.03	N2H6Hn4dH1	dN-**TRF**(6)	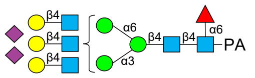	0.941	0.081			
75.40	25.97		82.20		1099.28	1099.42	2H+	2196.83	N1H4Hn5dH1	N-**BI**BsF(6)-Ga	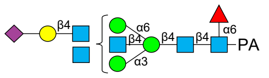	0.292	0.025			
75.54	26.11		82.33		1552.71	1552.57	2H+	3103.13	N3H6Hn5dH1	trN-**TRF**(6)	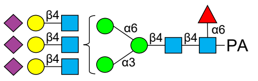	10.835	0.937	✓		
75.54	26.11	24.25	82.33		1407.25	1407.02	2H+	2812.03	N2H6Hn5dH1	dN-**TRF**(6)	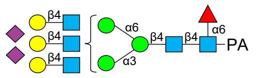	1.979	0.171			
75.54	26.11	26.99	82.33		1144.34	1144.44	2H+	2286.86	H6Hn6	**TR**Bs	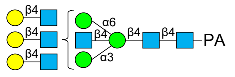	0.831	0.072	✓		
75.54	26.11		82.33		1326.30	1326.00	2H+	2649.98	N2H5Hn5dH1	dN-**BI**BsF(6)	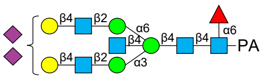	0.281	0.024			
75.54	26.11		82.33		1099.00	1099.42	2H+	2196.83	N1H4Hn5dH1	N-**BI**BsF(6)-Ga	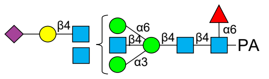	0.130	0.011		✓	
76.79	27.35	27.33	83.53	83.11	1180.41	1180.45	2H+	2358.88	N1H5Hn5dH1	30N-**BI**BsF(6)	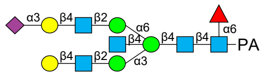	1.024	0.089	✓		
77.45	28.05	27.52	84.16		1132.56	1132.41	3H+	3394.22	N4H6Hn5dH1	teN-**TRF**(6)	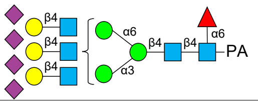	0.691	0.060			
77.45	28.05		84.16		1552.71	1552.57	2H+	3103.13	N3H6Hn5dH1	trN-Ln-**BI**F(6)	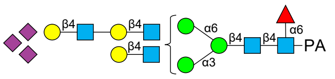	0.215	0.019			
77.45	28.05		84.16		1107.63	1107.93	2H+	2213.85	H5Hn5dH2	F-**BI**BsF(6)	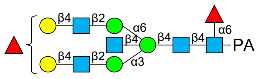	0.121	0.010			
78.04	28.70		84.73		1099.06	1099.42	2H+	2196.83	N1H4Hn5dH1	N-**BI**BsF(6)-Ga	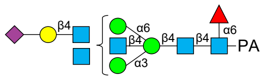	0.277	0.024			
79.16	29.99		85.80		1180.58	1180.96	2H+	2359.90	H5Hn5dH3	dF-**BI**BsF(6)	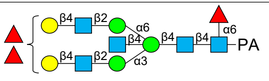	0.687	0.059			
79.16	29.99		85.80		1362.99	1363.01	2H+	2724.02	N1H6Hn6dH1	N-**TR**BsF(6)	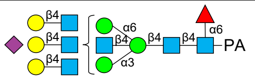	0.217	0.019			
80.16	31.23		86.76		1325.66	1326.00	2H+	2649.98	N2H5Hn5dH1	63N-**BI**BsF(6)	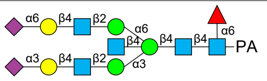	2.918	0.252	✓		
81.19	32.59		87.75		1180.68	1180.96	2H+	2359.90	H5Hn5dH3	dF-**BI**BsF(6)	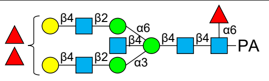	10.262	0.887		✓	
82.68	34.72	34.05	89.18		1290.14	1290.50	2H+	2578.98	H6Hn6dH2	F(3)-**TR**BsF(6)	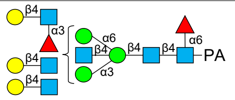	1.740	0.150	-		Le^x
84.37	37.40		90.80		1217.37	1217.47	2H+	2432.92	H6Hn6dH1	**TR**BsF(6)	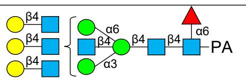	2.149	0.186	-		
87.15	42.50		93.47		1435.51	1435.53	2H+	2870.07	N1H6Hn6dH2	N-F-**TR**BsF(6)	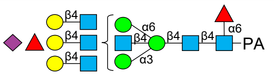	1.265	0.109			
87.15	42.50		93.47		1230.54	1229.79	3H+	3686.34	N4H6Hn5dH3	teN-dF-**TRF**(6)	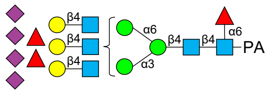	0.198	0.017		✓	

S5 Table. *N*-glycans from human urine M2 analyzed using the improved method.

RT (min)	GU		RP		*m/z*			MS	Composition (+PA)		Glycan structure	Quantity ratio		MS^2	Only	Note
	experi-mental	std.	experi-mental	std.	experi-mental	theoretical	ion form					% for top	% for whole			
23.69	5.18	5.11	30.67	29.07	899.72	900.33	2H+	1798.65	H8Hn2	**M8A**	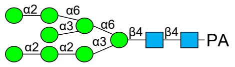	2.953	0.462	✓		
23.69	5.18	5.14	30.67		1049.53	1049.89	2H+	2097.76	N2H5Hn4	66N-**BI'**	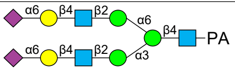	0.988	0.155		✓	
23.69	5.18		30.67		1186.37		2H+		N2H2Hn2+1059			0.443	0.069			
24.81	5.36	5.31	31.75	30.36	818.71	819.31	2H+	1636.60	H7Hn2	**M7A**	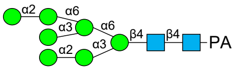	2.762	0.432	✓		
25.29	5.44		32.22		1069.32	1069.39	H+	1068.39	H6	**Glc6**	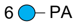	1.714	0.268	✓	✓	
25.29	5.44		32.22		1041.22	1041.89	2H+	2081.77	N2H4Hn3dH1	dN-F-Ln-**M2'**	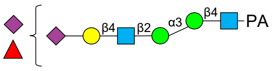	0.239	0.037			
25.29	5.44		32.22		818.79	819.31	2H+	1636.60	H7Hn2	**M7A**						
26.02	5.56	5.50	32.92	31.33	980.83	981.36	2H+	1960.70	H9Hn2	**M9A**	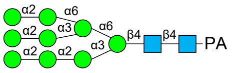	1.319	0.206	✓		
26.02	5.56		32.92		1186.02		2H+					0.313	0.049			
26.84	5.70		33.71		1231.35	1231.45	H+	1230.44	H7	**Glc7**	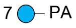	4.132	0.646	✓		
27.44	5.81	5.76	34.29	32.76	899.39	900.33	2H+	1798.65	H8Hn2	**M8B**	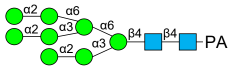	0.322	0.050	✓		
28.08	5.93	5.88	34.90	33.44	818.97	819.31	2H+	1636.60	H7Hn2	**M7B**	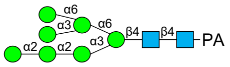	3.736	0.584	✓		
28.08	5.93		34.90		1231.42	1231.45	H+	1230.44	H7	**Glc7**	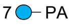	2.911	0.455	✓		
28.08	5.93		34.90		1106.51	1107.42	2H+	2212.83	N1H5Hn5	N-**BI**Bs/ga	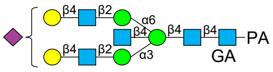					
29.32	6.16	6.11	36.10	34.37	737.68	738.28	2H+	1474.54	H6Hn2	**M6B**	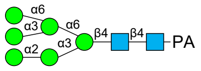	13.093	2.048	✓		
30.36	6.37		37.10		1393.36	1393.50	H+	1392.49	H8	**Glc8**	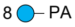	0.288	0.045	✓	✓	
30.99	6.50	6.48	37.71		899.90	900.33	2H+	1798.65	H8Hn2	**M8C**	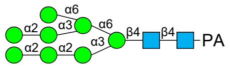	0.436	0.068			
31.84	6.67	6.72	38.52		1004.70	1005.88	2H+	2009.75	N1H5Hn4	06N-**BI**/ga	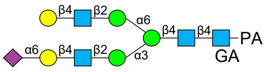					
32.71	6.86		39.36		1004.56	1005.88	2H+	2009.75	N1H5Hn4	06N-**BI**/epi	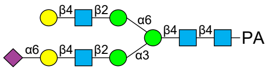			✓		
32.71	6.86	6.80	39.36	37.73	818.72	819.31	2H+	1636.60	H7Hn2	**M7D**	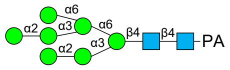	0.406	0.064			
32.71	6.86		39.36		1135.34	1135.45	H+	1134.44	H3Hn2dH1	**M3B**F(6)/epi	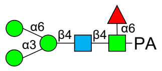			✓		
32.71	6.86	7.08	39.36	38.72	989.31	989.39	H+	988.39	H3Hn2	**M3B**				✓		
33.34	7.00	7.00	39.99	38.72	1313.32	1313.50	H+	1312.49	H5Hn2	**M5A**	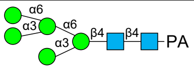	17.833	2.790	✓		
33.34	7.00	7.08	39.99	38.72	989.30	989.39	H+	988.39	H3Hn2	**M3B**	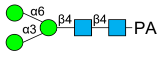	12.538	1.962	✓		
33.34	7.00	7.04	39.99	38.72	827.28	827.34	H+	826.33	H2Hn2	**M2B**	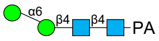	7.424	1.162	✓		
33.34	7.00		39.99		1133.25	1135.45	H+	1134.44	H3Hn2dH1	**M3B**F(6)/ga	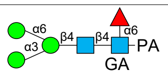			✓		
33.34	7.00	6.72	39.99		1151.29	1151.45	H+	1150.44	H4Hn2	**M4A**	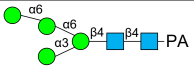	1.581	0.247	✓		

S5 Table continued.

RT (min)	GU experi-mental	GU std.	RP experi-mental	RP std.	*m/z* experi-mental	*m/z* theoretical	*m/z* ion form	MS	Composition (+PA)		Glycan structure	Quantity ratio % for top	Quantity ratio % for whole	MS2	Only	Note
34.04	7.16	7.06	40.73		1151.31	1151.45	H+	1150.44	H4Hn2	**M4B**	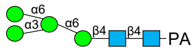	0.440	0.069	✓		
34.04	7.16		40.73		1004.84	1005.88	2H+	2009.75	N1H5Hn4	30N-**BI**/ga	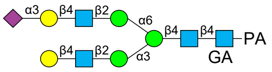					
34.04	7.16		40.73		899.75	900.33	2H+	1798.65	H8Hn2	**M8**	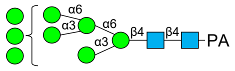	0.100	0.016		✓	
34.04	7.16		40.73		1313.39	1313.50	H+	1312.49	H5Hn2	**M5A**						
34.04	7.16	7.00	40.73		1133.16	1135.45	H+	1134.44	H3Hn2dH1	**M3BF**(6)/ga						
35.25	7.45		42.00		859.14							0.535	0.084	-		
35.25	7.45		42.00		1004.55	1005.88	2H+	2009.75	N1H5Hn4	30N-**BI**/ga				✓		
35.25	7.45		42.00		1150.91	1151.43	2H+	2300.84	N2H5Hn4	66N-**BI**/ga						
35.85	7.60	7.56	42.64		1150.40	1151.43	2H+	2300.84	N2H5Hn4	66N-**BI**/ga	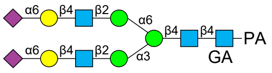			✓		
37.05	7.90	7.88	43.91		1151.24	1151.43	2H+	2300.84	N2H5Hn4	66N-**BI**/epi	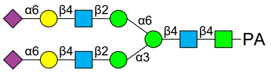	3.821	0.598	✓		
37.64	8.05		44.53		1151.31	1151.43	2H+	2300.84	N2H5Hn4	36N-**BI**/epi	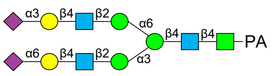	5.873	0.919	✓		
37.64	8.05	8.03	44.53		985.09	985.37	2H+	1968.72	N1H6Hn3	6N-Ln-**M5A**	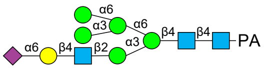	3.738	0.585	✓		
37.64	8.05	8.09	44.53	42.84	823.18	823.31	2H+	1644.61	N1H4Hn3	6N-**MO1**	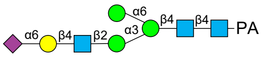	3.702	0.579	✓		
37.64	8.05		44.53		1179.11	1179.41	2H+	2356.80	P1 N1H7Hn3dH1	6P-N-Ln-**M6**F(6)	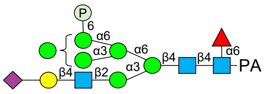	0.767	0.120			
38.20	8.20	8.30	45.12		1150.95	1151.43	2H+	2300.84	N2H5Hn4	36N-**BI**/ga	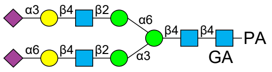			✓		
38.20	8.20	8.25	45.12	43.68	904.19	904.34	2H+	1806.67	N1H5Hn3	6N-Ln-**M4C**	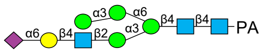	0.791	0.124	✓		
38.20	8.20		45.12		1078.78	1078.91	2H+	2155.80	N1H5Hn4dH1	3N-F(3)-**BI**	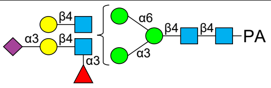	0.259	0.041			SLe^x
39.44	8.54		46.43		1179.86	1179.41	2H+	2356.80	P1 N1H7Hn3dH1	6P-N-Ln-**M6**F(6)	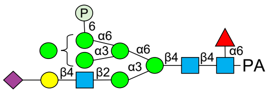	0.244	0.038			
39.44	8.54		46.43		879.51							0.176	0.028			
39.44	8.54		46.43		1150.39	1151.43	2H+	2300.84	N2H5Hn4	dN-**BI**/ga	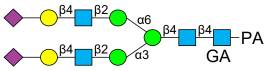					
39.44	8.54	8.67	46.43		770.64	771.31	2H+	1540.60	H3Hn4dH1	**AG12**F(6)/ga	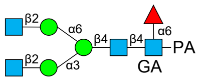					
39.74	8.62		46.75		1179.54	1179.41	2H+	2356.80	P1 N1H7Hn3dH1	6P-N-Ln-**M6**F(6)	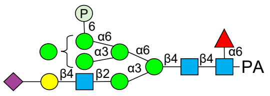	0.784	0.123			
39.74	8.62		46.75		1150.85	1151.43	2H+	2300.84	N2H5Hn4	dN-**BI**/ga	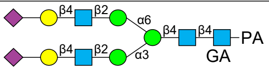					
39.74	8.62	8.64	46.75		1079.38	1079.42	2H+	2156.82	H5Hn4dH3	F(2)-F(3)-**BI**F(6)	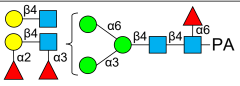	0.564	0.088			Le^y
40.57	8.85		47.62		851.26	851.33	2H+	1700.64	H4Hn4dH1	**BI**F(6)-Ga/epi	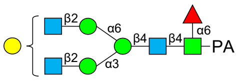	0.254	0.040		✓	
40.57	8.85		47.62		1332.88	1333.99	2H+	2665.97	N2H6Hn5	dN-**TR**/ga	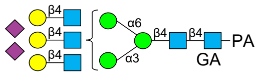					

S5 Table continued.

RT (min)	GU experi-mental	GU std.	RP experi-mental	RP std.	*m/z* experi-mental	*m/z* theoretical	*m/z* ion form	MS	Composition (+PA)	Glycan structure		Quantity ratio % for top	Quantity ratio % for whole	MS^2	Only	Note
40.57	8.85	9.13	47.62		1150.62	1151.43	2H+	2300.84	N2H5Hn4	33N-**BI**/epi						
41.57	9.14		48.68		1551.38	1552.57	2H+	3103.13	N3H6Hn5dH1	trN-F-**TR**	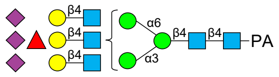	0.138	0.022		✓	
41.57	9.14		48.68		851.05	851.33	2H+	1700.64	H4Hn4dH1	**BI**F(6)-Ga/epi		0.128	0.020			
41.57	9.14	9.09	48.68		985.05	985.37	2H+	1968.72	N1H6Hn3	N-Ln-**M5A**	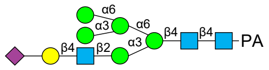	0.087	0.014		✓	
41.57	9.14	9.13	48.68		1151.32	1151.43	2H+	2300.84	N2H5Hn4	33N-**BI**/epi	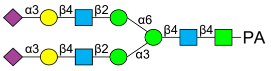	0.076	0.012			
41.57	9.14		48.68		811.26	811.35	H+	810.34	H1Hn2dH1	**M1A**F(6)	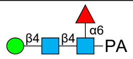	0.028	0.004			
42.04	9.28		49.17		697.81	697.25	2H+	1392.49	H8	**Glc8**	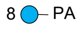	3.279	0.513	-		
42.04	9.28	9.56	49.17		1192.31	1192.47	H+	1191.47	H3Hn3	**AG2**	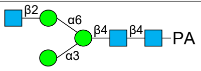	1.309	0.205	✓		
42.04	9.28		49.17		1551.12	1552.57	2H+	3103.13	N3H6Hn5dH1	trN-F-**TR**/ga	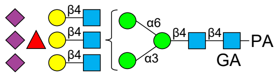				✓	
42.04	9.28		49.17		932.55	933.36	2H+	1864.71	H5Hn4dH1	**BI**F(6)/ga	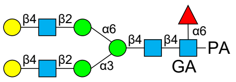					
42.04	9.28		49.17		1333.50	1333.99	2H+	2665.97	N2H6Hn5	dN-**TR**	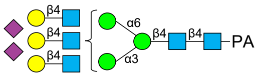	0.425	0.066			
42.99	9.57	9.63	50.18	48.64	924.71	924.85	2H+	1847.69	N1H4Hn4	06N-**BI**-Ga2	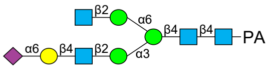	0.397	0.062	✓		
42.99	9.57		50.18		1478.09	1479.54	2H+	2957.07	N3H6Hn5	trN-F-**TR**/ga						
42.99	9.57		50.18		1297.06	1297.49	2H+	2592.96	N2H5Hn4dH2	dN-F-**BI**F(6)/ga						
43.40	9.69		50.61		1478.53	1479.54	2H+	2957.07	N3H6Hn5	trN-F-**TR**/ga	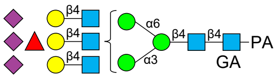					
43.40	9.69		50.61		1026.08	1026.39	2H+	2050.77	N1H4Hn5	N-**BI**Bs-Ga	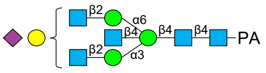	0.665	0.104			
43.40	9.69		50.61		985.96	986.70	3H+	2957.07	N3H6Hn5	trN-**TR**/ga	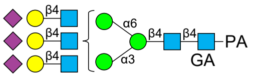					
43.40	9.69		50.61		1296.57	1297.49	2H+	2592.96	N2H5Hn4dH2	N-3N-F(3)-**BI**F(6)/ga	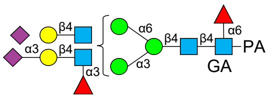					SLe^x
43.40	9.69	9.63	50.61	48.64	924.54	924.85	2H+	1847.69	N1H4Hn4	06N-**BI**-Ga2		0.169	0.027			
44.28	9.96	10.07	51.54		1224.24	1224.46	2H+	2446.90	N2H5Hn4dH1	N-3N-F(3)-**BI**	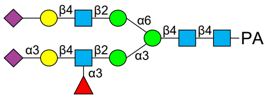	2.329	0.364	✓		SLe^x
44.28	9.96	10.05	51.54		860.19	860.33	2H+	1718.65	H5Hn4	**BI**	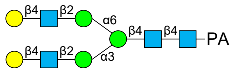	1.680	0.263	✓		
44.28	9.96	10.07	51.54	50.51	973.33	973.40	H+	972.39	H2Hn2dH1	**M2B**F(6)						
44.92	10.17		52.21	50.87	1005.87	1005.88	2H+	2009.75	N1H5Hn4	06N-**BI**	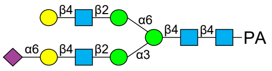	17.620	2.757	✓		
44.92	10.17	10.07	52.21	50.51	1135.33	1135.45	H+	1134.44	H3Hn2dH1	**M3B**F(6)	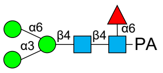	13.063	2.044	✓		
44.92	10.17		52.21		1077.95	1078.91	2H+	2155.80	N1H5Hn4dH1	06N-**BI**F(6)/ga	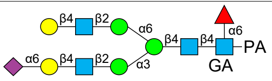			✓		
44.92	10.17	10.07	52.21	50.51	973.42	973.40	H+	972.39	H2Hn2dH1	**M2B**F(6)	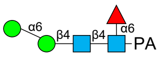	8.431	1.319	✓		

S5 Table continued.

RT (min)	GU		RP		*m/z*			MS	Composition (+PA)	Glycan structure		Quantity ratio		MS^2	Only	Note
	experi-mental	std.	experi-mental	std.	experi-mental	theoretical	ion form					% for top	% for whole			
46.09	10.54	10.55	53.39		1223.41	1224.97	2H+	2446.90	N2H5Hn4dH1	66N-**BI**F(6)/ga	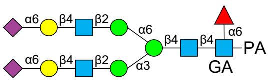					
44.92	10.17		52.21		1118.92	1118.89	2H+	2235.76	S1N1H5Hn4dH	S-N-**BI**F(6)					✓	
46.09	10.54	10.55	53.39		1223.41	1224.97	2H+	2446.90	N2H5Hn4dH1	66N-**BI**F(6)/ga				✓		
46.09	10.54		53.39		1118.92	1118.89	2H+	2235.76	S1N1H5Hn4d H1	S-N-**BI**F(6)	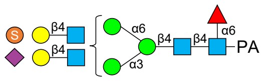	3.285	0.514	✓	✓	N-Ldn
46.09	10.54		53.39		1333.69	1333.99	2H+	2665.97	N2H6Hn5	dN-**TR**	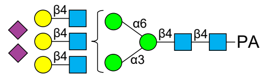	1.827	0.286			
47.01	10.85		54.32		1243.88	1243.46	3H+	3727.36	N4H5Hn6dH3	teN-dF-Ldn-**BI**F(6)	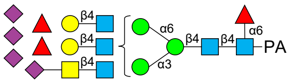	0.154	0.024	✓		N-Ldn
47.01	10.85		54.32		1078.29	1078.91	2H+	2155.80	N1H5Hn4dH1	N-**BI**F(6)/ga	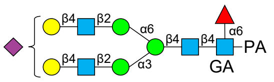			✓		
47.01	10.85		54.32		1119.84	1119.94	2H+	2237.86	N1H3Hn6dH1	N-dLdn-**M3**F(6)	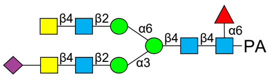	0.081	0.013	✓		N-Ldn
47.01	10.85	10.91	54.32		1223.13	1224.46	2H+	2446.90	N2H5Hn4dH1	36N-**BI**F(6)/ga						
47.61	11.05	10.91	54.92		1223.91	1224.46	2H+	2446.90	N2H5Hn4dH1	36N-**BI**F(6)/ga	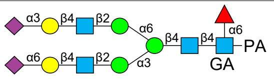			✓		
47.61	11.05	11.15	54.92		1077.85	1078.91	2H+	2155.80	N1H5Hn4dH1	03N-**BI**F(6)/ga	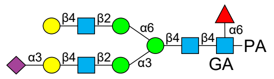			✓		
47.61	11.05		54.92		1180.84	1180.96	2H+	2359.90	H5Hn5dH3	F(2)-F(3)-**BI**BsF(6)	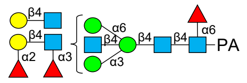	2.604	0.407			Le^y
47.61	11.05	11.73	54.92		1261.06	1261.48	2H+	2520.94	N1H6Hn5dH1	N-**TR**F(6)	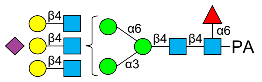	1.721	0.269			
47.61	11.05	10.91	54.92		1034.92	1034.90	2H+	2067.79	H5Hn5dH1	F-**BI**Bs	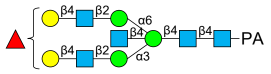	0.883	0.138			
48.02	11.19	11.49	55.34	54.54	1151.44	1151.43	2H+	2300.84	N2H5Hn4	66N-**BI**				✓		
48.02	11.19		55.34		1180.50	1180.96	2H+	2359.90	H5Hn5dH3	F(2)-F(3)-**BI**BsF(6)	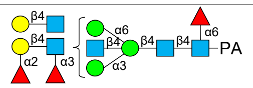	0.129	0.020	✓		Le^y
48.02	11.19	11.12	55.34		1034.64	1034.90	2H+	2067.79	H5Hn5dH1	**BI**BsF(6)	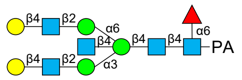	0.071	0.011			
48.02	11.19	11.33	55.34	55.68	916.81	916.86	2H+	1831.70	N1H3Hn4dH1	N-Ldn1-**M3**F(6)	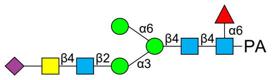	0.059	0.009			N-Ldn
48.02	11.19	11.65	55.34		1223.87	1224.46	2H+	2446.90	N2H5Hn4dH1	33N-**BI**F(6)/ga	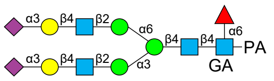					
48.02	11.19	11.06	55.34		1077.58	1078.91	2H+	2155.80	N1H5Hn4dH1	66N-**BI**F(6)/ga						
48.67	11.41	11.49	55.99	54.54	1151.33	1151.43	2H+	2300.84	N2H5Hn4	66N-**BI**	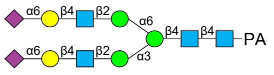	100	15.575	✓		
49.52	11.71	11.65	56.85		1223.20	1224.46	2H+	2446.90	N2H5Hn4dH1	33N-**BI**F(6)/ga	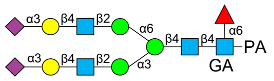			✓		
49.52	11.71	11.98	56.85	55.95	1005.86	1005.88	2H+	2009.75	N1H5Hn4	30N-**BI**	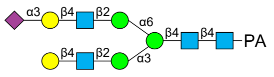	0.808	0.126	✓		
49.52	11.71	12.02	56.85	56.14	1151.42	1151.43	2H+	2300.84	N2H5Hn4	66N-**BI**				✓		
50.21	11.96	12.02	57.54	56.14	1151.07	1151.43	2H+	2300.84	N2H5Hn4	36N-**BI**	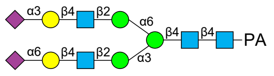	10.353	1.620	✓		
50.21	11.96	11.65	57.54		1223.22	1224.46	2H+	2446.90	N2H5Hn4dH1	33N-**BI**F(6)/ga				✓		

S5 Table continued.

RT (min)	GU experi-mental	GU std.	RP experi-mental	RP std.	m/z experi-mental	m/z theoretical	m/z ion form	MS	Composition (+PA)	Glycan structure		Quantity ratio % for top	Quantity ratio % for whole	MS^2	Only	Note
50.21	11.96	11.98	57.54	55.95	1005.48	1005.88	2H+	2009.75	N1H5Hn4	30N-**BI**	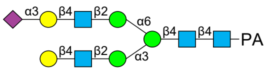	1.740	0.272	✓		
50.21	11.96	12.00	57.54	56.09	771.75	771.31	2H+	1540.60	H3Hn4dH1	**AG12**F(6)	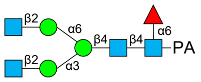	1.092	0.171	✓		
50.21	11.96	11.68	57.54	59.36	1338.32	1338.53	H+	1337.52	H3Hn3dH1	**AG2**F(6)	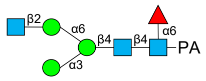	0.513	0.080			
51.90	12.58		59.25		1253.13	1253.48	2H+	2504.94	N1H5Hn5dH2	N-F-Gn-Ln-**M4**F(6)	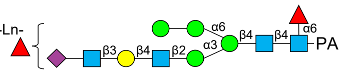	0.557	0.087			
51.90	12.58		59.25		1406.10	1407.02	2H+	2812.03	N2H6Hn4dH1	dN-F-**TR**						
51.90	12.58		59.25		851.97	852.33	2H+	1702.66	H4Hn4dH1	**BI**F(6)-Ga2					✓	
51.90	12.58		59.25		1551.86	1552.57	2H+	3103.13	N3H6Hn5dH1	trN-F-**TR**/ga	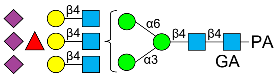					
51.90	12.58		59.25		1188.33	1188.45	2H+	2374.88	N1H6Hn5	N-**TR**	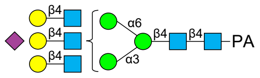	0.172	0.027			
52.21	12.69	13.11	59.56	60.83	852.29	852.33	2H+	1702.66	H4Hn4dH1	**BI**F(6)-Ga2	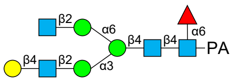	5.561	0.870	✓		
52.21	12.69		59.56		1224.04	1224.46	2H+	2446.90	N2H5Hn4dH1	33N-**BI**F(6)/epi	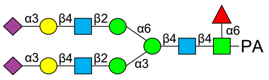	1.534	0.240			
52.21	12.69		59.56		1406.98	1407.02	2H+	2812.03	N2H6Hn4dH1	dN-F-**TR**	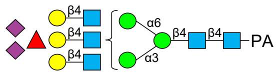	1.325	0.207			
52.21	12.69	13.08	59.56	60.45	1551.82	1552.57	2H+	3103.13	N3H6Hn5dH1	trN-F-**TR**						
52.82	12.92	13.08	60.17	60.45	1551.82	1552.57	2H+	3103.13	N3H6Hn5dH1	trN-F-**TR**	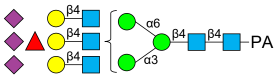	1.189	0.186			
52.82	12.92		60.17		1018.16	1018.40	2H+	2034.78	N1H3Hn5dH1	N-F-Gn-Ldn-**M3**	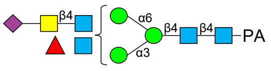	0.358	0.056		✓	N-Ldn
52.82	12.92		60.17		1405.86	1407.02	2H+	2812.03	N2H6Hn4dH1	dN-F-**TR**/ga	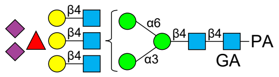					
52.82	12.92		60.17		1034.53	1034.90	2H+	2067.79	H5Hn5dH1	Gn-**BI**F(6)	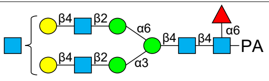	0.278	0.044			
52.82	12.92	13.35	60.17		1151.16	1151.43	2H+	2300.84	N2H5Hn4	63N-**BI**	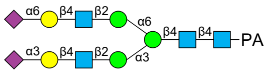	0.218	0.034			
52.82	12.92		60.17		852.27	852.33	2H+	1702.66	H4Hn4dH1	**BI**F(6)-Ga2		0.159	0.025			
54.34	13.51	13.61	61.70	60.83	933.11	933.36	2H+	1864.71	H5Hn4dH1	**BI**F(6)	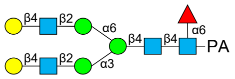	5.748	0.899	✓		
54.34	13.51		61.70		1099.59	1099.42	2H+	2196.83	N1H4Hn5dH1	N-Ldn-**MO**F(6)	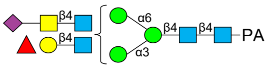	5.102	0.798	✓		N-Ldn
54.34	13.51		61.70		1552.76	1552.57	2H+	3103.13	N3H6Hn5dH1	trN-F(3)-**TR**				✓		SLex
54.34	13.51	14.01	61.70		1334.40	1333.99	2H+	2665.97	N2H6Hn5	dN-**TR**	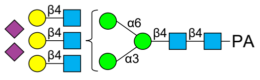	1.048	0.164			
54.34	13.51		61.70		1406.41	1407.02	2H+	2812.03	N2H6Hn4dH1	dN-Ln-**BI**F(6)	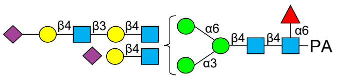	0.912	0.143			N-dLn
54.34	13.51	13.72	61.70		997.81	997.88	2H+	1993.75	N1H4Hn4dH1	06N-**BI**F(6)-Ga2	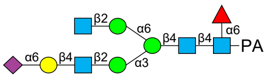	0.791	0.124			
54.34	13.51	13.35	61.70		1151.02	1151.43	2H+	2300.84	N2H5Hn4	63N-**BI**	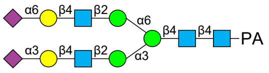	0.435	0.068			

S5 Table continued.

RT (min)	GU experi-mental	GU std.	RP experi-mental	RP std.	*m/z* experi-mental	*m/z* theoretical	*m/z* ion form	MS	Composition (+PA)	Glycan structure	Quantity ratio % for top	Quantity ratio % for whole	MS^2 Only	Note
54.34	13.51	13.37	61.70		1296.97	1297.49	2H+	2592.96	N2H5Hn4dH2	dN-F(3)2-**BI**F(6) 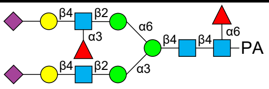	0.361	0.056		SLe^x
55.09	13.81	13.80	62.46		1552.3	1552.57	2H+	3103.13	N3H6Hn5dH1	3N-dN-F(3)-**TR** 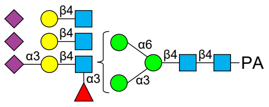	17.297	2.706	-	SLe^x
55.09	13.81		62.46		1333.92	1333.99	2H+	2665.97	N2H6Hn5	dN-Ln-**BI** 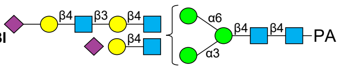	3.244	0.507		N-dLn
55.09	13.81	14.08	62.46		1324.56	1326.00	2H+	2649.98	N2H5Hn5dH1	66N-**BI**BsF(6)/ga 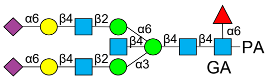				
55.09	13.81		62.46		1369.61	1370.00	2H+	2737.99	N3H5Hn4dH1	6N-3N-N-**BI**F(6) 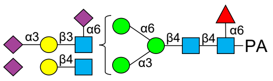	2.681	0.419		dN-Ln
55.09	13.81	14.12	62.46	62.25	1078.83	1078.91	2H+	2155.80	N1H5Hn4dH1	06N-**BI**F(6)			✓	
55.09	13.81		62.46		1099.35	1099.42	2H+	2196.83	N1H4Hn5dH1	N-Ldn-**MO**F(6)				
55.82	14.11	14.12	63.20	62.25	1078.87	1078.91	2H+	2155.80	N1H5Hn4dH1	06N-**BI**F(6) 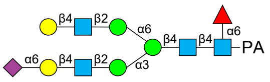	32.384	5.067	✓	
55.82	14.11	14.12	63.20		1552.42	1552.57	2H+	3103.13	N3H6Hn5dH1	3N-dN-F(3)-**TR** 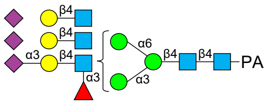	3.327	0.521	-	SLe^x
55.82	14.11	14.23	63.20	62.56	1151.54	1151.43	2H+	2300.84	N2H5Hn4	33N-**BI** 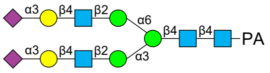	1.799	0.282	✓	
55.82	14.11		63.20		1179.44	1180.45	2H+	2358.88	N1H5Hn5dH1	06N-**BI**BsF(6)/ga 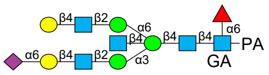				
55.82	14.11	14.01	63.20		1333.82	1333.99	2H+	2665.97	N2H6Hn5	dN-Ln-**BI** 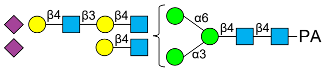	0.940	0.147		dLn
56.96	14.58	14.99	64.34	65.17	1479.86	1479.54	2H+	2957.07	N3H6Hn5	trN-**TR** 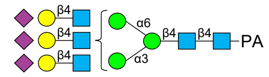	29.488	4.613	✓	
56.96	14.58	14.96	64.34		1107.08	1107.42	2H+	2212.83	N1H5Hn5	06N-**BI**Bs 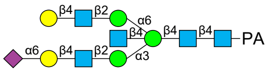	6.337	0.991	✓	
56.96	14.58		64.34		1552.35	1552.57	2H+	3103.13	N3H6Hn5dH1	trN-F-**TR** 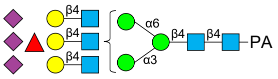	5.400	0.845		
56.96	14.58		64.34		1232.63	1232.96	2H+	2463.91	N1H6Hn4dH2	N-Ga-dF-**BI** 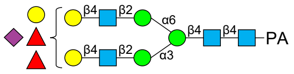	5.385	0.843	✓	
56.96	14.58		64.34		1244.47	1244.97	2H+	2487.93	N2H4Hn5dH1	dN-Ldn-**MO**F(6) 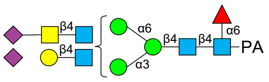	2.810	0.440	✓	N−Ldn
56.96	14.58	14.48	64.34		1026.94	1026.90	2H+	2051.79	H4Hn5dH2	F-**BI**BsF(6)-Ga 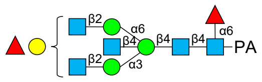	2.400	0.375		
58.09	15.06	14.87	65.48	64.27	1224.36	1224.46	2H+	2446.90	N2H5Hn4dH1	66N-**BI**F(6) 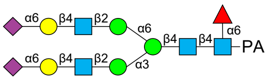	46.135	7.218	✓	
58.09	15.06	14.79	65.48		1119.88	1119.94	2H+	2237.86	N1H3Hn6dH1	06N-dLdn12-**M3**F(6) 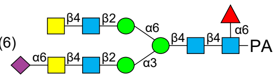	13.557	2.121		N-Ldn
58.09	15.06	15.35	65.48		1138.87	1139.40	2H+	2276.79	S1N1H4 Hn5dH1	S-N-Ldn1-**MO2**F(6) 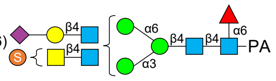	3.767	0.589		S-Ldn
58.09	15.06		65.48		1054.01	1054.34	2H+	2106.67	S2H3Hn6dH1	dS-dLdn-**M3**F(6)				
58.78	15.37	15.57	66.18	66.49	1479.32	1479.54	2H+	2957.07	N3H6Hn5	trN-Ln-**BI** 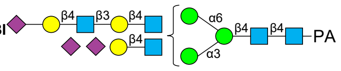	2.518	0.394	✓	N-dLn

S5 Table continued.

RT (min)	GU experi-mental	GU std.	RP experi-mental	RP std.	*m/z* experi-mental	*m/z* theoretical	*m/z* ion form	MS	Composition (+PA)	Glycan structure	Quantity ratio % for top	Quantity ratio % for whole	MS[2]	Only	Note
58.78	15.37	15.86	66.18	65.53	1078.62	1078.91	2H+	2155.80	N1H5Hn4dH1	60N-**BI**F(6) 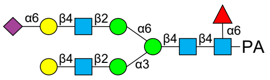	1.775	0.278	✓		
58.78	15.37		66.18		1244.51	1244.97	2H+	2487.93	N2H4Hn5dH1	36N-Ldn2-**MO1**F(6)					
58.78	15.37		66.18		974.29	974.39	2H+	1946.76	H3Hn6dH1	dLdn12-**M3**F(6) 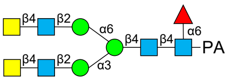	1.108	0.173			Ldn
58.78	15.37		66.18		1053.81	1054.34	2H+	2106.67	S2H3Hn6dH1	dS-dLdn-**M3**F(6) 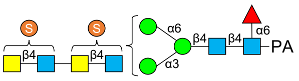	1.846	0.289			S-Ldn
58.78	15.37	14.79	66.18		1119.55	1119.94	2H+	2237.86	N1H3Hn6dH1	06N-dLdn12-**M3**F(6) 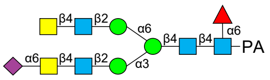	0.675	0.106	✓		N-Ldn
58.78	15.37	14.87	66.18	64.27	1224.25	1224.46	2H+	2446.90	N2H5Hn4dH1	66N-**BI**F(6)					
59.74	15.80	15.48	67.15		1244.86	1244.97	2H+	2487.93	N2H4Hn5dH1	36N-Ldn2-**MO1**F(6) 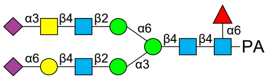	4.042	0.632	✓		N-Ldn
59.74	15.80	15.85	67.15		1157.22	1157.09	3H+	3468.26	N3Hn7H6dH1	trN-**TE**F(6) 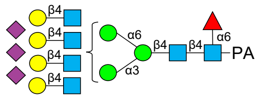	0.681	0.107			
59.74	15.80	16.73	67.15		961.52	961.87	2H+	1921.73	H5Hn5	**BI**Bs 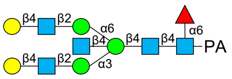	0.048	0.007			
61.04	16.41	16.30	68.42	67.84	1224.08	1224.46	2H+	2446.90	N2H5Hn4dH1	36N-**BI**F(6)					
61.04	16.41	16.26	68.42		1107.86	1107.93	2H+	2213.85	H5Hn5dH2	F(3)-**BI**BsF(6) 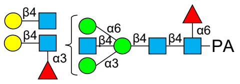	0.273	0.043			Le[x]
61.04	16.41	16.73	68.42		961.46	961.87	2H+	1921.73	H5Hn5	**BI**Bs 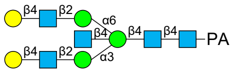	0.115	0.018			
61.46	16.61	16.30	68.82	67.84	1224.12	1224.46	2H+	2446.90	N2H5Hn4dH1	36N-**BI**F(6) 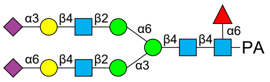	16.028	2.508	✓		
61.46	16.61	17.28	68.82		1078.48	1078.91	2H+	2155.80	N1H5Hn4dH1	03N-**BI**F(6) 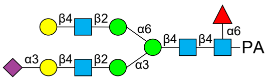	10.911	1.707	✓		
61.46	16.61	16.96	68.82		1479.08	1479.54	2H+	2957.07	N3H6Hn5	trN-**TR** 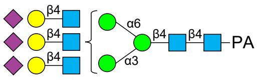	3.570	0.559			
62.14	16.94	17.66	69.47	68.72	1078.56	1078.91	2H+	2155.80	N1H5Hn4dH1	30N-**BI**F(6) 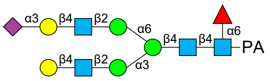	1.339	0.210	✓		
62.14	16.94		69.47		1261.28	1261.48	2H+	2520.94	N1H6Hn5dH1	N-**TR**F(6) 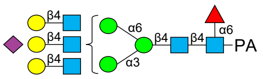	0.368	0.058			
63.34	17.55		70.63		1325.65	1326.00	2H+	2649.98	N2H5Hn5dH1	dN-Gn-**BI**F(6) 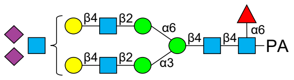	2.008	0.314			
63.34	17.55		70.63		1099.06	1099.42	2H+	2196.83	N1H4Hn5dH1	N-Ldn-**MO**F(6) 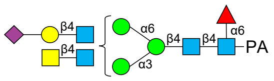	1.322	0.207			Ldn
63.34	17.55	17.43	70.63	70.68	1224.19	1224.46	2H+	2446.90	N2H5Hn4dH1	63N-**BI**F(6) 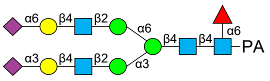	0.591	0.092			
64.94	18.41		72.16		1253.96	1254.13	3H+	3759.35	N4H7Hn6dH1	6N-3N-dN-F(3)-Ln-**TR** 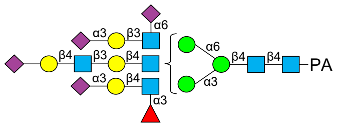	7.912	1.238			dN-Ln, N-dLn, SLe[x]
64.94	18.41		72.16		1151.82	1151.94	2H+	2301.86	N1H5Hn4dH2	3N-F(3)-**BI**F(6) 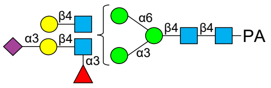	1.363	0.213			SLe[x]

S5 Table continued.

RT (min)	GU experi-mental	GU std.	RP experi-mental	RP std.	*m/z* experi-mental	*m/z* theoretical	*m/z* ion form	MS	Composition (+PA)	Glycan structure	Quantity ratio % for top	Quantity ratio % for whole	MS^2	Only	Note
64.94	18.41		72.16		1156.95	1157.43	3H+	3469.28	N2H7Hn6dH3	dN-trF-**TE** 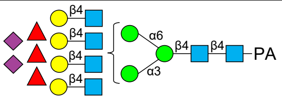	1.173	0.184			
65.86	18.93		73.04		1289.15	1289.99	2H+	2577.96	N1H6Hn6	N-**TR**Bs 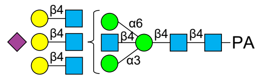	0.450	0.070			
65.86	18.93		73.04		1407.02	1407.02	2H+	2812.03	N2H6Hn4dH1	dN-**TR**F(6) 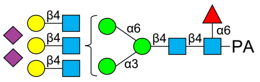	0.408	0.064			
65.86	18.93		73.04		1099.55	1099.42	2H+	2196.83	N1H4Hn5dH1	N-Ldn-**MO**F(6) 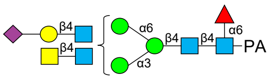	0.155	0.024			Ldn
67.01	19.61	19.43	74.15	73.91	1224.17	1224.46	2H+	2446.90	N2H5Hn4dH1	33N-**BI**F(6) 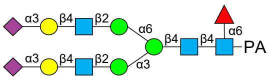	24.252	3.794	✓		
67.01	19.61		74.15		1407.24	1407.02	2H+	2812.03	N2H6Hn5dH1	dN-**TR**F(6) 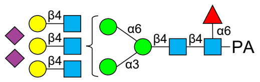	5.610	0.878			
67.01	19.61		74.15		1552.33	1552.57	2H+	3103.13	N3H6Hn5dH1	trN-**TR**F(6) 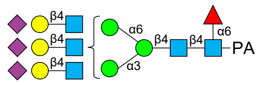	0.872	0.136			
68.11	20.29	21.99	75.20	77.61	872.62	872.85	2H+	1743.68	H3Hn5dH1	**AG12**BsF(6) 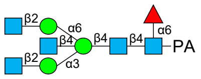	1.618	0.253	✓		
68.11	20.29		75.20		1216.04						0.213	0.033			
69.92	21.50	20.35	76.94		1552.33	1552.57	2H+	3103.13	N3H6Hn5dH1	trN-**TR**F(6) 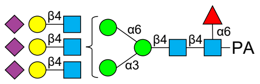	9.222	1.443			
71.41	22.59	21.99	78.37	77.61	1180.37	1180.45	2H+	2358.88	N1H5Hn5dH1	06N-**BI**BsF(6) 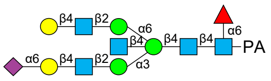	3.832	0.600	✓		
71.41	22.59	22.47	78.37	78.21	953.83	953.87	2H+	1905.73	H4Hn5dH1	**BI**BsF(6)-Ga2 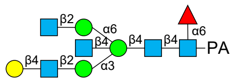	1.255	0.196	✓		
71.41	22.59	22.02	78.37		1552.85	1552.57	2H+	3103.13	N3H6Hn5dH1	trN-F-**TR** 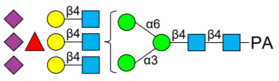	1.114	0.174			
71.41	22.59		78.37		1261.42	1261.48	2H+	2520.94	N1H6Hn5dH1	N-**TR**F(6) 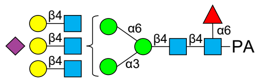	0.577	0.090			
71.41	22.59		78.37		1406.83	1407.02	2H+	2812.03	N2H6Hn5dH1	dN-**TR**F(6) 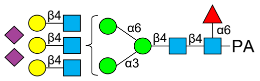	0.338	0.053			
72.00	23.04	23.53	78.94		1325.65	1326.00	2H+	2649.98	N2H5Hn5dH1	66N-**BI**BsF(6) 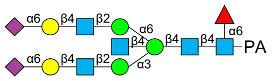	2.850	0.446	✓		
72.00	23.04	23.29	78.94		1406.89	1407.02	2H+	2812.03	N2H6Hn5dH1	dN-**TR**F(6) 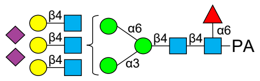	1.517	0.237			
72.00	23.04	23.35	78.94	78.21	953.60	953.87	2H+	1905.73	H4Hn5dH1	**BI**BsF(6)-Ga1 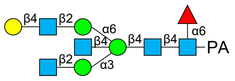	0.502	0.079	✓		
72.00	23.04		78.94		1454.82						0.343	0.054			
73.45	24.22	23.84	80.33	80.00	1034.79	1034.90	2H+	2067.79	H5Hn5dH1	**BI**BsF(6) 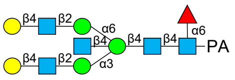	8.156	1.276	✓		
73.45	24.22	23.29	80.33		1407.20	1407.02	2H+	2812.03	N2H6Hn5dH1	dN-**TR**F(6) 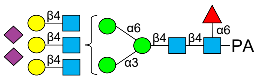	2.486	0.389			
73.45	24.22		80.33		1363.01	1363.01	2H+	2724.02	N1H6Hn6dH1	N-**TR**BsF(6) 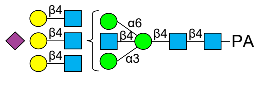	1.137	0.178		✓	
73.45	24.22		80.33		1217.33	1217.47	2H+	2432.92	H6Hn6dH1	**TR**BsF(6) 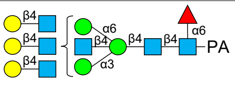	0.572	0.089			

S5 Table continued.

RT (min)	GU experi-mental	GU std.	RP experi-mental	RP std.	*m/z* experi-mental	*m/z* theoretical	*m/z* ion form	MS	Composition (+PA)	Glycan structure		Quantity ratio % for top	Quantity ratio % for whole	MS^2	Only	Note
75.17	25.76	25.99	81.98		1325.85	1326.00	2H+	2649.98	N2H5Hn5dH1	36N-**BI**BsF(6)	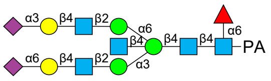	0.835	0.131			
75.17	25.76		81.98		1099.47	1099.42	2H+	2196.83	N1H4Hn5dH1	N-**BI**BsF(6)-Ga	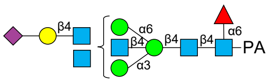	0.328	0.051			
75.17	25.76		81.98		1407.08	1407.02	2H+	2812.03	N2H6Hn4dH1	dN-**TR**F(6)	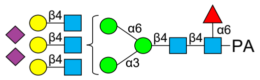	0.296	0.046			
75.55	26.12		82.34		1552.71	1552.57	2H+	3103.13	N3H6Hn5dH1	trN-**TR**F(6)	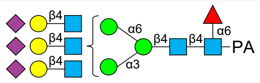	5.056	0.791	-		
75.55	26.12	24.25	82.34		1407.25	1407.02	2H+	2812.03	N2H6Hn5dH1	dN-**TR**F(6)	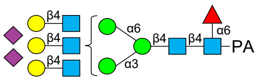	0.715	0.112			
75.55	26.12		82.34		1326.30	1326.00	2H+	2649.98	N2H5Hn5dH1	dN-**BI**BsF(6)	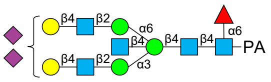	0.240	0.038			
75.55	26.12	26.99	82.34		1144.34	1144.44	2H+	2286.86	H6Hn6	**TR**Bs	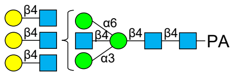	0.229	0.036			
76.76	27.32	27.33	83.50	83.11	1180.13	1180.45	2H+	2358.88	N1H5Hn5dH1	30N-**BI**BsF(6)	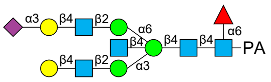	0.607	0.095			
77.48	28.08		84.19		1552.84	1552.57	2H+	3103.13	N3H6Hn5dH1	trN-Ln-**BI**F(6)	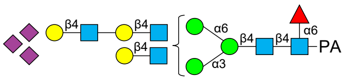	0.324	0.051			dLn
77.48	28.08	27.52	84.19		1132.17	1132.41	3H+	3394.22	N4H6Hn5dH1	teN-**TR**F(6)	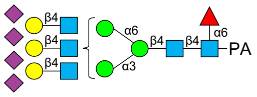	0.167	0.026			
77.48	28.08		84.19		1107.71	1107.93	2H+	2213.85	H5Hn5dH2	F-**BI**BsF(6)	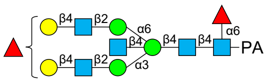	0.160	0.025			
78.15	28.82		84.84		1098.85	1099.42	2H+	2196.83	N1H4Hn5dH1	N-**BI**BsF(6)-Ga	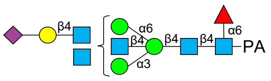	0.262	0.041			
79.17	30.00		85.81		1180.58	1180.96	2H+	2359.90	H5Hn5dH3	dF-**BI**BsF(6)	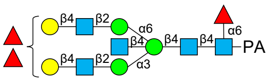	0.352	0.055			
79.17	30.00		85.81		1363.17	1363.01	2H+	2724.02	N1H6Hn6dH1	N-**TR**BsF(6)	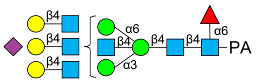	0.107	0.017			
80.28	31.38		86.88		1326.12	1326.00	2H+	2649.98	N2H5Hn5dH1	63N-**BI**BsF(6)	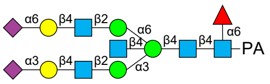	1.522	0.238			
81.33	32.78		87.89		1209.29							8.837	1.383		✓	
82.71	34.77	34.05	89.21		1290.73	1290.50	2H+	2578.98	H6Hn6dH2	F(3)-**TR**BsF(6)	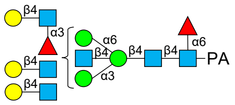	1.984	0.310	-		Le^x
84.44	37.52		90.87		1217.16	1217.47	2H+	2432.92	H6Hn6dH1	**TR**BsF(6)	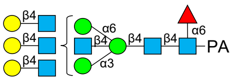	0.986	0.154			
87.27	42.74		93.59		1435.69	1435.53	2H+	2870.07	N1H6Hn6dH2	N-F-**TR**BsF(6)	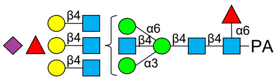	0.861	0.135			

S6 Table. *N*-glycans from CHO-K1 analyzed using the improved method.
In the “Note” column, “pep” denotes peptide-like peaks based on MS/MS data.

RT (min)	GU experi-mental	GU std.	RP experi-mental	RP std.	*m/z* experi-mental	*m/z* theoretical	*m/z* ion form	MS	Composition (+PA)	Glycan structure		Quantity ratio % for top	Quantity ratio % for whole	MS^2	Note
23.51	5.09	5.11	29.89	29.07	899.87	900.33	2H+	1798.65	H8Hn2	**M8A**	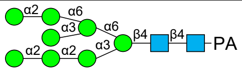	50.58	8.47	✓	
23.51	5.09		29.89		651.12		H+							✓	pep
24.74	5.29	5.31	31.07	30.36	818.82	819.31	2H+	1636.60	H7Hn2	**M7A**	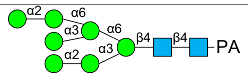	18.44	3.09	✓	
25.87	5.48	5.54	32.15	31.33	980.97	981.36	2H+	1960.70	H9Hn2	**M9A**	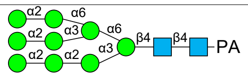	17.95	3.01	✓	
25.87	5.48		32.15		652.15		H+							✓	pep
27.41	5.75		33.63		635.04		H+							✓	pep
27.41	5.75	5.76	33.63	32.76	899.96	900.33	2H+	1798.65	H8Hn2	**M8B**	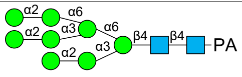	2.99	0.50	-	
28.05	5.87	5.88	34.25	33.44	818.95	819.31	2H+	1636.60	H7Hn2	**M7B**	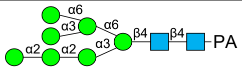	11.36	1.90	✓	
28.05	5.87		34.25		907.81		3H+					0.68	0.11		
28.74	6.00		34.91		636.14		H+							✓	pep
28.74	6.00		34.91		629.11		2H+							✓	pep
28.74	6.00	6.11	34.91	34.37	738.07	738.28	2H+	1474.54	H6Hn2	**M6B**					
29.29	6.11	6.11	35.44	34.37	737.95	738.28	2H+	1474.54	H6Hn2	**M6B**	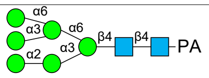	100	16.75	✓	
29.29	6.11	6.15	35.44		980.98	981.36	2H+	1960.70	H9Hn2	**G1M8A**	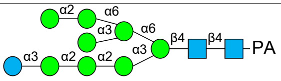	0.85	0.14		
29.29	6.11		35.44		636.19		H+							✓	pep
29.29	6.11	6.72	35.44		1151.41	1151.45	H+	1150.44	H4Hn2	**M4A**	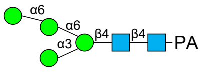	0.17	0.03		
29.90	6.23		36.02		737.86	738.28	2H+	1474.54	H6Hn2	**M6B**					
31.03	6.46	6.48	37.11	36.27	899.90	900.33	2H+	1798.65	H8Hn2	**M8C**	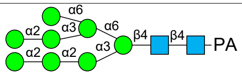	2.58	0.43	✓	
31.95	6.66	6.70	37.99		1062.20	1062.39	2H+	2122.76	H10Hn2	**G1M9A**	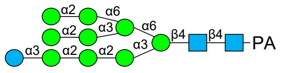	5.72	0.96	✓	
31.95	6.66		37.99		669.11		H+					0.58	0.10		
32.56	6.80	6.80	38.58	37.73	818.86	819.31	2H+	1636.60	H7Hn2	**M7D**	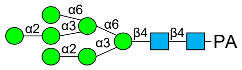	0.51	0.09	✓	
33.55	7.03	7.00	39.54	38.72	1313.49	1313.50	H+	1312.49	H5Hn2	**M5A**	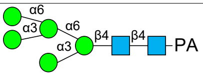	45.54	7.63	✓	
33.55	7.03	7.08	39.54	38.72	989.42	989.39	H+	988.39	H3Hn2	**M3B**	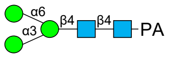	15.34	2.57	✓	
33.55	7.03	7.04	39.54	38.72	827.41	827.34	H+	826.33	H2Hn2	**M2B**	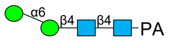	12.35	2.07	✓	
33.55	7.03		39.54		652.06		H+							✓	pep
33.55	7.03	7.06	39.54	38.72	1151.45	1151.45	H+	1150.44	H4Hn2	**M4B**	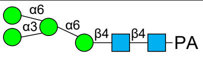	5.82	0.97	✓	
35.33	7.46		41.43		916.14		H+							✓	pep
35.33	7.46		41.43		1004.88	1005.88	2H+	2009.75	N1H5Hn4	N-**BI**/ga	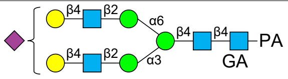				
35.58	7.53	7.52	41.70		839.81	839.82	2H+	1677.62	H6Hn3	Ln-**M5A**	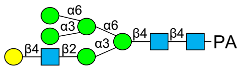	3.91	0.65		
37.04	7.91		43.25		659.09		H+							✓	pep
37.04	7.91		43.25		653.15		H+								pep
37.04	7.91		43.25		917.12		H+							✓	pep
38.03	8.18		44.30		675.00		H+							✓	pep
38.03	8.18		44.30		691.04		H+							✓	pep
38.03	8.18		44.30		669.09		H+					1.54	0.26		
38.03	8.18		44.30		653.10		?					0.67	0.11		

S6 Table continued.

RT (min)	GU experi-mental	GU std.	RP experi-mental	RP std.	*m/z* experi-mental	*m/z* theoretical	*m/z* ion form	MS	Composition (+PA)		Glycan structure	Quantity ratio % for top	Quantity ratio % for whole	MS2	Note
38.03	8.18		44.30		956.12		?					0.00	0.00		
39.98	8.73		46.37		917.15		H+							✓	pep
39.98	8.73	9.71	46.37		941.18	941.36	2H+	1880.70	H6Hn4	Ln-**M5A**Bs	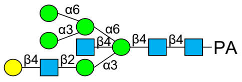	1.87	0.31		
41.63	9.22		48.13		956.14		H+							✓	pep
41.63	9.22	9.09	48.13		985.37	985.37	2H+		N1H6Hn3	3N-Ln-**M5A**	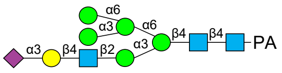	3.67	0.61	✓	
41.63	9.22		48.13		918.10		H+							✓	pep
42.34	9.44	9.54	48.88		932.38	933.36	2H+	1864.71	H5Hn4dH1	**BI**F(6)/ga	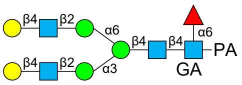			✓	
42.34	9.44	9.26	48.88		823.09	823.31	2H+	1644.61	N1H4Hn3	3N-**MO1**	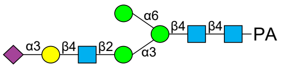	3.80	0.64	✓	
42.83	9.59	9.43	49.40		904.20	904.34	2H+	1806.67	N1H5Hn3	3N-Ln-**M4C**	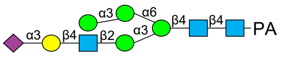	0.63	0.11	✓	
42.83	9.59	9.54	49.40		932.37	933.36	2H+	1864.71	H5Hn4dH1	**BI**F(6)/ga					
43.35	9.75	9.71	49.95		889.91	889.67	3H+	2665.97	N2H6Hn5	33N-**TR**	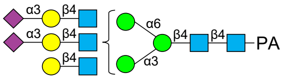	1.12	0.19	-	
43.35	9.75		49.95		980.13		H+					0.00	0.00	✓	pep
44.48	10.11	10.05	51.15		860.44	860.33	2H+	1718.65	H5Hn4dH1	**BI**	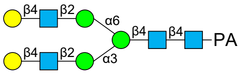	6.88	1.15	✓	
44.48	10.11		51.15		693.09		2H+							✓	pep
44.48	10.11		51.15		942.09		H+					0.26	0.04		
44.48	10.11	10.15	51.15		973.40	973.40	H+	972.39	H2Hn2dH1	**M2B**F(6)					
44.48	10.11		51.15		957.14		H+							✓	pep
44.99	10.28	10.15	51.69		973.42	973.40	H+	972.39	H2Hn2dH1	**M2B**F(6)	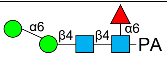	18.23	3.05	✓	
44.99	10.28	10.15	51.69	50.51	1135.46	1135.45	H+	1134.44	H3Hn2dH1	**M3B**F(6)	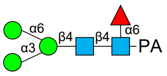	9.82	1.65	✓	
44.99	10.28	10.12	51.69		1115.66	1115.93	2H+	2229.84	H6H5dH1	**TR**F(6)	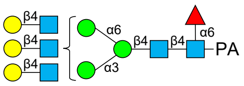	0.41	0.07	✓	
44.99	10.28		51.69		693.15		H+							✓	pep
46.36	10.73		53.04		986.92	986.70	3H+	2957.07	N3H6Hn5	trN-**TR**	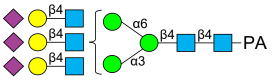	2.78	0.47		
46.36	10.73		53.04		693.15		H+							✓	pep
46.36	10.73		53.04		957.16		H+							✓	pep
46.36	10.73	10.95	53.04		1078.19	1078.91	2H+	2155.80	N1H5Hn4dH1	03N-**BI**F(6)/ga					
46.62	10.81	10.91	53.30		1034.94	1034.90	2H+	2067.79	H5Hn5dH1	F(3)-**BI**Bs	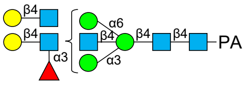	14.09	2.36		Le^x
47.62	11.15	10.95	54.28		1077.94	1078.91	2H+	2155.80	N1H5Hn4dH1	03N-**BI**F(6)/ga	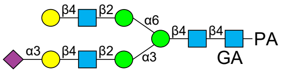			✓	
47.62	11.15		54.28		980.10		H+							✓	pep
48.49	11.45		55.14		981.12		H+							✓	pep
48.49	11.45		55.14		941.11		H+							✓	pep
48.49	11.45		55.14		709.11		H+							✓	pep
48.49	11.45	11.15	55.14		924.90	924.85	2H+	1847.69	N1H4Hn4	03N-**BI**-Ga2	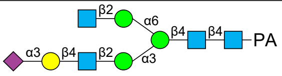	0.06	0.01		
50.17	12.03	11.68	56.79	56.09	771.34	771.31	2H+	1540.60	H3Hn4dH1	**AG12**F(6)	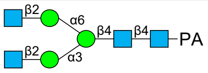	3.57	0.60	✓	
50.17	12.03	11.65	56.79		1223.49	1224.46	2H+	2446.90	N2H5Hn4dH1	33N-**BI**F(6)/ga	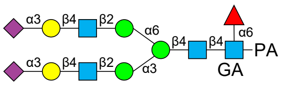	2.92	0.49	✓	

S6 Table continued.

RT (min)	GU experi-mental	GU std.	RP experi-mental	RP std.	m/z experi-mental	m/z theoretical	ion form	MS	Composition (+PA)	Glycan structure		Quantity ratio % for top	Quantity ratio % for whole	MS^2	Note
50.17	12.03		56.79		1261.40	1261.48	2H+	2520.94	N1H6Hn5dH1	N-**TR**F(6)	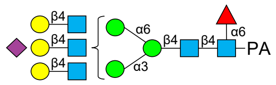	1.46	0.24	✓	
50.17	12.03		56.79		980.10		H+							✓	pep
50.17	12.03		56.79		611.18		?					0.39	0.06		
50.17	12.03	11.98	56.79		1006.35	1005.88	2H+	2009.75	N1H5Hn4	30N-**BI**					
50.69	12.22	11.98	57.30		1005.82	1005.88	2H+	2009.75	N1H5Hn4	30N-**BI**	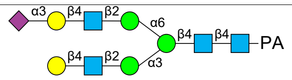	19.97	3.34	✓	
50.69	12.22		57.30		996.14		H+							✓	pep
50.69	12.22		57.30		1028.06		?					0.13	0.02		
51.77	12.61		58.36		693.08		H+							✓	pep
51.77	12.61	12.27	58.36		1005.86	1005.88	2H+	2009.75	N1H5Hn4	03N-**BI**	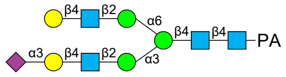	1.66	0.28	✓	
51.77	12.61		58.36		841.25	841.32	3H+	2520.94	N1H6Hn5dH1	N-**TR**F(6)	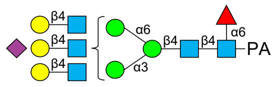	0.55	0.09	✓	
52.22	12.77	12.59	58.81		1224.85	1224.46	2H+	2446.90	N2H5Hn4dH1	33N-**BI**F(6)/epi	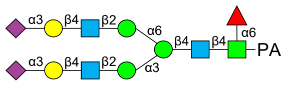	5.27	0.88	✓	
52.22	12.77		58.81		965.13		H+							✓	pep
53.13	13.10		59.70		997.14	997.88	2H+	1993.75	N1H4Hn4dH1	N-**BI**F(6)-Ga	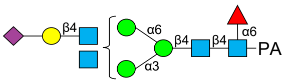	0.47	0.08		
53.13	13.10		59.70		980.16		H+							✓	pep
53.13	13.10		59.70		965.08		H+							✓	pep
53.13	13.10		59.70		957.05		H+							✓	pep
54.00	13.42		60.56		631.10		2H+							✓	pep
54.00	13.42		60.56		938.33	938.35	3H+	2812.03	N2H6Hn4dH1	dN-Ln-**BI**F(6)	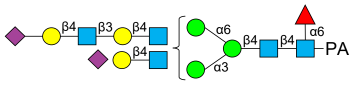	2.74	0.46	✓	N-dLn
54.55	13.63	13.61	61.10	60.83	933.37	933.36	2H+	1864.71	H5Hn4dH1	**BI**F(6)	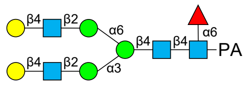	20.95	3.51	✓	
54.55	13.63		61.10		502.73		2H+							✓	pep
54.55	13.63		61.10		611.69		?					2.24	0.37		
54.55	13.63		61.10		1004.17		H+							✓	pep
55.50	13.99	11.79	62.03	60.83	623.25		2H+							✓	pep
55.50	13.99		62.03		933.46	933.36	2H+	1864.71	H5Hn4dH1	**BI**F(6)					
56.34	14.31	14.13	62.86	62.56	1151.28	1151.43	2H+	2300.84	N2H5Hn4	33N-**BI**	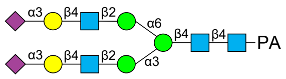	22.42	3.76	✓	
56.34	14.31	14.36	62.86		1035.58	1035.38	3H+	3103.13	N3H6Hn5dH1	trN-F-**TR**	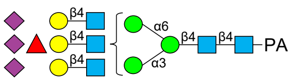	12.85	2.15	✓	
56.34	14.31		62.86		631.22		2H+							✓	pep
56.34	14.31		62.86		623.14		2H+							✓	pep
56.34	14.31		62.86		643.15		?							✓	pep
58.18	15.04		64.67		643.14		?					0.76	0.13		
58.18	15.04		64.67		1004.11		H+							✓	pep
58.18	15.04	14.99	64.67		986.96	986.70	3H+	2957.07	N3H6Hn5	trN-**TR**	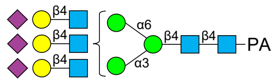	0.06	0.01		
59.03	15.38	15.35	65.50		986.71	986.70	3H+	2957.07	N3H6Hn5	trN-**TR**	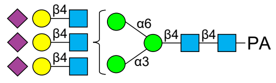	4.18	0.70		
59.03	15.38		65.50		1060.18	1060.06	3H+	3177.16	N2H7Hn6dH1	dN-**TE**F(6)	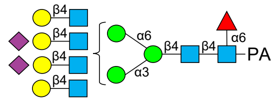	2.03	0.34		
61.42	16.38		67.85		643.26		2H+							✓	pep
61.42	16.38		67.85		651.11		2H+					1.69	0.28		

S6 Table continued.

RT (min)	GU experi-mental	GU std.	RP experi-mental	RP std.	*m/z* experi-mental	*m/z* theoretical	ion form	MS	Composition (+PA)	Glycan structure	Quantity ratio % for top	Quantity ratio % for whole	MS[2]	Note
61.42	16.38		67.85		1157.18	1157.09	3H+	3468.26	N3Hn7H6dH1	trN-F-**TE** 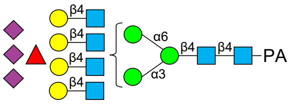	0.56	0.09		
61.42	16.38		67.85		631.38		2H+				0.21	0.03		
62.04	16.65	17.28	68.41		1078.97	1078.91	2H+	2155.80	N1H5Hn4dH1	03N-**BIF**(6) 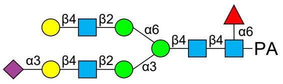	37.94	6.35	✓	
62.04	16.65	16.39	68.41		1157.24	1157.09	3H+	3468.26	N3H7Hn6dH1	trN-**TEF**(6) 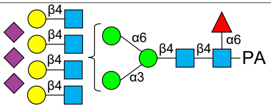	0.41	0.07		
62.04	16.65		68.41		1060.12	1060.06	3H+	3177.16	N2H7Hn6dH1	dN-dLn-**BIF**(6) 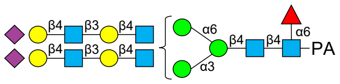	0.18	0.03		
62.63	16.91	17.66	68.94		1078.94	1078.91	2H+	2155.80	N1H5Hn4dH1	30N-**BIF**(6) 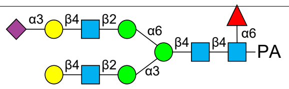	5.75	0.96	✓	
63.20	17.17		69.46		1157.06	1157.09	3H+	3468.26	N3Hn7H6dH1	trN-F-**TE** 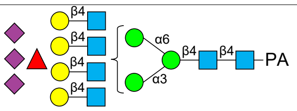	2.90	0.49		
63.20	17.17		69.46		651.12		?				2.05	0.34		
63.20	17.17		69.46		1078.94	1078.91	2H+	2155.80	N1H5Hn4dH1	30N-**BIF**(6)				
64.25	17.65		70.40		1232.56	1232.45	2H+	2462.89	Ng1N1H5Hn4dH1	Ng-N-**BIF**(6) 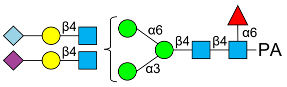	1.74	0.29	✓	Ng-Ln, N-Ln
64.25	17.65		70.40		635.06		2H+						✓	pep
65.13	18.07		71.20		1254.33	1254.13	3H+	3759.35	N4H7Hn6dH1	teN-Ln-**TRF**(6) 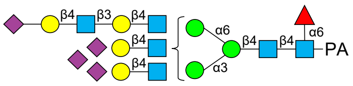	8.85	1.48	✓	N-dLn
65.13	18.07		71.20		635.25		2H+						✓	pep
65.13	18.07		71.20		643.12		2H+						✓	pep
65.13	18.07	17.70	71.20		1157.23	1157.09	3H+	3468.26	N3Hn7H6dH1	trN-**TEF**(6) 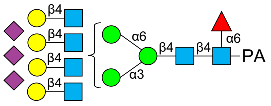	1.65	0.28		
66.46	18.73		72.40		655.26		?				1.07	0.18		
66.46	18.73		72.40		643.10		3H+				0.86	0.14		
66.46	18.73		72.40		1278.78		3H+				0.29	0.05		
66.46	18.73	18.74	72.40		1157.23	1157.09	3H+	3468.26	N3Hn7H6dH1	trN-**TEF**(6) 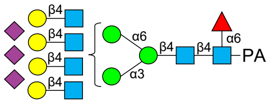	0.08	0.01		
67.46	19.25	19.41	73.31	73.91	1224.19	1224.46	2H+	2446.90	N2H5Hn4dH1	33N-**BIF**(6) 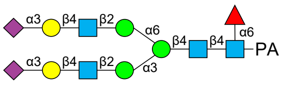	42.42	7.10	✓	
67.46	19.25		73.31		651.22		3H+				1.51	0.25		
67.46	19.25		73.31		663.17		3H+				0.56	0.09		
69.56	20.42		75.21		1375.82	1375.53	2H+	2749.05	N1H4Hn7dH2	N-F-dLdn-**MOF**(6) 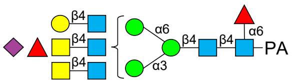	3.33	0.56		Ldn
71.80	21.81	22.65	77.23		938.40	938.35	3H+	2812.03	N2H6Hn4dH1	33N-Ln-**BIF**(6) 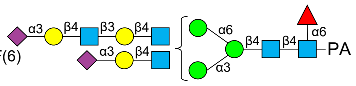	3.25	0.54		3N-dLn
72.95	22.59		78.27		655.22		H+				1.01	0.17		
72.95	22.59		78.27		1123.71		?				0.16	0.03		
73.72	23.14		78.97		655.13		3H+				3.34	0.56		
73.72	23.14		78.97		775.94		3H+				0.61	0.10		
73.72	23.14	23.07	78.97		938.24	938.35	3H+	2812.03	N2H6Hn4dH1	33N-Ln-**BIF**(6) 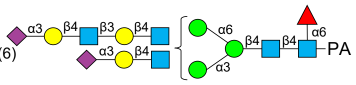	0.53	0.09		3N-dLn
74.27	23.55		79.47		663.26		H+				0.47	0.08		

S6 Table continued.

RT (min)	GU		RP		*m/z*			MS	Composition (+PA)		Glycan structure	Quantity ratio		MS^2	Note
	experi-mental	std.	experi-mental	std.	experi-mental	theoretical	ion form					% for top	% for whole		
75.73	24.70		80.79		1035.53	1035.38	3H+	3103.13	N3H6Hn5dH1	trN-F-**TR**	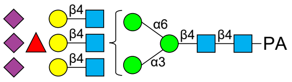	3.06	0.51		
75.73	24.70		80.79		655.66		3H+					2.25	0.38		
75.73	24.70		80.79		783.85		2H+					0.62	0.10		
76.85	25.67		81.80		659.19		H+							✓	pep
76.85	25.67		81.80		783.78		2H+					0.11	0.02		
76.85	25.67		81.80		667.18		3H+					0.07	0.01		
76.85	25.67		81.80		635.73		2H+					0.07	0.01		
77.69	26.43		82.56		667.16		3H+					1.13	0.19		
